# Spatial patterns and temporal trends in stillbirth, neonatal, and infant mortality: an exploration of country-level data from 2000 to 2021

**DOI:** 10.7189/jogh.15.04034

**Published:** 2025-02-21

**Authors:** Andrea Maugeri, Martina Barchitta, Gaia Schillaci, Antonella Agodi

**Affiliations:** 1 Department of Medical and Surgical Sciences and Advanced Technologies ‘GF Ingrassia’, University of Catania, Catania, Italy; 2 Department of Economics and Business, University of Catania, Catania, Italy

## Abstract

**Background:**

Despite significant progress in improving child survival and health, substantial disparities persist and are being increasingly threatened by a complex and dynamic global environment. In this ecological study, we investigated spatial patterns, temporal trends, and key determinants of disparities in stillbirth, neonatal, and infant mortality rates across 195 countries from 2000 to 2021.

**Methods:**

We sourced our data from two publicly available databases: the United Nations Children’s Fund Data Warehouse for mortality indicators and the World Bank for World Development Indicators. We conducted spatial analysis to assess spatial autocorrelation and identify geographical clusters of countries and applied joinpoint regression to evaluate temporal trends in mortality indicators, reported as annual percentage change. We also used forward regression analysis to determine the primary indicators influencing stillbirth, neonatal, and infant mortality rates.

**Results:**

The average stillbirth rate in 2021 was 10.9 per 1000 total births, a significant decrease from 16.3 per 1000 in 2000. Neonatal mortality also declined from 23.6 to 13.5 per 1000 live births during the same period, while infant mortality dropped from 45.0 to 22.5 per 1000 live births. Despite these improvements, spatial analysis showed notable positive spatial autocorrelations for stillbirth, neonatal, and infant mortality rates, indicating that high mortality rates were geographically clustered, particularly in African countries forming hot-spot clusters. Conversely, developed countries in Europe and Asia formed cold-spot clusters characterised by low mortality indicators. Some countries, identified as low-high or high-low clusters, stood out due to dissimilar mortality rates compared to their neighbours, warranting further investigation. Key determinants of mortality rates included the young-age dependency ratio, prevalence of undernourishment, the percentage of women aged 15 and older living with HIV, the incidence of tuberculosis, and the adolescent fertility rate – all of which showed a positive association with higher mortality rates. In contrast, factors such as the use of at least basic sanitation services, mean years of schooling, and government effectiveness had an inverse relationship, contributing to lower mortality rates.

**Conclusions:**

By identifying hotspots and outliers, this study highlights the need for targeted health interventions and efficient resource allocation. This approach ensures that efforts are strategic and impactful, focussing on areas with the greatest need.

The past few decades have witnessed outstanding progress in child survival, health, and well-being, alongside significant strides in ensuring children’s rights. The chances of a child surviving to their fifth birthday have improved considerably over the last 30 years. More specifically, the global number of under-five deaths has decreased from 12.8 million in 1990 to 4.9 million in 2022, representing a nearly 60% reduction in the mortality rate [1]. Neonatal deaths have also declined, from 5.2 million in 1990 to 2.3 million in 2022 [1]. However, the reduction in neonatal mortality has been slower compared to post-neonatal under-five mortality [2]. Each day, approximately 6300 newborns die, representing nearly 47% of all under-five deaths. If current trends persist, 59 countries will not meet the Sustainable Development Goal (SDG) target for under-five mortality by 2030, and over 60 will miss the neonatal mortality target [3].

These achievements are now under severe threat from an increasingly complex and dynamic global environment. New and ongoing challenges such as the COVID-19 pandemic [4], antimicrobial resistance [5], climate change [6], environmental degradation [7], conflicts [8], population migration [9,10], economic instability [11], exploitative commercial practices [12], and declining investments from bilateral, multilateral, and private-sector sources [13] pose substantial barriers. Significant disparities also remain, with children facing different survival chances based on their birthplace. For instance, a child born in sub-Saharan Africa is 11 times more likely to die in the first month of life than one born in Australia and New Zealand [14]. Indeed, Sub-Saharan Africa and Southern Asia accounted for more than 80% of the 4.9 million under-five deaths in 2022 [14].

In 2021, the United Nations Children’s Fund (UNICEF) concluded its 2018–21 strategic plan and launched a new, ambitious one aimed at accelerating progress toward the 2030 SDG targets [15]. Despite significant advances in child survival driven by global health initiatives, substantial disparities in stillbirth, neonatal, and infant mortality persist worldwide. Here we present the findings from our analysis of spatial and temporal mortality patterns across 195 countries from 2000 to 2021, focussing on identifying mortality trends and examining the socioeconomic determinants that influence these rates. Unlike previous studies, which often concentrate on single regions or specific indicators [16–18], our research offers a broad, updated global perspective by applying spatial autocorrelation to identify clusters within an extensive data set, thus delivering an integrated view of stillbirth, neonatal, and infant mortality trends over multiple decades.

## METHODS

### Study design and data source

We conducted this ecological analysis using country-level data from two publicly available databases: the UNICEF Data Warehouse for mortality indicators [19] and the World Bank’s World Development Indicators [20]. We selected these sources due to their extensive country coverage, standardised reporting protocols, and consistency across years, facilitating robust cross-country comparisons. This analysis included data from 195 countries over the period 2000 to 2021. As it relies on publicly accessible, aggregated, and de-identified data, this study was exempt from ethical review.

### Dependent variables

The analysis focussed on three indicators reflecting maternal and child health, health care quality and policies, the safety of maternity services and socioeconomic conditions in a given region or country: stillbirth rate, which is the number of babies born with no sign of life at 28 weeks or more of gestation per 1000 total births; neonatal mortality rate, which is the probability of dying during the first 28 days of life, expressed per 1000 live births; infant mortality rate, which is the probability of dying between birth and exactly 1 year of age, expressed per 1000 live births [1].

Nationally representative stillbirth rates are derived by the United Nations Inter-agency Group for Child Mortality Estimation and its Core Stillbirth Estimation Group using a model-based approach. The input data are sourced from administrative systems (*e.g.* vital registration systems, birth or death registries), health management information systems, household surveys, and population studies. Data recorded using alternative definitions of stillbirth are standardised to the 28-week definition before model fitting. A hierarchical Bayesian model with spline smoothing is employed to estimate the stillbirth rate for each country-year, incorporating covariates such as socioeconomic, demographic, and biomedical factors, perinatal outcome markers, and indicators of access to health care [1].Nationally representative estimates of neonatal and infant mortality are derived from multiple sources, including civil registration, censuses, and sample surveys. Neonatal mortality rates are calculated using a statistical model that incorporates available national data and estimated under-five mortality rates [1]. Infant mortality rates are generated either by applying a statistical model or by transforming under-five mortality rates based on model life Tables [1].

### Independent variables

Our analysis included 20 independent variables measured at the country level, selected from the 1492 World Development Indicators available for 2021 [20] using a data-driven approach (Table S1 in the **Online Supplementary Document**). We initially excluded variables with more than 20% missing values; for the remaining ones, we imputed missing values using the column mean. Next, we assessed the correlation between the indicators and the three dependent variables separately using Spearman's rank correlation coefficient. Indicators with a correlation coefficient of 0.7 or higher (in absolute value) were retained for further analysis. For variables containing redundant information (*e.g.* gross domestic product vs gross national product), only one was retained. We further excluded variables representing broader domains, such as the Human Development Index and Universal Health Coverage. The selected indicators were normalised as rates, percentages, or standardised measures, ensuring they are not influenced by population size and are directly comparable across countries.

### Analysis of spatial patterns

We examined spatial patterns in stillbirth, neonatal, and infant mortality rates using spatial analysis via the ‘PySAL’ library in Python, version XXX (Python Software Foundation, Amsterdam, The Netherlands). The country served as the unit of analysis, and a distance-based spatial weight matrix was created with a threshold distance of 4270 km, utilising a geographic coordinate shapefile sourced from the Union of International Associations [21]. We validated the suitability of this spatial weight by evaluating the symmetry of the connectivity histogram and connectivity map. All 195 countries were interconnected, facilitating the assessment of spatial dependence. We generated quantile maps to illustrate the spatial distribution of stillbirth, neonatal, and infant mortality rates worldwide. We assessed the local spatial autocorrelation using the local Moran’s I index, which ranges from +1 to −1, indicating strong positive autocorrelation (perfect clustering) to negative autocorrelation (perfect dispersion). Positive and negative indices suggest the clustering of neighbourhoods with similar and dissimilar values across geographical space compared to random distribution, while zero implies no autocorrelation (*i.e.* perfect randomness). We then created local indicator of spatial autocorrelation (LISA) cluster maps to highlight hotspots and cold spots of statistically significant spatial clusters of neighbouring countries with high and low mortality rates, respectively. In LISA clustering, five categories were delineated to represent various types of spatial autocorrelation: high-high (HH) clusters, where countries with high values were surrounded by others with high values; high-low (HL) clusters, where countries with high values were surrounded by those with low values; low-high (LH) clusters, where countries with low values were surrounded by those with high values; low-low (LL) clusters, where countries with low values were surrounded by others with low values; and the ‘not significant’ category, indicating no evidence of significant spatial autocorrelation for the considered country (*i.e.* its value is not significantly correlated with the values in surrounding countries).

### Analysis of temporal trends

We analysed temporal trends in stillbirth, neonatal, and infant mortality rates using joinpoint regression through the Joinpoint Regression Program, version 4.9.0.0 (Statistical Research and Applications Branch, National Cancer Institute, Maryland, USA). This method identifies significant changes in trends by pinpointing joinpoints (ie, time points where trends shift) and estimates the regression function between these points [22,23]. In our study, we applied joinpoint regression to the logarithmically transformed dependent variable, assuming uncorrelated errors. The model determined the number and placement of joinpoints using a grid search method, ranging from 0 to 5 joinpoints [24]. The optimal model was selected by minimising the sum of squared errors. Model selection was based on a permutation test with 5000 permutations and a significance level of 0.05, with the Bonferroni adjustment applied to control the overall type I error rate.

The analysis produced annual percent changes (APCs) and their 95% confidence intervals (95% CIs), reflecting the average yearly percentage change between identified joinpoints. Furthermore, we calculated the average annual percentage change (AAPC) to represent the overall trend across the specified period 2000-2021. This metric, derived as a weighted average of the APCs from the joinpoint model, provides a comprehensive view of the average APCs over multiple years, accounting for any shifts in trends. As outlined previously, we also applied spatial analysis to the AAPCs calculated for each country.

To facilitate meaningful comparisons of temporal trends, we grouped countries into UNICEF reporting regions: East Asia and the Pacific, Eastern and Southern Africa, Eastern Europe and Central Asia, Europe and Central Asia, Latin America and the Caribbean, the Middle East and North Africa, North America, South Asia, Sub-Saharan Africa, West and Central Africa, and Western Europe. We conducted pairwise comparisons to assess whether the temporal trends, each modelled using joinpoint regression, were parallel [25]. This test of parallelism evaluates if the regression mean functions exhibit parallel trends across different country groups, as detailed elsewhere [25].

### Statistical analysis

We used descriptive analysis to explore the distribution of both dependent and independent factors, summarising normally distributed variables as means (x̄) and standard deviations (SDs)) and skewed variables as medians and interquartile ranges. We employed linear regression models using forward entry as the variable selection method to identify the primary indicators influencing stillbirth, neonatal, and infant mortality rates. We selected variables based on criteria where the probability of F-to-enter was ≤0.05 and the probability of F-to-remove was ≥0.10. This approach allowed for the identification of key socioeconomic and health-related indicators associated with mortality rates, ensuring a data-driven selection of predictors based on their statistical significance. We set statistical significance at *P* < 0.05 and reported our findings as β coefficients and standard errors. We used SPSS, version 26.0 (IBM, Armonk, New York, USA) for all statistical analyses to ensure consistency and reliability across findings.

## RESULTS

**Figure 1:**
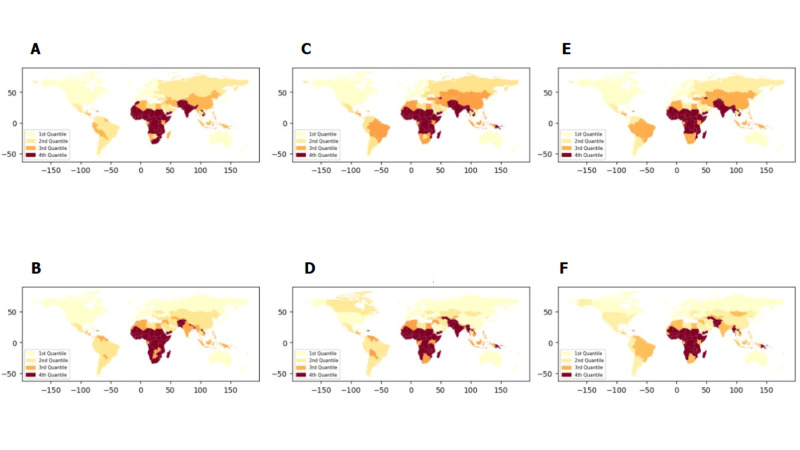
Quantile maps of stillbirth, neonatal, and infant mortality rates in 2000 and 2021. **Panels A and B.** Stillbirth rates in 2021 and 2000. **Panels C and D.** Neonatal mortality rates in 2021 and 2000.**Panels E and F.** Infant mortality rates in 2021 and 2000.

The country-level rates of stillbirth, neonatal, and infant mortality for 2000 and 2021 differed across regions (**Figure 1**). In 2021, the average stillbirth rate was 10.9 (SD = 7.7) per 1000 total births, with rates ranging from 2.7 in Western Europe to 23.0 in West and Central Africa. This marked a significant improvement from 2000, when the average stillbirth rate was 16.3 (SD = 10.6) per 1000 total births, with the lowest rate in North America (3.3) and the highest in South Asia (32.1). The average neonatal mortality rate in 2021 was 13.5 (SD = 10.5) per 1000 live births, ranging from 2.3 per 1000 in Western Europe to 30.4 in West and Central Africa. This was an improvement from the 2000 average neonatal mortality rate of 23.6 (SD = 15.6) per 1000 live births, with the lowest rate in Western Europe (3.4) and the highest in South Asia (46.4). In 2021, the average infant mortality rate was 22.5 (SD = 19.6) per 1000 live births, with rates ranging from 3.1 per 1000 in Western Europe to 60.9 in West and Central Africa. This represented an improvement from 2000, when the average infant mortality rate was 45.0 (SD = 34.3) per 1000 live births, with the lowest rate in Western Europe (5.1) and the highest in West and Central Africa (100.2).

**Figure 2:**
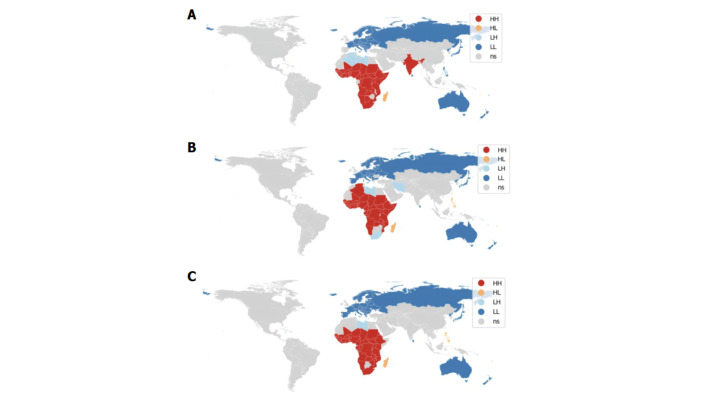
LISA cluster maps of stillbirth, neonatal, and infant mortality rates in 2021. Maps highlight HH, HL, LH, and LL clusters. **Panel A.** Clusters for stillbirth rates. **Panel B.** Clusters for neonatal mortality rates. **Panel C.** Clusters for infant mortality rates. HH – high-high, HL – high-low, LH – low-high, LL – low-low, NS – no evidence of significant spatial autocorrelation.

The Moran's I indexes showed positive spatial autocorrelations for stillbirth (Moran’s I = 0.837), neonatal (Moran’s I = 0.811), and infant mortality rates (Moran’s I = 0.853) across the 195 countries studied in 2021 (**Figure 2**). For stillbirth rates, the LISA cluster map (**Figure 2**, Panel A) revealed HH clusters involving 35 African countries and India (n/N = 36/195, 18.46%). Conversely, the LL clusters, consisting of 58 countries (29.74%), had low stillbirth rates and were primarily located in Europe, as well as in Russia, North Korea, Japan, the Philippines, Australia, and New Zealand. Apart from these hot and cold spots, outliers were identified as LH and HL clusters. Specifically, Libya and Algeria were identified as an LH outliers (1.03%), while 13 countries were identified as HL outliers (6.67%). The LISA cluster map for neonatal mortality rates (**Figure 2**, Panel B) indicated that 35 African countries formed HH clusters (17.95%). South Africa and Libya were exceptions, along with Iran, forming LH clusters instead (1.54%). LL clusters, encompassing 64 countries (32.82%), were predominantly situated in Europe, along with Russia, North Korea, Japan, Australia, and New Zealand. Additionally, HL clusters were observed in 11 countries (5.64%), characterised by high values surrounded by regions with low values. For infant mortality rates (**Figure 2**, Panel C), HH clusters were detected in 36 African countries (18.46%), while Libya was a LH outlier. LL clusters, comprising 66 countries (33.85%), were mainly situated in Europe, Russia, North Korea, Japan, Australia, and New Zealand. Similar to the other maps, HL clusters were observed in 12 countries (6.15%), including Madagascar and the Philippines.

We initially analysed and compared temporal trends in stillbirth, neonatal, and infant mortality rates at the regional level (Figures S1–3 in the **Online Supplementary Document**). Each region exhibited a distinct temporal trend, with no parallelism found in any pairwise comparison (*P*-values <0.05). We observed most significant reductions from 2000 to 2021 in East Asia and the Pacific for stillbirth rates (AAPC = −3.3; 95% CI = −3.5, −3.1) and in Eastern Europe and Central Asia for both neonatal (AAPC = −5.0; 95% CI = −5.2, −4.8) and infant mortality rates (AAPC = −5.2; 95% CI = −5.2, −5.1). In contrast, North America reported the slowest declines across all rates: stillbirth (AAPC = −1.0; 95% CI = −1.3, −0.6), neonatal mortality (AAPC = −1.4; 95% CI = −1.6, −1.1), and infant mortality (AAPC = −1.3; 95% CI = −1.3, −1.2).

**Figure 3:**
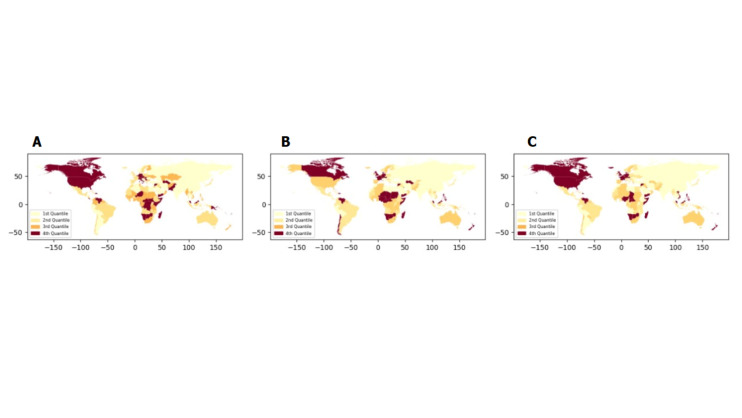
Quantile maps of AAPCs from 2000 to 2021 for stillbirth, neonatal, and infant mortality rates. **Panel A.** Stillbirth rates. **Panel B.** Neonatal mortality rates. **Panel C.** Infant mortality rates.

We then analysed temporal trends in stillbirth, neonatal, and infant mortality rates at the country level to identify joinpoints, segment lengths, and their corresponding APCs (Tables S2–4 in the **Online Supplementary Document**). We also calculated the overall AAPCs for the period from 2000 to 2021 (**Figure 3**). For stillbirth rates, we observed the lowest AAPCs in the Maldives, China, and North Macedonia, and the highest AAPCs in Saint Vincent and the Grenadines, Botswana, and Dominica (**Figure 3**, Panel A). For neonatal mortality rates, the lowest AAPCs were recorded in Montenegro, China, and Belarus, while the highest were seen in Venezuela, Dominica, and Botswana (**Figure 3**, Panel B). Regarding infant mortality rates, Montenegro, the Maldives, and China had the lowest AAPCs, while Fiji, Saint Lucia, and Dominica had the highest (**Figure 3**, Panel C).

**Figure 4:**
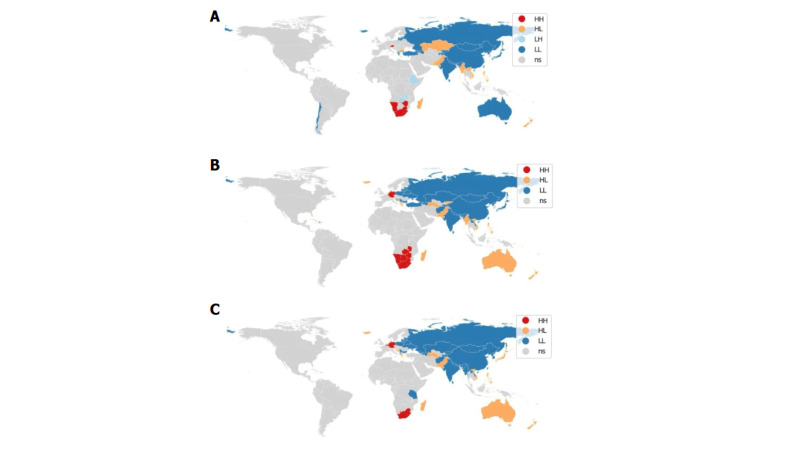
LISA cluster maps of AAPCs from 2000 to 2021 for stillbirth, neonatal, and infant mortality rates. Maps highlight HH, HL, LH, and LL clusters. **Panel A.** Clusters for stillbirth rates. **Panel B.** Clusters for neonatal mortality rates. **Panel C.** Clusters for infant mortality rates. HH – high-high, HL – high-low, LH – low-high, LL – low-low, NS – no evidence of significant spatial autocorrelation.

The Moran's I indexes showed positive spatial autocorrelations in AAPCs for stillbirth (Moran’s I = 0.328), neonatal (Moran’s I = 0.390), and infant mortality rates (Moran’s I = 0.346) across the 195 countries studied in 2021 (**Figure 4**). The LISA cluster map for AAPCs in stillbirth rates (**Figure 4**, Panel A) revealed interesting patterns across different regions. Namibia, South Africa, Zimbabwe, and Austria formed HH clusters, indicating ‘hot spots’ with concentrated high values. In contrast, Madagascar, New Zealand, Vietnam, Myanmar, Pakistan, Kazakhstan, and Bulgaria exhibited HL clusters, where high values were surrounded by regions with low values. Zambia and Ethiopia were identified as LH clusters, showing low values amid high-value regions. Additionally, Chile, Iceland, Greece, Turkey, Lithuania, Latvia, Russia, Azerbaijan, Armenia, Georgia, Mongolia, China, North Korea, Japan, Taiwan, India, and Australia formed LL clusters, or ‘cold spots’, with consistently low values for the variable of interest. The LISA cluster map for neonatal mortality (**Figure 4**, Panel B) showed spatial patterns across countries: Namibia, Botswana, South Africa, Zimbabwe, Zambia, and Germany emerge as HH clusters. These areas exhibited high values for AAPCs related to neonatal mortality rates, and were also surrounded by neighbouring countries sharing similar high values. Conversely, countries like Madagascar, Australia, New Zealand, Vietnam, the Philippines, Myanmar, Pakistan, Turkmenistan, Kyrgyzstan, Iceland, and Greece stood out with HL clusters. Meanwhile, LL clusters encompassed Russia, Kazakhstan, Mongolia, China, Japan, North Korea, Taiwan, India, Laos, Nepal, Afghanistan, Turkey, Bulgaria, Ukraine, Belarus, Poland, Lithuania, Latvia, and Estonia. The LISA cluster analysis revealed further patterns in AAPCs for infant mortality rates across different countries (**Figure 4**, Panel C). South Africa, Germany, and Belgium were identified as HH clusters. Madagascar, Australia, New Zealand, Vietnam, the Philippines, Japan, Pakistan, Turkmenistan, Iceland, and Greece were highlighted in HL clusters. Meanwhile, Russia, Kazakhstan, Mongolia, China, North Korea, South Korea, Taiwan, India, Laos, Nepal, Afghanistan, Tanzania, Bulgaria, Ukraine, Belarus, Poland, Lithuania, Latvia, and Estonia formed LL clusters.

Finally, we evaluated which World Development Indicators were associated with the examined mortality rates (**Table 1**). For the stillbirth rate, we found a positive association with the young-age dependency ratio, prevalence of undernourishment, percentage of women aged ≥15 living with HIV, and incidence of tuberculosis. Consequently, the stillbirth rate increased with higher values of these indicators. Conversely, we observed negative associations with mean years of schooling and government effectiveness. We identified similar associations for the neonatal mortality rate. For the infant mortality rate, we found a positive association with the young-age dependency ratio, incidence of tuberculosis, and adolescent fertility rate, and negative associations with mean years of schooling and the percentage of people using at least basic sanitation services.

**Table 1:** Forward regression analyses of the association between World Development Indicators and mortality rates

Dependent variable	Indicator	β coefficient	Standard error	*P*-value
**Stillbirth rate**	Age dependency ratio, young (% of working-age population)	0.089	0.022	<0.001
	Mean years of schooling	−0.642	0.132	<0.001
	Prevalence of undernourishment (% of population)	0.097	0.034	0.005
	Women's share of population ages ≥15 living with HIV (%)	0.074	0.022	0.001
	Incidence of tuberculosis (per 100 000 people)	0.006	0.002	0.007
	Government effectiveness	−1.015	0.376	0.008
**Neonatal mortality rate**	Age dependency ratio, young (% of working-age population)	0.160	0.032	<0.001
	Mean years of schooling	−0.794	0.196	<0.001
	Incidence of tuberculosis (per 100 000 people)	0.011	0.003	0.001
	Prevalence of undernourishment (% of population)	0.108	0.051	0.035
	Women's share of population ages ≥15 living with HIV (%)	0.080	0.033	0.018
	Government effectiveness	−1.131	0.558	0.044
**Infant mortality rate**	Age dependency ratio, young (% of working-age population)	0.278	0.065	<0.001
	People using at least basic sanitation services (% of population)	−0.126	0.048	0.009
	Incidence of tuberculosis (per 100 000 people)	0.023	0.005	<0.001
	Adolescent fertility rate (births per 1000 women ages 15–19)	0.097	0.032	0.003
	Mean years of schooling	−0.969	0.321	0.003

## DISCUSSION

We conducted a comprehensive study of stillbirth, neonatal, and infant mortality rates across 195 countries from 2000 to 2021 and found significant trends and spatial patterns that underscore both progress and persistent disparities in global health. The reduction in stillbirth, neonatal, and infant mortality rates over the past two decades is a remarkable achievement. For instance, the average stillbirth rate in 2021 was 10.9 per 1000 total births, down from 16.3 per 1000 in 2000. This decrease reflects substantial improvements in prenatal and maternal health care. Similarly, neonatal mortality decreased from 23.6 to 13.5 per 1000 live births, and infant mortality from 45.0 to 22.5 per 1000 live births. These improvements indicate that global health initiatives, better health care infrastructure, and increased access to medical care have made a significant impact [26].

However, significant regional disparities remain despite this overall progress. In 2021, the stillbirth rate in Western Europe was as low as 2.7 per 1000, whereas it was 23.0 per 1000 in West and Central Africa. Similar disparities are seen in neonatal and infant mortality rates, highlighting the urgent need for targeted interventions in high-burden areas. The higher rates in regions like West and Central Africa could be attributed to factors such as limited access to health care, inadequate medical facilities, socioeconomic challenges, and higher prevalence of infectious diseases [27]. Conversely, the lower mortality rates observed in Western Europe and North America can be attributed to well-established health care systems, comprehensive public health policies, and higher socioeconomic status [17,28]. The latter is closely linked to healthier maternal lifestyles and better health choices [29–32].

The spatial analysis revealed significant positive spatial autocorrelations for stillbirth, neonatal, and infant mortality rates, indicating that countries with high mortality rates are geographically clustered together. This suggests that similar health outcomes are shared by neighbouring countries, reflecting regional health trends and common underlying factors. LISA cluster maps provide a detailed insight into spatial mortality patterns, delineating regions with varying mortality rates. ‘Hot spots’ (HH clusters) denote areas characterised by consistently high mortality rates, surrounded by similarly high-value regions, highlighting persistent health challenges. Conversely, ‘cold spots’ (LL clusters) indicate regions with consistently low mortality rates amidst surrounding low-value areas. The presence of these cold spots provides valuable insights into best practices that could be emulated by higher-mortality regions to improve their health outcomes [33,34]. In addition to hot and cold spots, the identification of HL and LH clusters in our analysis highlights countries that deviate significantly from the mortality patterns of their neighbouring regions. HL clusters represent countries with higher-than-expected mortality rates relative to adjacent countries, while LH clusters indicate areas with lower mortality in otherwise high-mortality regions. These outliers are crucial to understanding localised health inequities that might be masked in broader regional trends. The unique position of these outlier countries suggests that they face specific health challenges or possess strengths that differ from their neighbours, warranting more targeted, localised interventions. By focussing on these clusters, policymakers can better understand the unique context and needs of these outliers, rather than applying blanket interventions based solely on regional averages.

Although global averages have improved, the rates of decline vary significantly across regions, indicating that progress is uneven. For example, East Asia and the Pacific region showed the most substantial reductions in stillbirth rates, while Eastern Europe and Central Asia demonstrated notable improvements in both neonatal and infant mortality rates. These reductions can be attributed to several factors, including economic growth and development, public health initiatives, and advances in prenatal and neonatal care [35]. The slow progress in North America primarily arises from a more favourable starting point, rendering further improvements more challenging. However, issues such as unequal access to health care services, high prevalence of chronic health conditions among mothers, and persistent socioeconomic disparities cannot be disregarded [36].

Spatial analysis has identified geographic clusters in the rate changes from 2000 to 2021. Particularly noteworthy is the critical situation in several Southern African countries (such as Namibia, Botswana, South Africa, Zimbabwe, Zambia), which are characterised as hot spots with high mortality rates in 2021 and slower rates of decline over the preceding two decades. In contrast, countries in Central and South Asia, along with several in Eastern Europe and the Pacific region, show marked improvements, forming clusters that demonstrate the most significant reductions in mortality rates. These findings align with previous studies conducted at global, regional, and country levels [37–47]. Overall, it has been demonstrated that the reduction in mortality rates is far from uniform, varying significantly between and even within countries. While mortality rates typically follow a well-known socioeconomic gradient, the general finding is that changes in mortality and clustering often transcend regional boundaries [48]. These variations are more closely influenced by local and national factors, rather than solely by regional characteristics [47].

Our evaluation of the World Development Indicators uncovered significant ecological relationships with various factors. Specifically, our findings indicate that countries with higher proportions of young dependents, greater undernourishment, higher HIV prevalence among women, and more tuberculosis cases tend to have higher stillbirth rates. This underscores the significant impact of public health issues such as infectious diseases and malnutrition [49], alongside sociodemographic factors [44], on stillbirth occurrences. Conversely, we observed negative associations with mean years of schooling and government effectiveness. These results highlight the crucial role of education [50] and effective governance [51] in improving maternal and child health outcomes. Higher educational attainment often leads to better health literacy, economic opportunities, and access to health care services, all of which can contribute to reducing stillbirth rates [50]. Effective government policies and programmes, meanwhile, can enhance health care infrastructure, ensure the provision of essential services, and create supportive environments for maternal and child health [51].

We found similar associations for the neonatal mortality rate, reinforcing the need for comprehensive interventions that address these underlying issues to improve overall child survival rates. For the infant mortality rate, we observed positive associations with the young-age dependency ratio, incidence of tuberculosis, and adolescent fertility rate. These indicators suggest that high fertility rates among adolescents and high dependency ratios strain health care resources, potentially leading to increased infant mortality [9,10]. Furthermore, tuberculosis remains a significant health threat to infants, who are more vulnerable to infections due to their developing immune systems [52]. While the associations we identified here align with previous research on mortality determinants [27–32], this confirmation is valuable as it provides contemporary evidence across a large sample of countries over two decades. By using updated data, our study reinforces the importance of these factors in the current global health context, where socioeconomic conditions and health care infrastructure continue to evolve. In this way, it provides policymakers with robust evidence that highlights enduring health risks, thereby enabling more effective targeting of resources to address factors that continue to drive mortality disparities. In particular, policies aimed at improving education, health care access, nutrition, and sanitation can significantly reduce mortality rates [53]. Identifying the key factors associated with high mortality rates allows governments and organisations to allocate resources more efficiently to areas with the greatest need, ensuring that interventions have the maximum impact and can effectively address the root causes of high mortality rates [54]. Additionally, these indicators serve as benchmarks for monitoring and evaluating the effectiveness of public health interventions; tracking how they change over time could provide insights into the success of implemented strategies and guides future actions [54].

This study has several strengths. First, it offers comprehensive coverage of 195 countries, spanning a diverse range of socioeconomic and health system contexts and thus allowing for a nuanced examination of stillbirth, neonatal, and infant mortality rates worldwide. Second, the longitudinal analysis from 2000 to 2021 provides insights into mortality trends over two decades. This temporal breadth enables the identification of long-term patterns and shifts in mortality rates, offering valuable perspectives on the impact of global health interventions and policies over time. Additionally, we employed advanced spatial autocorrelation and cluster analysis techniques, providing a spatial perspective that is crucial for identifying geographical patterns in mortality rates. This approach helps in effectively targeting interventions and allocating resources where they are most needed, thereby enhancing the study's policy relevance.

However, like many in global health, our study has limitations related to data quality and completeness, the foremost being our reliance on two similar data sources, which, while robust, may contain systematic biases that could influence the validity of our results. We acknowledge that both databases used may be subject to similar systematic limitations and that differences in the reliability and comprehensiveness of health data systems across countries may introduce further biases. In particular, variations in the definitions and reporting practices for stillbirths, neonatal, and infant deaths across countries pose significant challenges to data comparability. These discrepancies – stemming from differences in health care practices, reporting standards, and sociocultural contexts – can impact the accuracy and interpretation of the results, potentially affecting the study’s overall conclusions. Another limitation arises from the modelling and averaging of country-level data, which may smooth out extreme values and reduce sensitivity to identifying mortality hotspots. Although averaging is valuable for capturing overall trends, it can obscure outliers and localised patterns, potentially missing high-mortality areas or unique national trends that would benefit from targeted interventions. Additionally, the ecological approach of aggregating data at the country level constrains our ability to infer individual-level factors influencing mortality rates, limiting causal interpretations. Our decision to exclude variables with more than 20% missing data was necessary for maintaining data reliability, but this exclusion may have further limited the ability to detect certain hotspots. Future studies could explore advanced imputation techniques or adjusted thresholds to improve hotspot sensitivity without compromising data quality. Finally, we acknowledge that the AAPC values, while potentially conservative, were chosen for their simplicity and interpretability. The AAPC offers a consistent metric for examining long-term mortality trends across diverse regions, balancing interpretability with the accurate depiction of extended trends across countries.

## CONCLUSIONS

Our findings offer valuable insights into global mortality trends and spatial patterns. By identifying hotspots and outliers, targeted health interventions can be implemented and resources allocated efficiently to areas with the greatest need, ensuring strategic and impactful efforts. Regional collaboration plays a pivotal role, allowing neighbouring countries confronting similar health challenges to exchange best practices and pool resources, thereby enhancing regional health outcomes. Moreover, the consistent associations across different mortality rates underscore the imperative for holistic public health approaches that address multiple determinants simultaneously. Implementing comprehensive, evidence-based strategies integrating education, health care, nutrition, and sanitation is crucial for reducing stillbirth, neonatal, and infant mortality rates, ultimately improving overall child health and well-being. These integrated approaches contribute to more effective public health strategies, leading to reduced mortality rates and healthier global populations.

## Additional material


Online Supplementary Document
