# Supplementary material for: Spatial patterns and temporal trends in stillbirth, neonatal, and infant mortality: an exploration of country-level data from 2000 to 2021
Source: J Glob Health. 2025 Feb 21;15:04034. doi: 10.7189/jogh.15.04034 (PMC11843375; doi:10.7189/jogh.15.04034)
Supplement: Online Supplementary Document [file jogh-15-04034-s001.pdf]

Supplement to: Maugeri A, Barchitta M, Schillaci G, Agodi A. Spatial patterns and temporal trends in stillbirth, neonatal, and infant mortality: an exploration of country-level data from 2000 to 2021. J Glob Health. 2025;15:04034.

Table S1. List of World Development Indicators included in the analysis.

Table S2. Results from the joinpoint regression analysis of stillbirth rates.

Table S3. Results from the joinpoint regression analysis of neonatal mortality rate.

Table S4. Results from the joinpoint regression analysis of infant mortality rate.

Figure S1. Pairwise comparisons of temporal trend in stillbirth rates across UNICEF reporting regions using joinpoint regression.

Figure S2. Pairwise comparisons of temporal trend in neonatal mortality rates across UNICEF reporting regions using joinpoint regression.

Figure S3. Pairwise comparisons of temporal trend in infant mortality rates across UNICEF reporting regions using joinpoint regression.

Table S1. List of World Development Indicators included in the analysis

Indicator	Definition
Adolescent fertility rate (births per 1,000 women ages 15-19)	The number of births per 1,000 women ages 15-19.
Age dependency ratio, old (% of working-age population)	the ratio of older dependents--people older than 15--to the working-age population--those ages 15-64.
Age dependency ratio, young (% of working-age population)	The ratio of younger dependents--people younger than 15--to the working-age population--those ages 15-64.
Fixed telephone subscriptions (per 100 people)	The sum of active number of analogue fixed telephone lines, voice-over-IP (VoIP) subscriptions, fixed wireless local loop (WLL) subscriptions, ISDN voice-channel equivalents and fixed public payphones.
GDP per capita (current US$)	Gross domestic product divided by midyear population. GDP is the sum of gross value added by all resident producers in the economy plus any product taxes and minus any subsidies not included in the value of the products. Data are in constant 2015 U.S. dollars.
Government Effectiveness: Estimate	Government Effectiveness captures perceptions of the quality of public services, the quality of the civil service and the degree of its independence from political pressures, the quality of policy formulation and implementation, and the credibility of the government's commitment to such policies. Estimate gives the country's score on the aggregate indicator, in units of a standard normal distribution, i.e. ranging from approximately -2.5 to 2.5.
Incidence of tuberculosis (per 100,000 people)	The estimated number of new and relapse tuberculosis cases arising in a given year, expressed as the rate per 100,000 population. All forms of TB are included, including cases in people living with HIV.
Individuals using the Internet (% of population)	The percentage of internet users, individuals who have used the Internet (from any location) in the last 3 months. The Internet can be used via a computer, mobile phone, personal digital assistant, games machine, digital TV etc.
People practicing open defecation (% of population)	The percentage of the population defecating in the open, such as in fields, forest, bushes, open bodies of water, on beaches, in other open spaces or disposed of with solid waste.
People using at least basic drinking water services (% of population)	The percentage of people using at least basic water services. This indicator encompasses both people using basic water services as well as those using safely managed water services. Basic drinking water services is defined as drinking water from an improved source, provided collection time is not more than 30 minutes for a round trip. Improved water sources include piped water, boreholes or tubewells, protected dug wells, protected springs, and packaged or delivered water.
People using at least basic sanitation services (% of population)	The percentage of people using at least basic sanitation services, that is, improved sanitation facilities that are not shared with other households. This indicator encompasses both people using basic sanitation services as well as those using safely managed sanitation services. Improved

	sanitation facilities include flush/pour flush to piped sewer systems, septic tanks or pit latrines; ventilated improved pit latrines, compositing toilets or pit latrines with slabs.
Prevalence of undernourishment (% of population)	The percentage of the population whose habitual food consumption is insufficient to provide the dietary energy levels that are required to maintain a normal active and healthy life.
Regulatory Quality: Estimate	Regulatory Quality captures perceptions of the ability of the government to formulate and implement sound policies and regulations that permit and promote private sector development. Estimate gives the country's score on the aggregate indicator, in units of a standard normal distribution, i.e. ranging from approximately -2.5 to 2.5.
Rule of Law: Estimate	Rule of Law captures perceptions of the extent to which agents have confidence in and abide by the rules of society, and in particular the quality of contract enforcement, property rights, the police, and the courts, as well as the likelihood of crime and violence. Estimate gives the country's score on the aggregate indicator, in units of a standard normal distribution, i.e. ranging from approximately -2.5 to 2.5.
Urban population growth (annual %)	People living in urban areas as defined by national statistical offices. It is calculated using World Bank population estimates and urban ratios from the United Nations World Urbanization Prospects
Women's share of population ages 15+ living with HIV (%)	Prevalence of HIV is the percentage of people who are infected with HIV. Female rate is as a percentage of the total population ages 15+ who are living with HIV.
Mean years of schooling	The average number of years of education (primary/ISCED 1 or higher) completed by a country's female/male/total population (25 years and older) excluding years spent repeating grades
Carbon dioxide emissions (production) index	CO2 emissions from electricity and heat production, total (% of total fuel combustion)
Density of medical doctors (per 10,000 population)	The number of physicians (including generalist and specialist medical practitioners) per 10,000 population
Density of nursing and midwifery personnel (per 10,000 population)	The number of nursing and midwifery personnel per 10,000 population

Table S2. Results from the joinpoint regression analysis of stillbirth rates

Country	Segment	APC	APC 95% LCL	APC 95% UCL	p-value
AFG: Afghanistan	2000-2006	-1.212	-1.262	-1.162	<0.001
AFG: Afghanistan	2006-2019	-1.577	-1.594	-1.56	<0.001
AFG: Afghanistan	2019-2021	-2.045	-2.336	-1.753	<0.001
AGO: Angola	2000-2003	-1.458	-1.721	-1.193	<0.001
AGO: Angola	2003-2012	-2.507	-2.564	-2.45	<0.001
AGO: Angola	2012-2015	-2.028	-2.552	-1.501	<0.001
AGO: Angola	2015-2018	-0.933	-1.463	-0.401	0.004
AGO: Angola	2018-2021	-0.52	-0.787	-0.254	0.002
ALB: Albania	2000-2006	-2.917	-3.135	-2.699	<0.001
ALB: Albania	2006-2010	-6.599	-7.217	-5.977	<0.001
ALB: Albania	2010-2015	-2.315	-2.724	-1.904	<0.001
ALB: Albania	2015-2021	1.484	1.257	1.712	<0.001
AND: Andorra	2000-2009	-2.793	-2.844	-2.742	<0.001
AND: Andorra	2009-2021	-1.912	-1.945	-1.878	<0.001
ARE: United Arab Emirates	2000-2004	-3.151	-3.537	-2.763	<0.001
ARE: United Arab Emirates	2004-2007	-0.344	-1.595	0.922	0.546
ARE: United Arab Emirates	2007-2013	-4.607	-4.876	-4.337	<0.001
ARE: United Arab Emirates	2013-2017	-2.806	-3.417	-2.19	<0.001
ARE: United Arab Emirates	2017-2021	-1.57	-1.962	-1.176	<0.001
ARG: Argentina	2000-2002	-3.254	-8.024	1.764	0.17
ARG: Argentina	2002-2005	-7.135	-11.714	-2.318	0.01
ARG: Argentina	2005-2010	-3.288	-4.822	-1.729	0.001
ARG: Argentina	2010-2018	0.77	0.091	1.453	0.031
ARG: Argentina	2018-2021	-6.821	-9.147	-4.435	<0.001
ARM: Armenia	2000-2008	-3.492	-3.639	-3.346	<0.001
ARM: Armenia	2008-2017	-1.63	-1.779	-1.481	<0.001
ARM: Armenia	2017-2021	-4.895	-5.312	-4.476	<0.001
ATG: Antigua and Barbuda	2000-2003	-1.318	-1.592	-1.043	<0.001
ATG: Antigua and Barbuda	2003-2006	-2.569	-3.11	-2.025	<0.001
ATG: Antigua and Barbuda	2006-2012	-3.631	-3.751	-3.511	<0.001
ATG: Antigua and Barbuda	2012-2015	-2.877	-3.416	-2.334	<0.001
ATG: Antigua and Barbuda	2015-2021	-2.007	-2.099	-1.915	<0.001
AUS: Australia	2000-2002	-5.026	-7.955	-2.004	0.005
AUS: Australia	2002-2007	-1.368	-2.34	-0.386	0.012
AUS: Australia	2007-2010	0.12	-2.968	3.306	0.932
AUS: Australia	2010-2015	-3.249	-4.203	-2.286	<0.001
AUS: Australia	2015-2021	-1.453	-1.973	-0.93	<0.001
AUT: Austria	2000-2002	-0.5	-2.006	1.029	0.47
AUT: Austria	2002-2008	-2.067	-2.401	-1.733	<0.001
AUT: Austria	2008-2012	0.212	-0.549	0.979	0.54
AUT: Austria	2012-2018	-1.532	-1.868	-1.196	<0.001
AUT: Austria	2018-2021	2.744	1.964	3.531	<0.001
AZE: Azerbaijan	2000-2004	-2.06	-2.31	-1.809	<0.001
AZE: Azerbaijan	2004-2009	-3.447	-3.694	-3.199	<0.001
AZE: Azerbaijan	2009-2016	-5.159	-5.289	-5.029	<0.001

AZE: Azerbaijan	2016-2019	-3.389	-4.168	-2.604	<0.001
AZE: Azerbaijan	2019-2021	-2.324	-3.111	-1.53	<0.001
BDI: Burundi	2000-2018	-0.991	-1.138	-0.844	<0.001
BDI: Burundi	2018-2021	-9.943	-11.999	-7.84	<0.001
BEL: Belgium	2000-2003	0.029	-0.907	0.973	0.946
BEL: Belgium	2003-2009	-1.537	-1.95	-1.122	<0.001
BEL: Belgium	2009-2012	0.628	-1.245	2.537	0.464
BEL: Belgium	2012-2016	-1.313	-2.236	-0.381	0.012
BEL: Belgium	2016-2021	-0.267	-0.685	0.153	0.181
BEN: Benin	2000-2004	-1.369	-1.529	-1.21	<0.001
BEN: Benin	2004-2011	-0.853	-0.938	-0.767	<0.001
BEN: Benin	2011-2014	-4.655	-5.141	-4.167	<0.001
BEN: Benin	2014-2021	-0.987	-1.054	-0.919	<0.001
BFA: Burkina Faso	2000-2003	-1.548	-2.298	-0.793	0.002
BFA: Burkina Faso	2003-2011	-2.442	-2.641	-2.243	<0.001
BFA: Burkina Faso	2011-2016	-3.595	-4.06	-3.128	<0.001
BFA: Burkina Faso	2016-2019	2.288	0.736	3.863	0.009
BFA: Burkina Faso	2019-2021	0.059	-1.459	1.6	0.932
BGD: Bangladesh	2000-2007	-3.174	-3.327	-3.02	<0.001
BGD: Bangladesh	2007-2011	-4.059	-4.626	-3.489	<0.001
BGD: Bangladesh	2011-2014	-0.226	-1.401	0.963	0.672
BGD: Bangladesh	2014-2017	-2.89	-4.034	-1.733	<0.001
BGD: Bangladesh	2017-2021	-5.497	-5.851	-5.142	<0.001
BGR: Bulgaria	2000-2003	1.991	-0.57	4.616	0.119
BGR: Bulgaria	2003-2019	-2.562	-2.771	-2.353	<0.001
BGR: Bulgaria	2019-2021	1.355	-3.67	6.641	0.579
BHR: Bahrain	2000-2009	-3.832	-3.881	-3.783	<0.001
BHR: Bahrain	2009-2012	1.33	0.765	1.898	0.001
BHR: Bahrain	2012-2015	-1.09	-1.641	-0.536	0.002
BHR: Bahrain	2015-2018	0.904	0.342	1.47	0.006
BHR: Bahrain	2018-2021	-0.897	-1.174	-0.62	<0.001
BHS: Bahamas	2000-2004	5.996	5.001	7	<0.001
BHS: Bahamas	2004-2015	-2.052	-2.279	-1.824	<0.001
BHS: Bahamas	2015-2021	-0.917	-1.415	-0.416	0.002
BIH: Bosnia and Herzegovina	2000-2003	-3.248	-3.559	-2.935	<0.001
BIH: Bosnia and Herzegovina	2003-2008	-0.878	-1.08	-0.675	<0.001
BIH: Bosnia and Herzegovina	2008-2012	-3.995	-4.304	-3.685	<0.001
BIH: Bosnia and Herzegovina	2012-2021	-1.197	-1.255	-1.139	<0.001
BLR: Belarus	2000-2004	-5.281	-5.934	-4.623	<0.001
BLR: Belarus	2004-2010	-8.608	-9.054	-8.159	<0.001
BLR: Belarus	2010-2013	-3.325	-5.417	-1.186	0.007
BLR: Belarus	2013-2019	0.818	0.326	1.313	0.005
BLR: Belarus	2019-2021	-2.766	-4.87	-0.615	0.018
BLZ: Belize	2000-2007	-2.575	-2.759	-2.391	<0.001
BLZ: Belize	2007-2013	-0.078	-0.393	0.238	0.597
BLZ: Belize	2013-2018	-4.046	-4.473	-3.616	<0.001
BLZ: Belize	2018-2021	-0.468	-1.168	0.238	0.172

BOL: Bolivia	2000-2006	-1.647	-1.719	-1.575	<0.001
BOL: Bolivia	2006-2009	-3.285	-3.702	-2.865	<0.001
BOL: Bolivia	2009-2014	-3.894	-4.025	-3.762	<0.001
BOL: Bolivia	2014-2017	-3.049	-3.467	-2.629	<0.001
BOL: Bolivia	2017-2021	-2.092	-2.226	-1.958	<0.001
BRA: Brazil	2000-2006	-2.148	-2.339	-1.957	<0.001
BRA: Brazil	2006-2015	-1.03	-1.155	-0.905	<0.001
BRA: Brazil	2015-2018	-4.351	-5.449	-3.24	<0.001
BRA: Brazil	2018-2021	-0.412	-0.985	0.164	0.144
BRB: Barbados	2000-2006	0.619	0.492	0.746	<0.001
BRB: Barbados	2006-2011	-0.24	-0.476	-0.004	0.047
BRB: Barbados	2011-2015	-0.77	-1.141	-0.398	0.001
BRB: Barbados	2015-2021	-1.437	-1.562	-1.313	<0.001
BRN: Brunei Darussalam	2000-2011	-1.901	-2.021	-1.781	<0.001
BRN: Brunei Darussalam	2011-2015	0.844	-0.066	1.762	0.066
BRN: Brunei Darussalam	2015-2019	2.82	1.892	3.756	<0.001
BRN: Brunei Darussalam	2019-2021	0.405	-1.399	2.242	0.634
BTN: Bhutan	2000-2006	-2.962	-3.042	-2.882	<0.001
BTN: Bhutan	2006-2015	-3.727	-3.779	-3.676	<0.001
BTN: Bhutan	2015-2018	-2.243	-2.719	-1.763	<0.001
BTN: Bhutan	2018-2021	-1.692	-1.932	-1.451	<0.001
BWA: Botswana	2000-2005	9.313	8.793	9.835	<0.001
BWA: Botswana	2005-2008	2.851	0.682	5.067	0.016
BWA: Botswana	2008-2011	-0.907	-2.996	1.228	0.353
BWA: Botswana	2011-2014	-2.752	-4.803	-0.657	0.017
BWA: Botswana	2014-2021	-1.756	-2.036	-1.476	<0.001
CAF: Central African Republic	2000-2005	-0.964	-1.051	-0.877	<0.001
CAF: Central African Republic	2005-2012	-1.196	-1.262	-1.13	<0.001
CAF: Central African Republic	2012-2017	0.075	-0.049	0.199	0.211
CAF: Central African Republic	2017-2021	-0.824	-0.947	-0.701	<0.001
CAN: Canada	2000-2004	-2.411	-3.146	-1.669	<0.001
CAN: Canada	2004-2007	1.538	-0.863	3.998	0.188
CAN: Canada	2007-2011	-1.69	-2.86	-0.507	0.009
CAN: Canada	2011-2021	-0.393	-0.579	-0.208	0.001
CHE: Switzerland	2000-2003	-0.186	-0.511	0.141	0.226
CHE: Switzerland	2003-2011	-2.595	-2.68	-2.51	<0.001
CHE: Switzerland	2011-2014	0.968	0.311	1.63	0.009
CHE: Switzerland	2014-2017	-0.378	-1.027	0.275	0.218
CHE: Switzerland	2017-2021	1.681	1.471	1.892	<0.001
CHL: Chile	2000-2009	-1.214	-1.485	-0.942	<0.001
CHL: Chile	2009-2021	-2.856	-3.029	-2.683	<0.001
CHN: China	2000-2003	0.1	-1.144	1.359	0.863
CHN: China	2003-2013	-5.313	-5.528	-5.096	<0.001
CHN: China	2013-2017	-8.543	-9.679	-7.392	<0.001
CHN: China	2017-2021	-5.38	-6.126	-4.629	<0.001
CIV: CÃ´te d'Ivoire	2000-2010	-1.137	-1.165	-1.108	<0.001
CIV: CÃ´te d'Ivoire	2010-2013	-0.909	-1.28	-0.535	<0.001

CIV: CÃ´te d'Ivoire	2013-2016	-2.572	-2.938	-2.206	<0.001
CIV: CÃ´te d'Ivoire	2016-2021	-1.631	-1.713	-1.548	<0.001
CMR: Cameroon	2000-2004	-1.157	-1.3	-1.013	<0.001
CMR: Cameroon	2004-2014	-0.774	-0.816	-0.733	<0.001
CMR: Cameroon	2014-2021	-1.405	-1.465	-1.344	<0.001
COD: Congo DRC	2000-2003	0.053	-0.166	0.271	0.607
COD: Congo DRC	2003-2011	-1.057	-1.115	-0.999	<0.001
COD: Congo DRC	2011-2019	-0.387	-0.445	-0.329	<0.001
COD: Congo DRC	2019-2021	-1.12	-1.551	-0.687	<0.001
COG: Congo	2000-2004	-1.527	-1.718	-1.335	<0.001
COG: Congo	2004-2009	-2.514	-2.703	-2.324	<0.001
COG: Congo	2009-2015	-1.659	-1.795	-1.524	<0.001
COG: Congo	2015-2021	-0.239	-0.343	-0.135	<0.001
COK: Cook Islands	2000-2006	-2.846	-2.928	-2.763	<0.001
COK: Cook Islands	2006-2012	-4.591	-4.698	-4.484	<0.001
COK: Cook Islands	2012-2016	-3.379	-3.621	-3.136	<0.001
COK: Cook Islands	2016-2021	-2.536	-2.646	-2.427	<0.001
COL: Colombia	2000-2002	-1.519	-1.994	-1.042	<0.001
COL: Colombia	2002-2006	-0.216	-0.457	0.025	0.073
COL: Colombia	2006-2011	-0.867	-1.018	-0.715	<0.001
COL: Colombia	2011-2017	-1.869	-1.975	-1.763	<0.001
COL: Colombia	2017-2021	-1.564	-1.714	-1.413	<0.001
COM: Comoros	2000-2005	-0.377	-0.472	-0.282	<0.001
COM: Comoros	2005-2010	-0.912	-1.046	-0.779	<0.001
COM: Comoros	2010-2015	-1.383	-1.516	-1.25	<0.001
COM: Comoros	2015-2021	-1.105	-1.177	-1.034	<0.001
CPV: Cabo Verde	2000-2003	-2.553	-2.738	-2.367	<0.001
CPV: Cabo Verde	2003-2009	-0.323	-0.408	-0.239	<0.001
CPV: Cabo Verde	2009-2012	-2.273	-2.643	-1.9	<0.001
CPV: Cabo Verde	2012-2018	-3.711	-3.793	-3.629	<0.001
CPV: Cabo Verde	2018-2021	-2.592	-2.777	-2.407	<0.001
CRI: Costa Rica	2000-2011	0.187	-0.033	0.407	0.091
CRI: Costa Rica	2011-2021	-1.78	-2.029	-1.53	<0.001
CUB: Cuba	2000-2006	-3.705	-4.269	-3.138	<0.001
CUB: Cuba	2006-2011	-5.245	-6.281	-4.199	<0.001
CUB: Cuba	2011-2014	-2.466	-5.797	0.982	0.136
CUB: Cuba	2014-2018	3.874	2.085	5.694	0.001
CUB: Cuba	2018-2021	-0.956	-2.661	0.78	0.238
CYP: Cyprus	2000-2008	-4.362	-4.501	-4.223	<0.001
CYP: Cyprus	2008-2011	-3.419	-4.699	-2.122	<0.001
CYP: Cyprus	2011-2014	-0.068	-1.392	1.274	0.913
CYP: Cyprus	2014-2021	-0.923	-1.1	-0.747	<0.001
CZE: Czech Republic	2000-2005	-0.743	-1.459	-0.021	0.044
CZE: Czech Republic	2005-2008	-2.496	-5.602	0.713	0.116
CZE: Czech Republic	2008-2021	0.67	0.499	0.841	<0.001
DEU: Germany	2000-2004	-1.969	-2.312	-1.625	<0.001
DEU: Germany	2004-2009	-3.575	-3.912	-3.237	<0.001

DEU: Germany	2009-2013	0.266	-0.288	0.822	0.301
DEU: Germany	2013-2017	5.045	4.465	5.628	<0.001
DEU: Germany	2017-2021	-0.344	-0.693	0.005	0.053
DJI: Djibouti	2000-2002	-0.879	-1.133	-0.625	<0.001
DJI: Djibouti	2002-2009	-0.405	-0.448	-0.362	<0.001
DJI: Djibouti	2009-2012	-1.479	-1.731	-1.226	<0.001
DJI: Djibouti	2012-2018	-2.278	-2.334	-2.222	<0.001
DJI: Djibouti	2018-2021	-1.975	-2.1	-1.849	<0.001
DMA: Dominica	2000-2004	1.373	1.168	1.579	<0.001
DMA: Dominica	2004-2017	1.777	1.739	1.816	<0.001
DMA: Dominica	2017-2021	0.908	0.704	1.112	<0.001
DNK: Denmark	2000-2002	-1.916	-2.765	-1.059	0.001
DNK: Denmark	2002-2009	-4.651	-4.791	-4.51	<0.001
DNK: Denmark	2009-2012	-2.041	-2.889	-1.186	0.001
DNK: Denmark	2012-2017	-0.162	-0.436	0.113	0.212
DNK: Denmark	2017-2021	-1.609	-1.879	-1.338	<0.001
DOM: Dominican Republic	2000-2002	-0.846	-1.16	-0.531	<0.001
DOM: Dominican Republic	2002-2007	-0.29	-0.39	-0.19	<0.001
DOM: Dominican Republic	2007-2017	-0.864	-0.892	-0.835	<0.001
DOM: Dominican Republic	2017-2021	-1.364	-1.463	-1.265	<0.001
DZA: Algeria	2000-2003	-0.657	-0.857	-0.456	<0.001
DZA: Algeria	2003-2006	-4.349	-4.735	-3.962	<0.001
DZA: Algeria	2006-2016	-4.948	-4.983	-4.913	<0.001
DZA: Algeria	2016-2019	-1.923	-2.318	-1.526	<0.001
DZA: Algeria	2019-2021	-0.524	-0.925	-0.121	0.017
ECU: Ecuador	2000-2002	-2.941	-3.297	-2.584	<0.001
ECU: Ecuador	2002-2012	-3.451	-3.484	-3.419	<0.001
ECU: Ecuador	2012-2015	-3.105	-3.461	-2.749	<0.001
ECU: Ecuador	2015-2018	-1.579	-1.94	-1.217	<0.001
ECU: Ecuador	2018-2021	-0.451	-0.634	-0.268	<0.001
EGY: Egypt	2000-2005	-2.587	-2.696	-2.478	<0.001
EGY: Egypt	2005-2013	-3.433	-3.498	-3.369	<0.001
EGY: Egypt	2013-2016	-3.19	-3.675	-2.703	<0.001
EGY: Egypt	2016-2021	-2.247	-2.357	-2.138	<0.001
ERI: Eritrea	2000-2005	-1.205	-1.303	-1.107	<0.001
ERI: Eritrea	2005-2013	-1.471	-1.53	-1.412	<0.001
ERI: Eritrea	2013-2021	-1.295	-1.343	-1.247	<0.001
ESP: Spain	2000-2004	-3.429	-4.138	-2.715	<0.001
ESP: Spain	2004-2008	-1.481	-2.622	-0.327	0.018
ESP: Spain	2008-2012	0.572	-0.593	1.75	0.292
ESP: Spain	2012-2015	-5.68	-7.852	-3.457	<0.001
ESP: Spain	2015-2021	-0.991	-1.38	-0.6	<0.001
EST: Estonia	2000-2002	-3.279	-5.244	-1.274	0.006
EST: Estonia	2002-2012	-4.299	-4.478	-4.12	<0.001
EST: Estonia	2012-2016	-2.335	-3.331	-1.327	0.001
EST: Estonia	2016-2019	-5.915	-7.826	-3.965	<0.001
EST: Estonia	2019-2021	-4.223	-6.169	-2.238	0.001

ETH: Ethiopia	2000-2006	-1.283	-1.316	-1.25	<0.001
ETH: Ethiopia	2006-2012	-1.928	-1.972	-1.885	<0.001
ETH: Ethiopia	2012-2016	-2.923	-3.019	-2.826	<0.001
ETH: Ethiopia	2016-2019	-3.2	-3.392	-3.007	<0.001
ETH: Ethiopia	2019-2021	-2.489	-2.683	-2.295	<0.001
FIN: Finland	2000-2004	-3.937	-4.168	-3.706	<0.001
FIN: Finland	2004-2007	-0.118	-0.874	0.643	0.729
FIN: Finland	2007-2012	-3.005	-3.238	-2.772	<0.001
FIN: Finland	2012-2016	1.138	0.755	1.523	<0.001
FIN: Finland	2016-2021	-0.944	-1.112	-0.776	<0.001
FJI: Fiji	2000-2005	1.625	0.672	2.587	0.003
FJI: Fiji	2005-2012	-2.003	-2.699	-1.303	<0.001
FJI: Fiji	2012-2015	-4.342	-8.289	-0.226	0.041
FJI: Fiji	2015-2021	1.59	0.869	2.316	<0.001
FRA: France	2000-2002	-2.591	-4.637	-0.501	0.02
FRA: France	2002-2006	-5.657	-6.653	-4.65	<0.001
FRA: France	2006-2012	-4.125	-4.579	-3.669	<0.001
FRA: France	2012-2021	0.623	0.428	0.819	<0.001
FSM: Micronesia	2000-2003	-1.106	-1.243	-0.968	<0.001
FSM: Micronesia	2003-2008	-0.426	-0.513	-0.338	<0.001
FSM: Micronesia	2008-2012	-1.151	-1.288	-1.013	<0.001
FSM: Micronesia	2012-2018	-1.717	-1.778	-1.656	<0.001
FSM: Micronesia	2018-2021	-1.906	-2.043	-1.77	<0.001
GAB: Gabon	2000-2004	-0.48	-0.612	-0.347	<0.001
GAB: Gabon	2004-2008	-0.732	-0.941	-0.522	<0.001
GAB: Gabon	2008-2011	-1.025	-1.442	-0.606	<0.001
GAB: Gabon	2011-2016	-1.642	-1.773	-1.51	<0.001
GAB: Gabon	2016-2021	-1.209	-1.302	-1.115	<0.001
GBR: United Kingdom	2000-2005	-0.446	-0.66	-0.232	0.001
GBR: United Kingdom	2005-2011	-2.49	-2.699	-2.28	<0.001
GBR: United Kingdom	2011-2015	-4.963	-5.418	-4.505	<0.001
GBR: United Kingdom	2015-2019	-3.304	-3.767	-2.838	<0.001
GBR: United Kingdom	2019-2021	0.853	-0.111	1.827	0.076
GEO: Georgia	2000-2003	-3.814	-4.796	-2.821	<0.001
GEO: Georgia	2003-2008	-7.072	-7.673	-6.467	<0.001
GEO: Georgia	2008-2012	-4.879	-5.851	-3.898	<0.001
GEO: Georgia	2012-2017	-6.398	-7.004	-5.789	<0.001
GEO: Georgia	2017-2021	-0.608	-1.251	0.039	0.062
GHA: Ghana	2000-2002	-1.39	-1.699	-1.08	<0.001
GHA: Ghana	2002-2008	-0.801	-0.871	-0.732	<0.001
GHA: Ghana	2008-2011	-1.582	-1.89	-1.272	<0.001
GHA: Ghana	2011-2014	-0.777	-1.089	-0.465	<0.001
GHA: Ghana	2014-2021	-1.858	-1.899	-1.817	<0.001
GIN: Guinea	2000-2002	-2.237	-2.791	-1.68	<0.001
GIN: Guinea	2002-2006	-1.451	-1.731	-1.171	<0.001
GIN: Guinea	2006-2015	-1.12	-1.181	-1.058	<0.001
GIN: Guinea	2015-2021	-0.584	-0.68	-0.489	<0.001

GMB: Gambia	2000-2008	-1.093	-1.139	-1.048	<0.001
GMB: Gambia	2008-2015	-1.259	-1.329	-1.188	<0.001
GMB: Gambia	2015-2021	-1.42	-1.49	-1.35	<0.001
GNB: Guinea-Bissau	2000-2005	-1.412	-1.512	-1.311	<0.001
GNB: Guinea-Bissau	2005-2009	-1.787	-2.011	-1.562	<0.001
GNB: Guinea-Bissau	2009-2015	-2.27	-2.37	-2.17	<0.001
GNB: Guinea-Bissau	2015-2021	-1.805	-1.881	-1.73	<0.001
GNQ: Equatorial Guinea	2000-2002	-0.821	-1.124	-0.518	<0.001
GNQ: Equatorial Guinea	2002-2005	-1.242	-1.543	-0.94	<0.001
GNQ: Equatorial Guinea	2005-2009	-2.467	-2.616	-2.318	<0.001
GNQ: Equatorial Guinea	2009-2017	-0.747	-0.788	-0.707	<0.001
GNQ: Equatorial Guinea	2017-2021	-0.152	-0.249	-0.056	0.007
GRC: Greece	2000-2007	-6.326	-7.181	-5.463	<0.001
GRC: Greece	2007-2011	2.676	-0.789	6.261	0.114
GRC: Greece	2011-2015	-1.21	-4.544	2.24	0.437
GRC: Greece	2015-2019	1.593	-1.835	5.141	0.319
GRC: Greece	2019-2021	-3.971	-10.342	2.853	0.211
GRD: Grenada	2000-2007	-0.397	-0.476	-0.319	<0.001
GRD: Grenada	2007-2014	1.424	1.323	1.525	<0.001
GRD: Grenada	2014-2017	0.428	-0.162	1.021	0.139
GRD: Grenada	2017-2021	-0.782	-0.967	-0.597	<0.001
GTM: Guatemala	2000-2009	-2.07	-2.124	-2.016	<0.001
GTM: Guatemala	2009-2012	-3.112	-3.696	-2.525	<0.001
GTM: Guatemala	2012-2017	-0.953	-1.142	-0.763	<0.001
GTM: Guatemala	2017-2021	-1.541	-1.729	-1.352	<0.001
GUY: Guyana	2000-2007	-1.166	-1.215	-1.116	<0.001
GUY: Guyana	2007-2011	-1.305	-1.489	-1.12	<0.001
GUY: Guyana	2011-2015	-1.995	-2.178	-1.812	<0.001
GUY: Guyana	2015-2019	-1.831	-2.014	-1.647	<0.001
GUY: Guyana	2019-2021	-3.03	-3.392	-2.667	<0.001
HND: Honduras	2000-2003	-1.974	-2.187	-1.76	<0.001
HND: Honduras	2003-2007	-2.828	-3.039	-2.616	<0.001
HND: Honduras	2007-2010	-3.234	-3.655	-2.812	<0.001
HND: Honduras	2010-2015	-3.607	-3.74	-3.474	<0.001
HND: Honduras	2015-2021	-1.793	-1.866	-1.721	<0.001
HRV: Croatia	2000-2005	-3.891	-4.511	-3.268	<0.001
HRV: Croatia	2005-2008	-5.656	-8.346	-2.887	0.001
HRV: Croatia	2008-2012	-3.478	-4.864	-2.072	<0.001
HRV: Croatia	2012-2021	-0.21	-0.473	0.054	0.108
HTI: Haiti	2000-2008	-0.453	-0.553	-0.353	<0.001
HTI: Haiti	2008-2021	-0.995	-1.043	-0.947	<0.001
HUN: Hungary	2000-2012	-1.761	-1.929	-1.592	<0.001
HUN: Hungary	2012-2015	1.515	-1.388	4.504	0.285
HUN: Hungary	2015-2021	-0.154	-0.642	0.337	0.511
IDN: Indonesia	2000-2002	-1.743	-2.232	-1.252	<0.001
IDN: Indonesia	2002-2005	-2.79	-3.273	-2.304	<0.001
IDN: Indonesia	2005-2009	-2.186	-2.429	-1.942	<0.001

IDN: Indonesia	2009-2015	-2.992	-3.1	-2.884	<0.001
IDN: Indonesia	2015-2021	-2.206	-2.288	-2.123	<0.001
IND: India	2000-2004	-2.475	-2.609	-2.341	<0.001
IND: India	2004-2011	-4.175	-4.245	-4.105	<0.001
IND: India	2011-2014	-2.603	-3.026	-2.179	<0.001
IND: India	2014-2018	-6.955	-7.157	-6.753	<0.001
IND: India	2018-2021	-4.25	-4.458	-4.042	<0.001
IRL: Ireland	2000-2008	-2.856	-3.231	-2.48	<0.001
IRL: Ireland	2008-2012	-4.12	-5.804	-2.405	0.001
IRL: Ireland	2012-2015	-1.681	-5.105	1.866	0.302
IRL: Ireland	2015-2018	-6.007	-9.28	-2.615	0.004
IRL: Ireland	2018-2021	-1.685	-3.412	0.073	0.058
IRN: Iran	2000-2005	-2.365	-2.481	-2.25	<0.001
IRN: Iran	2005-2012	-3.153	-3.239	-3.066	<0.001
IRN: Iran	2012-2021	-2.264	-2.311	-2.217	<0.001
IRQ: Iraq	2000-2004	-0.941	-1.144	-0.737	<0.001
IRQ: Iraq	2004-2007	-1.442	-2.08	-0.8	0.001
IRQ: Iraq	2007-2012	-1.943	-2.145	-1.742	<0.001
IRQ: Iraq	2012-2019	-2.506	-2.613	-2.399	<0.001
IRQ: Iraq	2019-2021	-1.921	-2.555	-1.282	<0.001
ISL: Iceland	2000-2011	-2.791	-2.86	-2.722	<0.001
ISL: Iceland	2011-2016	-0.409	-0.74	-0.077	0.019
ISL: Iceland	2016-2021	-1.5	-1.731	-1.268	<0.001
ISR: Israel	2000-2004	-8.512	-9.411	-7.604	<0.001
ISR: Israel	2004-2007	1.178	-1.934	4.388	0.412
ISR: Israel	2007-2010	-4.301	-7.244	-1.264	0.012
ISR: Israel	2010-2013	0.893	-2.21	4.095	0.53
ISR: Israel	2013-2021	-0.336	-0.675	0.004	0.052
ITA: Italy	2000-2007	-2.592	-2.75	-2.434	<0.001
ITA: Italy	2007-2011	0.296	-0.309	0.905	0.293
ITA: Italy	2011-2015	3.379	2.755	4.007	<0.001
ITA: Italy	2015-2018	-3.732	-4.89	-2.559	<0.001
ITA: Italy	2018-2021	-2.286	-2.875	-1.692	<0.001
JAM: Jamaica	2000-2002	-0.518	-1.073	0.04	0.066
JAM: Jamaica	2002-2010	-1.325	-1.399	-1.251	<0.001
JAM: Jamaica	2010-2017	-0.672	-0.766	-0.578	<0.001
JAM: Jamaica	2017-2021	-1.927	-2.1	-1.753	<0.001
JOR: Jordan	2000-2002	-1.431	-1.741	-1.121	<0.001
JOR: Jordan	2002-2008	-2.051	-2.12	-1.982	<0.001
JOR: Jordan	2008-2012	-1.368	-1.523	-1.213	<0.001
JOR: Jordan	2012-2018	-0.999	-1.068	-0.929	<0.001
JOR: Jordan	2018-2021	-1.25	-1.405	-1.095	<0.001
JPN: Japan	2000-2007	-2.919	-3.456	-2.38	<0.001
JPN: Japan	2007-2010	-0.711	-4.747	3.496	0.712
JPN: Japan	2010-2019	-2.935	-3.373	-2.494	<0.001
JPN: Japan	2019-2021	0.145	-3.926	4.389	0.94
KAZ: Kazakhstan	2000-2007	-2.976	-3.745	-2.201	<0.001

KAZ: Kazakhstan	2007-2010	-6.034	-11.467	-0.268	0.042
KAZ: Kazakhstan	2010-2018	-3.37	-4.136	-2.598	<0.001
KAZ: Kazakhstan	2018-2021	13.32	9.995	16.745	<0.001
KEN: Kenya	2000-2004	-0.571	-0.948	-0.192	0.007
KEN: Kenya	2004-2010	-1.109	-1.374	-0.843	<0.001
KEN: Kenya	2010-2019	1.027	0.894	1.159	<0.001
KEN: Kenya	2019-2021	-2.762	-3.924	-1.586	<0.001
KGZ: Kyrgyzstan	2000-2007	-1.446	-1.545	-1.347	<0.001
KGZ: Kyrgyzstan	2007-2017	-3.286	-3.352	-3.22	<0.001
KGZ: Kyrgyzstan	2017-2021	-2.553	-2.784	-2.321	<0.001
KHM: Cambodia	2000-2011	-3.928	-3.963	-3.893	<0.001
KHM: Cambodia	2011-2015	-4.155	-4.413	-3.896	<0.001
KHM: Cambodia	2015-2018	-3.144	-3.666	-2.62	<0.001
KHM: Cambodia	2018-2021	-2.289	-2.552	-2.025	<0.001
KIR: Kiribati	2000-2005	-1.059	-1.148	-0.97	<0.001
KIR: Kiribati	2005-2012	-0.475	-0.542	-0.407	<0.001
KIR: Kiribati	2012-2016	-1.675	-1.872	-1.477	<0.001
KIR: Kiribati	2016-2021	-1.404	-1.492	-1.315	<0.001
KNA: Saint Kitts and Nevis	2000-2006	-2.642	-2.718	-2.567	<0.001
KNA: Saint Kitts and Nevis	2006-2009	-1.751	-2.199	-1.3	<0.001
KNA: Saint Kitts and Nevis	2009-2015	-0.744	-0.845	-0.642	<0.001
KNA: Saint Kitts and Nevis	2015-2021	-1.649	-1.725	-1.573	<0.001
KOR: South Korea	2000-2004	-3.259	-4.005	-2.507	<0.001
KOR: South Korea	2004-2011	-4.298	-4.694	-3.901	<0.001
KOR: South Korea	2011-2014	0.284	-2.143	2.77	0.797
KOR: South Korea	2014-2017	-5.642	-7.925	-3.303	0.001
KOR: South Korea	2017-2021	0.488	-0.287	1.269	0.185
KWT: Kuwait	2000-2003	-1.808	-2.109	-1.505	<0.001
KWT: Kuwait	2003-2009	-1.281	-1.417	-1.145	<0.001
KWT: Kuwait	2009-2014	-1.619	-1.81	-1.427	<0.001
KWT: Kuwait	2014-2021	0.166	0.084	0.248	0.001
LAO: Laos	2000-2007	-1.541	-1.579	-1.503	<0.001
LAO: Laos	2007-2012	-1.642	-1.732	-1.551	<0.001
LAO: Laos	2012-2019	-2.055	-2.103	-2.007	<0.001
LAO: Laos	2019-2021	-1.494	-1.78	-1.207	<0.001
LBN: Lebanon	2000-2003	-3.238	-3.469	-3.008	<0.001
LBN: Lebanon	2003-2010	-4.204	-4.281	-4.127	<0.001
LBN: Lebanon	2010-2014	-2.514	-2.746	-2.281	<0.001
LBN: Lebanon	2014-2019	-1.682	-1.83	-1.533	<0.001
LBN: Lebanon	2019-2021	-0.598	-1.071	-0.124	0.02
LBR: Liberia	2000-2006	-2.289	-2.346	-2.231	<0.001
LBR: Liberia	2006-2010	-1.606	-1.776	-1.435	<0.001
LBR: Liberia	2010-2016	-0.451	-0.527	-0.374	<0.001
LBR: Liberia	2016-2021	-0.913	-0.99	-0.837	<0.001
LBY: Libya	2000-2004	-1.96	-2.15	-1.771	<0.001
LBY: Libya	2004-2009	-3.741	-3.927	-3.555	<0.001
LBY: Libya	2009-2012	-2.906	-3.498	-2.311	<0.001

LBY: Libya	2012-2021	-1.768	-1.823	-1.713	<0.001
LCA: Saint Lucia	2000-2006	-0.55	-0.756	-0.343	<0.001
LCA: Saint Lucia	2006-2013	-1.363	-1.568	-1.158	<0.001
LCA: Saint Lucia	2013-2017	-1.836	-2.437	-1.231	<0.001
LCA: Saint Lucia	2017-2021	0.232	-0.156	0.623	0.215
LKA: Sri Lanka	2000-2005	-3.633	-3.911	-3.354	<0.001
LKA: Sri Lanka	2005-2009	-2.716	-3.342	-2.085	<0.001
LKA: Sri Lanka	2009-2013	-4.303	-4.919	-3.682	<0.001
LKA: Sri Lanka	2013-2018	-2.294	-2.693	-1.894	<0.001
LKA: Sri Lanka	2018-2021	0.684	0.035	1.337	0.041
LSO: Lesotho	2000-2002	-1.348	-1.878	-0.814	<0.001
LSO: Lesotho	2002-2005	-2.375	-2.9	-1.847	<0.001
LSO: Lesotho	2005-2012	-1.752	-1.842	-1.663	<0.001
LSO: Lesotho	2012-2021	-1.09	-1.139	-1.041	<0.001
LTU: Lithuania	2000-2005	-1.689	-2.024	-1.352	<0.001
LTU: Lithuania	2005-2009	-5.343	-6.064	-4.617	<0.001
LTU: Lithuania	2009-2012	-3.637	-5.098	-2.152	0.001
LTU: Lithuania	2012-2018	-1.563	-1.899	-1.226	<0.001
LTU: Lithuania	2018-2021	-3.973	-4.704	-3.236	<0.001
LUX: Luxembourg	2000-2004	-3.616	-3.896	-3.334	<0.001
LUX: Luxembourg	2004-2007	0.559	-0.364	1.49	0.201
LUX: Luxembourg	2007-2011	3.202	2.728	3.679	<0.001
LUX: Luxembourg	2011-2019	0.486	0.362	0.609	<0.001
LUX: Luxembourg	2019-2021	-0.409	-1.323	0.512	0.335
LVA: Latvia	2000-2002	-3.101	-3.956	-2.239	<0.001
LVA: Latvia	2002-2006	-5.866	-6.282	-5.448	<0.001
LVA: Latvia	2006-2011	-1.079	-1.356	-0.801	<0.001
LVA: Latvia	2011-2015	-3.21	-3.638	-2.78	<0.001
LVA: Latvia	2015-2021	-1.188	-1.336	-1.04	<0.001
MAR: Morocco	2000-2004	-1.534	-1.67	-1.397	<0.001
MAR: Morocco	2004-2010	-2.519	-2.615	-2.423	<0.001
MAR: Morocco	2010-2015	-1.684	-1.82	-1.547	<0.001
MAR: Morocco	2015-2019	-2.493	-2.707	-2.278	<0.001
MAR: Morocco	2019-2021	-2.027	-2.457	-1.595	<0.001
MCO: Monaco	2000-2005	-2.224	-2.366	-2.082	<0.001
MCO: Monaco	2005-2009	-2.862	-3.177	-2.545	<0.001
MCO: Monaco	2009-2013	-2.429	-2.746	-2.112	<0.001
MCO: Monaco	2013-2019	-3.538	-3.678	-3.397	<0.001
MCO: Monaco	2019-2021	-2.496	-3.128	-1.86	<0.001
MDA: Moldova	2000-2004	-5.395	-5.627	-5.163	<0.001
MDA: Moldova	2004-2007	-3.265	-4.013	-2.512	<0.001
MDA: Moldova	2007-2011	-1.371	-1.753	-0.988	<0.001
MDA: Moldova	2011-2014	-1.94	-2.698	-1.176	<0.001
MDA: Moldova	2014-2021	-1.598	-1.7	-1.496	<0.001
MDG: Madagascar	2000-2006	-1.696	-1.747	-1.645	<0.001
MDG: Madagascar	2006-2009	-0.125	-0.433	0.185	0.394
MDG: Madagascar	2009-2017	0.297	0.256	0.339	<0.001

MDG: Madagascar	2017-2021	-0.655	-0.752	-0.558	<0.001
MDV: Maldives	2000-2010	-6.732	-6.837	-6.626	<0.001
MDV: Maldives	2010-2019	-4.075	-4.227	-3.923	<0.001
MDV: Maldives	2019-2021	-3.083	-4.481	-1.665	<0.001
MEX: Mexico	2000-2006	-3.865	-3.981	-3.749	<0.001
MEX: Mexico	2006-2010	-0.871	-1.224	-0.517	<0.001
MEX: Mexico	2010-2015	-1.714	-1.936	-1.492	<0.001
MEX: Mexico	2015-2018	0.742	0.026	1.463	0.044
MEX: Mexico	2018-2021	-1.007	-1.36	-0.654	<0.001
MHL: Marshall Islands	2000-2002	-0.149	-0.54	0.244	0.421
MHL: Marshall Islands	2002-2013	-0.461	-0.491	-0.43	<0.001
MHL: Marshall Islands	2013-2016	-1.523	-1.909	-1.136	<0.001
MHL: Marshall Islands	2016-2021	-1.724	-1.811	-1.638	<0.001
MKD: North Macedonia	2000-2002	-0.999	-4.535	2.667	0.542
MKD: North Macedonia	2002-2007	-7.594	-8.65	-6.525	<0.001
MKD: North Macedonia	2007-2011	-5.057	-6.768	-3.315	<0.001
MKD: North Macedonia	2011-2016	0.218	-0.928	1.377	0.675
MKD: North Macedonia	2016-2021	-8.72	-9.459	-7.975	<0.001
MLI: Mali	2000-2005	-2.104	-2.217	-1.99	<0.001
MLI: Mali	2005-2011	-1.433	-1.547	-1.319	<0.001
MLI: Mali	2011-2021	-1.265	-1.305	-1.225	<0.001
MLT: Malta	2000-2006	-1.316	-1.444	-1.188	<0.001
MLT: Malta	2006-2009	1.527	0.75	2.311	0.001
MLT: Malta	2009-2018	-2.173	-2.255	-2.091	<0.001
MLT: Malta	2018-2021	-0.993	-1.373	-0.612	<0.001
MMR: Myanmar	2000-2007	-1.745	-1.845	-1.645	<0.001
MMR: Myanmar	2007-2012	-1.447	-1.685	-1.209	<0.001
MMR: Myanmar	2012-2021	-1.668	-1.736	-1.599	<0.001
MNE: Montenegro	2000-2002	-0.516	-2.29	1.291	0.54
MNE: Montenegro	2002-2007	-3.078	-3.628	-2.525	<0.001
MNE: Montenegro	2007-2013	0.743	0.339	1.149	0.002
MNE: Montenegro	2013-2021	-3.753	-3.942	-3.564	<0.001
MNG: Mongolia	2000-2003	-2.741	-3.682	-1.79	<0.001
MNG: Mongolia	2003-2009	-7.485	-7.886	-7.081	<0.001
MNG: Mongolia	2009-2019	-4.102	-4.272	-3.931	<0.001
MNG: Mongolia	2019-2021	-2.35	-4.231	-0.432	0.021
MOZ: Mozambique	2000-2003	-2.85	-3.047	-2.653	<0.001
MOZ: Mozambique	2003-2007	-2.059	-2.258	-1.861	<0.001
MOZ: Mozambique	2007-2012	-1.542	-1.668	-1.416	<0.001
MOZ: Mozambique	2012-2018	-1.698	-1.787	-1.609	<0.001
MOZ: Mozambique	2018-2021	-0.835	-1.036	-0.634	<0.001
MRT: Mauritania	2000-2003	-1.778	-1.939	-1.616	<0.001
MRT: Mauritania	2003-2008	-2.683	-2.784	-2.582	<0.001
MRT: Mauritania	2008-2017	-1.624	-1.659	-1.589	<0.001
MRT: Mauritania	2017-2021	-1.433	-1.535	-1.33	<0.001
MUS: Mauritius	2000-2002	-7.175	-9.741	-4.535	<0.001
MUS: Mauritius	2002-2005	-3.446	-6.115	-0.7	0.02

MUS: Mauritius	2005-2010	-1.704	-2.572	-0.829	0.002
MUS: Mauritius	2010-2014	2.12	0.699	3.562	0.009
MUS: Mauritius	2014-2021	0.891	0.513	1.269	0.001
MWI: Malawi	2000-2002	-3.288	-3.953	-2.618	<0.001
MWI: Malawi	2002-2005	-1.37	-2.048	-0.687	0.002
MWI: Malawi	2005-2009	0.61	0.263	0.958	0.004
MWI: Malawi	2009-2015	-2.652	-2.802	-2.502	<0.001
MWI: Malawi	2015-2021	-1.133	-1.249	-1.018	<0.001
MYS: Malaysia	2000-2004	-2.405	-3.043	-1.763	<0.001
MYS: Malaysia	2004-2010	0.674	0.208	1.142	0.01
MYS: Malaysia	2010-2014	-0.9	-1.923	0.134	0.079
MYS: Malaysia	2014-2017	8.31	6.086	10.581	<0.001
MYS: Malaysia	2017-2021	-1.799	-2.441	-1.152	<0.001
NAM: Namibia	2000-2008	-1.375	-1.896	-0.852	<0.001
NAM: Namibia	2008-2021	0.636	0.381	0.892	<0.001
NER: Niger	2000-2009	-1.973	-2.019	-1.926	<0.001
NER: Niger	2009-2012	-0.267	-0.779	0.249	0.285
NER: Niger	2012-2021	0.038	-0.009	0.085	0.102
NGA: Nigeria	2000-2002	-1.55	-1.829	-1.271	<0.001
NGA: Nigeria	2002-2007	-1.855	-1.943	-1.767	<0.001
NGA: Nigeria	2007-2012	-1.201	-1.289	-1.112	<0.001
NGA: Nigeria	2012-2018	-0.544	-0.608	-0.481	<0.001
NGA: Nigeria	2018-2021	-0.784	-0.924	-0.643	<0.001
NIC: Nicaragua	2000-2004	-2.246	-2.351	-2.141	<0.001
NIC: Nicaragua	2004-2011	-1.57	-1.626	-1.513	<0.001
NIC: Nicaragua	2011-2014	-1.914	-2.246	-1.58	<0.001
NIC: Nicaragua	2014-2019	-2.768	-2.873	-2.664	<0.001
NIC: Nicaragua	2019-2021	-1.878	-2.21	-1.544	<0.001
NIU: Niue	2000-2004	0.718	0.546	0.89	<0.001
NIU: Niue	2004-2007	-0.633	-1.168	-0.095	0.024
NIU: Niue	2007-2021	-1.821	-1.846	-1.796	<0.001
NLD: Netherlands	2000-2002	-2.753	-5.286	-0.153	0.04
NLD: Netherlands	2002-2013	-6.091	-6.283	-5.898	<0.001
NLD: Netherlands	2013-2017	-4.182	-5.438	-2.909	<0.001
NLD: Netherlands	2017-2021	1.825	0.979	2.679	0.001
NOR: Norway	2000-2004	-4.115	-4.917	-3.306	<0.001
NOR: Norway	2004-2007	-0.46	-3.069	2.219	0.709
NOR: Norway	2007-2016	-2.153	-2.436	-1.869	<0.001
NOR: Norway	2016-2021	-3.979	-4.547	-3.407	<0.001
NPL: Nepal	2000-2005	-2.053	-2.169	-1.936	<0.001
NPL: Nepal	2005-2009	-3.203	-3.46	-2.946	<0.001
NPL: Nepal	2009-2018	-3.678	-3.734	-3.622	<0.001
NPL: Nepal	2018-2021	-2.827	-3.084	-2.569	<0.001
NRU: Nauru	2000-2005	-0.511	-0.625	-0.397	<0.001
NRU: Nauru	2005-2010	-0.196	-0.358	-0.034	0.024
NRU: Nauru	2010-2013	-1.093	-1.6	-0.584	0.001
NRU: Nauru	2013-2017	-1.376	-1.629	-1.123	<0.001

NRU: Nauru	2017-2021	-1.581	-1.74	-1.421	<0.001
NZL: New Zealand	2000-2004	-0.073	-0.333	0.188	0.538
NZL: New Zealand	2004-2007	-2.949	-3.748	-2.145	<0.001
NZL: New Zealand	2007-2010	-1.493	-2.303	-0.676	0.003
NZL: New Zealand	2010-2013	-3.136	-3.932	-2.333	<0.001
NZL: New Zealand	2013-2021	-1.331	-1.42	-1.242	<0.001
OMN: Oman	2000-2005	-2.749	-3.003	-2.496	<0.001
OMN: Oman	2005-2008	0.638	-0.528	1.818	0.244
OMN: Oman	2008-2014	-3.046	-3.299	-2.793	<0.001
OMN: Oman	2014-2018	0.846	0.26	1.435	0.01
OMN: Oman	2018-2021	-0.163	-0.743	0.421	0.538
PAK: Pakistan	2000-2008	-0.231	-0.379	-0.084	0.005
PAK: Pakistan	2008-2012	-1.814	-2.478	-1.145	<0.001
PAK: Pakistan	2012-2018	-1.076	-1.375	-0.775	<0.001
PAK: Pakistan	2018-2021	-1.821	-2.486	-1.153	<0.001
PAN: Panama	2000-2005	-1.493	-1.545	-1.441	<0.001
PAN: Panama	2005-2011	-2.232	-2.283	-2.18	<0.001
PAN: Panama	2011-2016	-2.461	-2.533	-2.388	<0.001
PAN: Panama	2016-2019	-2.175	-2.406	-1.943	<0.001
PAN: Panama	2019-2021	-1.68	-1.913	-1.448	<0.001
PER: Peru	2000-2004	-3.549	-3.844	-3.254	<0.001
PER: Peru	2004-2008	-5.734	-6.188	-5.277	<0.001
PER: Peru	2008-2016	-2.735	-2.861	-2.609	<0.001
PER: Peru	2016-2019	-3.237	-4.169	-2.297	<0.001
PER: Peru	2019-2021	-1.666	-2.613	-0.711	0.004
PHL: Philippines	2000-2002	0.267	-0.171	0.707	0.207
PHL: Philippines	2002-2005	-0.451	-0.886	-0.014	0.044
PHL: Philippines	2005-2014	-2.224	-2.271	-2.177	<0.001
PHL: Philippines	2014-2021	-1.61	-1.668	-1.553	<0.001
PLW: Palau	2000-2003	-1.062	-1.175	-0.948	<0.001
PLW: Palau	2003-2010	-1.362	-1.4	-1.323	<0.001
PLW: Palau	2010-2015	-1.646	-1.718	-1.575	<0.001
PLW: Palau	2015-2021	-1.922	-1.96	-1.884	<0.001
PNG: Papua New Guinea	2000-2003	-0.964	-1.081	-0.848	<0.001
PNG: Papua New Guinea	2003-2009	-0.726	-0.779	-0.674	<0.001
PNG: Papua New Guinea	2009-2015	-0.948	-1.001	-0.896	<0.001
PNG: Papua New Guinea	2015-2018	-0.832	-1.065	-0.598	<0.001
PNG: Papua New Guinea	2018-2021	-1.062	-1.179	-0.946	<0.001
POL: Poland	2000-2012	-3.244	-3.595	-2.891	<0.001
POL: Poland	2012-2016	-5.725	-8.578	-2.783	0.001
POL: Poland	2016-2021	2.398	1.001	3.815	0.002
PRK: North Korea	2000-2004	-4.844	-5.142	-4.545	<0.001
PRK: North Korea	2004-2010	-1.311	-1.53	-1.092	<0.001
PRK: North Korea	2010-2014	-3.689	-4.166	-3.21	<0.001
PRK: North Korea	2014-2021	-2.586	-2.715	-2.457	<0.001
PRT: Portugal	2000-2004	-5.775	-6.575	-4.968	<0.001
PRT: Portugal	2004-2011	-2.968	-3.409	-2.524	<0.001

PRT: Portugal	2011-2018	-0.615	-1.067	-0.161	0.013
PRT: Portugal	2018-2021	-3.543	-4.836	-2.234	<0.001
PRY: Paraguay	2000-2005	-0.563	-0.663	-0.463	<0.001
PRY: Paraguay	2005-2011	-3.373	-3.47	-3.276	<0.001
PRY: Paraguay	2011-2017	-2.979	-3.076	-2.881	<0.001
PRY: Paraguay	2017-2021	-2.081	-2.221	-1.942	<0.001
PSE: Palestinian Territory	2000-2004	-0.711	-0.837	-0.585	<0.001
PSE: Palestinian Territory	2004-2019	-2.033	-2.052	-2.015	<0.001
PSE: Palestinian Territory	2019-2021	-1.622	-2.016	-1.227	<0.001
QAT: Qatar	2000-2003	-2.306	-4.171	-0.404	0.024
QAT: Qatar	2003-2008	1.499	0.269	2.745	0.023
QAT: Qatar	2008-2014	-0.929	-1.78	-0.071	0.037
QAT: Qatar	2014-2019	-13.236	-14.288	-12.172	<0.001
QAT: Qatar	2019-2021	-5.409	-8.987	-1.691	0.01
ROU: Romania	2000-2004	0.336	-0.371	1.049	0.305
ROU: Romania	2004-2009	-7.17	-7.824	-6.511	<0.001
ROU: Romania	2009-2013	-0.706	-1.81	0.41	0.182
ROU: Romania	2013-2018	-5.324	-5.991	-4.652	<0.001
ROU: Romania	2018-2021	3.094	1.947	4.253	<0.001
RUS: Russian Federation	2000-2013	-3.566	-3.852	-3.279	<0.001
RUS: Russian Federation	2013-2016	1.494	-4.1	7.414	0.584
RUS: Russian Federation	2016-2021	-5.512	-6.702	-4.306	<0.001
RWA: Rwanda	2000-2011	-4.066	-4.171	-3.961	<0.001
RWA: Rwanda	2011-2014	1.59	-0.046	3.253	0.056
RWA: Rwanda	2014-2021	-1.527	-1.741	-1.314	<0.001
SAU: Saudi Arabia	2000-2004	-3.085	-3.212	-2.958	<0.001
SAU: Saudi Arabia	2004-2011	-3.455	-3.523	-3.388	<0.001
SAU: Saudi Arabia	2011-2015	-3.844	-4.043	-3.645	<0.001
SAU: Saudi Arabia	2015-2018	-3.398	-3.798	-2.997	<0.001
SAU: Saudi Arabia	2018-2021	-2.341	-2.543	-2.138	<0.001
SDN: Sudan	2000-2003	-1.391	-1.608	-1.174	<0.001
SDN: Sudan	2003-2007	-1.028	-1.245	-0.81	<0.001
SDN: Sudan	2007-2011	-0.668	-0.887	-0.45	<0.001
SDN: Sudan	2011-2017	-1.423	-1.52	-1.326	<0.001
SDN: Sudan	2017-2021	-0.979	-1.117	-0.841	<0.001
SEN: Senegal	2000-2007	-1.693	-1.896	-1.49	<0.001
SEN: Senegal	2007-2013	-0.748	-1.091	-0.405	0.001
SEN: Senegal	2013-2016	-3.46	-4.942	-1.955	0.001
SEN: Senegal	2016-2019	0.255	-1.284	1.818	0.714
SEN: Senegal	2019-2021	-1.784	-3.292	-0.253	0.028
SGP: Singapore	2000-2005	-2.039	-2.893	-1.177	0.001
SGP: Singapore	2005-2008	-3.475	-7.183	0.381	0.071
SGP: Singapore	2008-2011	0.817	-3.056	4.845	0.645
SGP: Singapore	2011-2017	-1.485	-2.344	-0.619	0.004
SGP: Singapore	2017-2021	-4.111	-5.292	-2.916	<0.001
SLB: Solomon Islands	2000-2004	0	-0.176	0.177	0.998
SLB: Solomon Islands	2004-2007	-1.126	-1.677	-0.572	0.001

SLB: Solomon Islands	2007-2021	-1.861	-1.887	-1.836	<0.001
SLE: Sierra Leone	2000-2002	-1.136	-1.377	-0.895	<0.001
SLE: Sierra Leone	2002-2006	-1.574	-1.694	-1.454	<0.001
SLE: Sierra Leone	2006-2009	-2.4	-2.638	-2.162	<0.001
SLE: Sierra Leone	2009-2014	-2.62	-2.695	-2.545	<0.001
SLE: Sierra Leone	2014-2021	-0.976	-1.009	-0.944	<0.001
SLV: El Salvador	2000-2006	-5.864	-5.95	-5.777	<0.001
SLV: El Salvador	2006-2014	-3.492	-3.563	-3.422	<0.001
SLV: El Salvador	2014-2018	-2.84	-3.104	-2.575	<0.001
SLV: El Salvador	2018-2021	-2.229	-2.495	-1.963	<0.001
SMR: San Marino	2000-2004	-4.65	-4.816	-4.483	<0.001
SMR: San Marino	2004-2008	-3.628	-3.893	-3.362	<0.001
SMR: San Marino	2008-2011	-2.174	-2.711	-1.634	<0.001
SMR: San Marino	2011-2016	-1.232	-1.404	-1.06	<0.001
SMR: San Marino	2016-2021	-2.325	-2.445	-2.204	<0.001
SOM: Somalia	2000-2007	0.046	-0.028	0.121	0.205
SOM: Somalia	2007-2021	-1.132	-1.158	-1.106	<0.001
SRB: Serbia	2000-2009	-0.58	-0.92	-0.24	0.003
SRB: Serbia	2009-2012	-2.482	-6.071	1.245	0.173
SRB: Serbia	2012-2021	0.24	-0.103	0.583	0.156
SSD: South Sudan	2000-2004	-1.191	-1.311	-1.07	<0.001
SSD: South Sudan	2004-2008	-1.424	-1.614	-1.234	<0.001
SSD: South Sudan	2008-2011	-1.722	-2.099	-1.343	<0.001
SSD: South Sudan	2011-2014	-0.723	-1.104	-0.339	0.002
SSD: South Sudan	2014-2021	-0.193	-0.244	-0.141	<0.001
STP: Sao Tome and Principe	2000-2002	-1.352	-1.783	-0.92	<0.001
STP: Sao Tome and Principe	2002-2010	-1.83	-1.887	-1.772	<0.001
STP: Sao Tome and Principe	2010-2013	-3.587	-4.008	-3.164	<0.001
STP: Sao Tome and Principe	2013-2018	-4.128	-4.26	-3.995	<0.001
STP: Sao Tome and Principe	2018-2021	-2.809	-3.021	-2.596	<0.001
SUR: Suriname	2000-2004	-0.096	-0.167	-0.025	0.013
SUR: Suriname	2004-2007	-1.133	-1.356	-0.909	<0.001
SUR: Suriname	2007-2012	-1.752	-1.822	-1.682	<0.001
SUR: Suriname	2012-2021	-1.429	-1.449	-1.409	<0.001
SVK: Slovakia	2000-2008	-1.053	-1.253	-0.853	<0.001
SVK: Slovakia	2008-2011	-4.733	-6.481	-2.953	<0.001
SVK: Slovakia	2011-2018	-1.582	-1.889	-1.273	<0.001
SVK: Slovakia	2018-2021	2.977	2.028	3.935	<0.001
SVN: Slovenia	2000-2004	-0.177	-0.551	0.198	0.307
SVN: Slovenia	2004-2011	-3.672	-3.865	-3.479	<0.001
SVN: Slovenia	2011-2016	-0.366	-0.738	0.009	0.054
SVN: Slovenia	2016-2019	-2.595	-3.743	-1.433	0.001
SVN: Slovenia	2019-2021	1.018	-0.173	2.222	0.084
SWE: Sweden	2000-2005	-4.256	-4.832	-3.676	<0.001
SWE: Sweden	2005-2014	-0.529	-0.821	-0.236	0.002
SWE: Sweden	2014-2021	-2.297	-2.649	-1.945	<0.001
SWZ: Eswatini	2000-2002	-0.303	-0.743	0.139	0.159

SWZ: Eswatini	2002-2007	-1.472	-1.61	-1.334	<0.001
SWZ: Eswatini	2007-2014	0.109	0.034	0.183	0.009
SWZ: Eswatini	2014-2021	-0.849	-0.908	-0.791	<0.001
SYC: Seychelles	2000-2003	1.019	0.715	1.324	<0.001
SYC: Seychelles	2003-2009	0.223	0.088	0.358	0.005
SYC: Seychelles	2009-2013	1.647	1.341	1.954	<0.001
SYC: Seychelles	2013-2017	0.029	-0.272	0.331	0.83
SYC: Seychelles	2017-2021	-0.92	-1.109	-0.731	<0.001
SYR: Syria	2000-2007	-1.216	-1.358	-1.073	<0.001
SYR: Syria	2007-2010	-0.396	-1.465	0.685	0.436
SYR: Syria	2010-2016	0.701	0.458	0.944	<0.001
SYR: Syria	2016-2021	-0.328	-0.569	-0.087	0.012
TCD: Chad	2000-2015	-1.383	-1.417	-1.35	<0.001
TCD: Chad	2015-2021	-0.706	-0.84	-0.572	<0.001
TGO: Togo	2000-2004	-1.381	-1.451	-1.311	<0.001
TGO: Togo	2004-2008	-1.141	-1.252	-1.03	<0.001
TGO: Togo	2008-2013	-1.35	-1.42	-1.28	<0.001
TGO: Togo	2013-2021	-1.277	-1.301	-1.253	<0.001
THA: Thailand	2000-2005	-3.181	-3.296	-3.065	<0.001
THA: Thailand	2005-2015	-2.718	-2.765	-2.671	<0.001
THA: Thailand	2015-2021	-2.21	-2.298	-2.121	<0.001
TJK: Tajikistan	2000-2004	-2.443	-2.519	-2.366	<0.001
TJK: Tajikistan	2004-2009	-1.391	-1.469	-1.313	<0.001
TJK: Tajikistan	2009-2014	-2.357	-2.434	-2.28	<0.001
TJK: Tajikistan	2014-2021	-1.749	-1.782	-1.717	<0.001
TKM: Turkmenistan	2000-2002	-1.122	-1.663	-0.579	0.001
TKM: Turkmenistan	2002-2008	-1.939	-2.059	-1.819	<0.001
TKM: Turkmenistan	2008-2012	-0.793	-1.065	-0.521	<0.001
TKM: Turkmenistan	2012-2018	0.353	0.23	0.476	<0.001
TKM: Turkmenistan	2018-2021	-0.641	-0.913	-0.368	0.001
TLS: Timor-Leste	2000-2005	-2.448	-2.77	-2.125	<0.001
TLS: Timor-Leste	2005-2009	-4.019	-4.727	-3.306	<0.001
TLS: Timor-Leste	2009-2013	-2.334	-3.054	-1.609	<0.001
TLS: Timor-Leste	2013-2021	-0.201	-0.362	-0.039	0.019
TON: Tonga	2000-2008	-1.709	-1.779	-1.639	<0.001
TON: Tonga	2008-2011	-1.217	-1.861	-0.569	0.002
TON: Tonga	2011-2016	-0.476	-0.682	-0.27	<0.001
TON: Tonga	2016-2021	-1.391	-1.536	-1.247	<0.001
TTO: Trinidad and Tobago	2000-2003	-0.602	-0.809	-0.393	<0.001
TTO: Trinidad and Tobago	2003-2007	-1.305	-1.511	-1.098	<0.001
TTO: Trinidad and Tobago	2007-2010	-2.359	-2.767	-1.95	<0.001
TTO: Trinidad and Tobago	2010-2021	-1.602	-1.63	-1.574	<0.001
TUN: Tunisia	2000-2004	-3.184	-3.324	-3.044	<0.001
TUN: Tunisia	2004-2007	-2.311	-2.757	-1.863	<0.001
TUN: Tunisia	2007-2010	-1.59	-2.039	-1.138	<0.001
TUN: Tunisia	2010-2013	-0.797	-1.25	-0.342	0.004
TUN: Tunisia	2013-2021	-1.035	-1.084	-0.985	<0.001

TUR: Turkiye	2000-2004	-5.213	-6.26	-4.154	<0.001
TUR: Turkiye	2004-2010	-7.348	-8.073	-6.617	<0.001
TUR: Turkiye	2010-2017	-3.577	-4.148	-3.002	<0.001
TUR: Turkiye	2017-2021	-1.612	-2.699	-0.512	0.008
TUV: Tuvalu	2000-2003	-0.853	-1.05	-0.657	<0.001
TUV: Tuvalu	2003-2006	-1.663	-2.053	-1.273	<0.001
TUV: Tuvalu	2006-2011	-3.524	-3.645	-3.403	<0.001
TUV: Tuvalu	2011-2015	-2.962	-3.154	-2.77	<0.001
TUV: Tuvalu	2015-2021	-2.019	-2.085	-1.954	<0.001
TZA: Tanzania	2000-2003	-3.274	-3.617	-2.929	<0.001
TZA: Tanzania	2003-2007	-2.121	-2.468	-1.773	<0.001
TZA: Tanzania	2007-2012	0.026	-0.199	0.251	0.804
TZA: Tanzania	2012-2021	-1.839	-1.903	-1.776	<0.001
UGA: Uganda	2000-2005	-1.757	-1.855	-1.659	<0.001
UGA: Uganda	2005-2010	-0.025	-0.166	0.116	0.701
UGA: Uganda	2010-2019	-1.872	-1.919	-1.824	<0.001
UGA: Uganda	2019-2021	-7.396	-7.807	-6.982	<0.001
UKR: Ukraine	2000-2002	-3.097	-3.668	-2.523	<0.001
UKR: Ukraine	2002-2007	-3.866	-4.046	-3.687	<0.001
UKR: Ukraine	2007-2014	-1.778	-1.876	-1.68	<0.001
UKR: Ukraine	2014-2017	-0.754	-1.338	-0.166	0.018
UKR: Ukraine	2017-2021	1.278	1.089	1.467	<0.001
URY: Uruguay	2000-2006	-2.234	-2.918	-1.546	<0.001
URY: Uruguay	2006-2009	-8.083	-11.82	-4.189	0.001
URY: Uruguay	2009-2021	-0.336	-0.58	-0.091	0.011
USA: United States	2000-2005	-2.174	-2.786	-1.558	<0.001
USA: United States	2005-2008	0.562	-2.221	3.424	0.675
USA: United States	2008-2021	-0.849	-0.995	-0.703	<0.001
UZB: Uzbekistan	2000-2003	-2.193	-2.403	-1.983	<0.001
UZB: Uzbekistan	2003-2007	-2.835	-3.043	-2.626	<0.001
UZB: Uzbekistan	2007-2011	-4.289	-4.494	-4.083	<0.001
UZB: Uzbekistan	2011-2015	-4.03	-4.236	-3.823	<0.001
UZB: Uzbekistan	2015-2021	-3.357	-3.427	-3.286	<0.001
VCT: Saint Vincent and the Grenadines	2000-2002	1.291	0.715	1.872	0.001
VCT: Saint Vincent and the Grenadines	2002-2007	2.909	2.724	3.095	<0.001
VCT: Saint Vincent and the Grenadines	2007-2010	3.633	3.043	4.227	<0.001
VCT: Saint Vincent and the Grenadines	2010-2015	0.105	-0.076	0.286	0.217
VCT: Saint Vincent and the Grenadines	2015-2021	-2.319	-2.413	-2.224	<0.001
VEN: Venezuela	2000-2013	-0.065	-0.307	0.178	0.577
VEN: Venezuela	2013-2016	5.081	0.328	10.06	0.038
VEN: Venezuela	2016-2021	-0.212	-1.239	0.826	0.667
VNM: Vietnam	2000-2003	-2.39	-2.565	-2.214	<0.001
VNM: Vietnam	2003-2006	-1.923	-2.275	-1.569	<0.001

VNM: Vietnam	2006-2011	-1.3	-1.412	-1.188	<0.001
VNM: Vietnam	2011-2016	-1.508	-1.62	-1.396	<0.001
VNM: Vietnam	2016-2021	-1.028	-1.107	-0.948	<0.001
VUT: Vanuatu	2000-2002	-0.517	-0.955	-0.076	0.027
VUT: Vanuatu	2002-2006	0.214	-0.007	0.436	0.056
VUT: Vanuatu	2006-2013	-0.021	-0.096	0.054	0.532
VUT: Vanuatu	2013-2017	-1.054	-1.272	-0.835	<0.001
VUT: Vanuatu	2017-2021	-1.699	-1.836	-1.561	<0.001
WSM: Samoa	2000-2004	-1.673	-1.892	-1.453	<0.001
WSM: Samoa	2004-2012	-0.21	-0.304	-0.116	<0.001
WSM: Samoa	2012-2017	-1.024	-1.244	-0.802	<0.001
WSM: Samoa	2017-2021	-1.782	-2.001	-1.563	<0.001
YEM: Yemen	2000-2008	-1.485	-1.509	-1.46	<0.001
YEM: Yemen	2008-2011	-0.232	-0.46	-0.004	0.047
YEM: Yemen	2011-2016	2.642	2.568	2.716	<0.001
YEM: Yemen	2016-2021	-0.327	-0.378	-0.276	<0.001
ZAF: South Africa	2000-2003	-1.637	-1.814	-1.46	<0.001
ZAF: South Africa	2003-2013	-1.919	-1.952	-1.887	<0.001
ZAF: South Africa	2013-2016	-0.393	-0.751	-0.033	0.036
ZAF: South Africa	2016-2019	0.072	-0.288	0.434	0.655
ZAF: South Africa	2019-2021	-0.342	-0.7	0.018	0.06
ZMB: Zambia	2000-2008	-2.012	-2.305	-1.718	<0.001
ZMB: Zambia	2008-2012	-3.167	-4.489	-1.828	<0.001
ZMB: Zambia	2012-2016	-0.905	-2.257	0.466	0.173
ZMB: Zambia	2016-2021	-2.235	-2.834	-1.633	<0.001
ZWE: Zimbabwe	2000-2008	1.076	0.716	1.438	<0.001
ZWE: Zimbabwe	2008-2012	-2.496	-4.078	-0.888	0.006
ZWE: Zimbabwe	2012-2017	-7.684	-8.634	-6.724	<0.001
ZWE: Zimbabwe	2017-2021	6.059	4.967	7.162	<0.001

Table S3. Results from the joinpoint regression analysis of neonatal mortality rate

Country	Segment	APC	APC 95% LCL	APC 95% UCL	p-value
AFG: Afghanistan	2000-2003	-2.074	-2.159	-1.989	<0.001
AFG: Afghanistan	2003-2007	-2.448	-2.533	-2.363	<0.001
AFG: Afghanistan	2007-2010	-2.903	-3.072	-2.734	<0.001
AFG: Afghanistan	2010-2017	-3.131	-3.159	-3.102	<0.001
AFG: Afghanistan	2017-2021	-2.685	-2.738	-2.631	<0.001
AGO: Angola	2000-2003	-2.542	-2.811	-2.272	<0.001
AGO: Angola	2003-2015	-3.487	-3.523	-3.451	<0.001
AGO: Angola	2015-2021	-2.282	-2.373	-2.191	<0.001
AIA: Anguilla	2000-2003	-4.7	-4.87	-4.529	<0.001
AIA: Anguilla	2003-2010	-4.424	-4.482	-4.366	<0.001
AIA: Anguilla	2010-2013	-4.16	-4.502	-3.817	<0.001
AIA: Anguilla	2013-2021	-3.538	-3.575	-3.5	<0.001
ALB: Albania	2000-2006	-3.078	-3.309	-2.847	<0.001
ALB: Albania	2006-2011	-9.574	-9.976	-9.169	<0.001
ALB: Albania	2011-2014	-1.551	-2.93	-0.152	0.034
ALB: Albania	2014-2019	4.212	3.748	4.678	<0.001
ALB: Albania	2019-2021	-0.468	-1.863	0.946	0.465
AND: Andorra	2000-2003	-5.606	-5.903	-5.309	<0.001
AND: Andorra	2003-2009	-3.904	-4.04	-3.769	<0.001
AND: Andorra	2009-2017	-4.414	-4.494	-4.333	<0.001
AND: Andorra	2017-2021	-3.943	-4.134	-3.751	<0.001
ARE: United Arab Emirates	2000-2005	-2.304	-2.364	-2.245	<0.001
ARE: United Arab Emirates	2005-2008	-2.05	-2.317	-1.782	<0.001
ARE: United Arab Emirates	2008-2012	-2.699	-2.832	-2.566	<0.001
ARE: United Arab Emirates	2012-2017	-2.277	-2.362	-2.193	<0.001
ARE: United Arab Emirates	2017-2021	-2.731	-2.815	-2.647	<0.001
ARG: Argentina	2000-2002	-1.621	-6.21	3.193	0.467
ARG: Argentina	2002-2008	-4.896	-5.906	-3.874	<0.001
ARG: Argentina	2008-2013	-1.701	-3.175	-0.205	0.03
ARG: Argentina	2013-2021	-3.974	-4.474	-3.473	<0.001
ARM: Armenia	2000-2005	-4.775	-4.802	-4.749	<0.001
ARM: Armenia	2005-2010	-4.975	-5.012	-4.938	<0.001
ARM: Armenia	2010-2014	-4.872	-4.93	-4.813	<0.001
ARM: Armenia	2014-2017	-4.766	-4.883	-4.648	<0.001
ARM: Armenia	2017-2021	-4.879	-4.916	-4.842	<0.001
ATG: Antigua and Barbuda	2000-2003	-2.503	-2.78	-2.226	<0.001
ATG: Antigua and Barbuda	2003-2006	-4.768	-5.307	-4.225	<0.001
ATG: Antigua and Barbuda	2006-2013	-7.02	-7.109	-6.931	<0.001
ATG: Antigua and Barbuda	2013-2016	-5.043	-5.581	-4.503	<0.001
ATG: Antigua and Barbuda	2016-2021	-3.728	-3.85	-3.605	<0.001
AUS: Australia	2000-2004	-1.39	-1.618	-1.161	<0.001
AUS: Australia	2004-2007	-2.123	-2.837	-1.403	<0.001
AUS: Australia	2007-2013	-4.157	-4.314	-4	<0.001
AUS: Australia	2013-2016	-1.437	-2.156	-0.713	0.002
AUS: Australia	2016-2021	0.44	0.275	0.604	<0.001

AUT: Austria	2000-2003	-0.905	-1.166	-0.643	<0.001
AUT: Austria	2003-2007	-2.893	-3.149	-2.637	<0.001
AUT: Austria	2007-2015	-2.219	-2.288	-2.15	<0.001
AUT: Austria	2015-2018	-0.403	-0.927	0.124	0.116
AUT: Austria	2018-2021	2.036	1.767	2.306	<0.001
AZE: Azerbaijan	2000-2004	-4.717	-4.873	-4.56	<0.001
AZE: Azerbaijan	2004-2010	-5.288	-5.398	-5.178	<0.001
AZE: Azerbaijan	2010-2016	-7.806	-7.913	-7.699	<0.001
AZE: Azerbaijan	2016-2019	-6.029	-6.514	-5.54	<0.001
AZE: Azerbaijan	2019-2021	-4.46	-4.953	-3.963	<0.001
BDI: Burundi	2000-2004	-1.833	-1.962	-1.704	<0.001
BDI: Burundi	2004-2007	-2.843	-3.246	-2.438	<0.001
BDI: Burundi	2007-2015	-3.459	-3.513	-3.405	<0.001
BDI: Burundi	2015-2019	-2.481	-2.683	-2.277	<0.001
BDI: Burundi	2019-2021	-2.104	-2.51	-1.696	<0.001
BEL: Belgium	2000-2011	-2.33	-2.423	-2.237	<0.001
BEL: Belgium	2011-2014	-0.379	-1.777	1.039	0.574
BEL: Belgium	2014-2021	1.133	0.942	1.324	<0.001
BEN: Benin	2000-2002	-1.955	-2.115	-1.794	<0.001
BEN: Benin	2002-2005	-1.636	-1.797	-1.475	<0.001
BEN: Benin	2005-2011	-1	-1.037	-0.964	<0.001
BEN: Benin	2011-2017	-1.304	-1.341	-1.268	<0.001
BEN: Benin	2017-2021	-1.532	-1.582	-1.481	<0.001
BFA: Burkina Faso	2000-2003	-2.029	-2.169	-1.889	<0.001
BFA: Burkina Faso	2003-2008	-3.135	-3.223	-3.047	<0.001
BFA: Burkina Faso	2008-2011	-2.212	-2.491	-1.932	<0.001
BFA: Burkina Faso	2011-2021	-1.846	-1.868	-1.824	<0.001
BGD: Bangladesh	2000-2002	-4.199	-4.465	-3.932	<0.001
BGD: Bangladesh	2002-2012	-3.909	-3.934	-3.885	<0.001
BGD: Bangladesh	2012-2015	-4.651	-4.916	-4.386	<0.001
BGD: Bangladesh	2015-2019	-6.43	-6.56	-6.3	<0.001
BGD: Bangladesh	2019-2021	-5.864	-6.125	-5.601	<0.001
BGR: Bulgaria	2000-2002	-4.022	-5.633	-2.385	<0.001
BGR: Bulgaria	2002-2007	-6.145	-6.646	-5.641	<0.001
BGR: Bulgaria	2007-2011	-1.338	-2.169	-0.5	0.005
BGR: Bulgaria	2011-2021	-5.081	-5.206	-4.956	<0.001
BHR: Bahrain	2000-2002	-6.767	-7.437	-6.092	<0.001
BHR: Bahrain	2002-2006	-4.377	-4.721	-4.032	<0.001
BHR: Bahrain	2006-2009	-2.891	-3.589	-2.189	<0.001
BHR: Bahrain	2009-2015	0.297	0.136	0.459	0.003
BHR: Bahrain	2015-2021	-1.093	-1.213	-0.972	<0.001
BHS: Bahamas	2000-2004	2.001	1.852	2.15	<0.001
BHS: Bahamas	2004-2007	-0.046	-0.507	0.417	0.824
BHS: Bahamas	2007-2013	-2.57	-2.671	-2.469	<0.001
BHS: Bahamas	2013-2019	-0.086	-0.189	0.018	0.092
BHS: Bahamas	2019-2021	-1.654	-2.107	-1.198	<0.001
BIH: Bosnia and Herzegovina	2000-2005	-4.642	-4.911	-4.372	<0.001

BIH: Bosnia and Herzegovina	2005-2009	-0.945	-1.569	-0.318	0.006
BIH: Bosnia and Herzegovina	2009-2021	-1.932	-2.006	-1.859	<0.001
BLR: Belarus	2000-2002	-14.484	-17.128	-11.755	<0.001
BLR: Belarus	2002-2005	-5.452	-8.376	-2.435	0.003
BLR: Belarus	2005-2010	-11.13	-12.009	-10.243	<0.001
BLR: Belarus	2010-2016	-2.542	-3.224	-1.855	<0.001
BLR: Belarus	2016-2021	-10.742	-11.367	-10.113	<0.001
BLZ: Belize	2000-2007	-3.712	-3.884	-3.54	<0.001
BLZ: Belize	2007-2012	3.396	2.96	3.834	<0.001
BLZ: Belize	2012-2015	-2.133	-3.433	-0.815	0.005
BLZ: Belize	2015-2021	-5.331	-5.545	-5.117	<0.001
BOL: Bolivia	2000-2007	-2.29	-2.4	-2.181	<0.001
BOL: Bolivia	2007-2011	-4.218	-4.618	-3.816	<0.001
BOL: Bolivia	2011-2016	-5.216	-5.466	-4.964	<0.001
BOL: Bolivia	2016-2021	-3.923	-4.103	-3.743	<0.001
BRA: Brazil	2000-2011	-5.101	-5.421	-4.78	<0.001
BRA: Brazil	2011-2021	-2.006	-2.388	-1.623	<0.001
BRB: Barbados	2000-2006	1.488	1.355	1.621	<0.001
BRB: Barbados	2006-2009	0.054	-0.717	0.831	0.876
BRB: Barbados	2009-2013	-0.883	-1.266	-0.499	0.001
BRB: Barbados	2013-2016	-1.979	-2.734	-1.218	<0.001
BRB: Barbados	2016-2021	-2.917	-3.085	-2.749	<0.001
BRN: Brunei Darussalam	2000-2003	0.632	0.176	1.091	0.013
BRN: Brunei Darussalam	2003-2010	-0.779	-0.931	-0.626	<0.001
BRN: Brunei Darussalam	2010-2014	0.851	0.394	1.311	0.003
BRN: Brunei Darussalam	2014-2019	4.045	3.746	4.344	<0.001
BRN: Brunei Darussalam	2019-2021	1.228	0.312	2.152	0.015
BTN: Bhutan	2000-2004	-3.033	-3.108	-2.958	<0.001
BTN: Bhutan	2004-2007	-3.417	-3.653	-3.181	<0.001
BTN: Bhutan	2007-2013	-3.779	-3.832	-3.727	<0.001
BTN: Bhutan	2013-2017	-3.969	-4.087	-3.852	<0.001
BTN: Bhutan	2017-2021	-3.47	-3.544	-3.395	<0.001
BWA: Botswana	2000-2002	18.6	16.153	21.098	<0.001
BWA: Botswana	2002-2005	22.35	19.826	24.927	<0.001
BWA: Botswana	2005-2008	7.73	5.508	9.999	<0.001
BWA: Botswana	2008-2011	-0.88	-2.925	1.208	0.357
BWA: Botswana	2011-2021	-3.327	-3.484	-3.17	<0.001
CAF: Central African Republic	2000-2005	-1.635	-1.663	-1.606	<0.001
CAF: Central African Republic	2005-2009	-1.891	-1.954	-1.827	<0.001
CAF: Central African Republic	2009-2012	-1.406	-1.533	-1.279	<0.001
CAF: Central African Republic	2012-2018	-0.959	-0.988	-0.931	<0.001
CAF: Central African Republic	2018-2021	-1.36	-1.423	-1.296	<0.001
CAN: Canada	2000-2004	1.354	1.241	1.467	<0.001
CAN: Canada	2004-2007	-1.399	-1.746	-1.05	<0.001
CAN: Canada	2007-2012	-0.544	-0.655	-0.434	<0.001
CAN: Canada	2012-2017	-1.258	-1.368	-1.148	<0.001
CAN: Canada	2017-2021	-0.46	-0.571	-0.349	<0.001

CHE: Switzerland	2000-2002	-0.668	-1.116	-0.218	0.009
CHE: Switzerland	2002-2005	-1.44	-1.885	-0.994	<0.001
CHE: Switzerland	2005-2008	-0.489	-0.938	-0.038	0.037
CHE: Switzerland	2008-2015	-1.161	-1.236	-1.085	<0.001
CHE: Switzerland	2015-2021	-1.579	-1.654	-1.503	<0.001
CHL: Chile	2000-2002	-3.652	-5.328	-1.946	0.001
CHL: Chile	2002-2005	1.011	-0.746	2.8	0.223
CHL: Chile	2005-2014	-0.457	-0.648	-0.267	0.001
CHL: Chile	2014-2018	-1.713	-2.571	-0.846	0.002
CHL: Chile	2018-2021	-4.2	-5.037	-3.355	<0.001
CHN: China	2000-2003	-7.434	-7.671	-7.196	<0.001
CHN: China	2003-2010	-9.671	-9.749	-9.593	<0.001
CHN: China	2010-2018	-8.589	-8.652	-8.527	<0.001
CHN: China	2018-2021	-7.819	-8.055	-7.582	<0.001
CIV: CÃ´te d'Ivoire	2000-2002	-1.179	-1.281	-1.076	<0.001
CIV: CÃ´te d'Ivoire	2002-2006	-1.536	-1.587	-1.485	<0.001
CIV: CÃ´te d'Ivoire	2006-2010	-1.795	-1.846	-1.743	<0.001
CIV: CÃ´te d'Ivoire	2010-2017	-1.72	-1.737	-1.703	<0.001
CIV: CÃ´te d'Ivoire	2017-2021	-1.948	-1.98	-1.915	<0.001
CMR: Cameroon	2000-2002	-2.084	-2.32	-1.848	<0.001
CMR: Cameroon	2002-2007	-0.747	-0.823	-0.672	<0.001
CMR: Cameroon	2007-2012	-1.175	-1.25	-1.1	<0.001
CMR: Cameroon	2012-2015	-1.666	-1.903	-1.429	<0.001
CMR: Cameroon	2015-2021	-2.195	-2.234	-2.155	<0.001
COD: Congo DRC	2000-2002	-0.782	-1.063	-0.5	<0.001
COD: Congo DRC	2002-2005	-1.417	-1.697	-1.138	<0.001
COD: Congo DRC	2005-2009	-2.241	-2.379	-2.102	<0.001
COD: Congo DRC	2009-2012	-2.013	-2.29	-1.735	<0.001
COD: Congo DRC	2012-2021	-1.806	-1.832	-1.781	<0.001
COG: Congo	2000-2002	-1.799	-2.223	-1.374	<0.001
COG: Congo	2002-2008	-3.533	-3.626	-3.44	<0.001
COG: Congo	2008-2011	-1.991	-2.413	-1.566	<0.001
COG: Congo	2011-2014	-1.557	-1.982	-1.131	<0.001
COG: Congo	2014-2021	-2.07	-2.127	-2.014	<0.001
COK: Cook Islands	2000-2004	-4.859	-4.937	-4.78	<0.001
COK: Cook Islands	2004-2010	-5.311	-5.366	-5.256	<0.001
COK: Cook Islands	2010-2015	-4.091	-4.17	-4.013	<0.001
COK: Cook Islands	2015-2021	-3.479	-3.522	-3.437	<0.001
COL: Colombia	2000-2007	-2.807	-2.847	-2.768	<0.001
COL: Colombia	2007-2021	-3.225	-3.239	-3.212	<0.001
COM: Comoros	2000-2005	-1.4	-1.472	-1.328	<0.001
COM: Comoros	2005-2011	-1.994	-2.065	-1.922	<0.001
COM: Comoros	2011-2021	-2.359	-2.384	-2.334	<0.001
CPV: Cabo Verde	2000-2003	-4.636	-5.018	-4.253	<0.001
CPV: Cabo Verde	2003-2009	0.273	0.094	0.453	0.008
CPV: Cabo Verde	2009-2013	-4.057	-4.441	-3.672	<0.001
CPV: Cabo Verde	2013-2018	-7.011	-7.246	-6.774	<0.001

CPV: Cabo Verde	2018-2021	-5.14	-5.519	-4.758	<0.001
CRI: Costa Rica	2000-2002	-2.363	-3.475	-1.237	0.001
CRI: Costa Rica	2002-2018	-1.224	-1.272	-1.176	<0.001
CRI: Costa Rica	2018-2021	-3.639	-4.19	-3.085	<0.001
CUB: Cuba	2000-2002	-3.287	-4.37	-2.191	<0.001
CUB: Cuba	2002-2005	-5.549	-6.606	-4.479	<0.001
CUB: Cuba	2005-2009	-3.08	-3.624	-2.533	<0.001
CUB: Cuba	2009-2016	-3.843	-4.026	-3.66	<0.001
CUB: Cuba	2016-2021	0.813	0.56	1.067	<0.001
CYP: Cyprus	2000-2008	-6.524	-6.67	-6.378	<0.001
CYP: Cyprus	2008-2013	-4.538	-4.969	-4.105	<0.001
CYP: Cyprus	2013-2016	-2.456	-3.843	-1.049	0.003
CYP: Cyprus	2016-2021	0.511	0.189	0.833	0.005
CZE: Czech Republic	2000-2006	-3.938	-4.276	-3.598	<0.001
CZE: Czech Republic	2006-2010	-5.656	-6.636	-4.667	<0.001
CZE: Czech Republic	2010-2013	-1.964	-3.989	0.105	0.06
CZE: Czech Republic	2013-2018	0.958	0.294	1.627	0.01
CZE: Czech Republic	2018-2021	-4.111	-5.107	-3.105	<0.001
DEU: Germany	2000-2007	-1.366	-1.489	-1.242	<0.001
DEU: Germany	2007-2010	-3.207	-4.11	-2.296	<0.001
DEU: Germany	2010-2019	-0.084	-0.186	0.018	0.096
DEU: Germany	2019-2021	-1.864	-2.779	-0.94	0.001
DJI: Djibouti	2000-2008	-1.434	-1.475	-1.393	<0.001
DJI: Djibouti	2008-2016	-1.981	-2.031	-1.931	<0.001
DJI: Djibouti	2016-2021	-2.254	-2.337	-2.17	<0.001
DMA: Dominica	2000-2006	4.041	3.953	4.129	<0.001
DMA: Dominica	2006-2014	5.041	4.971	5.111	<0.001
DMA: Dominica	2014-2018	3.537	3.279	3.796	<0.001
DMA: Dominica	2018-2021	1.119	0.867	1.372	<0.001
DNK: Denmark	2000-2009	-2.295	-2.482	-2.107	<0.001
DNK: Denmark	2009-2012	2.779	0.644	4.96	0.015
DNK: Denmark	2012-2015	-0.006	-2.084	2.115	0.995
DNK: Denmark	2015-2021	-3.351	-3.693	-3.008	<0.001
DOM: Dominican Republic	2000-2004	-0.097	-0.227	0.034	0.13
DOM: Dominican Republic	2004-2009	1.035	0.903	1.167	<0.001
DOM: Dominican Republic	2009-2017	0.079	0.024	0.135	0.009
DOM: Dominican Republic	2017-2021	-1.591	-1.719	-1.462	<0.001
DZA: Algeria	2000-2008	-2.518	-2.707	-2.329	<0.001
DZA: Algeria	2008-2011	-1.781	-3.509	-0.023	0.048
DZA: Algeria	2011-2016	-2.834	-3.378	-2.287	<0.001
DZA: Algeria	2016-2019	2.33	0.531	4.162	0.017
DZA: Algeria	2019-2021	-1.564	-3.295	0.198	0.075
ECU: Ecuador	2000-2002	-5.258	-5.752	-4.762	<0.001
ECU: Ecuador	2002-2011	-4.369	-4.424	-4.314	<0.001
ECU: Ecuador	2011-2014	-4.102	-4.602	-3.599	<0.001
ECU: Ecuador	2014-2017	-2.697	-3.204	-2.187	<0.001
ECU: Ecuador	2017-2021	-0.925	-1.088	-0.761	<0.001

EGY: Egypt	2000-2005	-3.155	-3.232	-3.078	<0.001
EGY: Egypt	2005-2014	-4.334	-4.371	-4.297	<0.001
EGY: Egypt	2014-2021	-3.489	-3.535	-3.443	<0.001
ERI: Eritrea	2000-2006	-1.807	-1.851	-1.764	<0.001
ERI: Eritrea	2006-2011	-2.394	-2.475	-2.313	<0.001
ERI: Eritrea	2011-2021	-2.141	-2.161	-2.121	<0.001
ESP: Spain	2000-2002	-1.628	-2.32	-0.931	0.001
ESP: Spain	2002-2008	-3.716	-3.868	-3.564	<0.001
ESP: Spain	2008-2012	-1.043	-1.392	-0.693	<0.001
ESP: Spain	2012-2015	-2.182	-2.87	-1.49	<0.001
ESP: Spain	2015-2021	-1.364	-1.481	-1.246	<0.001
EST: Estonia	2000-2006	-8.737	-8.765	-8.708	<0.001
EST: Estonia	2006-2010	-9.113	-9.199	-9.028	<0.001
EST: Estonia	2010-2013	-8.063	-8.235	-7.891	<0.001
EST: Estonia	2013-2019	-7.519	-7.558	-7.481	<0.001
EST: Estonia	2019-2021	-6.71	-6.885	-6.535	<0.001
ETH: Ethiopia	2000-2005	-2.074	-2.12	-2.028	<0.001
ETH: Ethiopia	2005-2008	-2.59	-2.795	-2.384	<0.001
ETH: Ethiopia	2008-2011	-2.991	-3.195	-2.786	<0.001
ETH: Ethiopia	2011-2018	-3.517	-3.551	-3.482	<0.001
ETH: Ethiopia	2018-2021	-3.021	-3.123	-2.919	<0.001
FIN: Finland	2000-2007	-3.444	-3.554	-3.334	<0.001
FIN: Finland	2007-2013	-4.846	-5.027	-4.665	<0.001
FIN: Finland	2013-2019	-1.221	-1.409	-1.033	<0.001
FIN: Finland	2019-2021	-2.244	-3.072	-1.41	<0.001
FJI: Fiji	2000-2005	3.811	3.268	4.357	<0.001
FJI: Fiji	2005-2011	-4.539	-5.038	-4.037	<0.001
FJI: Fiji	2011-2014	1.856	-0.505	4.273	0.108
FJI: Fiji	2014-2019	6.518	5.731	7.311	<0.001
FJI: Fiji	2019-2021	3.06	0.671	5.505	0.018
FRA: France	2000-2002	-0.622	-1.614	0.379	0.189
FRA: France	2002-2005	-3.717	-4.678	-2.747	<0.001
FRA: France	2005-2012	-0.795	-0.963	-0.626	<0.001
FRA: France	2012-2018	2.162	1.933	2.391	<0.001
FRA: France	2018-2021	-0.885	-1.38	-0.387	0.003
FSM: Micronesia	2000-2004	-1.41	-1.579	-1.241	<0.001
FSM: Micronesia	2004-2008	-0.328	-0.597	-0.057	0.023
FSM: Micronesia	2008-2012	-2.04	-2.305	-1.774	<0.001
FSM: Micronesia	2012-2016	-2.991	-3.254	-2.728	<0.001
FSM: Micronesia	2016-2021	-3.379	-3.496	-3.262	<0.001
GAB: Gabon	2000-2004	-1.135	-1.224	-1.046	<0.001
GAB: Gabon	2004-2009	-1.454	-1.543	-1.365	<0.001
GAB: Gabon	2009-2015	-1.97	-2.032	-1.908	<0.001
GAB: Gabon	2015-2019	-2.243	-2.382	-2.104	<0.001
GAB: Gabon	2019-2021	-2.539	-2.816	-2.262	<0.001
GBR: United Kingdom	2000-2006	-1.603	-1.7	-1.506	<0.001
GBR: United Kingdom	2006-2014	-2.82	-2.896	-2.745	<0.001

GBR: United Kingdom	2014-2019	0.96	0.774	1.147	<0.001
GBR: United Kingdom	2019-2021	-0.865	-1.442	-0.284	0.007
GEO: Georgia	2000-2003	-6.009	-6.626	-5.389	<0.001
GEO: Georgia	2003-2009	-8.836	-9.104	-8.567	<0.001
GEO: Georgia	2009-2017	-7.825	-7.987	-7.663	<0.001
GEO: Georgia	2017-2021	-1.717	-2.125	-1.307	<0.001
GHA: Ghana	2000-2003	-2.137	-2.2	-2.074	<0.001
GHA: Ghana	2003-2007	-1.174	-1.238	-1.11	<0.001
GHA: Ghana	2007-2010	-1.989	-2.116	-1.863	<0.001
GHA: Ghana	2010-2016	-2.574	-2.602	-2.546	<0.001
GHA: Ghana	2016-2021	-2.42	-2.448	-2.392	<0.001
GIN: Guinea	2000-2002	-3.98	-4.274	-3.685	<0.001
GIN: Guinea	2002-2007	-2.421	-2.516	-2.327	<0.001
GIN: Guinea	2007-2015	-1.43	-1.471	-1.39	<0.001
GIN: Guinea	2015-2021	-1.142	-1.193	-1.09	<0.001
GMB: Gambia	2000-2005	-1.581	-1.634	-1.528	<0.001
GMB: Gambia	2005-2009	-2.032	-2.15	-1.914	<0.001
GMB: Gambia	2009-2013	-2.327	-2.445	-2.21	<0.001
GMB: Gambia	2013-2019	-2.604	-2.657	-2.552	<0.001
GMB: Gambia	2019-2021	-2.304	-2.539	-2.069	<0.001
GNB: Guinea-Bissau	2000-2006	-1.546	-1.609	-1.483	<0.001
GNB: Guinea-Bissau	2006-2009	-2.413	-2.782	-2.044	<0.001
GNB: Guinea-Bissau	2009-2012	-3.009	-3.375	-2.641	<0.001
GNB: Guinea-Bissau	2012-2015	-2.62	-2.988	-2.251	<0.001
GNB: Guinea-Bissau	2015-2021	-2.218	-2.281	-2.155	<0.001
GNQ: Equatorial Guinea	2000-2003	-1.292	-1.476	-1.107	<0.001
GNQ: Equatorial Guinea	2003-2006	-2.863	-3.225	-2.499	<0.001
GNQ: Equatorial Guinea	2006-2009	-3.712	-4.072	-3.352	<0.001
GNQ: Equatorial Guinea	2009-2021	-2.035	-2.057	-2.014	<0.001
GRC: Greece	2000-2005	-8.779	-9.057	-8.501	<0.001
GRC: Greece	2005-2009	-4.711	-5.358	-4.058	<0.001
GRC: Greece	2009-2015	4.553	4.234	4.872	<0.001
GRC: Greece	2015-2018	-1.826	-3.156	-0.477	0.014
GRC: Greece	2018-2021	-3.921	-4.574	-3.263	<0.001
GRD: Grenada	2000-2002	1.377	0.5	2.262	0.007
GRD: Grenada	2002-2007	0.239	-0.036	0.515	0.08
GRD: Grenada	2007-2014	3.299	3.147	3.45	<0.001
GRD: Grenada	2014-2017	0.784	-0.088	1.663	0.072
GRD: Grenada	2017-2021	-0.604	-0.877	-0.33	0.001
GTM: Guatemala	2000-2003	-2.688	-2.786	-2.59	<0.001
GTM: Guatemala	2003-2009	-2.565	-2.609	-2.521	<0.001
GTM: Guatemala	2009-2017	-3.735	-3.761	-3.709	<0.001
GTM: Guatemala	2017-2021	-3.368	-3.429	-3.306	<0.001
GUY: Guyana	2000-2003	-1.883	-2.005	-1.762	<0.001
GUY: Guyana	2003-2011	-1.65	-1.682	-1.617	<0.001
GUY: Guyana	2011-2014	-2.412	-2.654	-2.17	<0.001
GUY: Guyana	2014-2021	-2.952	-2.984	-2.92	<0.001

HND: Honduras	2000-2004	-2.867	-2.983	-2.75	<0.001
HND: Honduras	2004-2008	-1.881	-2.067	-1.695	<0.001
HND: Honduras	2008-2012	-2.884	-3.068	-2.7	<0.001
HND: Honduras	2012-2021	-3.602	-3.635	-3.569	<0.001
HRV: Croatia	2000-2005	-5.656	-6.152	-5.157	<0.001
HRV: Croatia	2005-2013	-3.348	-3.652	-3.043	<0.001
HRV: Croatia	2013-2021	-1.656	-1.909	-1.403	<0.001
HTI: Haiti	2000-2010	-0.243	-0.333	-0.153	<0.001
HTI: Haiti	2010-2016	-1.271	-1.526	-1.015	<0.001
HTI: Haiti	2016-2021	-1.914	-2.168	-1.661	<0.001
HUN: Hungary	2000-2006	-6.419	-6.662	-6.175	<0.001
HUN: Hungary	2006-2013	-3.463	-3.714	-3.211	<0.001
HUN: Hungary	2013-2018	-5.937	-6.394	-5.477	<0.001
HUN: Hungary	2018-2021	-2.663	-3.41	-1.91	<0.001
IDN: Indonesia	2000-2003	-2.945	-3.172	-2.718	<0.001
IDN: Indonesia	2003-2008	-2.082	-2.227	-1.937	<0.001
IDN: Indonesia	2008-2011	-3.591	-4.041	-3.139	<0.001
IDN: Indonesia	2011-2017	-4.1	-4.2	-4	<0.001
IDN: Indonesia	2017-2021	-3.525	-3.668	-3.382	<0.001
IND: India	2000-2009	-3.289	-3.318	-3.26	<0.001
IND: India	2009-2012	-3.812	-4.125	-3.498	<0.001
IND: India	2012-2015	-4.075	-4.388	-3.762	<0.001
IND: India	2015-2018	-4.326	-4.637	-4.013	<0.001
IND: India	2018-2021	-5.658	-5.812	-5.504	<0.001
IRL: Ireland	2000-2002	-4.003	-5.016	-2.979	<0.001
IRL: Ireland	2002-2008	-6.706	-6.927	-6.485	<0.001
IRL: Ireland	2008-2013	0.539	0.202	0.877	0.005
IRL: Ireland	2013-2021	-2.477	-2.589	-2.364	<0.001
IRN: Iran	2000-2003	-3.559	-3.712	-3.405	<0.001
IRN: Iran	2003-2006	-4.04	-4.344	-3.734	<0.001
IRN: Iran	2006-2012	-4.911	-4.979	-4.844	<0.001
IRN: Iran	2012-2016	-3.978	-4.13	-3.825	<0.001
IRN: Iran	2016-2021	-3.586	-3.654	-3.517	<0.001
IRQ: Iraq	2000-2004	-1.322	-1.379	-1.265	<0.001
IRQ: Iraq	2004-2008	-1.859	-1.948	-1.77	<0.001
IRQ: Iraq	2008-2013	-2.953	-3.009	-2.898	<0.001
IRQ: Iraq	2013-2021	-3.141	-3.161	-3.122	<0.001
ISL: Iceland	2000-2005	-5.72	-5.853	-5.586	<0.001
ISL: Iceland	2005-2009	-3.522	-3.827	-3.216	<0.001
ISL: Iceland	2009-2012	-1.123	-1.747	-0.495	0.003
ISL: Iceland	2012-2019	0.759	0.651	0.867	<0.001
ISL: Iceland	2019-2021	-1.094	-1.718	-0.466	0.004
ISR: Israel	2000-2002	-3.804	-4.445	-3.158	<0.001
ISR: Israel	2002-2007	-5.851	-6.05	-5.652	<0.001
ISR: Israel	2007-2010	-0.833	-1.494	-0.167	0.02
ISR: Israel	2010-2016	-2.638	-2.783	-2.492	<0.001
ISR: Israel	2016-2021	-3.246	-3.391	-3.102	<0.001

ITA: Italy	2000-2003	-5.491	-5.763	-5.219	<0.001
ITA: Italy	2003-2007	-3.491	-3.768	-3.213	<0.001
ITA: Italy	2007-2011	-1.677	-1.959	-1.394	<0.001
ITA: Italy	2011-2017	-2.587	-2.712	-2.461	<0.001
ITA: Italy	2017-2021	-7.589	-7.757	-7.421	<0.001
JAM: Jamaica	2000-2002	-0.809	-1.139	-0.478	<0.001
JAM: Jamaica	2002-2007	-1.523	-1.627	-1.419	<0.001
JAM: Jamaica	2007-2010	-2.219	-2.545	-1.893	<0.001
JAM: Jamaica	2010-2016	-2.988	-3.06	-2.915	<0.001
JAM: Jamaica	2016-2021	-3.287	-3.359	-3.215	<0.001
JOR: Jordan	2000-2002	-2.525	-2.712	-2.338	<0.001
JOR: Jordan	2002-2008	-3.223	-3.265	-3.182	<0.001
JOR: Jordan	2008-2016	-2.776	-2.801	-2.751	<0.001
JOR: Jordan	2016-2021	-3.117	-3.159	-3.076	<0.001
JPN: Japan	2000-2002	-2.822	-3.678	-1.958	<0.001
JPN: Japan	2002-2011	-5.098	-5.19	-5.006	<0.001
JPN: Japan	2011-2014	-3.239	-4.091	-2.379	<0.001
JPN: Japan	2014-2021	-2.031	-2.147	-1.915	<0.001
KAZ: Kazakhstan	2000-2006	-4.088	-4.514	-3.661	<0.001
KAZ: Kazakhstan	2006-2009	-9.854	-12.193	-7.452	<0.001
KAZ: Kazakhstan	2009-2015	-14.415	-14.916	-13.91	<0.001
KAZ: Kazakhstan	2015-2018	-5.201	-7.661	-2.676	0.002
KAZ: Kazakhstan	2018-2021	2.95	1.605	4.312	0.001
KEN: Kenya	2000-2002	-0.884	-1.183	-0.584	<0.001
KEN: Kenya	2002-2005	-1.309	-1.607	-1.011	<0.001
KEN: Kenya	2005-2009	-2.075	-2.223	-1.928	<0.001
KEN: Kenya	2009-2019	-1.961	-1.988	-1.934	<0.001
KEN: Kenya	2019-2021	-2.601	-2.894	-2.306	<0.001
KGZ: Kyrgyzstan	2000-2007	-0.713	-0.954	-0.471	<0.001
KGZ: Kyrgyzstan	2007-2011	-3.856	-4.727	-2.977	<0.001
KGZ: Kyrgyzstan	2011-2015	-2.259	-3.144	-1.365	<0.001
KGZ: Kyrgyzstan	2015-2019	-4.384	-5.25	-3.51	<0.001
KGZ: Kyrgyzstan	2019-2021	-2.291	-4.053	-0.496	0.019
KHM: Cambodia	2000-2004	-5.39	-5.535	-5.245	<0.001
KHM: Cambodia	2004-2011	-4.581	-4.659	-4.503	<0.001
KHM: Cambodia	2011-2015	-5.287	-5.515	-5.057	<0.001
KHM: Cambodia	2015-2018	-4.325	-4.786	-3.861	<0.001
KHM: Cambodia	2018-2021	-3.641	-3.873	-3.407	<0.001
KIR: Kiribati	2000-2004	-1.747	-1.883	-1.612	<0.001
KIR: Kiribati	2004-2009	-0.382	-0.519	-0.244	<0.001
KIR: Kiribati	2009-2012	-0.848	-1.279	-0.414	0.002
KIR: Kiribati	2012-2017	-1.776	-1.912	-1.641	<0.001
KIR: Kiribati	2017-2021	-2.134	-2.269	-1.999	<0.001
KNA: Saint Kitts and Nevis	2000-2006	-4.406	-4.516	-4.296	<0.001
KNA: Saint Kitts and Nevis	2006-2009	-2.389	-3.052	-1.722	<0.001
KNA: Saint Kitts and Nevis	2009-2015	-0.621	-0.772	-0.469	<0.001
KNA: Saint Kitts and Nevis	2015-2018	-2.181	-2.845	-1.513	<0.001

KNA: Saint Kitts and Nevis	2018-2021	-2.811	-3.142	-2.48	<0.001
KOR: South Korea	2000-2003	-5.872	-6.244	-5.499	<0.001
KOR: South Korea	2003-2008	-8.027	-8.256	-7.796	<0.001
KOR: South Korea	2008-2011	-2.437	-3.206	-1.662	<0.001
KOR: South Korea	2011-2017	-1.448	-1.622	-1.273	<0.001
KOR: South Korea	2017-2021	-3.214	-3.456	-2.971	<0.001
KWT: Kuwait	2000-2004	-2.681	-2.88	-2.48	<0.001
KWT: Kuwait	2004-2009	-0.987	-1.19	-0.783	<0.001
KWT: Kuwait	2009-2014	-2.526	-2.727	-2.326	<0.001
KWT: Kuwait	2014-2021	-0.2	-0.286	-0.113	<0.001
LAO: Laos	2000-2003	-2.433	-2.531	-2.335	<0.001
LAO: Laos	2003-2006	-2.807	-3.003	-2.611	<0.001
LAO: Laos	2006-2016	-3.091	-3.108	-3.073	<0.001
LAO: Laos	2016-2021	-2.503	-2.547	-2.459	<0.001
LBN: Lebanon	2000-2007	-6.131	-6.22	-6.042	<0.001
LBN: Lebanon	2007-2011	-4.828	-5.167	-4.488	<0.001
LBN: Lebanon	2011-2014	-3.245	-3.932	-2.553	<0.001
LBN: Lebanon	2014-2021	-2.267	-2.36	-2.174	<0.001
LBR: Liberia	2000-2006	-4.394	-4.475	-4.314	<0.001
LBR: Liberia	2006-2010	-2.849	-3.091	-2.606	<0.001
LBR: Liberia	2010-2017	-0.326	-0.41	-0.242	<0.001
LBR: Liberia	2017-2021	-1.572	-1.728	-1.417	<0.001
LBY: Libya	2000-2004	-3.284	-3.527	-3.04	<0.001
LBY: Libya	2004-2010	-6.453	-6.62	-6.287	<0.001
LBY: Libya	2010-2014	-4.589	-4.967	-4.208	<0.001
LBY: Libya	2014-2021	-3.514	-3.617	-3.412	<0.001
LCA: Saint Lucia	2000-2004	2.169	2.031	2.307	<0.001
LCA: Saint Lucia	2004-2012	0.333	0.276	0.39	<0.001
LCA: Saint Lucia	2012-2017	-1.201	-1.335	-1.068	<0.001
LCA: Saint Lucia	2017-2021	1.636	1.498	1.773	<0.001
LKA: Sri Lanka	2000-2005	-6.704	-7.115	-6.291	<0.001
LKA: Sri Lanka	2005-2009	-1.181	-2.152	-0.2	0.024
LKA: Sri Lanka	2009-2013	-2.809	-3.765	-1.845	<0.001
LKA: Sri Lanka	2013-2017	-6.486	-7.405	-5.557	<0.001
LKA: Sri Lanka	2017-2021	-3.815	-4.414	-3.212	<0.001
LSO: Lesotho	2000-2004	0.263	0.144	0.381	0.001
LSO: Lesotho	2004-2009	1.076	0.957	1.195	<0.001
LSO: Lesotho	2009-2013	0.229	0.042	0.416	0.022
LSO: Lesotho	2013-2017	-0.441	-0.627	-0.255	0.001
LSO: Lesotho	2017-2021	-1.483	-1.6	-1.367	<0.001
LTU: Lithuania	2000-2005	-0.797	-1.45	-0.14	0.023
LTU: Lithuania	2005-2011	-7.762	-8.37	-7.151	<0.001
LTU: Lithuania	2011-2014	-3.75	-6.551	-0.864	0.018
LTU: Lithuania	2014-2018	-2.079	-3.514	-0.622	0.011
LTU: Lithuania	2018-2021	-4.024	-5.432	-2.596	<0.001
LUX: Luxembourg	2000-2005	-5.659	-5.817	-5.501	<0.001
LUX: Luxembourg	2005-2009	-2.322	-2.687	-1.955	<0.001

LUX: Luxembourg	2009-2013	0.045	-0.33	0.42	0.79
LUX: Luxembourg	2013-2018	1.772	1.531	2.014	<0.001
LUX: Luxembourg	2018-2021	-0.594	-0.965	-0.22	0.006
LVA: Latvia	2000-2003	-6.484	-7.124	-5.839	<0.001
LVA: Latvia	2003-2009	-3.837	-4.132	-3.541	<0.001
LVA: Latvia	2009-2015	-8.61	-8.891	-8.329	<0.001
LVA: Latvia	2015-2021	-4.486	-4.708	-4.264	<0.001
MAR: Morocco	2000-2004	-2.716	-2.797	-2.635	<0.001
MAR: Morocco	2004-2007	-3.532	-3.785	-3.279	<0.001
MAR: Morocco	2007-2016	-5.44	-5.467	-5.413	<0.001
MAR: Morocco	2016-2019	-4.533	-4.783	-4.282	<0.001
MAR: Morocco	2019-2021	-3.857	-4.109	-3.604	<0.001
MCO: Monaco	2000-2002	-3.064	-3.414	-2.714	<0.001
MCO: Monaco	2002-2005	-2.585	-2.936	-2.232	<0.001
MCO: Monaco	2005-2012	-2.3	-2.36	-2.24	<0.001
MCO: Monaco	2012-2015	-2.763	-3.114	-2.412	<0.001
MCO: Monaco	2015-2021	-3.307	-3.366	-3.248	<0.001
MDA: Moldova	2000-2004	-8.591	-8.817	-8.364	<0.001
MDA: Moldova	2004-2007	-3.318	-4.074	-2.557	<0.001
MDA: Moldova	2007-2011	-0.686	-1.075	-0.296	0.003
MDA: Moldova	2011-2021	-1.754	-1.814	-1.694	<0.001
MDG: Madagascar	2000-2006	-2.293	-2.36	-2.225	<0.001
MDG: Madagascar	2006-2010	-1.86	-2.06	-1.659	<0.001
MDG: Madagascar	2010-2013	-1.005	-1.409	-0.6	<0.001
MDG: Madagascar	2013-2016	-0.427	-0.833	-0.02	0.042
MDG: Madagascar	2016-2021	0.094	0.003	0.186	0.044
MDV: Maldives	2000-2005	-11.972	-12.223	-11.721	<0.001
MDV: Maldives	2005-2009	-8.832	-9.41	-8.25	<0.001
MDV: Maldives	2009-2013	-7.277	-7.865	-6.684	<0.001
MDV: Maldives	2013-2017	-2.709	-3.327	-2.088	<0.001
MDV: Maldives	2017-2021	-7.036	-7.41	-6.661	<0.001
MEX: Mexico	2000-2005	-7.624	-8.088	-7.158	<0.001
MEX: Mexico	2005-2015	-1.596	-1.798	-1.394	<0.001
MEX: Mexico	2015-2018	1.929	-0.34	4.249	0.089
MEX: Mexico	2018-2021	-2.283	-3.377	-1.177	0.001
MHL: Marshall Islands	2000-2002	0.017	-0.299	0.334	0.904
MHL: Marshall Islands	2002-2011	-0.669	-0.703	-0.635	<0.001
MHL: Marshall Islands	2011-2014	-1.418	-1.729	-1.106	<0.001
MHL: Marshall Islands	2014-2017	-2.706	-3.013	-2.398	<0.001
MHL: Marshall Islands	2017-2021	-3.132	-3.229	-3.036	<0.001
MKD: North Macedonia	2000-2005	-0.294	-1.974	1.415	0.71
MKD: North Macedonia	2005-2011	-4.72	-6.326	-3.087	<0.001
MKD: North Macedonia	2011-2016	3.817	1.352	6.342	0.006
MKD: North Macedonia	2016-2021	-16.555	-17.961	-15.125	<0.001
MLI: Mali	2000-2004	-3.159	-3.25	-3.068	<0.001
MLI: Mali	2004-2011	-1.917	-1.967	-1.868	<0.001
MLI: Mali	2011-2019	-1.482	-1.521	-1.443	<0.001

MLI: Mali	2019-2021	-1.836	-2.127	-1.544	<0.001
MLT: Malta	2000-2002	-3.204	-3.574	-2.832	<0.001
MLT: Malta	2002-2005	-1.466	-1.843	-1.088	<0.001
MLT: Malta	2005-2012	0.022	-0.043	0.087	0.458
MLT: Malta	2012-2016	-1.131	-1.32	-0.941	<0.001
MLT: Malta	2016-2021	-2.213	-2.297	-2.129	<0.001
MMR: Myanmar	2000-2010	-2.704	-2.79	-2.617	<0.001
MMR: Myanmar	2010-2021	-2.463	-2.539	-2.388	<0.001
MNE: Montenegro	2000-2003	-1.903	-2.671	-1.129	<0.001
MNE: Montenegro	2003-2009	-7.432	-7.757	-7.106	<0.001
MNE: Montenegro	2009-2013	-11.56	-12.252	-10.863	<0.001
MNE: Montenegro	2013-2019	-14.781	-15.08	-14.481	<0.001
MNE: Montenegro	2019-2021	-10.13	-11.531	-8.707	<0.001
MNG: Mongolia	2000-2006	-5.787	-5.913	-5.661	<0.001
MNG: Mongolia	2006-2010	-8.95	-9.309	-8.589	<0.001
MNG: Mongolia	2010-2013	-4.311	-5.065	-3.552	<0.001
MNG: Mongolia	2013-2016	-1.814	-2.588	-1.035	0.001
MNG: Mongolia	2016-2021	-4.276	-4.445	-4.107	<0.001
MOZ: Mozambique	2000-2002	-4.347	-4.7	-3.994	<0.001
MOZ: Mozambique	2002-2006	-3.541	-3.719	-3.363	<0.001
MOZ: Mozambique	2006-2009	-2.618	-2.976	-2.258	<0.001
MOZ: Mozambique	2009-2015	-1.744	-1.825	-1.663	<0.001
MOZ: Mozambique	2015-2021	-2.102	-2.163	-2.04	<0.001
MRT: Mauritania	2000-2005	-3.691	-3.73	-3.653	<0.001
MRT: Mauritania	2005-2008	-3.062	-3.235	-2.888	<0.001
MRT: Mauritania	2008-2016	-1.843	-1.866	-1.819	<0.001
MRT: Mauritania	2016-2021	-2.278	-2.318	-2.239	<0.001
MSR: Montserrat	2000-2002	-4.037	-4.516	-3.555	<0.001
MSR: Montserrat	2002-2010	-3.486	-3.551	-3.422	<0.001
MSR: Montserrat	2010-2021	-3.339	-3.371	-3.306	<0.001
MUS: Mauritius	2000-2002	-8.432	-10.78	-6.022	<0.001
MUS: Mauritius	2002-2006	-0.692	-1.973	0.607	0.253
MUS: Mauritius	2006-2009	-2.122	-4.632	0.454	0.093
MUS: Mauritius	2009-2016	-0.566	-1.002	-0.129	0.018
MUS: Mauritius	2016-2021	3.142	2.545	3.743	<0.001
MWI: Malawi	2000-2005	-5.48	-5.568	-5.393	<0.001
MWI: Malawi	2005-2010	-1.855	-1.984	-1.726	<0.001
MWI: Malawi	2010-2015	-3.391	-3.518	-3.264	<0.001
MWI: Malawi	2015-2021	-2.428	-2.496	-2.359	<0.001
MYS: Malaysia	2000-2002	-6.033	-7.545	-4.497	<0.001
MYS: Malaysia	2002-2005	-1.987	-3.564	-0.385	0.021
MYS: Malaysia	2005-2009	0.476	-0.336	1.294	0.214
MYS: Malaysia	2009-2013	-0.989	-1.789	-0.183	0.022
MYS: Malaysia	2013-2021	0.304	0.127	0.482	0.004
NAM: Namibia	2000-2004	-2.501	-2.624	-2.378	<0.001
NAM: Namibia	2004-2007	-0.907	-1.301	-0.51	0.001
NAM: Namibia	2007-2013	0.847	0.757	0.937	<0.001

NAM: Namibia	2013-2016	-0.676	-1.071	-0.278	0.004
NAM: Namibia	2016-2021	-1.804	-1.891	-1.716	<0.001
NER: Niger	2000-2005	-3.113	-3.197	-3.029	<0.001
NER: Niger	2005-2009	-2.101	-2.291	-1.91	<0.001
NER: Niger	2009-2012	-0.879	-1.264	-0.493	0.001
NER: Niger	2012-2019	0.135	0.069	0.201	0.001
NER: Niger	2019-2021	-0.383	-0.77	0.005	0.053
NGA: Nigeria	2000-2006	-2.486	-2.542	-2.43	<0.001
NGA: Nigeria	2006-2010	-1.237	-1.403	-1.07	<0.001
NGA: Nigeria	2010-2017	-0.442	-0.499	-0.386	<0.001
NGA: Nigeria	2017-2021	-1.279	-1.384	-1.173	<0.001
NIC: Nicaragua	2000-2004	-3.104	-3.371	-2.836	<0.001
NIC: Nicaragua	2004-2012	-1.583	-1.698	-1.468	<0.001
NIC: Nicaragua	2012-2015	-3.679	-4.515	-2.835	<0.001
NIC: Nicaragua	2015-2021	-7.461	-7.597	-7.324	<0.001
NIU: Niue	2000-2004	2.463	2.263	2.664	<0.001
NIU: Niue	2004-2007	-0.147	-0.763	0.472	0.611
NIU: Niue	2007-2011	-2.433	-2.735	-2.131	<0.001
NIU: Niue	2011-2021	-3.107	-3.154	-3.061	<0.001
NLD: Netherlands	2000-2004	-1.773	-1.997	-1.549	<0.001
NLD: Netherlands	2004-2010	-4.204	-4.358	-4.049	<0.001
NLD: Netherlands	2010-2016	-0.974	-1.134	-0.815	<0.001
NLD: Netherlands	2016-2021	0.549	0.387	0.711	<0.001
NOR: Norway	2000-2002	-3.034	-3.272	-2.795	<0.001
NOR: Norway	2002-2007	-3.473	-3.548	-3.398	<0.001
NOR: Norway	2007-2010	-4.036	-4.271	-3.799	<0.001
NOR: Norway	2010-2016	-3.081	-3.135	-3.028	<0.001
NOR: Norway	2016-2021	-3.632	-3.685	-3.579	<0.001
NPL: Nepal	2000-2010	-3.879	-3.893	-3.864	<0.001
NPL: Nepal	2010-2014	-4.14	-4.233	-4.046	<0.001
NPL: Nepal	2014-2018	-4.909	-5.001	-4.816	<0.001
NPL: Nepal	2018-2021	-3.9	-3.993	-3.806	<0.001
NRU: Nauru	2000-2003	-1.077	-1.27	-0.884	<0.001
NRU: Nauru	2003-2008	-0.288	-0.411	-0.165	0.001
NRU: Nauru	2008-2011	-0.903	-1.289	-0.515	0.001
NRU: Nauru	2011-2015	-2.52	-2.71	-2.33	<0.001
NRU: Nauru	2015-2021	-3.095	-3.159	-3.031	<0.001
NZL: New Zealand	2000-2003	-1.06	-2.673	0.579	0.181
NZL: New Zealand	2003-2007	-4.169	-5.731	-2.581	<0.001
NZL: New Zealand	2007-2012	3.416	2.347	4.497	<0.001
NZL: New Zealand	2012-2021	-3.333	-3.622	-3.042	<0.001
OMN: Oman	2000-2002	-6.114	-6.608	-5.618	<0.001
OMN: Oman	2002-2005	-4.124	-4.628	-3.617	<0.001
OMN: Oman	2005-2008	-1.908	-2.423	-1.389	<0.001
OMN: Oman	2008-2018	-0.997	-1.045	-0.949	<0.001
OMN: Oman	2018-2021	-2.119	-2.376	-1.86	<0.001
PAK: Pakistan	2000-2004	-1.571	-1.614	-1.528	<0.001

PAK: Pakistan	2004-2010	-1.141	-1.172	-1.111	<0.001
PAK: Pakistan	2010-2013	-1.784	-1.92	-1.648	<0.001
PAK: Pakistan	2013-2017	-2.158	-2.226	-2.09	<0.001
PAK: Pakistan	2017-2021	-2.3	-2.343	-2.257	<0.001
PAN: Panama	2000-2007	-2.586	-2.619	-2.553	<0.001
PAN: Panama	2007-2014	-3.134	-3.176	-3.092	<0.001
PAN: Panama	2014-2021	-3.26	-3.293	-3.227	<0.001
PER: Peru	2000-2003	-6.491	-6.853	-6.128	<0.001
PER: Peru	2003-2009	-4.798	-4.963	-4.633	<0.001
PER: Peru	2009-2013	-3.595	-3.968	-3.22	<0.001
PER: Peru	2013-2021	-2.341	-2.423	-2.258	<0.001
PHL: Philippines	2000-2002	-0.49	-0.779	-0.201	0.005
PHL: Philippines	2002-2008	-1.182	-1.246	-1.118	<0.001
PHL: Philippines	2008-2016	-1.025	-1.063	-0.986	<0.001
PHL: Philippines	2016-2019	-2.454	-2.737	-2.17	<0.001
PHL: Philippines	2019-2021	-2.879	-3.161	-2.596	<0.001
PLW: Palau	2000-2003	-1.753	-1.902	-1.604	<0.001
PLW: Palau	2003-2006	-1.942	-2.239	-1.644	<0.001
PLW: Palau	2006-2010	-2.599	-2.747	-2.452	<0.001
PLW: Palau	2010-2015	-3.129	-3.222	-3.037	<0.001
PLW: Palau	2015-2021	-3.362	-3.412	-3.313	<0.001
PNG: Papua New Guinea	2000-2003	-1.821	-1.918	-1.725	<0.001
PNG: Papua New Guinea	2003-2008	-1.037	-1.099	-0.976	<0.001
PNG: Papua New Guinea	2008-2018	-1.819	-1.837	-1.802	<0.001
PNG: Papua New Guinea	2018-2021	-2.041	-2.137	-1.944	<0.001
POL: Poland	2000-2008	-4.359	-4.488	-4.23	<0.001
POL: Poland	2008-2013	-5.255	-5.625	-4.885	<0.001
POL: Poland	2013-2016	-2.437	-3.634	-1.224	0.002
POL: Poland	2016-2019	-1.217	-2.429	0.011	0.052
POL: Poland	2019-2021	0.351	-0.88	1.598	0.531
PRK: North Korea	2000-2002	-9.589	-11.262	-7.885	<0.001
PRK: North Korea	2002-2005	-7.823	-9.528	-6.085	<0.001
PRK: North Korea	2005-2009	-1.069	-1.988	-0.14	0.029
PRK: North Korea	2009-2015	-6.523	-6.913	-6.132	<0.001
PRK: North Korea	2015-2021	-4.879	-5.179	-4.579	<0.001
PRT: Portugal	2000-2007	-6.776	-7.156	-6.394	<0.001
PRT: Portugal	2007-2016	0.188	-0.146	0.523	0.247
PRT: Portugal	2016-2021	-4.16	-4.813	-3.502	<0.001
PRY: Paraguay	2000-2007	-2.444	-2.488	-2.4	<0.001
PRY: Paraguay	2007-2016	-3.112	-3.148	-3.076	<0.001
PRY: Paraguay	2016-2021	-3.342	-3.416	-3.269	<0.001
PSE: Palestinian Territory	2000-2004	-1.839	-1.909	-1.769	<0.001
PSE: Palestinian Territory	2004-2010	-2.985	-3.034	-2.936	<0.001
PSE: Palestinian Territory	2010-2014	-2.75	-2.86	-2.641	<0.001
PSE: Palestinian Territory	2014-2021	-3.344	-3.373	-3.315	<0.001
QAT: Qatar	2000-2005	-3.301	-3.612	-2.988	<0.001
QAT: Qatar	2005-2012	-3.806	-4.04	-3.571	<0.001

QAT: Qatar	2012-2019	-2.085	-2.323	-1.846	<0.001
QAT: Qatar	2019-2021	-4.674	-6.039	-3.288	<0.001
ROU: Romania	2000-2004	-2.832	-3.287	-2.376	<0.001
ROU: Romania	2004-2009	-9.126	-9.552	-8.699	<0.001
ROU: Romania	2009-2014	-3.808	-4.258	-3.356	<0.001
ROU: Romania	2014-2018	-8.382	-9.059	-7.7	<0.001
ROU: Romania	2018-2021	-0.966	-1.698	-0.229	0.017
RUS: Russian Federation	2000-2005	-6.773	-7.441	-6.101	<0.001
RUS: Russian Federation	2005-2009	-8.325	-9.786	-6.839	<0.001
RUS: Russian Federation	2009-2013	1.157	-0.455	2.796	0.137
RUS: Russian Federation	2013-2019	-9.247	-9.897	-8.592	<0.001
RUS: Russian Federation	2019-2021	-12.223	-14.999	-9.355	<0.001
RWA: Rwanda	2000-2004	-7.201	-7.344	-7.057	<0.001
RWA: Rwanda	2004-2007	-6.311	-6.769	-5.852	<0.001
RWA: Rwanda	2007-2010	-4.588	-5.054	-4.12	<0.001
RWA: Rwanda	2010-2013	-3.546	-4.017	-3.073	<0.001
RWA: Rwanda	2013-2021	-2.105	-2.157	-2.053	<0.001
SAU: Saudi Arabia	2000-2010	-5.792	-5.813	-5.771	<0.001
SAU: Saudi Arabia	2010-2018	-6.508	-6.544	-6.472	<0.001
SAU: Saudi Arabia	2018-2021	-4.442	-4.58	-4.303	<0.001
SDN: Sudan	2000-2005	-1.727	-1.821	-1.633	<0.001
SDN: Sudan	2005-2011	-1.094	-1.189	-0.999	<0.001
SDN: Sudan	2011-2014	-1.384	-1.806	-0.96	<0.001
SDN: Sudan	2014-2021	-1.9	-1.956	-1.843	<0.001
SEN: Senegal	2000-2002	-2.807	-3.028	-2.585	<0.001
SEN: Senegal	2002-2008	-3.783	-3.832	-3.733	<0.001
SEN: Senegal	2008-2011	-2.792	-3.013	-2.57	<0.001
SEN: Senegal	2011-2017	-1.754	-1.804	-1.704	<0.001
SEN: Senegal	2017-2021	-2.783	-2.853	-2.713	<0.001
SGP: Singapore	2000-2003	-6.399	-7.019	-5.774	<0.001
SGP: Singapore	2003-2012	-2.054	-2.196	-1.912	<0.001
SGP: Singapore	2012-2016	0.044	-0.619	0.712	0.882
SGP: Singapore	2016-2019	-6.384	-7.621	-5.13	<0.001
SGP: Singapore	2019-2021	-8.813	-10.018	-7.592	<0.001
SLB: Solomon Islands	2000-2005	-1.072	-1.136	-1.008	<0.001
SLB: Solomon Islands	2005-2008	-2.126	-2.41	-1.842	<0.001
SLB: Solomon Islands	2008-2013	-2.933	-3.022	-2.844	<0.001
SLB: Solomon Islands	2013-2021	-3.157	-3.188	-3.127	<0.001
SLE: Sierra Leone	2000-2005	-1.335	-1.381	-1.288	<0.001
SLE: Sierra Leone	2005-2008	-2.491	-2.696	-2.285	<0.001
SLE: Sierra Leone	2008-2012	-2.832	-2.934	-2.729	<0.001
SLE: Sierra Leone	2012-2016	-2.221	-2.324	-2.118	<0.001
SLE: Sierra Leone	2016-2021	-2.017	-2.063	-1.971	<0.001
SLV: El Salvador	2000-2002	-5.222	-5.742	-4.699	<0.001
SLV: El Salvador	2002-2005	-4.884	-5.406	-4.359	<0.001
SLV: El Salvador	2005-2015	-4.128	-4.176	-4.079	<0.001
SLV: El Salvador	2015-2018	-3.65	-4.179	-3.118	<0.001

SLV: El Salvador	2018-2021	-3.377	-3.642	-3.11	<0.001
SMR: San Marino	2000-2004	-8.21	-8.372	-8.047	<0.001
SMR: San Marino	2004-2007	-7.006	-7.526	-6.483	<0.001
SMR: San Marino	2007-2010	-5.779	-6.306	-5.25	<0.001
SMR: San Marino	2010-2017	-5.077	-5.166	-4.987	<0.001
SMR: San Marino	2017-2021	-3.844	-4.015	-3.674	<0.001
SOM: Somalia	2000-2004	0.747	0.614	0.879	<0.001
SOM: Somalia	2004-2008	-0.498	-0.705	-0.291	<0.001
SOM: Somalia	2008-2021	-1.746	-1.768	-1.725	<0.001
SRB: Serbia	2000-2002	-5.036	-5.662	-4.405	<0.001
SRB: Serbia	2002-2006	-6.938	-7.245	-6.629	<0.001
SRB: Serbia	2006-2012	-2.416	-2.56	-2.272	<0.001
SRB: Serbia	2012-2017	-3.925	-4.126	-3.724	<0.001
SRB: Serbia	2017-2021	-1.038	-1.245	-0.831	<0.001
SSD: South Sudan	2000-2005	-2.363	-2.437	-2.288	<0.001
SSD: South Sudan	2005-2012	-2.818	-2.874	-2.762	<0.001
SSD: South Sudan	2012-2016	-0.488	-0.657	-0.318	<0.001
SSD: South Sudan	2016-2021	-0.127	-0.203	-0.051	0.004
STP: Sao Tome and Principe	2000-2002	-2.256	-3.133	-1.371	<0.001
STP: Sao Tome and Principe	2002-2010	-3.246	-3.363	-3.13	<0.001
STP: Sao Tome and Principe	2010-2013	-6.641	-7.479	-5.796	<0.001
STP: Sao Tome and Principe	2013-2018	-7.679	-7.942	-7.415	<0.001
STP: Sao Tome and Principe	2018-2021	-5.338	-5.763	-4.91	<0.001
SUR: Suriname	2000-2004	0.392	0.275	0.51	<0.001
SUR: Suriname	2004-2007	-1.601	-1.964	-1.237	<0.001
SUR: Suriname	2007-2021	-3.054	-3.071	-3.038	<0.001
SVK: Slovakia	2000-2006	-5.49	-5.712	-5.267	<0.001
SVK: Slovakia	2006-2009	-2.264	-3.617	-0.893	0.005
SVK: Slovakia	2009-2012	-0.909	-2.281	0.481	0.169
SVK: Slovakia	2012-2015	-2.145	-3.5	-0.772	0.007
SVK: Slovakia	2015-2021	-1.482	-1.714	-1.25	<0.001
SVN: Slovenia	2000-2002	-4.831	-5.445	-4.212	<0.001
SVN: Slovenia	2002-2009	-5.824	-5.928	-5.721	<0.001
SVN: Slovenia	2009-2018	-3.578	-3.646	-3.51	<0.001
SVN: Slovenia	2018-2021	-2.877	-3.191	-2.562	<0.001
SWE: Sweden	2000-2002	-1.799	-2.57	-1.021	0.001
SWE: Sweden	2002-2007	-4.693	-4.93	-4.455	<0.001
SWE: Sweden	2007-2010	-3.017	-3.779	-2.249	<0.001
SWE: Sweden	2010-2015	-0.042	-0.291	0.208	0.709
SWE: Sweden	2015-2021	-2.632	-2.761	-2.502	<0.001
SWZ: Eswatini	2000-2002	0.41	-0.318	1.142	0.242
SWZ: Eswatini	2002-2007	-2.17	-2.394	-1.945	<0.001
SWZ: Eswatini	2007-2014	0.217	0.094	0.34	0.003
SWZ: Eswatini	2014-2021	-1.638	-1.733	-1.542	<0.001
SYC: Seychelles	2000-2007	-0.028	-0.086	0.03	0.32
SYC: Seychelles	2007-2018	0.334	0.3	0.367	<0.001
SYC: Seychelles	2018-2021	-1.541	-1.753	-1.328	<0.001

SYR: Syria	2000-2006	-2.388	-2.615	-2.16	<0.001
SYR: Syria	2006-2010	-1.014	-1.694	-0.33	0.009
SYR: Syria	2010-2014	2.099	1.398	2.805	<0.001
SYR: Syria	2014-2019	0.399	-0.038	0.837	0.068
SYR: Syria	2019-2021	-1.226	-2.578	0.145	0.073
TCA: Turks and Caicos Islands	2000-2004	-4.188	-4.251	-4.125	<0.001
TCA: Turks and Caicos Islands	2004-2007	-3.545	-3.745	-3.345	<0.001
TCA: Turks and Caicos Islands	2007-2011	-2.902	-3.003	-2.801	<0.001
TCA: Turks and Caicos Islands	2011-2018	-2.574	-2.608	-2.539	<0.001
TCA: Turks and Caicos Islands	2018-2021	-3.149	-3.249	-3.048	<0.001
TCD: Chad	2000-2003	-2.155	-2.278	-2.033	<0.001
TCD: Chad	2003-2006	-1.598	-1.844	-1.351	<0.001
TCD: Chad	2006-2012	-0.939	-0.995	-0.884	<0.001
TCD: Chad	2012-2016	-1.45	-1.573	-1.326	<0.001
TCD: Chad	2016-2021	-1.618	-1.673	-1.563	<0.001
TGO: Togo	2000-2003	-2.266	-2.336	-2.195	<0.001
TGO: Togo	2003-2010	-2.163	-2.187	-2.139	<0.001
TGO: Togo	2010-2017	-1.779	-1.803	-1.756	<0.001
TGO: Togo	2017-2021	-1.98	-2.024	-1.935	<0.001
THA: Thailand	2000-2002	-4.932	-5.142	-4.721	<0.001
THA: Thailand	2002-2008	-4.712	-4.759	-4.665	<0.001
THA: Thailand	2008-2021	-4.339	-4.35	-4.327	<0.001
TJK: Tajikistan	2000-2004	-4.393	-4.558	-4.228	<0.001
TJK: Tajikistan	2004-2009	-2.452	-2.621	-2.284	<0.001
TJK: Tajikistan	2009-2014	-4.369	-4.534	-4.203	<0.001
TJK: Tajikistan	2014-2021	-2.974	-3.045	-2.903	<0.001
TKM: Turkmenistan	2000-2002	-2.305	-2.85	-1.757	<0.001
TKM: Turkmenistan	2002-2008	-3.194	-3.315	-3.073	<0.001
TKM: Turkmenistan	2008-2012	-0.989	-1.265	-0.712	<0.001
TKM: Turkmenistan	2012-2018	1.129	1.002	1.255	<0.001
TKM: Turkmenistan	2018-2021	-0.98	-1.256	-0.702	<0.001
TLS: Timor-Leste	2000-2003	-4.401	-4.593	-4.208	<0.001
TLS: Timor-Leste	2003-2006	-3.834	-4.221	-3.446	<0.001
TLS: Timor-Leste	2006-2009	-2.647	-3.039	-2.254	<0.001
TLS: Timor-Leste	2009-2021	-1.915	-1.938	-1.891	<0.001
TON: Tonga	2000-2008	-2.81	-2.851	-2.768	<0.001
TON: Tonga	2008-2011	-1.882	-2.264	-1.499	<0.001
TON: Tonga	2011-2016	-0.247	-0.37	-0.124	0.002
TON: Tonga	2016-2019	-1.24	-1.624	-0.854	<0.001
TON: Tonga	2019-2021	-2.675	-3.054	-2.295	<0.001
TTO: Trinidad and Tobago	2000-2004	-1.249	-1.449	-1.049	<0.001
TTO: Trinidad and Tobago	2004-2008	-2.605	-2.916	-2.292	<0.001
TTO: Trinidad and Tobago	2008-2021	-3.096	-3.128	-3.063	<0.001
TUN: Tunisia	2000-2004	-5.316	-5.543	-5.087	<0.001
TUN: Tunisia	2004-2007	-3.859	-4.589	-3.124	<0.001
TUN: Tunisia	2007-2011	-1.893	-2.266	-1.519	<0.001
TUN: Tunisia	2011-2019	-0.13	-0.232	-0.028	0.018

TUN: Tunisia	2019-2021	-1.954	-2.698	-1.204	<0.001
TUR: Turkiye	2000-2002	-6.556	-6.861	-6.251	<0.001
TUR: Turkiye	2002-2011	-7.065	-7.098	-7.032	<0.001
TUR: Turkiye	2011-2015	-6.322	-6.474	-6.168	<0.001
TUR: Turkiye	2015-2021	-5.279	-5.331	-5.227	<0.001
TUV: Tuvalu	2000-2003	-0.983	-1.289	-0.676	<0.001
TUV: Tuvalu	2003-2006	-3.197	-3.795	-2.595	<0.001
TUV: Tuvalu	2006-2012	-6.688	-6.818	-6.559	<0.001
TUV: Tuvalu	2012-2016	-4.943	-5.237	-4.648	<0.001
TUV: Tuvalu	2016-2021	-3.636	-3.769	-3.502	<0.001
TZA: Tanzania	2000-2005	-3.418	-3.466	-3.371	<0.001
TZA: Tanzania	2005-2008	-2.502	-2.716	-2.288	<0.001
TZA: Tanzania	2008-2015	-2.143	-2.179	-2.107	<0.001
TZA: Tanzania	2015-2021	-1.997	-2.033	-1.96	<0.001
UGA: Uganda	2000-2004	-2.871	-2.949	-2.793	<0.001
UGA: Uganda	2004-2007	-2.346	-2.593	-2.097	<0.001
UGA: Uganda	2007-2012	-2.133	-2.211	-2.054	<0.001
UGA: Uganda	2012-2017	-2.915	-2.993	-2.837	<0.001
UGA: Uganda	2017-2021	-2.475	-2.554	-2.397	<0.001
UKR: Ukraine	2000-2004	-5.006	-5.189	-4.822	<0.001
UKR: Ukraine	2004-2008	-4.07	-4.363	-3.776	<0.001
UKR: Ukraine	2008-2013	-4.72	-4.904	-4.536	<0.001
UKR: Ukraine	2013-2017	-3.312	-3.607	-3.016	<0.001
UKR: Ukraine	2017-2021	-1.916	-2.105	-1.726	<0.001
URY: Uruguay	2000-2005	-2.786	-3.279	-2.292	<0.001
URY: Uruguay	2005-2009	-10.176	-11.189	-9.15	<0.001
URY: Uruguay	2009-2015	0.813	0.302	1.326	0.006
URY: Uruguay	2015-2018	-7.226	-9.308	-5.096	<0.001
URY: Uruguay	2018-2021	-0.948	-2.066	0.183	0.089
USA: United States	2000-2003	0.125	-0.234	0.485	0.447
USA: United States	2003-2010	-1.735	-1.854	-1.616	<0.001
USA: United States	2010-2013	-0.477	-1.189	0.24	0.163
USA: United States	2013-2017	-1.651	-2.004	-1.298	<0.001
USA: United States	2017-2021	-3.589	-3.807	-3.369	<0.001
UZB: Uzbekistan	2000-2004	-4.358	-4.691	-4.023	<0.001
UZB: Uzbekistan	2004-2007	-5.977	-7.01	-4.934	<0.001
UZB: Uzbekistan	2007-2012	-8.164	-8.484	-7.843	<0.001
UZB: Uzbekistan	2012-2016	-6.436	-6.951	-5.918	<0.001
UZB: Uzbekistan	2016-2021	-4.668	-4.903	-4.432	<0.001
VCT: Saint Vincent and the Grenadines	2000-2009	-0.379	-0.42	-0.337	<0.001
VCT: Saint Vincent and the Grenadines	2009-2012	-1.991	-2.44	-1.541	<0.001
VCT: Saint Vincent and the Grenadines	2012-2017	-4.092	-4.231	-3.953	<0.001
VCT: Saint Vincent and the Grenadines	2017-2021	-3.451	-3.591	-3.311	<0.001
VEN: Venezuela	2000-2013	0.468	-0.044	0.982	0.07

VEN: Venezuela	2013-2016	12.069	1.661	23.543	0.025
VEN: Venezuela	2016-2021	-0.429	-2.576	1.765	0.678
VGB: British Virgin Islands	2000-2007	-0.504	-0.539	-0.468	<0.001
VGB: British Virgin Islands	2007-2010	-1.908	-2.17	-1.646	<0.001
VGB: British Virgin Islands	2010-2015	-2.778	-2.86	-2.696	<0.001
VGB: British Virgin Islands	2015-2021	-3.363	-3.407	-3.32	<0.001
VNM: Vietnam	2000-2002	-4.475	-4.814	-4.134	<0.001
VNM: Vietnam	2002-2005	-3.059	-3.404	-2.713	<0.001
VNM: Vietnam	2005-2008	-1.487	-1.837	-1.135	<0.001
VNM: Vietnam	2008-2015	-0.888	-0.948	-0.829	<0.001
VNM: Vietnam	2015-2021	-1.044	-1.104	-0.985	<0.001
VUT: Vanuatu	2000-2002	-1.088	-1.319	-0.857	<0.001
VUT: Vanuatu	2002-2009	0.399	0.359	0.438	<0.001
VUT: Vanuatu	2009-2013	-0.195	-0.311	-0.078	0.005
VUT: Vanuatu	2013-2017	-2.182	-2.297	-2.068	<0.001
VUT: Vanuatu	2017-2021	-2.995	-3.067	-2.924	<0.001
WSM: Samoa	2000-2004	-2.948	-3.398	-2.496	<0.001
WSM: Samoa	2004-2011	0.086	-0.163	0.335	0.464
WSM: Samoa	2011-2018	-1.401	-1.646	-1.156	<0.001
WSM: Samoa	2018-2021	-2.423	-3.138	-1.703	<0.001
XKX: Kosovo	2000-2003	-5.506	-5.619	-5.393	<0.001
XKX: Kosovo	2003-2007	-7.585	-7.696	-7.475	<0.001
XKX: Kosovo	2007-2012	-6.409	-6.48	-6.338	<0.001
XKX: Kosovo	2012-2015	-6.183	-6.408	-5.958	<0.001
XKX: Kosovo	2015-2021	-4.891	-4.93	-4.853	<0.001
YEM: Yemen	2000-2009	-3.014	-3.055	-2.974	<0.001
YEM: Yemen	2009-2021	-0.006	-0.033	0.02	0.623
ZAF: South Africa	2000-2013	-3.545	-3.59	-3.5	<0.001
ZAF: South Africa	2013-2019	0.251	0.052	0.451	0.017
ZAF: South Africa	2019-2021	-0.578	-1.458	0.309	0.183
ZMB: Zambia	2000-2006	-3.895	-4.053	-3.736	<0.001
ZMB: Zambia	2006-2009	-1.088	-2.048	-0.119	0.031
ZMB: Zambia	2009-2018	-0.529	-0.634	-0.423	<0.001
ZMB: Zambia	2018-2021	-1.567	-2.045	-1.086	<0.001
ZWE: Zimbabwe	2000-2003	4.229	3.773	4.686	<0.001
ZWE: Zimbabwe	2003-2006	2.171	1.28	3.07	<0.001
ZWE: Zimbabwe	2006-2009	-0.132	-1.003	0.747	0.738
ZWE: Zimbabwe	2009-2013	-3.181	-3.604	-2.756	<0.001
ZWE: Zimbabwe	2013-2021	-1.847	-1.941	-1.753	<0.001

Table S4. Results from the joinpoint regression analysis of infant mortality rate

Country	Segment	APC	APC 95% LCL	APC 95% UCL	p-value
AFG: Afghanistan	2000-2003	-2.943	-3.017	-2.869	<0.001
AFG: Afghanistan	2003-2006	-3.357	-3.504	-3.21	<0.001
AFG: Afghanistan	2006-2016	-3.752	-3.765	-3.738	<0.001
AFG: Afghanistan	2016-2021	-3.188	-3.221	-3.155	<0.001
AGO: Angola	2000-2003	-3.172	-3.294	-3.05	<0.001
AGO: Angola	2003-2006	-4.64	-4.881	-4.399	<0.001
AGO: Angola	2006-2013	-5.782	-5.822	-5.742	<0.001
AGO: Angola	2013-2016	-4.282	-4.524	-4.04	<0.001
AGO: Angola	2016-2021	-3.232	-3.286	-3.177	<0.001
AIA: Anguilla	2000-2012	-4.072	-4.09	-4.054	<0.001
AIA: Anguilla	2012-2021	-3.295	-3.323	-3.267	<0.001
ALB: Albania	2000-2005	-5.766	-6.013	-5.519	<0.001
ALB: Albania	2005-2013	-8.037	-8.181	-7.893	<0.001
ALB: Albania	2013-2016	-3.519	-4.642	-2.382	<0.001
ALB: Albania	2016-2021	0.3	0.038	0.563	0.029
AND: Andorra	2000-2005	-3.903	-4.029	-3.778	<0.001
AND: Andorra	2005-2018	-4.578	-4.611	-4.546	<0.001
AND: Andorra	2018-2021	-3.184	-3.466	-2.901	<0.001
ARE: United Arab Emirates	2000-2004	-2.572	-2.62	-2.525	<0.001
ARE: United Arab Emirates	2004-2008	-2.304	-2.379	-2.228	<0.001
ARE: United Arab Emirates	2008-2012	-2.895	-2.97	-2.82	<0.001
ARE: United Arab Emirates	2012-2016	-2.363	-2.439	-2.288	<0.001
ARE: United Arab Emirates	2016-2021	-2.997	-3.031	-2.963	<0.001
ARG: Argentina	2000-2005	-3.356	-3.425	-3.287	<0.001
ARG: Argentina	2005-2010	-2.566	-2.663	-2.468	<0.001
ARG: Argentina	2010-2015	-4.371	-4.467	-4.275	<0.001
ARG: Argentina	2015-2018	-6.602	-6.898	-6.304	<0.001
ARG: Argentina	2018-2021	-10.11	-10.253	-9.967	<0.001
ARM: Armenia	2000-2002	-4.599	-4.669	-4.528	<0.001
ARM: Armenia	2002-2005	-4.74	-4.81	-4.67	<0.001
ARM: Armenia	2005-2009	-4.834	-4.869	-4.799	<0.001
ARM: Armenia	2009-2014	-4.946	-4.968	-4.924	<0.001
ARM: Armenia	2014-2021	-4.859	-4.869	-4.85	<0.001
ATG: Antigua and Barbuda	2000-2002	-2.548	-3.008	-2.086	<0.001
ATG: Antigua and Barbuda	2002-2006	-3.955	-4.182	-3.728	<0.001
ATG: Antigua and Barbuda	2006-2014	-4.956	-5.016	-4.896	<0.001
ATG: Antigua and Barbuda	2014-2017	-4.239	-4.691	-3.785	<0.001
ATG: Antigua and Barbuda	2017-2021	-3.545	-3.689	-3.401	<0.001
AUS: Australia	2000-2005	-1.331	-1.445	-1.217	<0.001
AUS: Australia	2005-2008	-2.806	-3.306	-2.303	<0.001
AUS: Australia	2008-2013	-4.636	-4.792	-4.481	<0.001
AUS: Australia	2013-2016	-2.514	-3.015	-2.009	<0.001
AUS: Australia	2016-2021	-0.235	-0.35	-0.12	0.002
AUT: Austria	2000-2010	-2.564	-2.71	-2.418	<0.001
AUT: Austria	2010-2015	-3.447	-4.034	-2.857	<0.001

AUT: Austria	2015-2018	-1.03	-2.919	0.897	0.262
AUT: Austria	2018-2021	0.914	-0.054	1.891	0.062
AZE: Azerbaijan	2000-2004	-6.318	-6.442	-6.194	<0.001
AZE: Azerbaijan	2004-2010	-5.945	-6.033	-5.857	<0.001
AZE: Azerbaijan	2010-2018	-6.443	-6.495	-6.391	<0.001
AZE: Azerbaijan	2018-2021	-4.63	-4.829	-4.43	<0.001
BDI: Burundi	2000-2004	-3.431	-3.615	-3.247	<0.001
BDI: Burundi	2004-2013	-5.305	-5.367	-5.243	<0.001
BDI: Burundi	2013-2016	-4.327	-4.902	-3.748	<0.001
BDI: Burundi	2016-2021	-3.273	-3.403	-3.142	<0.001
BEL: Belgium	2000-2005	-3.089	-3.217	-2.962	<0.001
BEL: Belgium	2005-2012	-2.357	-2.454	-2.26	<0.001
BEL: Belgium	2012-2015	-1.516	-2.093	-0.936	<0.001
BEL: Belgium	2015-2021	0.215	0.116	0.315	0.001
BEN: Benin	2000-2006	-2.057	-2.068	-2.047	<0.001
BEN: Benin	2006-2014	-1.855	-1.863	-1.846	<0.001
BEN: Benin	2014-2018	-2.03	-2.061	-1.999	<0.001
BEN: Benin	2018-2021	-2.491	-2.522	-2.46	<0.001
BFA: Burkina Faso	2000-2002	-1.764	-1.927	-1.601	<0.001
BFA: Burkina Faso	2002-2005	-2.694	-2.856	-2.532	<0.001
BFA: Burkina Faso	2005-2009	-3.401	-3.481	-3.32	<0.001
BFA: Burkina Faso	2009-2014	-2.748	-2.799	-2.697	<0.001
BFA: Burkina Faso	2014-2021	-2.362	-2.384	-2.34	<0.001
BGD: Bangladesh	2000-2002	-4.938	-5.115	-4.76	<0.001
BGD: Bangladesh	2002-2009	-4.698	-4.728	-4.668	<0.001
BGD: Bangladesh	2009-2013	-4.429	-4.518	-4.339	<0.001
BGD: Bangladesh	2013-2016	-4.542	-4.72	-4.364	<0.001
BGD: Bangladesh	2016-2021	-4.978	-5.017	-4.938	<0.001
BGR: Bulgaria	2000-2002	-3.64	-4.825	-2.44	<0.001
BGR: Bulgaria	2002-2007	-5.83	-6.198	-5.461	<0.001
BGR: Bulgaria	2007-2011	-2.833	-3.432	-2.23	<0.001
BGR: Bulgaria	2011-2017	-5.944	-6.204	-5.684	<0.001
BGR: Bulgaria	2017-2021	-3.572	-3.949	-3.194	<0.001
BHR: Bahrain	2000-2006	-3.167	-3.316	-3.017	<0.001
BHR: Bahrain	2006-2011	-4.701	-4.976	-4.426	<0.001
BHR: Bahrain	2011-2014	-2.416	-3.302	-1.522	<0.001
BHR: Bahrain	2014-2021	-1.169	-1.289	-1.048	<0.001
BHS: Bahamas	2000-2021	-1.022	-1.468	-0.573	<0.001
BIH: Bosnia and Herzegovina	2000-2006	-2.207	-2.479	-1.935	<0.001
BIH: Bosnia and Herzegovina	2006-2012	-4.421	-4.772	-4.069	<0.001
BIH: Bosnia and Herzegovina	2012-2021	-1.796	-1.943	-1.648	<0.001
BLR: Belarus	2000-2005	-7.512	-7.677	-7.347	<0.001
BLR: Belarus	2005-2010	-8.846	-9.075	-8.616	<0.001
BLR: Belarus	2010-2013	-6.471	-7.214	-5.723	<0.001
BLR: Belarus	2013-2016	-5.446	-6.197	-4.689	<0.001
BLR: Belarus	2016-2021	-6.918	-7.084	-6.752	<0.001
BLZ: Belize	2000-2002	-2.893	-3.782	-1.996	<0.001

BLZ: Belize	2002-2010	-1.799	-1.92	-1.678	<0.001
BLZ: Belize	2010-2013	-3.655	-4.537	-2.765	<0.001
BLZ: Belize	2013-2021	-5.151	-5.246	-5.055	<0.001
BOL: Bolivia	2000-2003	-4.96	-5.016	-4.905	<0.001
BOL: Bolivia	2003-2015	-5.124	-5.131	-5.116	<0.001
BOL: Bolivia	2015-2019	-4	-4.056	-3.944	<0.001
BOL: Bolivia	2019-2021	-3.482	-3.595	-3.369	<0.001
BRA: Brazil	2000-2010	-6.045	-6.416	-5.671	<0.001
BRA: Brazil	2010-2021	-2.14	-2.475	-1.803	<0.001
BRB: Barbados	2000-2002	1.695	1.376	2.016	<0.001
BRB: Barbados	2002-2005	0.51	0.194	0.827	0.006
BRB: Barbados	2005-2014	-0.888	-0.923	-0.854	<0.001
BRB: Barbados	2014-2017	-2.213	-2.52	-1.904	<0.001
BRB: Barbados	2017-2021	-2.944	-3.04	-2.847	<0.001
BRN: Brunei Darussalam	2000-2003	-2.025	-2.223	-1.826	<0.001
BRN: Brunei Darussalam	2003-2007	-0.205	-0.407	-0.002	0.048
BRN: Brunei Darussalam	2007-2011	1.25	1.045	1.455	<0.001
BRN: Brunei Darussalam	2011-2019	2.209	2.154	2.264	<0.001
BRN: Brunei Darussalam	2019-2021	0.396	-0.01	0.804	0.055
BTN: Bhutan	2000-2004	-4.806	-4.899	-4.713	<0.001
BTN: Bhutan	2004-2010	-5.283	-5.349	-5.218	<0.001
BTN: Bhutan	2010-2013	-4.525	-4.82	-4.229	<0.001
BTN: Bhutan	2013-2017	-3.728	-3.877	-3.579	<0.001
BTN: Bhutan	2017-2021	-3.134	-3.229	-3.039	<0.001
BWA: Botswana	2000-2009	4.481	3.716	5.251	<0.001
BWA: Botswana	2009-2021	-4.989	-5.44	-4.536	<0.001
CAF: Central African Republic	2000-2003	-1.336	-1.709	-0.962	<0.001
CAF: Central African Republic	2003-2011	-1.932	-2.032	-1.833	<0.001
CAF: Central African Republic	2011-2018	-1.5	-1.626	-1.374	<0.001
CAF: Central African Republic	2018-2021	-2.007	-2.377	-1.635	<0.001
CAN: Canada	2000-2005	-0.086	-0.129	-0.043	0.001
CAN: Canada	2005-2015	-1.112	-1.129	-1.094	<0.001
CAN: Canada	2015-2019	-0.839	-0.935	-0.744	<0.001
CAN: Canada	2019-2021	-1.205	-1.395	-1.015	<0.001
CHE: Switzerland	2000-2002	-1.481	-1.613	-1.349	<0.001
CHE: Switzerland	2002-2007	-2.051	-2.092	-2.009	<0.001
CHE: Switzerland	2007-2010	-1.575	-1.707	-1.444	<0.001
CHE: Switzerland	2010-2015	-0.877	-0.919	-0.836	<0.001
CHE: Switzerland	2015-2021	-1.656	-1.678	-1.634	<0.001
CHL: Chile	2000-2002	-5.155	-5.808	-4.498	<0.001
CHL: Chile	2002-2005	-2.422	-3.094	-1.746	<0.001
CHL: Chile	2005-2010	-0.729	-0.945	-0.512	<0.001
CHL: Chile	2010-2017	-1.792	-1.907	-1.678	<0.001
CHL: Chile	2017-2021	-3.653	-3.864	-3.443	<0.001
CHN: China	2000-2002	-7.399	-7.574	-7.224	<0.001
CHN: China	2002-2008	-8.84	-8.878	-8.801	<0.001
CHN: China	2008-2018	-8.024	-8.04	-8.008	<0.001

CHN: China	2018-2021	-7.609	-7.697	-7.522	<0.001
CIV: CÃ´te d'Ivoire	2000-2004	-2.079	-2.227	-1.931	<0.001
CIV: CÃ´te d'Ivoire	2004-2014	-2.642	-2.684	-2.599	<0.001
CIV: CÃ´te d'Ivoire	2014-2017	-2.386	-2.852	-1.918	<0.001
CIV: CÃ´te d'Ivoire	2017-2021	-2.819	-2.966	-2.672	<0.001
CMR: Cameroon	2000-2009	-2.321	-2.372	-2.269	<0.001
CMR: Cameroon	2009-2012	-3.379	-3.933	-2.822	<0.001
CMR: Cameroon	2012-2017	-3.686	-3.861	-3.511	<0.001
CMR: Cameroon	2017-2021	-3.036	-3.212	-2.86	<0.001
COD: Congo DRC	2000-2003	-2.108	-2.187	-2.029	<0.001
COD: Congo DRC	2003-2006	-2.432	-2.59	-2.274	<0.001
COD: Congo DRC	2006-2010	-2.585	-2.664	-2.506	<0.001
COD: Congo DRC	2010-2021	-2.662	-2.672	-2.651	<0.001
COG: Congo	2000-2002	-2.742	-6.899	1.6	0.194
COG: Congo	2002-2009	-5.988	-6.679	-5.291	<0.001
COG: Congo	2009-2021	-2.582	-2.833	-2.33	<0.001
COK: Cook Islands	2000-2004	-4.697	-4.818	-4.576	<0.001
COK: Cook Islands	2004-2009	-5.337	-5.457	-5.217	<0.001
COK: Cook Islands	2009-2012	-4.522	-4.905	-4.137	<0.001
COK: Cook Islands	2012-2017	-3.672	-3.794	-3.549	<0.001
COK: Cook Islands	2017-2021	-3.307	-3.43	-3.184	<0.001
COL: Colombia	2000-2007	-2.842	-2.869	-2.814	<0.001
COL: Colombia	2007-2011	-3.065	-3.168	-2.963	<0.001
COL: Colombia	2011-2021	-3.187	-3.203	-3.171	<0.001
COM: Comoros	2000-2003	-2.061	-2.112	-2.01	<0.001
COM: Comoros	2003-2008	-2.49	-2.522	-2.458	<0.001
COM: Comoros	2008-2014	-2.788	-2.81	-2.765	<0.001
COM: Comoros	2014-2018	-3.162	-3.212	-3.111	<0.001
COM: Comoros	2018-2021	-2.852	-2.903	-2.802	<0.001
CPV: Cabo Verde	2000-2003	-7.627	-8.196	-7.054	<0.001
CPV: Cabo Verde	2003-2010	-1.152	-1.359	-0.945	<0.001
CPV: Cabo Verde	2010-2013	-4.66	-5.832	-3.473	<0.001
CPV: Cabo Verde	2013-2018	-6.881	-7.245	-6.516	<0.001
CPV: Cabo Verde	2018-2021	-5.108	-5.693	-4.52	<0.001
CRI: Costa Rica	2000-2002	-4.126	-5.974	-2.241	0.001
CRI: Costa Rica	2002-2010	-1.206	-1.463	-0.949	<0.001
CRI: Costa Rica	2010-2014	-3.875	-4.806	-2.935	<0.001
CRI: Costa Rica	2014-2019	-1.748	-2.351	-1.142	<0.001
CRI: Costa Rica	2019-2021	-6.987	-8.78	-5.159	<0.001
CUB: Cuba	2000-2004	-3.525	-3.608	-3.442	<0.001
CUB: Cuba	2004-2008	-3.097	-3.229	-2.965	<0.001
CUB: Cuba	2008-2011	-2.662	-2.927	-2.396	<0.001
CUB: Cuba	2011-2014	-2.335	-2.601	-2.068	<0.001
CUB: Cuba	2014-2021	-1.995	-2.03	-1.959	<0.001
CYP: Cyprus	2000-2007	-7.006	-7.167	-6.844	<0.001
CYP: Cyprus	2007-2013	-5.16	-5.435	-4.883	<0.001
CYP: Cyprus	2013-2016	-3.113	-4.367	-1.843	<0.001

CYP: Cyprus	2016-2021	0.254	-0.038	0.546	0.082
CZE: Czech Republic	2000-2005	-4.581	-4.765	-4.397	<0.001
CZE: Czech Republic	2005-2009	-5.337	-5.744	-4.927	<0.001
CZE: Czech Republic	2009-2012	-3.611	-4.439	-2.775	<0.001
CZE: Czech Republic	2012-2018	-0.087	-0.28	0.106	0.329
CZE: Czech Republic	2018-2021	-4.532	-4.943	-4.119	<0.001
DEU: Germany	2000-2009	-2.335	-2.364	-2.305	<0.001
DEU: Germany	2009-2012	-1.723	-2.047	-1.397	<0.001
DEU: Germany	2012-2017	-0.569	-0.673	-0.465	<0.001
DEU: Germany	2017-2021	-1.809	-1.912	-1.707	<0.001
DJI: Djibouti	2000-2002	-2.021	-2.271	-1.769	<0.001
DJI: Djibouti	2002-2007	-2.39	-2.469	-2.311	<0.001
DJI: Djibouti	2007-2017	-2.657	-2.68	-2.635	<0.001
DJI: Djibouti	2017-2021	-2.967	-3.045	-2.888	<0.001
DMA: Dominica	2000-2003	3.208	2.988	3.428	<0.001
DMA: Dominica	2003-2007	4.346	4.124	4.569	<0.001
DMA: Dominica	2007-2015	5.297	5.237	5.357	<0.001
DMA: Dominica	2015-2018	3.648	3.207	4.091	<0.001
DMA: Dominica	2018-2021	1.352	1.136	1.568	<0.001
DNK: Denmark	2000-2003	-2.335	-2.569	-2.101	<0.001
DNK: Denmark	2003-2007	-3.728	-3.959	-3.497	<0.001
DNK: Denmark	2007-2010	-2.524	-2.99	-2.055	<0.001
DNK: Denmark	2010-2016	0.063	-0.045	0.17	0.215
DNK: Denmark	2016-2021	-2.247	-2.352	-2.142	<0.001
DOM: Dominican Republic	2000-2003	-1.938	-2.095	-1.78	<0.001
DOM: Dominican Republic	2003-2006	-1.121	-1.437	-0.803	<0.001
DOM: Dominican Republic	2006-2009	-0.632	-0.951	-0.313	0.002
DOM: Dominican Republic	2009-2018	-0.098	-0.133	-0.063	<0.001
DOM: Dominican Republic	2018-2021	-1.66	-1.818	-1.502	<0.001
DZA: Algeria	2000-2002	-3.785	-4.006	-3.565	<0.001
DZA: Algeria	2002-2008	-4.168	-4.217	-4.119	<0.001
DZA: Algeria	2008-2011	-3.208	-3.429	-2.986	<0.001
DZA: Algeria	2011-2017	-1.583	-1.633	-1.532	<0.001
DZA: Algeria	2017-2021	-2.271	-2.341	-2.2	<0.001
ECU: Ecuador	2000-2002	-4.794	-5.005	-4.583	<0.001
ECU: Ecuador	2002-2007	-3.773	-3.841	-3.706	<0.001
ECU: Ecuador	2007-2014	-4.206	-4.242	-4.17	<0.001
ECU: Ecuador	2014-2019	-3.344	-3.412	-3.276	<0.001
ECU: Ecuador	2019-2021	-3.616	-3.829	-3.402	<0.001
EGY: Egypt	2000-2002	-5.013	-5.235	-4.79	<0.001
EGY: Egypt	2002-2005	-4.314	-4.538	-4.09	<0.001
EGY: Egypt	2005-2014	-3.891	-3.915	-3.866	<0.001
EGY: Egypt	2014-2021	-3.343	-3.373	-3.313	<0.001
ERI: Eritrea	2000-2003	-4.067	-4.149	-3.986	<0.001
ERI: Eritrea	2003-2007	-3.547	-3.628	-3.465	<0.001
ERI: Eritrea	2007-2010	-3.09	-3.254	-2.926	<0.001
ERI: Eritrea	2010-2016	-2.741	-2.778	-2.704	<0.001

ERI: Eritrea	2016-2021	-2.658	-2.694	-2.621	<0.001
ESP: Spain	2000-2005	-2.447	-2.514	-2.381	<0.001
ESP: Spain	2005-2011	-3.895	-3.961	-3.83	<0.001
ESP: Spain	2011-2014	-2.808	-3.104	-2.511	<0.001
ESP: Spain	2014-2019	-0.918	-1.013	-0.822	<0.001
ESP: Spain	2019-2021	-1.811	-2.11	-1.511	<0.001
EST: Estonia	2000-2002	-8.122	-8.339	-7.904	<0.001
EST: Estonia	2002-2008	-8.585	-8.633	-8.536	<0.001
EST: Estonia	2008-2011	-8.353	-8.57	-8.136	<0.001
EST: Estonia	2011-2019	-7.421	-7.451	-7.392	<0.001
EST: Estonia	2019-2021	-6.738	-6.959	-6.516	<0.001
ETH: Ethiopia	2000-2003	-4.139	-4.245	-4.032	<0.001
ETH: Ethiopia	2003-2009	-4.871	-4.918	-4.824	<0.001
ETH: Ethiopia	2009-2015	-4.504	-4.552	-4.457	<0.001
ETH: Ethiopia	2015-2018	-4.215	-4.428	-4.003	<0.001
ETH: Ethiopia	2018-2021	-3.351	-3.458	-3.244	<0.001
FIN: Finland	2000-2005	-2.596	-2.67	-2.523	<0.001
FIN: Finland	2005-2008	-4.082	-4.403	-3.759	<0.001
FIN: Finland	2008-2012	-4.54	-4.7	-4.38	<0.001
FIN: Finland	2012-2015	-3.475	-3.799	-3.151	<0.001
FIN: Finland	2015-2021	-2.18	-2.235	-2.124	<0.001
FJI: Fiji	2000-2002	-0.201	-1.312	0.922	0.699
FJI: Fiji	2002-2007	1.088	0.731	1.446	<0.001
FJI: Fiji	2007-2013	-0.133	-0.383	0.117	0.266
FJI: Fiji	2013-2021	1.962	1.837	2.086	<0.001
FRA: France	2000-2005	-4.102	-4.184	-4.02	<0.001
FRA: France	2005-2010	-1.25	-1.369	-1.131	<0.001
FRA: France	2010-2013	-0.373	-0.754	0.008	0.054
FRA: France	2013-2019	1.794	1.707	1.881	<0.001
FRA: France	2019-2021	0.491	0.107	0.875	0.018
FSM: Micronesia	2000-2011	-0.942	-1.185	-0.698	<0.001
FSM: Micronesia	2011-2021	-2.873	-3.149	-2.597	<0.001
GAB: Gabon	2000-2006	-2.298	-2.547	-2.048	<0.001
GAB: Gabon	2006-2021	-3.094	-3.155	-3.032	<0.001
GBR: United Kingdom	2000-2007	-1.698	-1.817	-1.579	<0.001
GBR: United Kingdom	2007-2013	-3.614	-3.809	-3.419	<0.001
GBR: United Kingdom	2013-2021	-0.993	-1.091	-0.896	<0.001
GEO: Georgia	2000-2003	-7.204	-7.553	-6.854	<0.001
GEO: Georgia	2003-2010	-9.755	-9.869	-9.64	<0.001
GEO: Georgia	2010-2013	-7.053	-7.75	-6.351	<0.001
GEO: Georgia	2013-2016	-3.569	-4.292	-2.841	<0.001
GEO: Georgia	2016-2021	-1.437	-1.602	-1.271	<0.001
GHA: Ghana	2000-2003	-3.406	-3.506	-3.307	<0.001
GHA: Ghana	2003-2008	-2.547	-2.61	-2.483	<0.001
GHA: Ghana	2008-2014	-3.759	-3.803	-3.715	<0.001
GHA: Ghana	2014-2017	-3.343	-3.542	-3.144	<0.001
GHA: Ghana	2017-2021	-2.937	-3.001	-2.874	<0.001

GIN: Guinea	2000-2004	-3.385	-3.671	-3.098	<0.001
GIN: Guinea	2004-2007	-2.569	-3.478	-1.651	<0.001
GIN: Guinea	2007-2011	-1.778	-2.237	-1.316	<0.001
GIN: Guinea	2011-2015	-1.206	-1.668	-0.742	<0.001
GIN: Guinea	2015-2021	-1.898	-2.053	-1.743	<0.001
GMB: Gambia	2000-2003	-2.893	-2.924	-2.861	<0.001
GMB: Gambia	2003-2007	-2.961	-2.992	-2.929	<0.001
GMB: Gambia	2007-2011	-2.888	-2.92	-2.857	<0.001
GMB: Gambia	2011-2017	-2.773	-2.787	-2.759	<0.001
GMB: Gambia	2017-2021	-2.527	-2.547	-2.507	<0.001
GNB: Guinea-Bissau	2000-2003	-2.908	-3.064	-2.753	<0.001
GNB: Guinea-Bissau	2003-2006	-3.655	-3.963	-3.346	<0.001
GNB: Guinea-Bissau	2006-2012	-4.224	-4.293	-4.156	<0.001
GNB: Guinea-Bissau	2012-2015	-3.546	-3.855	-3.237	<0.001
GNB: Guinea-Bissau	2015-2021	-2.962	-3.014	-2.909	<0.001
GNQ: Equatorial Guinea	2000-2002	-2.464	-2.714	-2.214	<0.001
GNQ: Equatorial Guinea	2002-2005	-2.905	-3.153	-2.656	<0.001
GNQ: Equatorial Guinea	2005-2012	-3.194	-3.236	-3.152	<0.001
GNQ: Equatorial Guinea	2012-2019	-2.887	-2.929	-2.845	<0.001
GNQ: Equatorial Guinea	2019-2021	-2.732	-2.981	-2.483	<0.001
GRC: Greece	2000-2004	-6.689	-6.939	-6.439	<0.001
GRC: Greece	2004-2007	-5.542	-6.339	-4.738	<0.001
GRC: Greece	2007-2010	-1.828	-2.657	-0.993	0.001
GRC: Greece	2010-2016	2.454	2.26	2.649	<0.001
GRC: Greece	2016-2021	-3.475	-3.658	-3.292	<0.001
GRD: Grenada	2000-2003	-0.151	-0.3	-0.003	0.046
GRD: Grenada	2003-2008	0.382	0.288	0.476	<0.001
GRD: Grenada	2008-2016	1.255	1.215	1.295	<0.001
GRD: Grenada	2016-2019	0.567	0.269	0.866	0.002
GRD: Grenada	2019-2021	-0.682	-0.977	-0.387	0.001
GTM: Guatemala	2000-2006	-3.456	-3.479	-3.433	<0.001
GTM: Guatemala	2006-2010	-3.581	-3.65	-3.513	<0.001
GTM: Guatemala	2010-2014	-3.718	-3.786	-3.649	<0.001
GTM: Guatemala	2014-2017	-3.457	-3.594	-3.32	<0.001
GTM: Guatemala	2017-2021	-3.095	-3.139	-3.052	<0.001
GUY: Guyana	2000-2004	-2.13	-2.193	-2.067	<0.001
GUY: Guyana	2004-2013	-1.816	-1.838	-1.794	<0.001
GUY: Guyana	2013-2017	-2.584	-2.684	-2.484	<0.001
GUY: Guyana	2017-2021	-2.775	-2.838	-2.712	<0.001
HND: Honduras	2000-2004	-3.946	-4.001	-3.891	<0.001
HND: Honduras	2004-2007	-3.784	-3.958	-3.61	<0.001
HND: Honduras	2007-2017	-3.451	-3.467	-3.435	<0.001
HND: Honduras	2017-2021	-3.373	-3.428	-3.318	<0.001
HRV: Croatia	2000-2002	-3.95	-4.318	-3.581	<0.001
HRV: Croatia	2002-2008	-4.425	-4.507	-4.343	<0.001
HRV: Croatia	2008-2011	-3.562	-3.931	-3.191	<0.001
HRV: Croatia	2011-2014	-2.181	-2.555	-1.804	<0.001

HRV: Croatia	2014-2021	-1.088	-1.139	-1.037	<0.001
HTI: Haiti	2000-2021	-2.231	-2.623	-1.837	<0.001
HUN: Hungary	2000-2004	-6.159	-6.357	-5.96	<0.001
HUN: Hungary	2004-2009	-4.877	-5.078	-4.675	<0.001
HUN: Hungary	2009-2014	-2.886	-3.091	-2.68	<0.001
HUN: Hungary	2014-2018	-6.233	-6.547	-5.919	<0.001
HUN: Hungary	2018-2021	-1.903	-2.23	-1.574	<0.001
IDN: Indonesia	2000-2021	-3.676	-3.767	-3.586	<0.001
IND: India	2000-2004	-3.503	-3.557	-3.449	<0.001
IND: India	2004-2007	-3.76	-3.931	-3.59	<0.001
IND: India	2007-2010	-4.342	-4.512	-4.173	<0.001
IND: India	2010-2013	-4.849	-5.018	-4.68	<0.001
IND: India	2013-2021	-5.146	-5.164	-5.128	<0.001
IRL: Ireland	2000-2002	-4.601	-5.104	-4.095	<0.001
IRL: Ireland	2002-2006	-7.039	-7.285	-6.793	<0.001
IRL: Ireland	2006-2009	-3.87	-4.377	-3.36	<0.001
IRL: Ireland	2009-2014	-2.099	-2.263	-1.935	<0.001
IRL: Ireland	2014-2021	-2.449	-2.518	-2.38	<0.001
IRN: Iran	2000-2002	-5.219	-5.483	-4.954	<0.001
IRN: Iran	2002-2009	-5.877	-5.921	-5.833	<0.001
IRN: Iran	2009-2012	-4.79	-5.055	-4.524	<0.001
IRN: Iran	2012-2015	-3.85	-4.118	-3.582	<0.001
IRN: Iran	2015-2021	-3.391	-3.437	-3.346	<0.001
IRQ: Iraq	2000-2005	-1.826	-1.851	-1.802	<0.001
IRQ: Iraq	2005-2008	-2.298	-2.406	-2.19	<0.001
IRQ: Iraq	2008-2011	-2.667	-2.775	-2.559	<0.001
IRQ: Iraq	2011-2019	-2.897	-2.911	-2.882	<0.001
IRQ: Iraq	2019-2021	-3.053	-3.16	-2.945	<0.001
ISL: Iceland	2000-2004	-5.561	-5.762	-5.359	<0.001
ISL: Iceland	2004-2008	-3.629	-3.954	-3.304	<0.001
ISL: Iceland	2008-2012	-1.38	-1.711	-1.047	<0.001
ISL: Iceland	2012-2019	0.413	0.299	0.527	<0.001
ISL: Iceland	2019-2021	-0.923	-1.588	-0.253	0.013
ISR: Israel	2000-2002	-3.783	-4.194	-3.369	<0.001
ISR: Israel	2002-2007	-4.586	-4.715	-4.457	<0.001
ISR: Israel	2007-2012	-3.497	-3.628	-3.366	<0.001
ISR: Israel	2012-2017	-2.49	-2.622	-2.358	<0.001
ISR: Israel	2017-2021	-2.843	-2.975	-2.712	<0.001
ITA: Italy	2000-2005	-4.353	-4.502	-4.203	<0.001
ITA: Italy	2005-2017	-2.26	-2.306	-2.214	<0.001
ITA: Italy	2017-2021	-5.914	-6.122	-5.706	<0.001
JAM: Jamaica	2000-2003	-2.134	-2.248	-2.02	<0.001
JAM: Jamaica	2003-2008	-1.479	-1.551	-1.406	<0.001
JAM: Jamaica	2008-2011	-2.299	-2.527	-2.07	<0.001
JAM: Jamaica	2011-2017	-2.843	-2.894	-2.792	<0.001
JAM: Jamaica	2017-2021	-3.226	-3.298	-3.155	<0.001
JOR: Jordan	2000-2002	-2.607	-2.671	-2.542	<0.001

JOR: Jordan	2002-2011	-2.695	-2.702	-2.688	<0.001
JOR: Jordan	2011-2015	-2.654	-2.686	-2.622	<0.001
JOR: Jordan	2015-2018	-2.907	-2.972	-2.843	<0.001
JOR: Jordan	2018-2021	-3.002	-3.034	-2.97	<0.001
JPN: Japan	2000-2002	-3.961	-4.89	-3.024	<0.001
JPN: Japan	2002-2014	-3.089	-3.153	-3.026	<0.001
JPN: Japan	2014-2021	-2.437	-2.563	-2.31	<0.001
KAZ: Kazakhstan	2000-2006	-5.785	-5.883	-5.687	<0.001
KAZ: Kazakhstan	2006-2009	-7.717	-8.283	-7.147	<0.001
KAZ: Kazakhstan	2009-2015	-10.422	-10.545	-10.299	<0.001
KAZ: Kazakhstan	2015-2018	-4.797	-5.382	-4.21	<0.001
KAZ: Kazakhstan	2018-2021	0.452	0.143	0.762	0.01
KEN: Kenya	2000-2003	-4.102	-4.677	-3.524	<0.001
KEN: Kenya	2003-2008	-5.143	-5.503	-4.782	<0.001
KEN: Kenya	2008-2013	-2.379	-2.749	-2.007	<0.001
KEN: Kenya	2013-2016	-3.576	-4.728	-2.41	<0.001
KEN: Kenya	2016-2021	-2.678	-2.939	-2.416	<0.001
KGZ: Kyrgyzstan	2000-2003	-4.518	-4.764	-4.272	<0.001
KGZ: Kyrgyzstan	2003-2007	-4.007	-4.254	-3.76	<0.001
KGZ: Kyrgyzstan	2007-2012	-5.947	-6.1	-5.794	<0.001
KGZ: Kyrgyzstan	2012-2018	-5.053	-5.162	-4.943	<0.001
KGZ: Kyrgyzstan	2018-2021	-2.972	-3.222	-2.722	<0.001
KHM: Cambodia	2000-2004	-7.995	-8.313	-7.676	<0.001
KHM: Cambodia	2004-2013	-6.803	-6.914	-6.691	<0.001
KHM: Cambodia	2013-2016	-5	-6.034	-3.954	<0.001
KHM: Cambodia	2016-2021	-3.891	-4.126	-3.655	<0.001
KIR: Kiribati	2000-2003	-1.473	-1.662	-1.284	<0.001
KIR: Kiribati	2003-2009	0.255	0.169	0.341	<0.001
KIR: Kiribati	2009-2012	-1.433	-1.81	-1.054	<0.001
KIR: Kiribati	2012-2016	-2.222	-2.409	-2.034	<0.001
KIR: Kiribati	2016-2021	-2.599	-2.682	-2.515	<0.001
KNA: Saint Kitts and Nevis	2000-2005	-4.363	-4.487	-4.239	<0.001
KNA: Saint Kitts and Nevis	2005-2008	-2.183	-2.748	-1.615	<0.001
KNA: Saint Kitts and Nevis	2008-2014	-0.495	-0.624	-0.366	<0.001
KNA: Saint Kitts and Nevis	2014-2017	-1.693	-2.261	-1.121	<0.001
KNA: Saint Kitts and Nevis	2017-2021	-2.671	-2.85	-2.493	<0.001
KOR: South Korea	2000-2003	-5.318	-5.5	-5.135	<0.001
KOR: South Korea	2003-2008	-6.756	-6.869	-6.642	<0.001
KOR: South Korea	2008-2011	-4.267	-4.635	-3.897	<0.001
KOR: South Korea	2011-2019	-2.888	-2.938	-2.838	<0.001
KOR: South Korea	2019-2021	-3.979	-4.348	-3.609	<0.001
KWT: Kuwait	2000-2009	-1.595	-1.64	-1.55	<0.001
KWT: Kuwait	2009-2014	-3.499	-3.652	-3.345	<0.001
KWT: Kuwait	2014-2017	-1.6	-2.094	-1.103	<0.001
KWT: Kuwait	2017-2021	-0.254	-0.413	-0.095	0.005
LAO: Laos	2000-2004	-3.594	-3.64	-3.548	<0.001
LAO: Laos	2004-2011	-4.014	-4.038	-3.989	<0.001

LAO: Laos	2011-2014	-4.166	-4.311	-4.022	<0.001
LAO: Laos	2014-2017	-3.739	-3.884	-3.594	<0.001
LAO: Laos	2017-2021	-3.23	-3.276	-3.183	<0.001
LBN: Lebanon	2000-2002	-5.49	-5.991	-4.985	<0.001
LBN: Lebanon	2002-2008	-5.977	-6.089	-5.865	<0.001
LBN: Lebanon	2008-2011	-4.567	-5.074	-4.058	<0.001
LBN: Lebanon	2011-2014	-3.552	-4.064	-3.037	<0.001
LBN: Lebanon	2014-2021	-2.235	-2.305	-2.165	<0.001
LBR: Liberia	2000-2006	-6.568	-6.95	-6.185	<0.001
LBR: Liberia	2006-2009	-4.683	-6.966	-2.345	0.001
LBR: Liberia	2009-2021	-2.109	-2.249	-1.969	<0.001
LBY: Libya	2000-2015	-5.201	-5.533	-4.867	<0.001
LBY: Libya	2015-2021	-3.315	-4.665	-1.946	<0.001
LCA: Saint Lucia	2000-2003	0.151	0.002	0.3	0.048
LCA: Saint Lucia	2003-2007	1.057	0.907	1.207	<0.001
LCA: Saint Lucia	2007-2011	1.92	1.769	2.071	<0.001
LCA: Saint Lucia	2011-2019	2.609	2.568	2.649	<0.001
LCA: Saint Lucia	2019-2021	1.672	1.371	1.975	<0.001
LKA: Sri Lanka	2000-2021	-4.673	-5.351	-3.989	<0.001
LSO: Lesotho	2000-2008	0.867	0.578	1.158	<0.001
LSO: Lesotho	2008-2011	-4.023	-6.522	-1.457	0.006
LSO: Lesotho	2011-2016	-0.46	-1.287	0.374	0.249
LSO: Lesotho	2016-2021	-1.987	-2.563	-1.407	<0.001
LTU: Lithuania	2000-2006	-3.257	-3.574	-2.938	<0.001
LTU: Lithuania	2006-2012	-8.214	-8.612	-7.814	<0.001
LTU: Lithuania	2012-2016	-1.931	-2.88	-0.973	0.001
LTU: Lithuania	2016-2021	-7.002	-7.405	-6.596	<0.001
LUX: Luxembourg	2000-2005	-5.898	-6.037	-5.759	<0.001
LUX: Luxembourg	2005-2008	-4.064	-4.695	-3.429	<0.001
LUX: Luxembourg	2008-2012	-1.87	-2.194	-1.546	<0.001
LUX: Luxembourg	2012-2019	0.289	0.177	0.401	<0.001
LUX: Luxembourg	2019-2021	-1.454	-2.102	-0.802	0.001
LVA: Latvia	2000-2011	-5.585	-5.627	-5.543	<0.001
LVA: Latvia	2011-2015	-8.642	-8.945	-8.338	<0.001
LVA: Latvia	2015-2018	-5.926	-6.55	-5.299	<0.001
LVA: Latvia	2018-2021	-3.807	-4.126	-3.486	<0.001
MAR: Morocco	2000-2002	-3.849	-4.05	-3.648	<0.001
MAR: Morocco	2002-2005	-4.361	-4.561	-4.161	<0.001
MAR: Morocco	2005-2016	-5.468	-5.483	-5.452	<0.001
MAR: Morocco	2016-2019	-4.436	-4.635	-4.236	<0.001
MAR: Morocco	2019-2021	-3.947	-4.147	-3.745	<0.001
MCO: Monaco	2000-2002	-3.064	-3.414	-2.714	<0.001
MCO: Monaco	2002-2005	-2.585	-2.936	-2.232	<0.001
MCO: Monaco	2005-2012	-2.3	-2.36	-2.24	<0.001
MCO: Monaco	2012-2015	-2.763	-3.114	-2.412	<0.001
MCO: Monaco	2015-2021	-3.307	-3.366	-3.248	<0.001
MDA: Moldova	2000-2004	-8.943	-9.132	-8.754	<0.001

MDA: Moldova	2004-2007	-4.862	-5.485	-4.236	<0.001
MDA: Moldova	2007-2021	-1.625	-1.655	-1.595	<0.001
MDG: Madagascar	2000-2005	-3.739	-3.812	-3.665	<0.001
MDG: Madagascar	2005-2009	-3.138	-3.303	-2.972	<0.001
MDG: Madagascar	2009-2012	-1.942	-2.278	-1.606	<0.001
MDG: Madagascar	2012-2015	-0.795	-1.135	-0.455	0.001
MDG: Madagascar	2015-2021	0.059	0.001	0.116	0.048
MDV: Maldives	2000-2007	-10.282	-10.674	-9.889	<0.001
MDV: Maldives	2007-2015	-6.95	-7.356	-6.542	<0.001
MDV: Maldives	2015-2021	-8.049	-8.556	-7.539	<0.001
MEX: Mexico	2000-2003	-4.013	-4.062	-3.964	<0.001
MEX: Mexico	2003-2006	-3.595	-3.693	-3.496	<0.001
MEX: Mexico	2006-2009	-3.317	-3.416	-3.218	<0.001
MEX: Mexico	2009-2016	-3.211	-3.228	-3.194	<0.001
MEX: Mexico	2016-2021	-3.32	-3.342	-3.298	<0.001
MHL: Marshall Islands	2000-2003	-0.165	-0.283	-0.046	0.013
MHL: Marshall Islands	2003-2011	-0.855	-0.886	-0.823	<0.001
MHL: Marshall Islands	2011-2014	-1.803	-2.036	-1.569	<0.001
MHL: Marshall Islands	2014-2018	-2.383	-2.499	-2.267	<0.001
MHL: Marshall Islands	2018-2021	-2.908	-3.023	-2.793	<0.001
MKD: North Macedonia	2000-2005	-2.603	-4.014	-1.172	0.002
MKD: North Macedonia	2005-2011	-5.123	-6.497	-3.729	<0.001
MKD: North Macedonia	2011-2016	2.218	0.13	4.348	0.039
MKD: North Macedonia	2016-2021	-14.598	-15.835	-13.343	<0.001
MLI: Mali	2000-2005	-2.693	-2.717	-2.67	<0.001
MLI: Mali	2005-2008	-2.513	-2.619	-2.407	<0.001
MLI: Mali	2008-2012	-2.328	-2.381	-2.275	<0.001
MLI: Mali	2012-2019	-2.106	-2.124	-2.088	<0.001
MLI: Mali	2019-2021	-2.213	-2.319	-2.107	<0.001
MLT: Malta	2000-2002	-3.054	-3.427	-2.68	<0.001
MLT: Malta	2002-2005	-1.396	-1.775	-1.016	<0.001
MLT: Malta	2005-2013	-0.242	-0.293	-0.191	<0.001
MLT: Malta	2013-2017	-1.399	-1.589	-1.209	<0.001
MLT: Malta	2017-2021	-2.337	-2.456	-2.218	<0.001
MMR: Myanmar	2000-2021	-3.254	-3.57	-2.938	<0.001
MNE: Montenegro	2000-2003	-5.139	-5.485	-4.791	<0.001
MNE: Montenegro	2003-2006	-7.55	-8.224	-6.872	<0.001
MNE: Montenegro	2006-2010	-9	-9.333	-8.667	<0.001
MNE: Montenegro	2010-2019	-9.929	-10.001	-9.857	<0.001
MNE: Montenegro	2019-2021	-8.728	-9.393	-8.058	<0.001
MNG: Mongolia	2000-2002	-6.966	-7.217	-6.714	<0.001
MNG: Mongolia	2002-2010	-7.795	-7.828	-7.762	<0.001
MNG: Mongolia	2010-2013	-6.153	-6.407	-5.899	<0.001
MNG: Mongolia	2013-2019	-4.557	-4.615	-4.499	<0.001
MNG: Mongolia	2019-2021	-4.093	-4.352	-3.833	<0.001
MOZ: Mozambique	2000-2008	-4.96	-5.178	-4.742	<0.001
MOZ: Mozambique	2008-2021	-2.859	-2.966	-2.752	<0.001

MRT: Mauritania	2000-2002	-3.864	-4.072	-3.655	<0.001
MRT: Mauritania	2002-2006	-4.86	-4.963	-4.757	<0.001
MRT: Mauritania	2006-2009	-3.646	-3.855	-3.437	<0.001
MRT: Mauritania	2009-2015	-2.176	-2.223	-2.128	<0.001
MRT: Mauritania	2015-2021	-2.39	-2.426	-2.354	<0.001
MSR: Montserrat	2000-2003	-4.03	-4.134	-3.927	<0.001
MSR: Montserrat	2003-2009	-3.445	-3.491	-3.398	<0.001
MSR: Montserrat	2009-2021	-3.264	-3.276	-3.251	<0.001
MUS: Mauritius	2000-2002	-7.57	-9.212	-5.898	<0.001
MUS: Mauritius	2002-2009	-1.826	-2.123	-1.528	<0.001
MUS: Mauritius	2009-2013	1.002	0.1	1.911	0.033
MUS: Mauritius	2013-2016	-0.169	-1.943	1.638	0.834
MUS: Mauritius	2016-2021	3.631	3.216	4.047	<0.001
MWI: Malawi	2000-2005	-8.497	-8.814	-8.179	<0.001
MWI: Malawi	2005-2010	-4.3	-4.768	-3.83	<0.001
MWI: Malawi	2010-2014	-6.074	-6.799	-5.342	<0.001
MWI: Malawi	2014-2018	-4.11	-4.851	-3.364	<0.001
MWI: Malawi	2018-2021	-2.892	-3.642	-2.136	<0.001
MYS: Malaysia	2000-2002	-6.81	-7.928	-5.678	<0.001
MYS: Malaysia	2002-2005	-2.738	-3.905	-1.556	<0.001
MYS: Malaysia	2005-2017	-0.012	-0.093	0.069	0.75
MYS: Malaysia	2017-2021	-1.71	-2.084	-1.334	<0.001
NAM: Namibia	2000-2003	-1.254	-3.142	0.672	0.17
NAM: Namibia	2003-2006	-4.307	-7.932	-0.538	0.03
NAM: Namibia	2006-2012	-0.049	-0.908	0.818	0.9
NAM: Namibia	2012-2015	-4.253	-7.881	-0.483	0.032
NAM: Namibia	2015-2021	-1.831	-2.469	-1.187	<0.001
NER: Niger	2000-2002	-3.863	-4.177	-3.549	<0.001
NER: Niger	2002-2007	-4.479	-4.578	-4.38	<0.001
NER: Niger	2007-2010	-3.199	-3.515	-2.882	<0.001
NER: Niger	2010-2014	-1.569	-1.73	-1.408	<0.001
NER: Niger	2014-2021	-0.466	-0.51	-0.423	<0.001
NGA: Nigeria	2000-2007	-2.82	-2.857	-2.782	<0.001
NGA: Nigeria	2007-2010	-2.114	-2.394	-1.832	<0.001
NGA: Nigeria	2010-2017	-1.233	-1.281	-1.186	<0.001
NGA: Nigeria	2017-2021	-2.201	-2.29	-2.112	<0.001
NIC: Nicaragua	2000-2003	-4.485	-4.566	-4.403	<0.001
NIC: Nicaragua	2003-2011	-4.187	-4.209	-4.166	<0.001
NIC: Nicaragua	2011-2014	-4.613	-4.775	-4.45	<0.001
NIC: Nicaragua	2014-2017	-5.249	-5.41	-5.087	<0.001
NIC: Nicaragua	2017-2021	-5.721	-5.772	-5.67	<0.001
NIU: Niue	2000-2002	2.691	2.08	3.306	<0.001
NIU: Niue	2002-2005	1.543	0.939	2.151	<0.001
NIU: Niue	2005-2008	-0.719	-1.31	-0.124	0.024
NIU: Niue	2008-2011	-2.009	-2.592	-1.423	<0.001
NIU: Niue	2011-2021	-2.785	-2.831	-2.74	<0.001
NLD: Netherlands	2000-2003	-2.389	-2.538	-2.24	<0.001

NLD: Netherlands	2003-2009	-3.589	-3.655	-3.523	<0.001
NLD: Netherlands	2009-2012	-2.397	-2.695	-2.098	<0.001
NLD: Netherlands	2012-2015	-1.057	-1.359	-0.754	<0.001
NLD: Netherlands	2015-2021	0.203	0.151	0.255	<0.001
NOR: Norway	2000-2002	-3.094	-3.241	-2.946	<0.001
NOR: Norway	2002-2006	-3.857	-3.931	-3.784	<0.001
NOR: Norway	2006-2012	-4.194	-4.226	-4.161	<0.001
NOR: Norway	2012-2017	-3.298	-3.345	-3.252	<0.001
NOR: Norway	2017-2021	-3.794	-3.84	-3.747	<0.001
NPL: Nepal	2000-2005	-4.803	-4.845	-4.76	<0.001
NPL: Nepal	2005-2013	-4.398	-4.423	-4.372	<0.001
NPL: Nepal	2013-2018	-4.472	-4.532	-4.412	<0.001
NPL: Nepal	2018-2021	-3.592	-3.688	-3.496	<0.001
NRU: Nauru	2000-2004	-2.117	-2.279	-1.954	<0.001
NRU: Nauru	2004-2009	-0.206	-0.372	-0.04	0.021
NRU: Nauru	2009-2012	-1.551	-2.067	-1.032	<0.001
NRU: Nauru	2012-2018	-2.508	-2.622	-2.393	<0.001
NRU: Nauru	2018-2021	-3.09	-3.344	-2.835	<0.001
NZL: New Zealand	2000-2004	-2.259	-2.352	-2.167	<0.001
NZL: New Zealand	2004-2010	-1.195	-1.261	-1.129	<0.001
NZL: New Zealand	2010-2013	-2.064	-2.356	-1.771	<0.001
NZL: New Zealand	2013-2018	-2.874	-2.965	-2.782	<0.001
NZL: New Zealand	2018-2021	-2.048	-2.194	-1.902	<0.001
OMN: Oman	2000-2002	-6.109	-6.572	-5.644	<0.001
OMN: Oman	2002-2005	-4.164	-4.637	-3.689	<0.001
OMN: Oman	2005-2008	-1.91	-2.394	-1.423	<0.001
OMN: Oman	2008-2018	-1.003	-1.047	-0.958	<0.001
OMN: Oman	2018-2021	-2.21	-2.452	-1.968	<0.001
PAK: Pakistan	2000-2002	-2.235	-2.402	-2.068	<0.001
PAK: Pakistan	2002-2010	-1.757	-1.78	-1.735	<0.001
PAK: Pakistan	2010-2013	-2.301	-2.468	-2.133	<0.001
PAK: Pakistan	2013-2018	-2.607	-2.659	-2.554	<0.001
PAK: Pakistan	2018-2021	-2.769	-2.852	-2.686	<0.001
PAN: Panama	2000-2006	-2.343	-2.382	-2.303	<0.001
PAN: Panama	2006-2009	-2.825	-3.059	-2.591	<0.001
PAN: Panama	2009-2016	-2.993	-3.033	-2.954	<0.001
PAN: Panama	2016-2021	-3.291	-3.343	-3.238	<0.001
PER: Peru	2000-2004	-6.999	-7.15	-6.848	<0.001
PER: Peru	2004-2008	-5.869	-6.111	-5.627	<0.001
PER: Peru	2008-2011	-4.837	-5.325	-4.347	<0.001
PER: Peru	2011-2014	-3.65	-4.144	-3.154	<0.001
PER: Peru	2014-2021	-2.605	-2.672	-2.538	<0.001
PHL: Philippines	2000-2007	-1.578	-1.602	-1.553	<0.001
PHL: Philippines	2007-2014	-1.092	-1.124	-1.061	<0.001
PHL: Philippines	2014-2017	-1.424	-1.608	-1.24	<0.001
PHL: Philippines	2017-2021	-2.579	-2.637	-2.521	<0.001
PLW: Palau	2000-2007	-1.596	-1.641	-1.551	<0.001

PLW: Palau	2007-2010	-2.368	-2.702	-2.032	<0.001
PLW: Palau	2010-2013	-2.78	-3.113	-2.445	<0.001
PLW: Palau	2013-2021	-3.107	-3.143	-3.071	<0.001
PNG: Papua New Guinea	2000-2004	-1.481	-1.523	-1.439	<0.001
PNG: Papua New Guinea	2004-2007	-1.767	-1.899	-1.634	<0.001
PNG: Papua New Guinea	2007-2012	-2.085	-2.127	-2.044	<0.001
PNG: Papua New Guinea	2012-2016	-2.259	-2.325	-2.193	<0.001
PNG: Papua New Guinea	2016-2021	-2.613	-2.642	-2.583	<0.001
POL: Poland	2000-2002	-4.937	-5.841	-4.023	<0.001
POL: Poland	2002-2007	-3.553	-3.844	-3.261	<0.001
POL: Poland	2007-2012	-5.347	-5.633	-5.061	<0.001
POL: Poland	2012-2019	-2.855	-3.012	-2.697	<0.001
POL: Poland	2019-2021	-0.704	-1.649	0.25	0.127
PRK: North Korea	2000-2004	-11.409	-11.722	-11.094	<0.001
PRK: North Korea	2004-2009	-1.935	-2.281	-1.587	<0.001
PRK: North Korea	2009-2015	-7.058	-7.291	-6.825	<0.001
PRK: North Korea	2015-2019	-4.721	-5.253	-4.186	<0.001
PRK: North Korea	2019-2021	-12.024	-13.003	-11.033	<0.001
PRT: Portugal	2000-2005	-8.354	-8.609	-8.098	<0.001
PRT: Portugal	2005-2008	-4.027	-5.218	-2.822	<0.001
PRT: Portugal	2008-2011	-1.968	-3.184	-0.737	0.006
PRT: Portugal	2011-2018	-0.232	-0.442	-0.021	0.035
PRT: Portugal	2018-2021	-4.661	-5.254	-4.064	<0.001
PRY: Paraguay	2000-2005	-2.203	-2.249	-2.156	<0.001
PRY: Paraguay	2005-2009	-2.575	-2.678	-2.472	<0.001
PRY: Paraguay	2009-2015	-2.887	-2.933	-2.841	<0.001
PRY: Paraguay	2015-2019	-3.242	-3.345	-3.14	<0.001
PRY: Paraguay	2019-2021	-3.05	-3.255	-2.845	<0.001
PSE: Palestinian Territory	2000-2014	-2.996	-3.058	-2.934	<0.001
PSE: Palestinian Territory	2014-2021	-3.838	-4.012	-3.663	<0.001
QAT: Qatar	2000-2005	-3.681	-3.77	-3.592	<0.001
QAT: Qatar	2005-2013	-2.525	-2.578	-2.471	<0.001
QAT: Qatar	2013-2016	-4.523	-4.916	-4.128	<0.001
QAT: Qatar	2016-2021	-6.411	-6.497	-6.324	<0.001
ROU: Romania	2000-2005	-2.627	-2.913	-2.34	<0.001
ROU: Romania	2005-2008	-9.288	-10.472	-8.088	<0.001
ROU: Romania	2008-2013	-5.276	-5.668	-4.881	<0.001
ROU: Romania	2013-2018	-7.443	-7.827	-7.058	<0.001
ROU: Romania	2018-2021	-4.942	-5.564	-4.315	<0.001
RUS: Russian Federation	2000-2002	-5.443	-7.305	-3.544	<0.001
RUS: Russian Federation	2002-2008	-7.115	-7.527	-6.701	<0.001
RUS: Russian Federation	2008-2013	-2.942	-3.551	-2.33	<0.001
RUS: Russian Federation	2013-2021	-7.77	-7.969	-7.569	<0.001
RWA: Rwanda	2000-2007	-9.67	-9.807	-9.533	<0.001
RWA: Rwanda	2007-2011	-6.712	-7.239	-6.181	<0.001
RWA: Rwanda	2011-2014	-4.443	-5.52	-3.353	<0.001
RWA: Rwanda	2014-2021	-2.638	-2.786	-2.49	<0.001

SAU: Saudi Arabia	2000-2007	-5.502	-5.537	-5.467	<0.001
SAU: Saudi Arabia	2007-2015	-5.871	-5.906	-5.836	<0.001
SAU: Saudi Arabia	2015-2018	-5.525	-5.784	-5.265	<0.001
SAU: Saudi Arabia	2018-2021	-4.392	-4.523	-4.26	<0.001
SDN: Sudan	2000-2005	-2.907	-2.932	-2.882	<0.001
SDN: Sudan	2005-2008	-2.661	-2.772	-2.549	<0.001
SDN: Sudan	2008-2015	-2.302	-2.321	-2.283	<0.001
SDN: Sudan	2015-2021	-2.483	-2.502	-2.465	<0.001
SEN: Senegal	2000-2002	-4.085	-4.205	-3.965	<0.001
SEN: Senegal	2002-2006	-5.182	-5.241	-5.122	<0.001
SEN: Senegal	2006-2009	-4.278	-4.398	-4.158	<0.001
SEN: Senegal	2009-2018	-3.585	-3.598	-3.571	<0.001
SEN: Senegal	2018-2021	-2.957	-3.017	-2.896	<0.001
SGP: Singapore	2000-2003	-6.527	-7.018	-6.034	<0.001
SGP: Singapore	2003-2006	-2.827	-3.844	-1.799	<0.001
SGP: Singapore	2006-2017	-0.29	-0.372	-0.208	<0.001
SGP: Singapore	2017-2021	-5.683	-5.996	-5.369	<0.001
SLB: Solomon Islands	2000-2006	-0.95	-0.979	-0.921	<0.001
SLB: Solomon Islands	2006-2009	-1.889	-2.056	-1.721	<0.001
SLB: Solomon Islands	2009-2015	-2.688	-2.725	-2.651	<0.001
SLB: Solomon Islands	2015-2021	-3.052	-3.08	-3.024	<0.001
SLE: Sierra Leone	2000-2005	-2.128	-2.58	-1.673	<0.001
SLE: Sierra Leone	2005-2021	-2.881	-2.958	-2.804	<0.001
SLV: El Salvador	2000-2005	-5.066	-5.106	-5.025	<0.001
SLV: El Salvador	2005-2011	-4.791	-4.832	-4.75	<0.001
SLV: El Salvador	2011-2014	-4.334	-4.519	-4.15	<0.001
SLV: El Salvador	2014-2017	-3.748	-3.933	-3.562	<0.001
SLV: El Salvador	2017-2021	-3.42	-3.479	-3.361	<0.001
SMR: San Marino	2000-2003	-7.436	-7.636	-7.235	<0.001
SMR: San Marino	2003-2006	-6.559	-6.963	-6.153	<0.001
SMR: San Marino	2006-2010	-5.844	-6.047	-5.639	<0.001
SMR: San Marino	2010-2017	-4.844	-4.914	-4.774	<0.001
SMR: San Marino	2017-2021	-3.868	-4	-3.737	<0.001
SOM: Somalia	2000-2006	0.003	-0.052	0.059	0.897
SOM: Somalia	2006-2009	-1.774	-2.097	-1.45	<0.001
SOM: Somalia	2009-2021	-2.746	-2.765	-2.727	<0.001
SRB: Serbia	2000-2002	-5.274	-5.852	-4.693	<0.001
SRB: Serbia	2002-2006	-7.158	-7.441	-6.873	<0.001
SRB: Serbia	2006-2011	-2.654	-2.842	-2.466	<0.001
SRB: Serbia	2011-2017	-3.679	-3.811	-3.548	<0.001
SRB: Serbia	2017-2021	-1.721	-1.911	-1.531	<0.001
SSD: South Sudan	2000-2010	-4.436	-4.46	-4.411	<0.001
SSD: South Sudan	2010-2013	-2.778	-3.096	-2.458	<0.001
SSD: South Sudan	2013-2021	0.012	-0.023	0.048	0.468
STP: Sao Tome and Principe	2000-2003	-6.027	-6.27	-5.784	<0.001
STP: Sao Tome and Principe	2003-2011	-6.715	-6.78	-6.651	<0.001
STP: Sao Tome and Principe	2011-2018	-8.043	-8.124	-7.963	<0.001

STP: Sao Tome and Principe	2018-2021	-5.762	-6.005	-5.518	<0.001
SUR: Suriname	2000-2004	-3.074	-3.139	-3.008	<0.001
SUR: Suriname	2004-2008	-2.669	-2.772	-2.565	<0.001
SUR: Suriname	2008-2016	-2.49	-2.518	-2.462	<0.001
SUR: Suriname	2016-2021	-2.936	-2.982	-2.89	<0.001
SVK: Slovakia	2000-2004	-3.613	-3.672	-3.554	<0.001
SVK: Slovakia	2004-2009	-3.363	-3.422	-3.303	<0.001
SVK: Slovakia	2009-2012	-2.695	-2.883	-2.506	<0.001
SVK: Slovakia	2012-2016	-2.041	-2.136	-1.946	<0.001
SVK: Slovakia	2016-2021	-1.703	-1.745	-1.66	<0.001
SVN: Slovenia	2000-2010	-5.296	-5.306	-5.287	<0.001
SVN: Slovenia	2010-2013	-4.602	-4.728	-4.475	<0.001
SVN: Slovenia	2013-2016	-3.836	-3.963	-3.709	<0.001
SVN: Slovenia	2016-2021	-2.993	-3.021	-2.964	<0.001
SWE: Sweden	2000-2002	-1.289	-1.976	-0.597	0.002
SWE: Sweden	2002-2009	-3.657	-3.771	-3.544	<0.001
SWE: Sweden	2009-2017	-1.426	-1.518	-1.334	<0.001
SWE: Sweden	2017-2021	-2.977	-3.191	-2.762	<0.001
SWZ: Eswatini	2000-2003	1.76	-2.464	6.167	0.397
SWZ: Eswatini	2003-2021	-2.884	-3.148	-2.619	<0.001
SYC: Seychelles	2000-2005	0.176	0.103	0.248	<0.001
SYC: Seychelles	2005-2016	0.405	0.38	0.431	<0.001
SYC: Seychelles	2016-2019	-0.4	-0.72	-0.078	0.019
SYC: Seychelles	2019-2021	-1.805	-2.121	-1.488	<0.001
SYR: Syria	2000-2010	-2.156	-3.436	-0.86	0.003
SYR: Syria	2010-2013	21.805	2.855	44.247	0.025
SYR: Syria	2013-2021	-5.746	-7.469	-3.991	<0.001
TCA: Turks and Caicos Islands	2000-2004	-4.041	-4.12	-3.962	<0.001
TCA: Turks and Caicos Islands	2004-2007	-3.421	-3.672	-3.169	<0.001
TCA: Turks and Caicos Islands	2007-2011	-2.688	-2.815	-2.561	<0.001
TCA: Turks and Caicos Islands	2011-2018	-2.289	-2.332	-2.246	<0.001
TCA: Turks and Caicos Islands	2018-2021	-2.567	-2.694	-2.44	<0.001
TCD: Chad	2000-2005	-1.49	-1.516	-1.465	<0.001
TCD: Chad	2005-2008	-1.776	-1.889	-1.663	<0.001
TCD: Chad	2008-2013	-2.003	-2.038	-1.967	<0.001
TCD: Chad	2013-2016	-2.095	-2.208	-1.983	<0.001
TCD: Chad	2016-2021	-2.379	-2.404	-2.354	<0.001
TGO: Togo	2000-2005	-2.624	-2.636	-2.612	<0.001
TGO: Togo	2005-2009	-2.505	-2.532	-2.478	<0.001
TGO: Togo	2009-2012	-2.639	-2.693	-2.585	<0.001
TGO: Togo	2012-2018	-2.731	-2.743	-2.719	<0.001
TGO: Togo	2018-2021	-2.538	-2.565	-2.511	<0.001
THA: Thailand	2000-2007	-4.627	-4.644	-4.611	<0.001
THA: Thailand	2007-2019	-4.51	-4.518	-4.502	<0.001
THA: Thailand	2019-2021	-4.065	-4.187	-3.942	<0.001
TJK: Tajikistan	2000-2005	-7.036	-7.112	-6.96	<0.001
TJK: Tajikistan	2005-2008	-5.523	-5.867	-5.178	<0.001

TJK: Tajikistan	2008-2011	-3.284	-3.636	-2.93	<0.001
TJK: Tajikistan	2011-2017	-2.376	-2.455	-2.296	<0.001
TJK: Tajikistan	2017-2021	-2.848	-2.96	-2.736	<0.001
TKM: Turkmenistan	2000-2006	-5.064	-5.164	-4.965	<0.001
TKM: Turkmenistan	2006-2009	-3.486	-4.081	-2.886	<0.001
TKM: Turkmenistan	2009-2012	-1.512	-2.12	-0.901	<0.001
TKM: Turkmenistan	2012-2018	0.49	0.351	0.629	<0.001
TKM: Turkmenistan	2018-2021	-1.246	-1.551	-0.94	<0.001
TLS: Timor-Leste	2000-2007	-4.098	-4.125	-4.071	<0.001
TLS: Timor-Leste	2007-2010	-3.314	-3.518	-3.108	<0.001
TLS: Timor-Leste	2010-2015	-2.558	-2.623	-2.492	<0.001
TLS: Timor-Leste	2015-2021	-2.942	-2.977	-2.907	<0.001
TON: Tonga	2000-2006	-2.733	-2.791	-2.675	<0.001
TON: Tonga	2006-2011	-2.095	-2.204	-1.986	<0.001
TON: Tonga	2011-2018	-1.091	-1.15	-1.032	<0.001
TON: Tonga	2018-2021	-2.32	-2.492	-2.147	<0.001
TTO: Trinidad and Tobago	2000-2003	-0.94	-1.099	-0.781	<0.001
TTO: Trinidad and Tobago	2003-2006	-2.074	-2.388	-1.758	<0.001
TTO: Trinidad and Tobago	2006-2010	-2.702	-2.858	-2.545	<0.001
TTO: Trinidad and Tobago	2010-2021	-2.986	-3.007	-2.965	<0.001
TUN: Tunisia	2000-2005	-5.585	-5.763	-5.407	<0.001
TUN: Tunisia	2005-2009	-3.684	-4.09	-3.276	<0.001
TUN: Tunisia	2009-2013	-1.765	-2.179	-1.349	<0.001
TUN: Tunisia	2013-2019	-0.501	-0.688	-0.312	<0.001
TUN: Tunisia	2019-2021	-1.621	-2.449	-0.786	0.002
TUR: Turkiye	2000-2003	-6.476	-6.538	-6.413	<0.001
TUR: Turkiye	2003-2010	-6.727	-6.748	-6.706	<0.001
TUR: Turkiye	2010-2013	-6.537	-6.662	-6.413	<0.001
TUR: Turkiye	2013-2019	-6.222	-6.25	-6.194	<0.001
TUR: Turkiye	2019-2021	-5.594	-5.719	-5.468	<0.001
TUV: Tuvalu	2000-2005	-1.859	-1.951	-1.768	<0.001
TUV: Tuvalu	2005-2008	-3.099	-3.503	-2.694	<0.001
TUV: Tuvalu	2008-2011	-3.744	-4.145	-3.341	<0.001
TUV: Tuvalu	2011-2021	-3.203	-3.235	-3.172	<0.001
TZA: Tanzania	2000-2005	-5.72	-6.044	-5.395	<0.001
TZA: Tanzania	2005-2009	-4.555	-5.287	-3.818	<0.001
TZA: Tanzania	2009-2021	-2.939	-3.027	-2.85	<0.001
UGA: Uganda	2000-2002	-4.531	-5.035	-4.025	<0.001
UGA: Uganda	2002-2007	-6.078	-6.235	-5.921	<0.001
UGA: Uganda	2007-2012	-5.143	-5.302	-4.985	<0.001
UGA: Uganda	2012-2018	-4.252	-4.365	-4.139	<0.001
UGA: Uganda	2018-2021	-3.292	-3.548	-3.036	<0.001
UKR: Ukraine	2000-2005	-4.442	-4.547	-4.337	<0.001
UKR: Ukraine	2005-2008	-3.82	-4.293	-3.345	<0.001
UKR: Ukraine	2008-2013	-4.738	-4.886	-4.59	<0.001
UKR: Ukraine	2013-2017	-3.411	-3.648	-3.172	<0.001
UKR: Ukraine	2017-2021	-1.895	-2.048	-1.742	<0.001

URY: Uruguay	2000-2004	-2.753	-2.884	-2.621	<0.001
URY: Uruguay	2004-2009	-6.083	-6.21	-5.956	<0.001
URY: Uruguay	2009-2015	-3.756	-3.849	-3.664	<0.001
URY: Uruguay	2015-2018	-6.42	-6.82	-6.018	<0.001
URY: Uruguay	2018-2021	-7.65	-7.848	-7.452	<0.001
USA: United States	2000-2007	-1.084	-1.116	-1.052	<0.001
USA: United States	2007-2012	-1.747	-1.822	-1.672	<0.001
USA: United States	2012-2019	-1.251	-1.292	-1.211	<0.001
USA: United States	2019-2021	-1.485	-1.723	-1.247	<0.001
UZB: Uzbekistan	2000-2002	-5.296	-5.53	-5.063	<0.001
UZB: Uzbekistan	2002-2005	-6.681	-6.911	-6.45	<0.001
UZB: Uzbekistan	2005-2013	-7.65	-7.681	-7.62	<0.001
UZB: Uzbekistan	2013-2017	-6.199	-6.315	-6.083	<0.001
UZB: Uzbekistan	2017-2021	-4.562	-4.637	-4.488	<0.001
VCT: Saint Vincent and the Grenadines	2000-2006	-0.316	-0.349	-0.283	<0.001
VCT: Saint Vincent and the Grenadines	2006-2009	-1.198	-1.391	-1.004	<0.001
VCT: Saint Vincent and the Grenadines	2009-2012	-2.655	-2.846	-2.464	<0.001
VCT: Saint Vincent and the Grenadines	2012-2021	-3.19	-3.208	-3.173	<0.001
VEN: Venezuela	2000-2008	-2.876	-2.993	-2.759	<0.001
VEN: Venezuela	2008-2014	0.798	0.55	1.047	<0.001
VEN: Venezuela	2014-2017	12.36	11.128	13.606	<0.001
VEN: Venezuela	2017-2021	-0.91	-1.255	-0.563	<0.001
VGB: British Virgin Islands	2000-2007	-0.238	-0.312	-0.163	<0.001
VGB: British Virgin Islands	2007-2010	-1.515	-2.065	-0.963	<0.001
VGB: British Virgin Islands	2010-2015	-2.567	-2.739	-2.395	<0.001
VGB: British Virgin Islands	2015-2021	-3.087	-3.179	-2.996	<0.001
VNM: Vietnam	2000-2002	-3.782	-4.01	-3.554	<0.001
VNM: Vietnam	2002-2005	-2.975	-3.205	-2.745	<0.001
VNM: Vietnam	2005-2008	-1.833	-2.065	-1.6	<0.001
VNM: Vietnam	2008-2011	-1.199	-1.432	-0.964	<0.001
VNM: Vietnam	2011-2021	-1.008	-1.026	-0.989	<0.001
VUT: Vanuatu	2000-2003	-0.666	-0.847	-0.484	<0.001
VUT: Vanuatu	2003-2012	0.187	0.148	0.227	<0.001
VUT: Vanuatu	2012-2015	-1.478	-1.837	-1.118	<0.001
VUT: Vanuatu	2015-2019	-2.34	-2.518	-2.161	<0.001
VUT: Vanuatu	2019-2021	-2.828	-3.182	-2.473	<0.001
WSM: Samoa	2000-2021	-0.799	-1.049	-0.547	<0.001
XKX: Kosovo	2000-2006	-8.531	-8.566	-8.495	<0.001
XKX: Kosovo	2006-2009	-7.879	-8.09	-7.667	<0.001
XKX: Kosovo	2009-2012	-7.149	-7.361	-6.935	<0.001
XKX: Kosovo	2012-2019	-6.084	-6.12	-6.047	<0.001
XKX: Kosovo	2019-2021	-5.267	-5.484	-5.049	<0.001
YEM: Yemen	2000-2007	-4.5	-4.718	-4.281	<0.001
YEM: Yemen	2007-2010	-3.266	-4.907	-1.597	0.001

YEM: Yemen	2010-2021	0.256	0.14	0.371	<0.001
ZAF: South Africa	2000-2004	0.673	0.404	0.942	<0.001
ZAF: South Africa	2004-2007	-1.395	-2.224	-0.559	0.005
ZAF: South Africa	2007-2011	-9.114	-9.497	-8.73	<0.001
ZAF: South Africa	2011-2015	-2.847	-3.256	-2.436	<0.001
ZAF: South Africa	2015-2021	-1.736	-1.876	-1.595	<0.001
ZMB: Zambia	2000-2006	-7.142	-7.594	-6.689	<0.001
ZMB: Zambia	2006-2009	-3.457	-6.2	-0.634	0.020
ZMB: Zambia	2009-2021	-2.185	-2.351	-2.018	<0.001
ZWE: Zimbabwe	2000-2003	-0.339	-1.967	1.316	0.647
ZWE: Zimbabwe	2003-2007	2.623	0.947	4.327	0.007
ZWE: Zimbabwe	2007-2010	-2.234	-5.401	1.039	0.152
ZWE: Zimbabwe	2010-2014	-5.382	-6.928	-3.812	<0.001
ZWE: Zimbabwe	2014-2021	-2.687	-3.114	-2.258	<0.001

Figure S1. Pairwise comparisons of temporal trend in stillbirth rates across UNICEF reporting regions using joinpoint regression

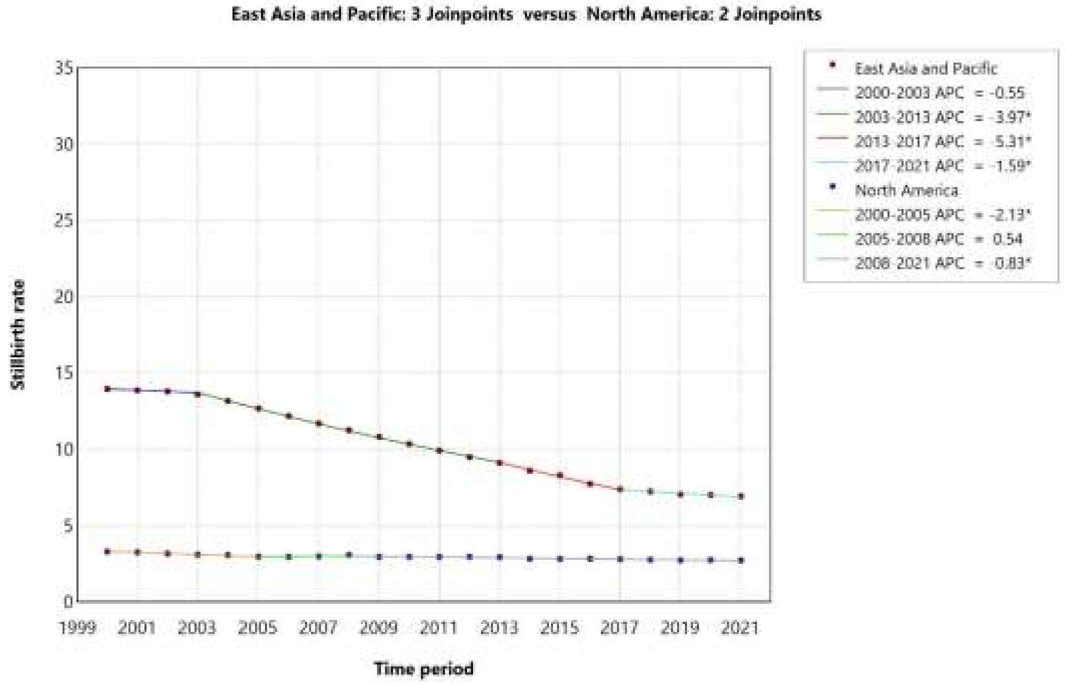

* Indicates that the Annual Percent Change (APC) is significantly different from zero at the alpha = 0.05 level.
Final Selected Model: East Asia and Pacific - 3 Joinpoints, North America - 2 Joinpoints. Rejected Parallelism.

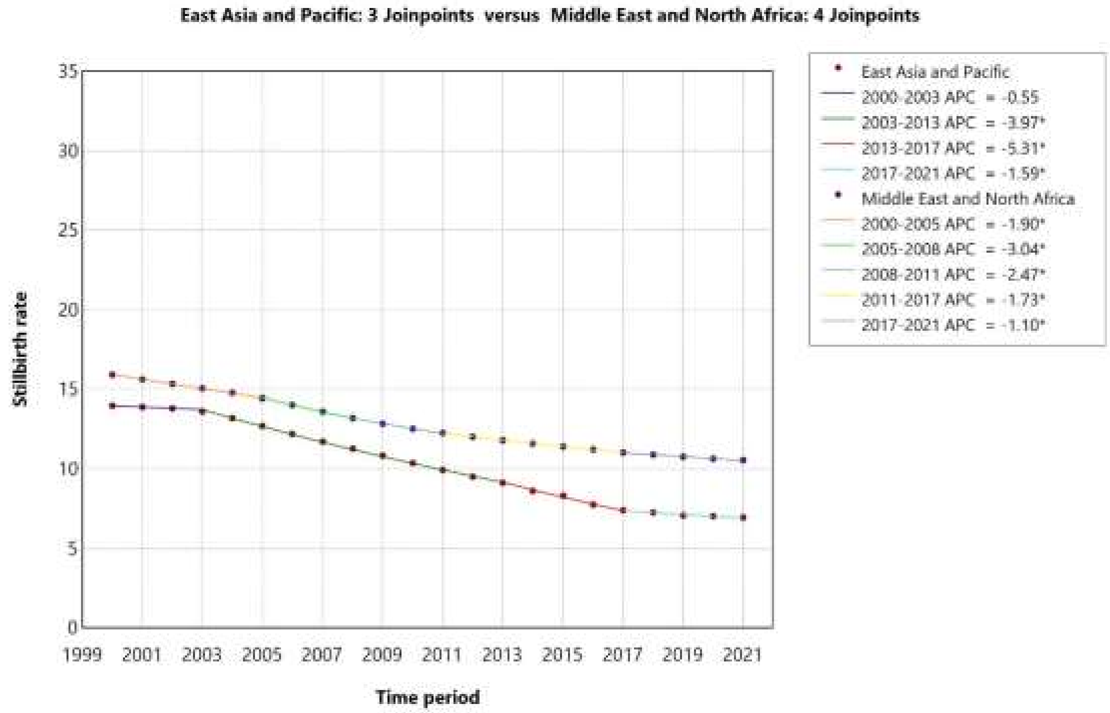

* Indicates that the Annual Percent Change (APC) is significantly different from zero at the alpha = 0.05 level.
Final Selected Model: East Asia and Pacific - 3 Joinpoints, Middle East and North Africa - 4 Joinpoints. Rejected Parallelism.

East Asia and Pacific: 3 Joinpoints versus Latin America and the Caribbean: 1 Joinpoint

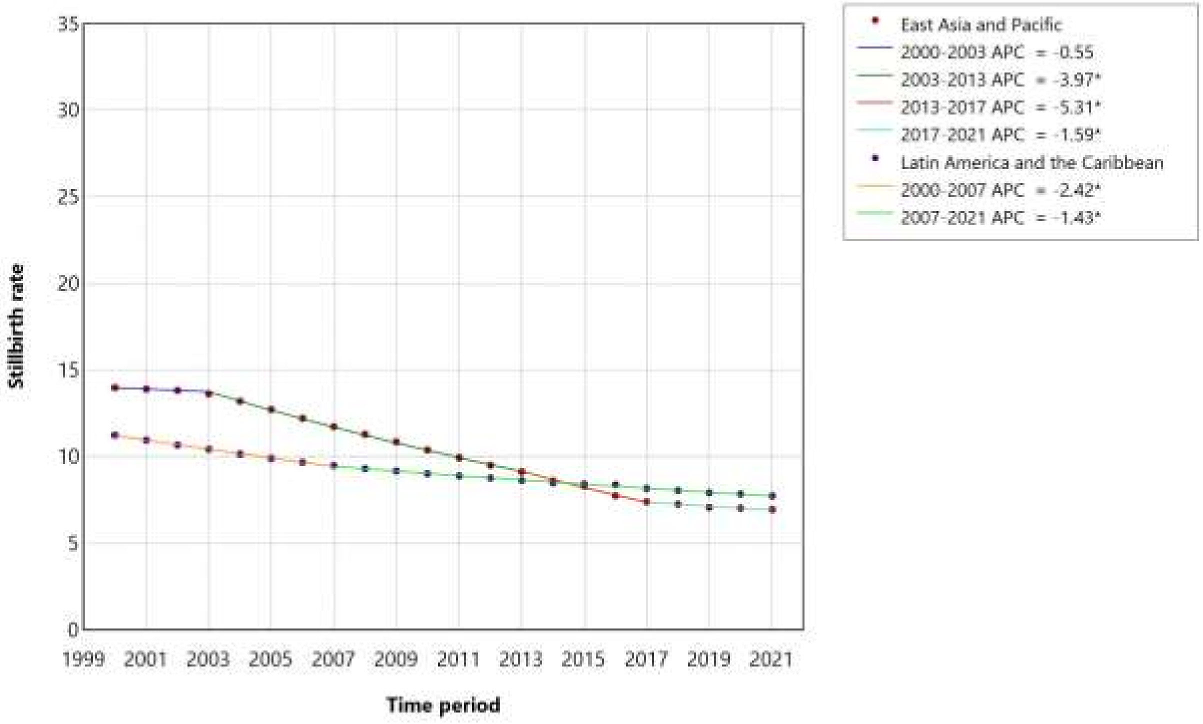

* Indicates that the Annual Percent Change (APC) is significantly different from zero at the alpha = 0.05 level.
Final Selected Model: East Asia and Pacific - 3 Joinpoints, Latin America and the Caribbean - 1 Joinpoint. Rejected Parallelism.

East Asia and Pacific: 3 Joinpoints versus Europe and Central Asia: 2 Joinpoints

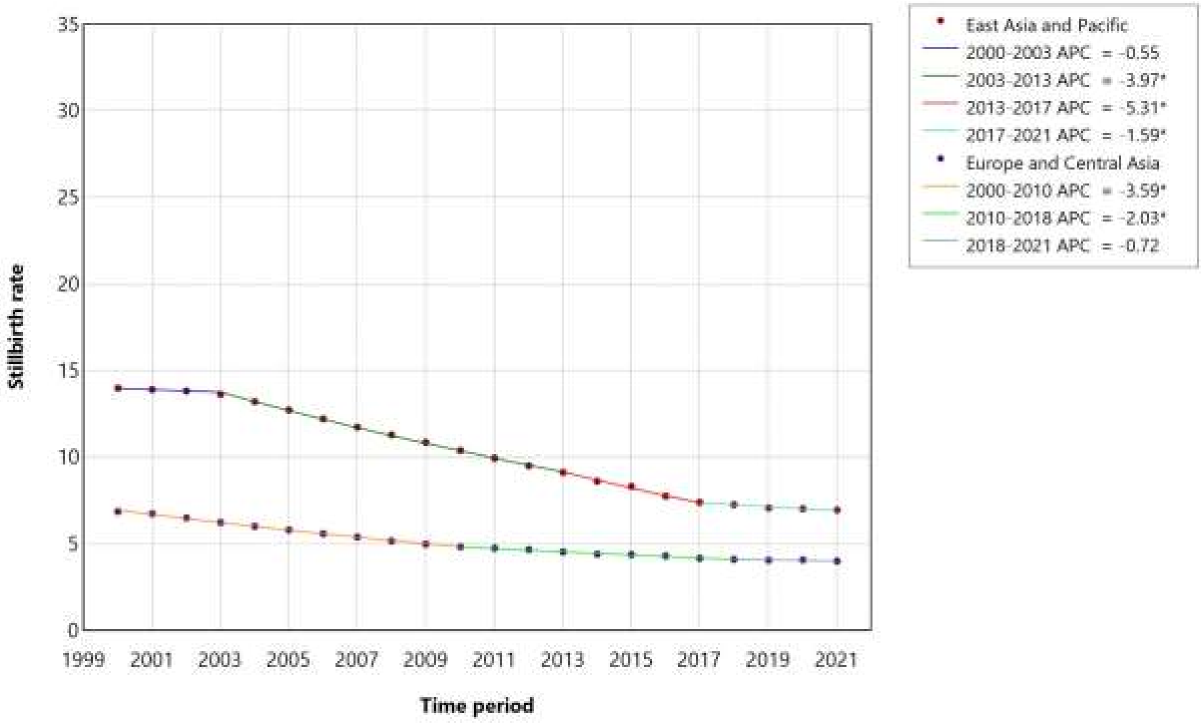

* Indicates that the Annual Percent Change (APC) is significantly different from zero at the alpha = 0.05 level.
Final Selected Model: East Asia and Pacific - 3 Joinpoints, Europe and Central Asia - 2 Joinpoints. Rejected Parallelism.

East Asia and Pacific: 3 Joinpoints versus Eastern Europe and Central Asia: 4 Joinpoints

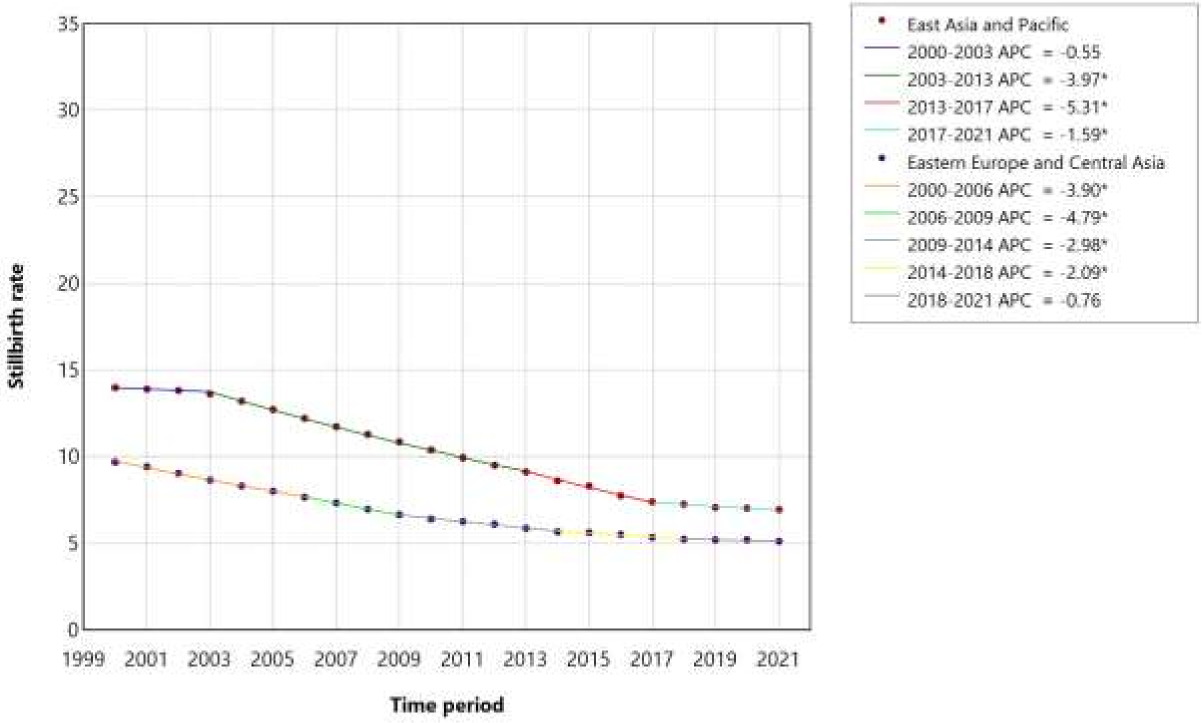

* Indicates that the Annual Percent Change (APC) is significantly different from zero at the alpha = 0.05 level.
Final Selected Model: East Asia and Pacific - 3 Joinpoints, Eastern Europe and Central Asia - 4 Joinpoints. Rejected Parallelism.

East Asia and Pacific: 3 Joinpoints versus Eastern and Southern Africa: 3 Joinpoints

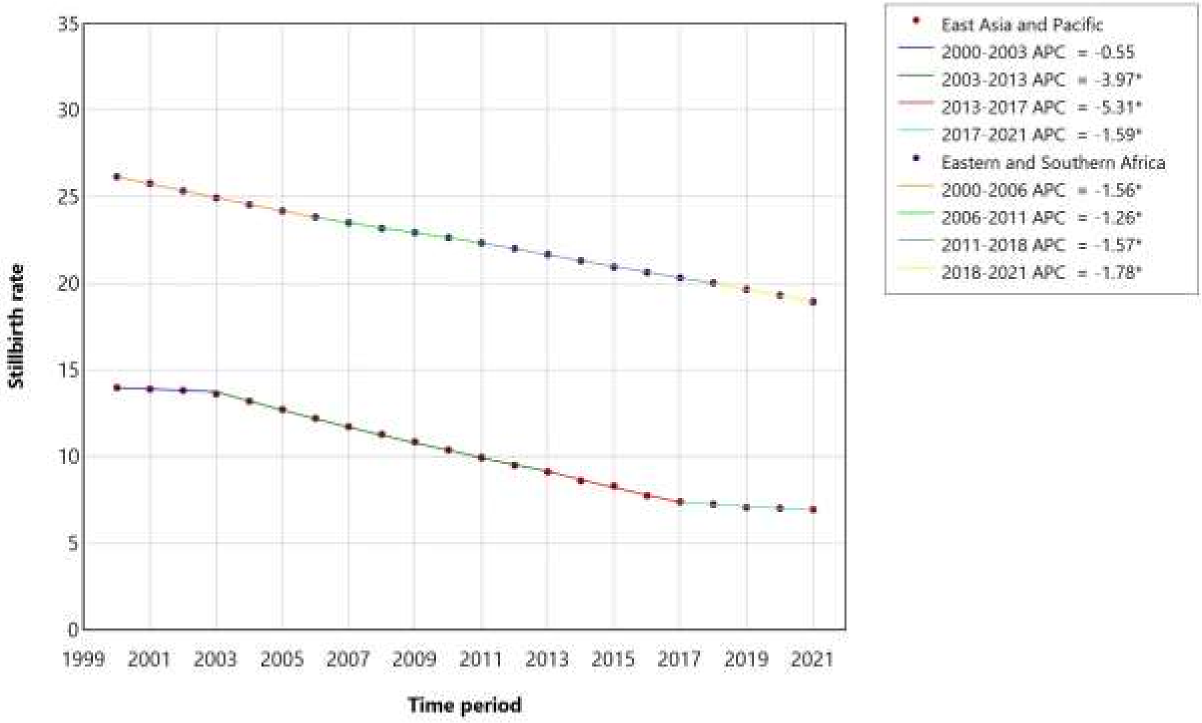

* Indicates that the Annual Percent Change (APC) is significantly different from zero at the alpha = 0.05 level.
Final Selected Model: East Asia and Pacific - 3 Joinpoints, Eastern and Southern Africa - 3 Joinpoints. Rejected Parallelism.

West and Central Africa: 5 Joinpoints versus Western Europe: 4 Joinpoints

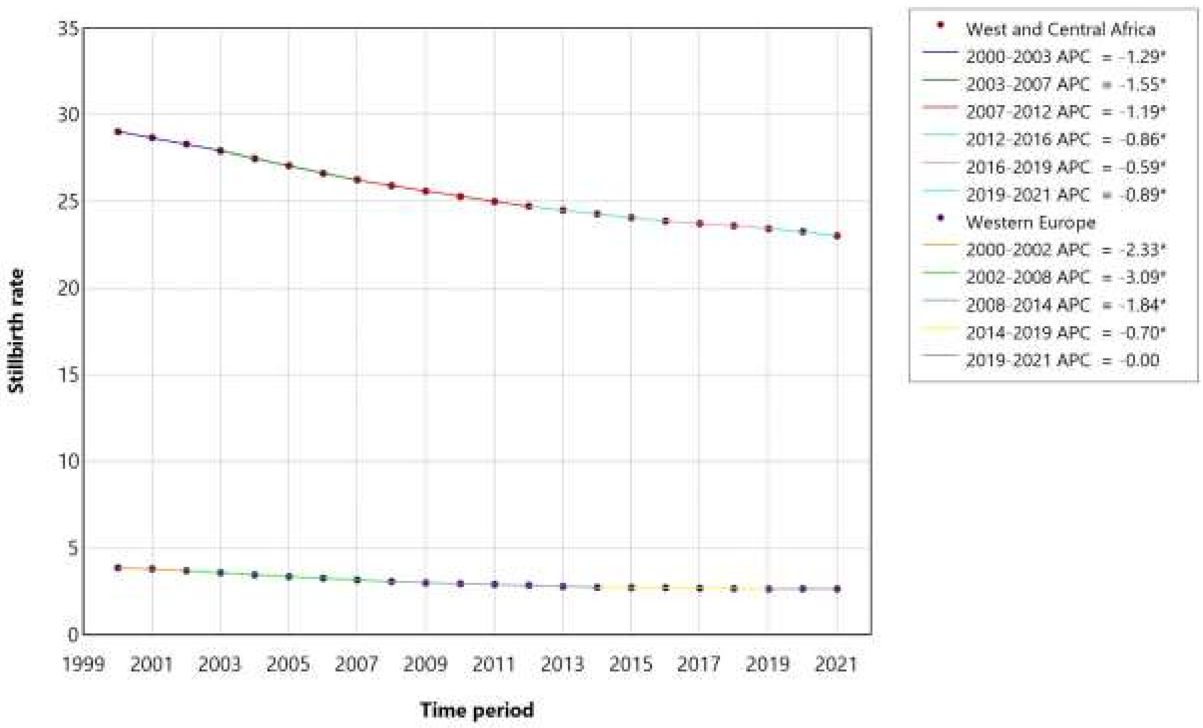

* Indicates that the Annual Percent Change (APC) is significantly different from zero at the alpha = 0.05 level.
Final Selected Model: West and Central Africa - 5 Joinpoints, Western Europe - 4 Joinpoints. Rejected Parallelism.

Sub-Saharan Africa: 3 Joinpoints versus Western Europe: 4 Joinpoints

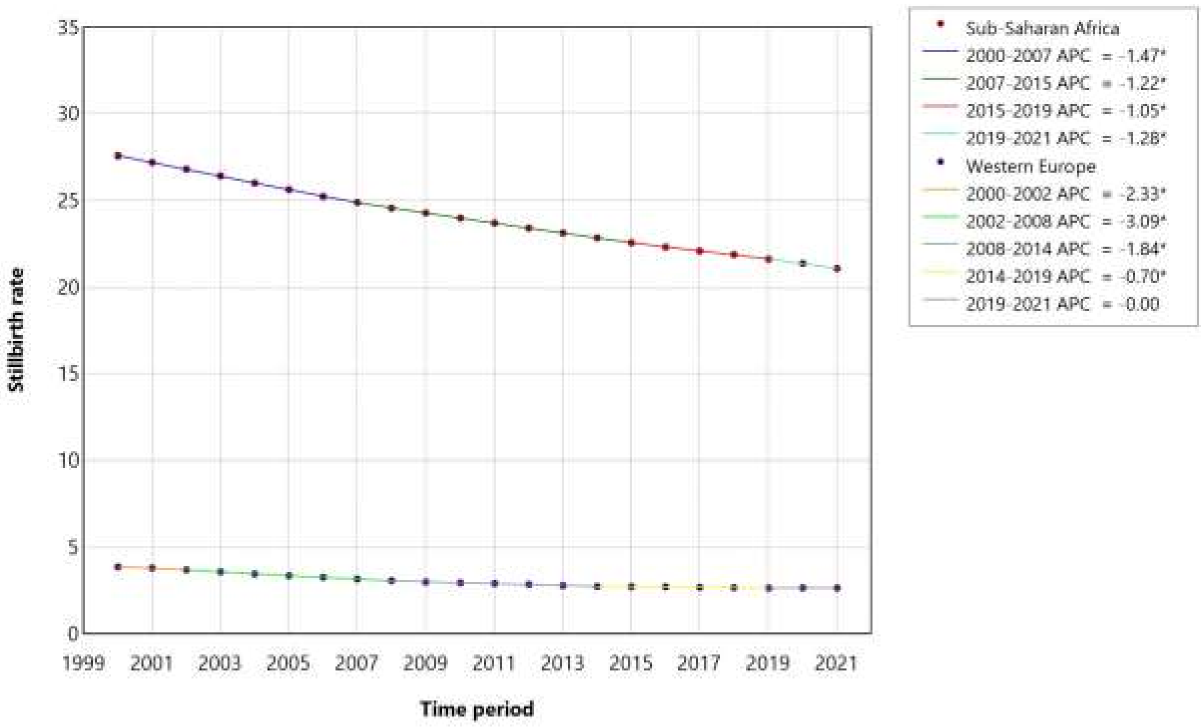

* Indicates that the Annual Percent Change (APC) is significantly different from zero at the alpha = 0.05 level.
Final Selected Model: Sub-Saharan Africa - 3 Joinpoints, Western Europe - 4 Joinpoints. Rejected Parallelism.

Sub-Saharan Africa: 3 Joinpoints versus West and Central Africa: 5 Joinpoints

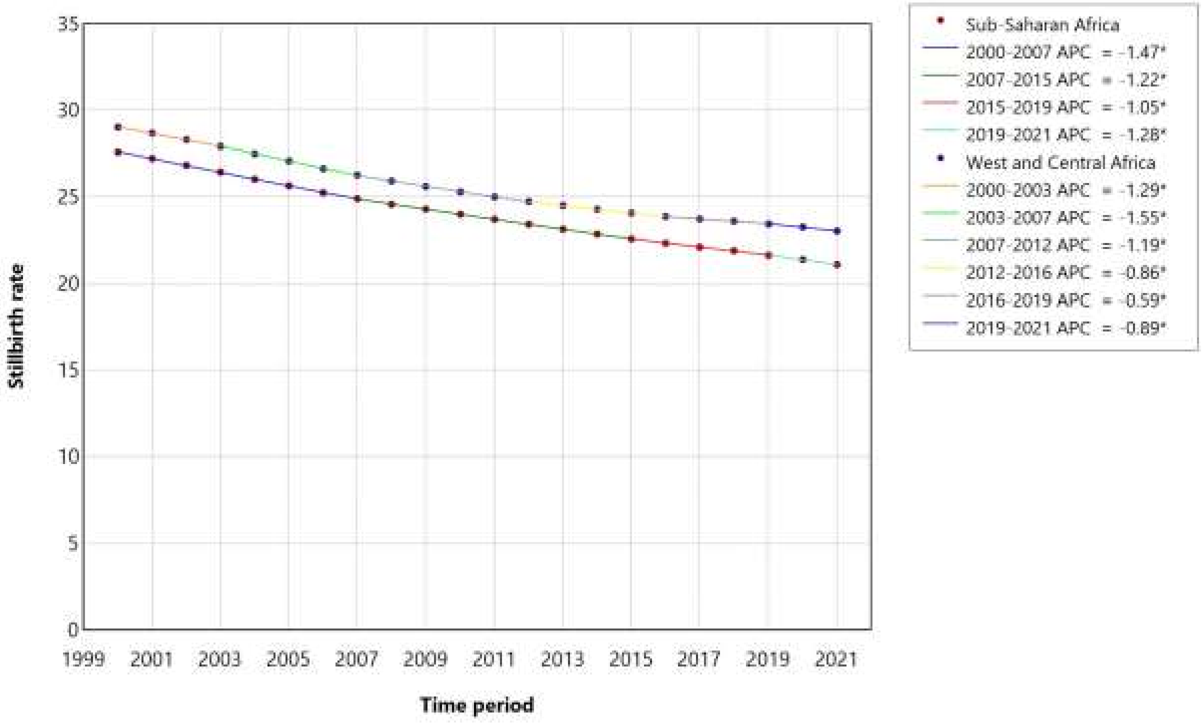

* Indicates that the Annual Percent Change (APC) is significantly different from zero at the alpha = 0.05 level.
Final Selected Model: Sub-Saharan Africa - 3 Joinpoints, West and Central Africa - 5 Joinpoints. Rejected Parallelism.

South Asia: 5 Joinpoints versus Western Europe: 4 Joinpoints

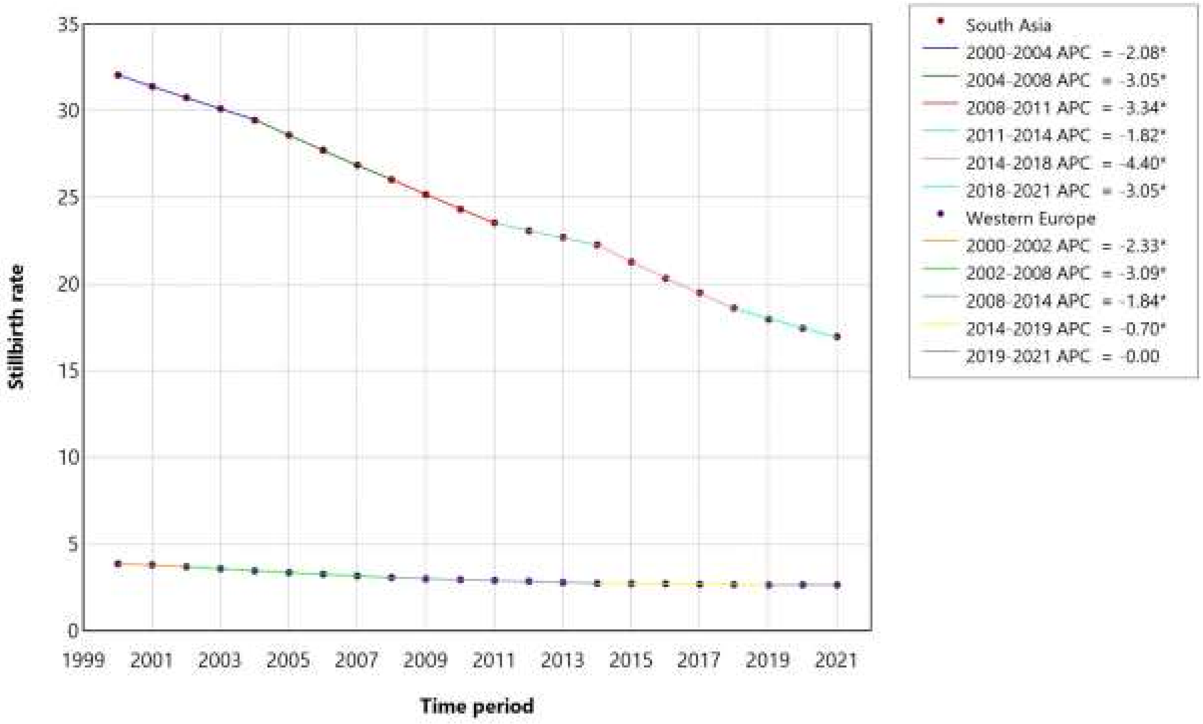

* Indicates that the Annual Percent Change (APC) is significantly different from zero at the alpha = 0.05 level.
Final Selected Model: South Asia - 5 Joinpoints, Western Europe - 4 Joinpoints. Rejected Parallelism.

South Asia: 5 Joinpoints versus West and Central Africa: 5 Joinpoints

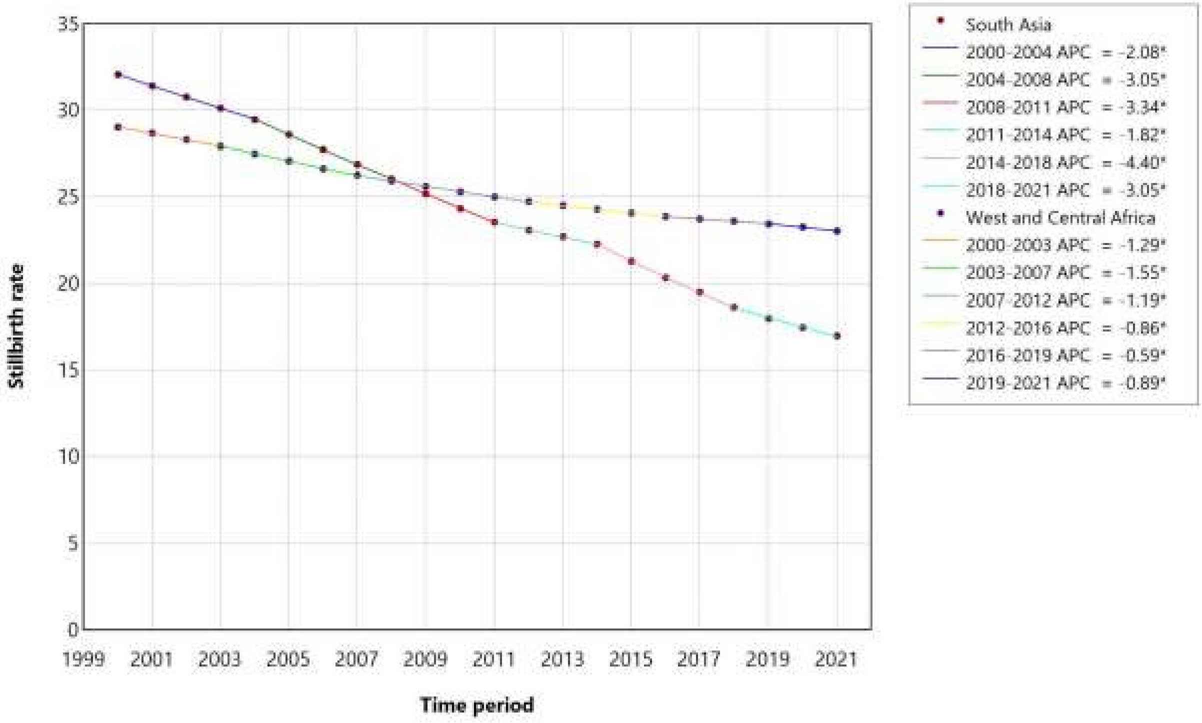

* Indicates that the Annual Percent Change (APC) is significantly different from zero at the alpha = 0.05 level.
Final Selected Model: South Asia - 5 Joinpoints, West and Central Africa - 5 Joinpoints. Rejected Parallelism.

South Asia: 5 Joinpoints versus Sub-Saharan Africa: 3 Joinpoints

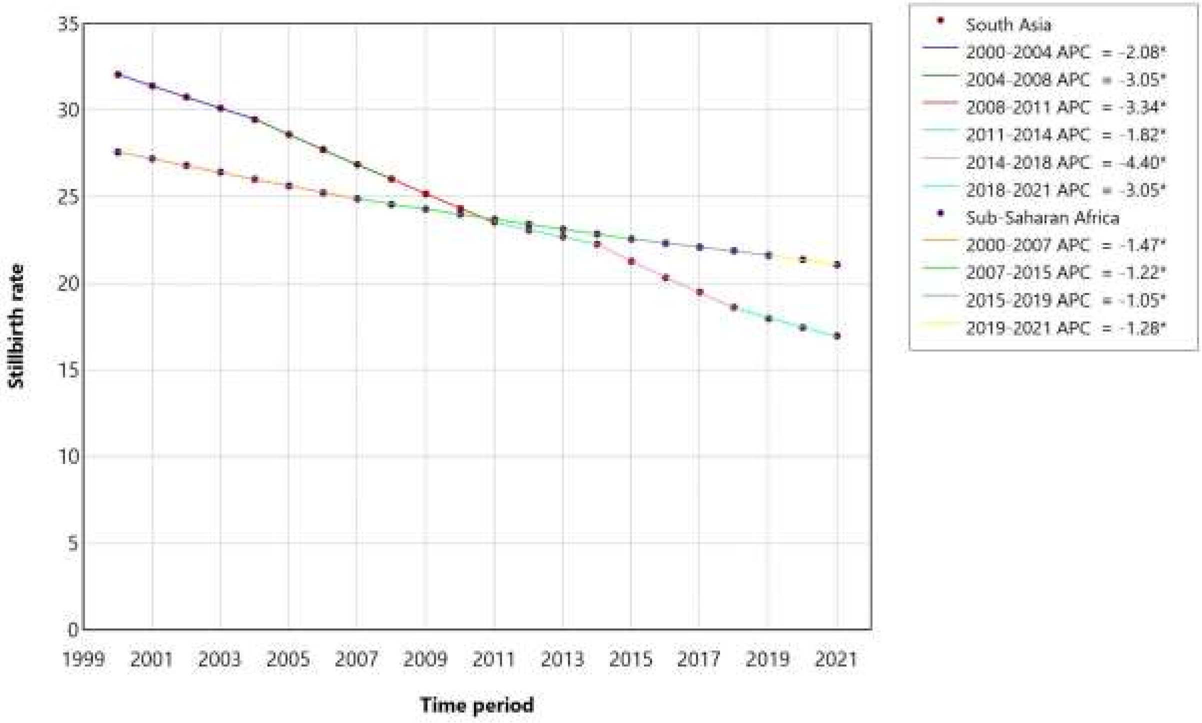

* Indicates that the Annual Percent Change (APC) is significantly different from zero at the alpha = 0.05 level.
Final Selected Model: South Asia - 5 Joinpoints, Sub-Saharan Africa - 3 Joinpoints. Rejected Parallelism.

North America: 2 Joinpoints versus Western Europe: 4 Joinpoints

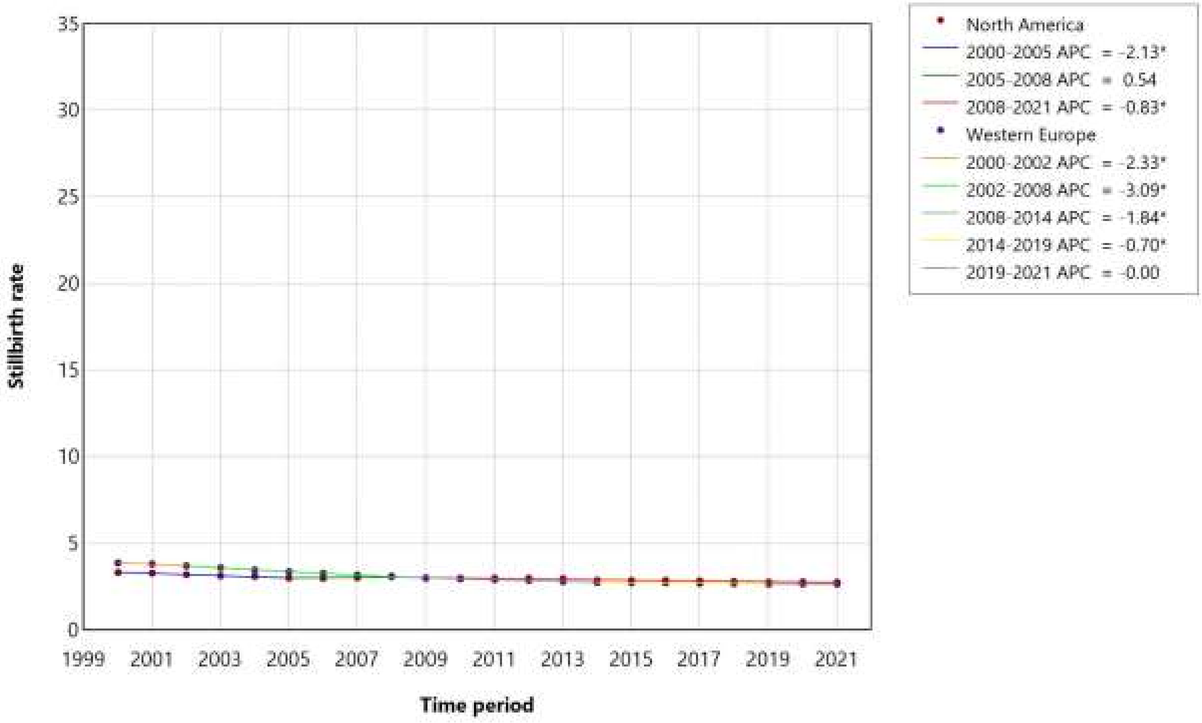

* Indicates that the Annual Percent Change (APC) is significantly different from zero at the alpha = 0.05 level.
Final Selected Model: North America - 2 Joinpoints, Western Europe - 4 Joinpoints. Rejected Parallelism.

North America: 2 Joinpoints versus West and Central Africa: 5 Joinpoints

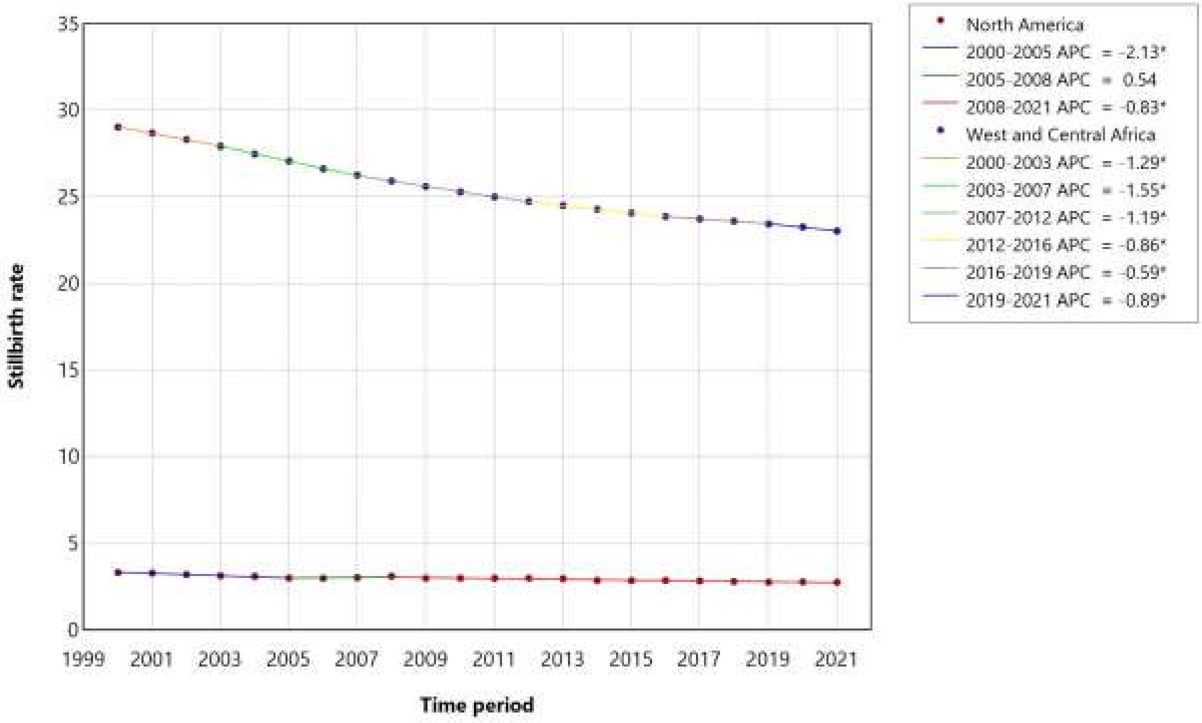

* Indicates that the Annual Percent Change (APC) is significantly different from zero at the alpha = 0.05 level.
Final Selected Model: North America - 2 Joinpoints, West and Central Africa - 5 Joinpoints. Rejected Parallelism.

North America: 2 Joinpoints versus Sub-Saharan Africa: 3 Joinpoints

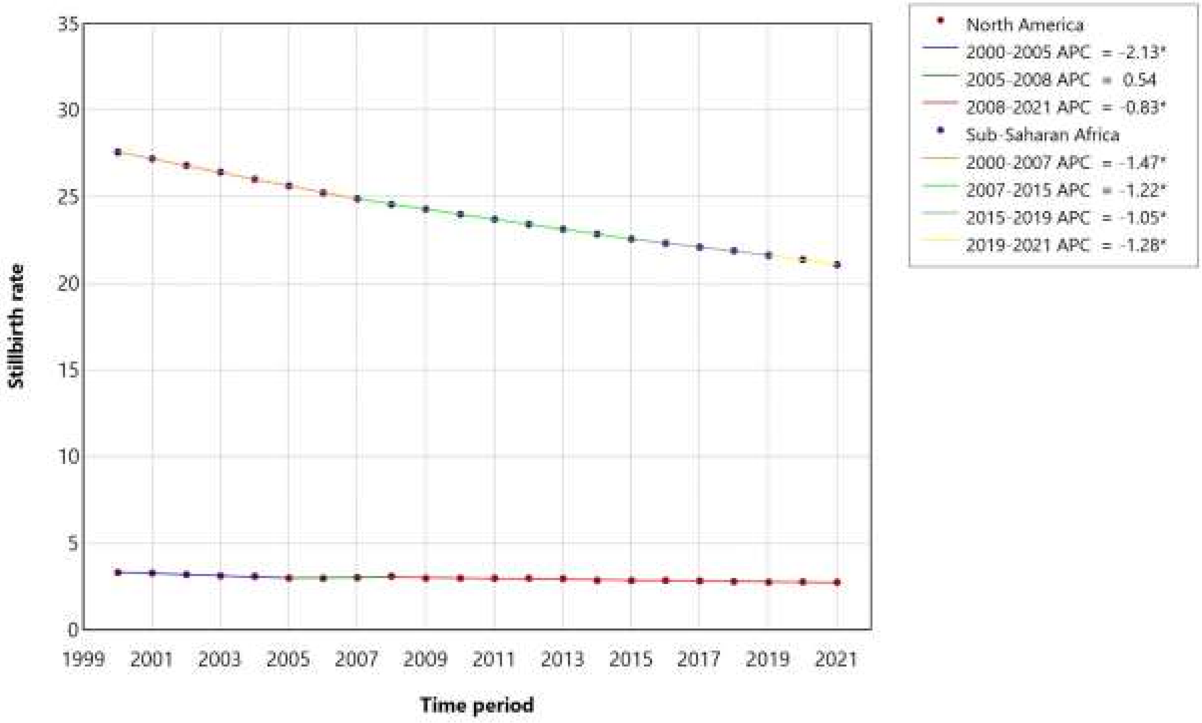

* Indicates that the Annual Percent Change (APC) is significantly different from zero at the alpha = 0.05 level.
Final Selected Model: North America - 2 Joinpoints, Sub-Saharan Africa - 3 Joinpoints. Rejected Parallelism.

North America: 2 Joinpoints versus South Asia: 5 Joinpoints

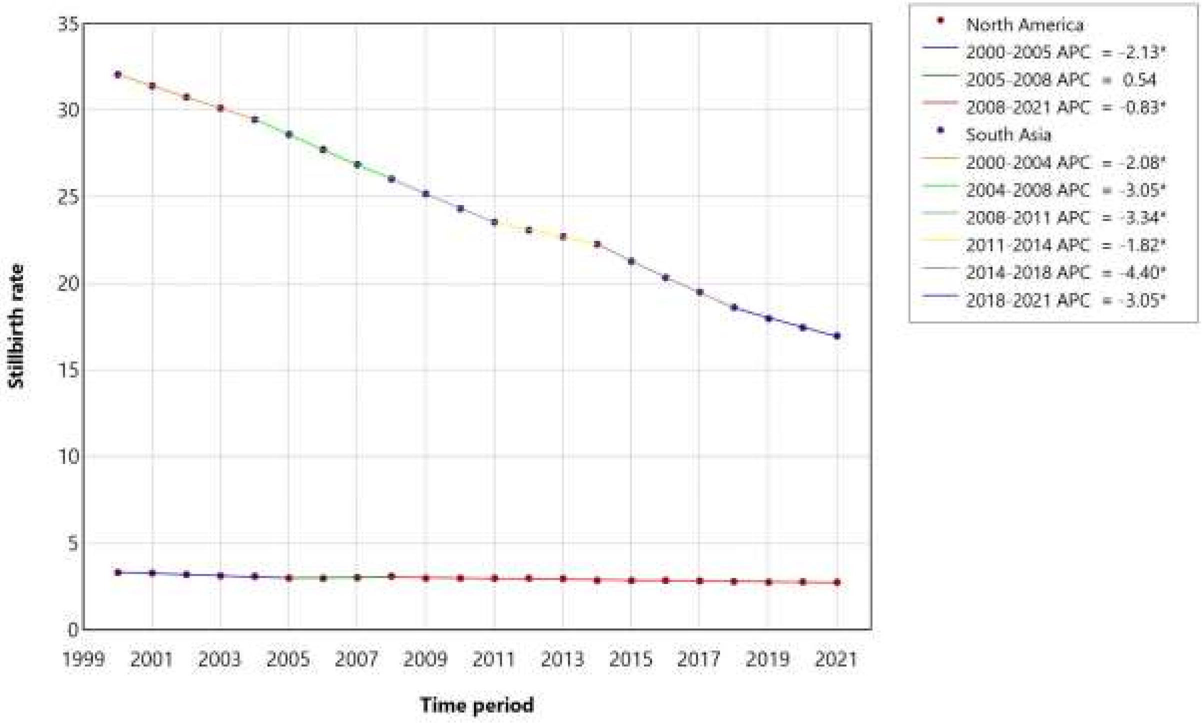

* Indicates that the Annual Percent Change (APC) is significantly different from zero at the alpha = 0.05 level.
Final Selected Model: North America - 2 Joinpoints, South Asia - 5 Joinpoints. Rejected Parallelism.

Middle East and North Africa: 4 Joinpoints versus Western Europe: 4 Joinpoints

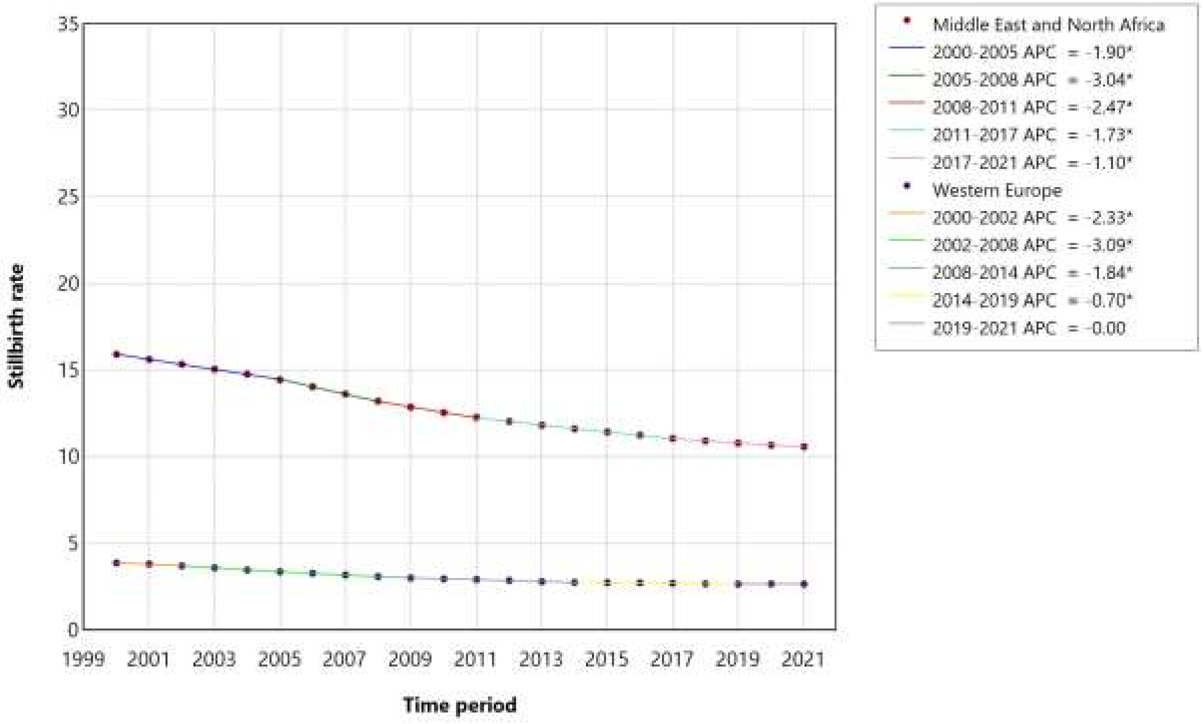

* Indicates that the Annual Percent Change (APC) is significantly different from zero at the alpha = 0.05 level.
Final Selected Model: Middle East and North Africa - 4 Joinpoints, Western Europe - 4 Joinpoints. Rejected Parallelism.

Middle East and North Africa: 4 Joinpoints versus West and Central Africa: 5 Joinpoints

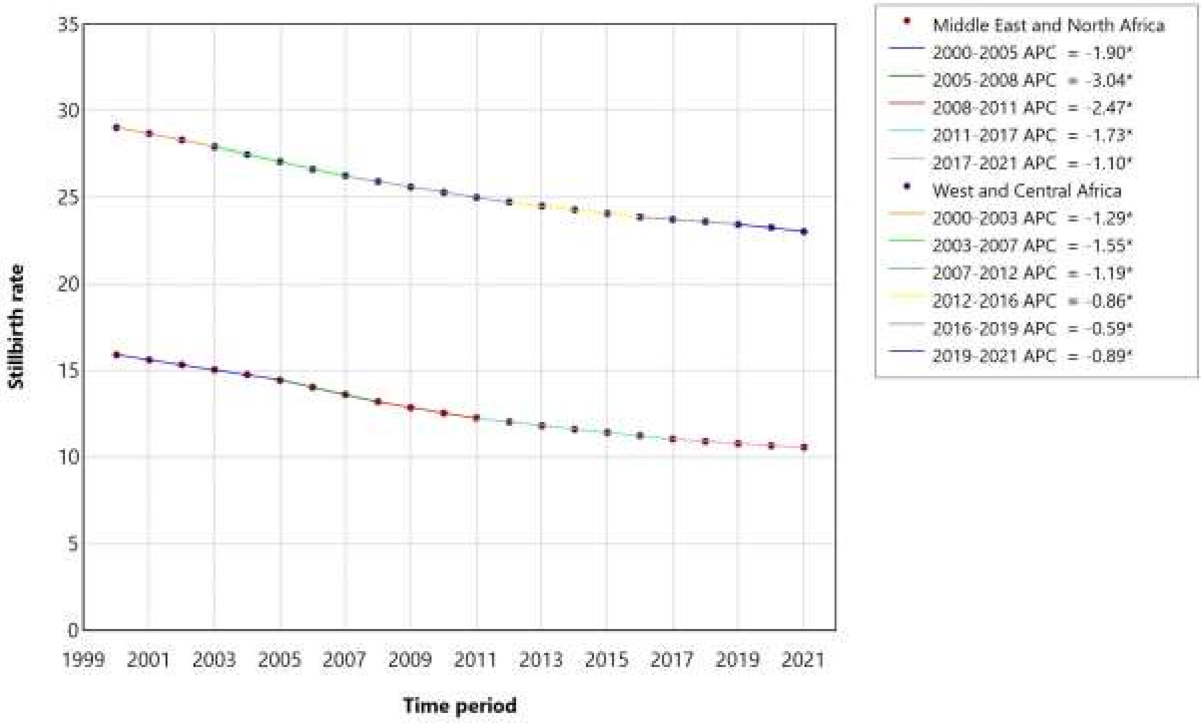

* Indicates that the Annual Percent Change (APC) is significantly different from zero at the alpha = 0.05 level.
Final Selected Model: Middle East and North Africa - 4 Joinpoints, West and Central Africa - 5 Joinpoints. Rejected Parallelism.

Middle East and North Africa: 4 Joinpoints versus Sub-Saharan Africa: 3 Joinpoints

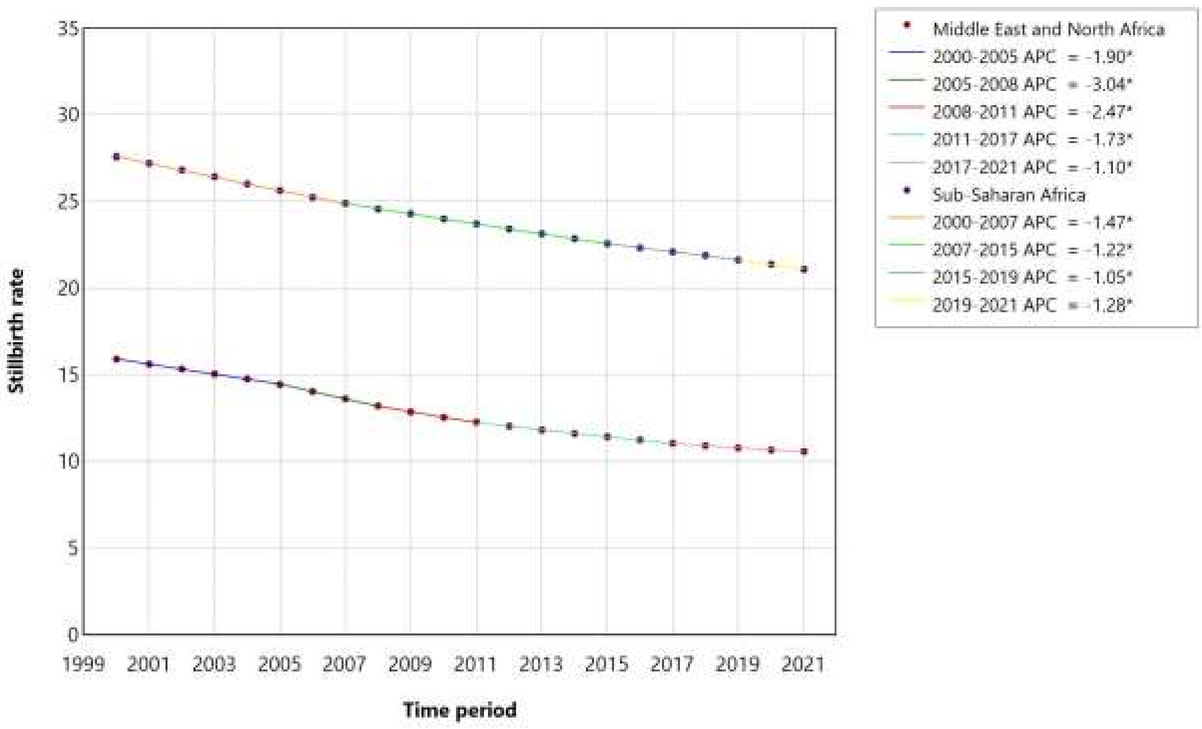

* Indicates that the Annual Percent Change (APC) is significantly different from zero at the alpha = 0.05 level.
Final Selected Model: Middle East and North Africa - 4 Joinpoints, Sub-Saharan Africa - 3 Joinpoints. Rejected Parallelism.

Middle East and North Africa: 4 Joinpoints versus South Asia: 5 Joinpoints

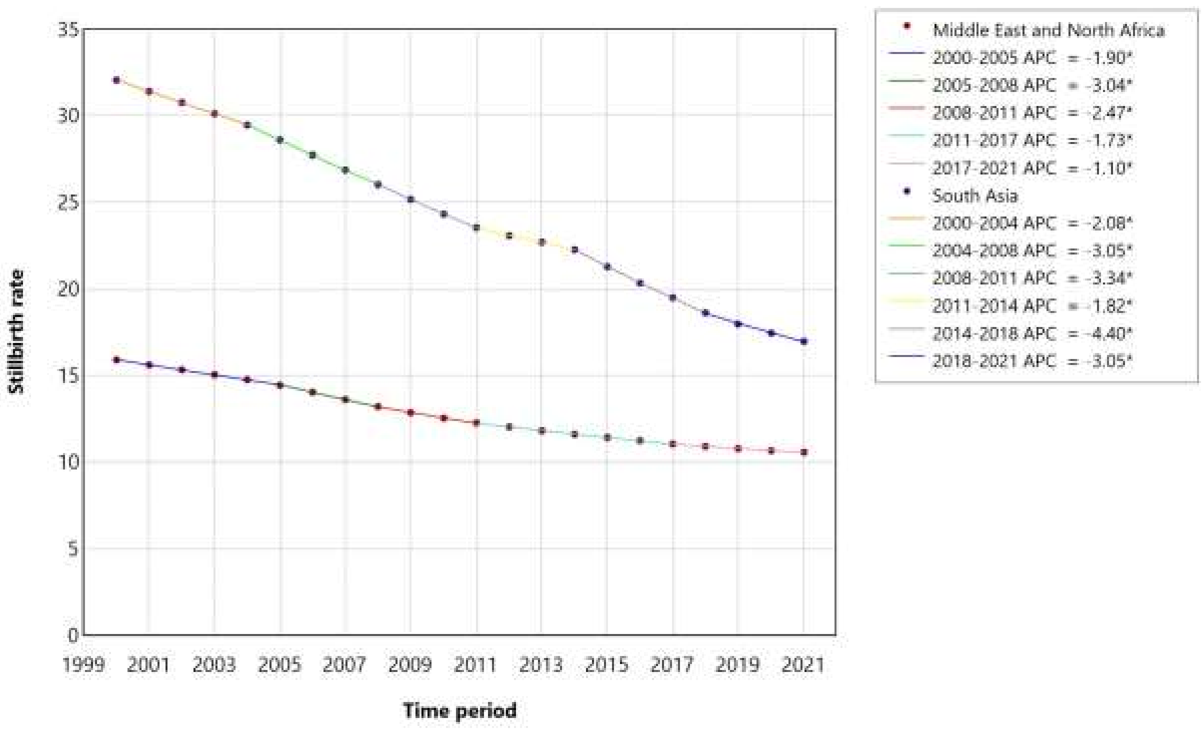

* Indicates that the Annual Percent Change (APC) is significantly different from zero at the alpha = 0.05 level.
Final Selected Model: Middle East and North Africa - 4 Joinpoints, South Asia - 5 Joinpoints. Rejected Parallelism.

Middle East and North Africa: 4 Joinpoints versus North America: 2 Joinpoints

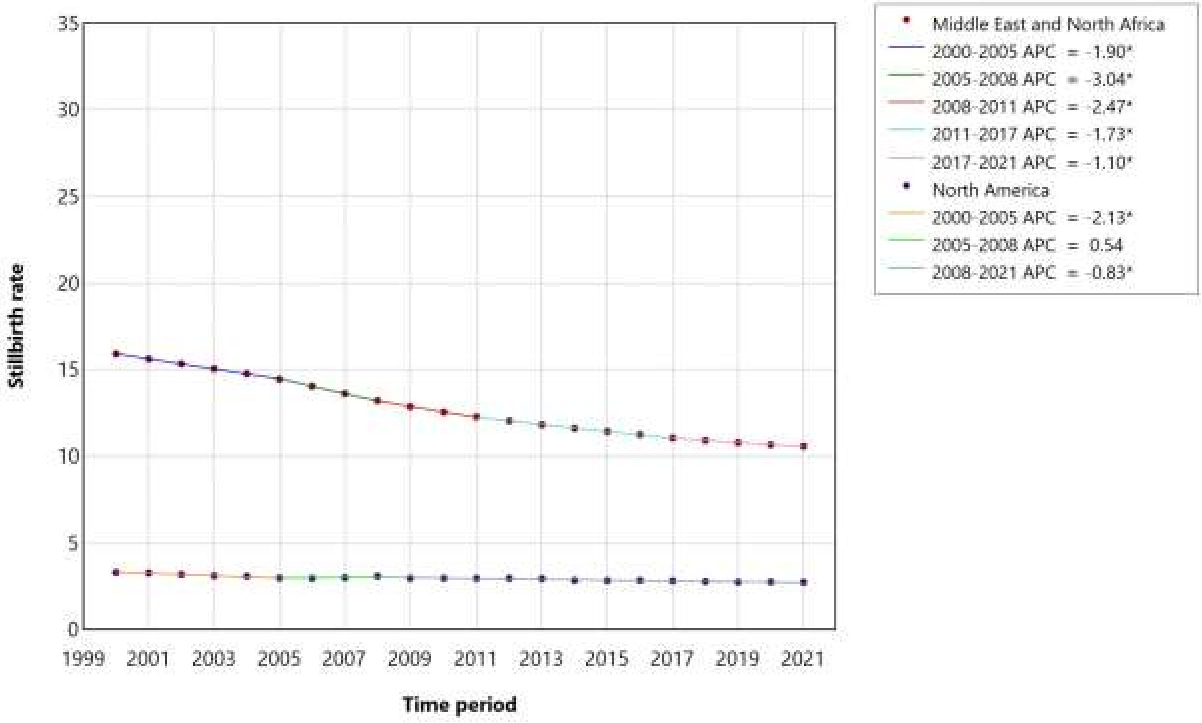

* Indicates that the Annual Percent Change (APC) is significantly different from zero at the alpha = 0.05 level.
Final Selected Model: Middle East and North Africa - 4 Joinpoints, North America - 2 Joinpoints. Rejected Parallelism.

Latin America and the Caribbean: 1 Joinpoint versus Western Europe: 4 Joinpoints

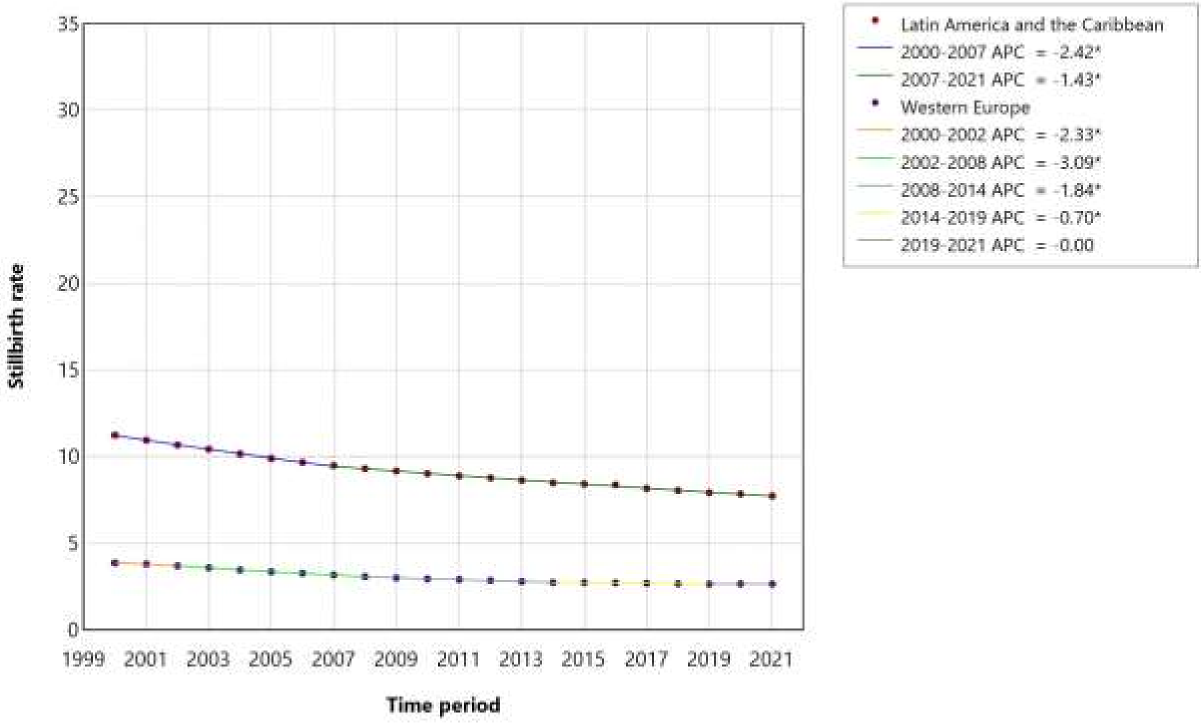

* Indicates that the Annual Percent Change (APC) is significantly different from zero at the alpha = 0.05 level.
Final Selected Model: Latin America and the Caribbean - 1 Joinpoint, Western Europe - 4 Joinpoints. Rejected Parallelism.

Latin America and the Caribbean: 1 Joinpoint versus West and Central Africa: 5 Joinpoints

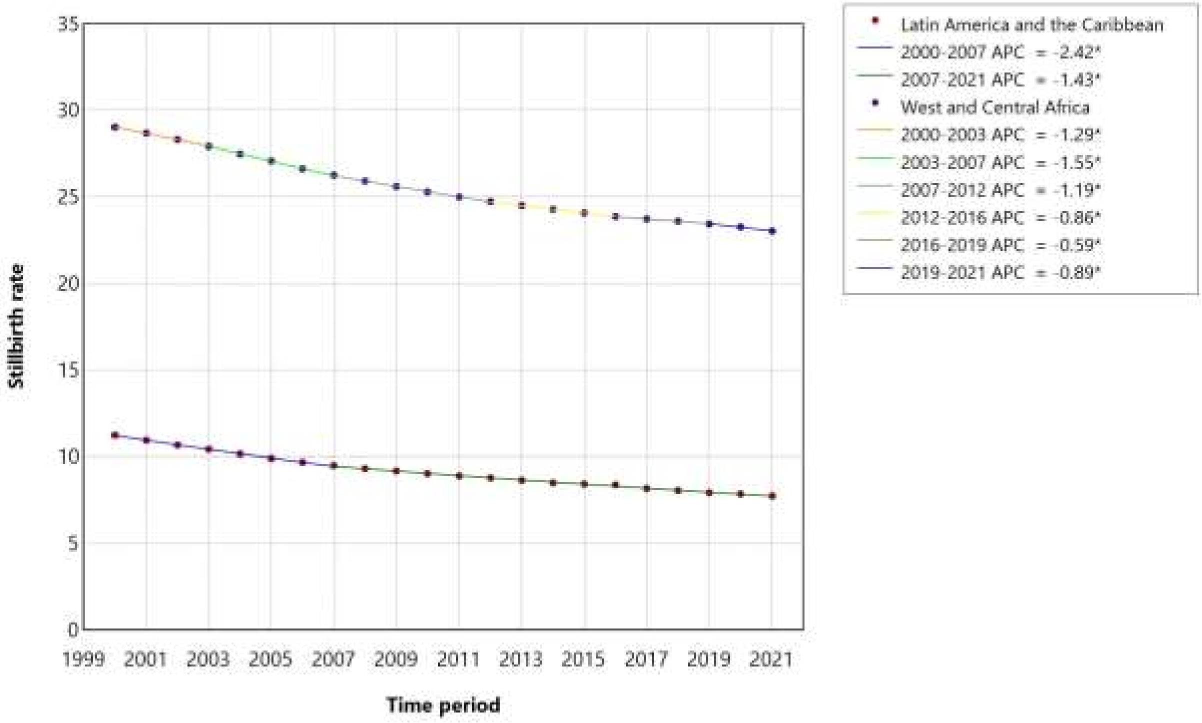

* Indicates that the Annual Percent Change (APC) is significantly different from zero at the alpha = 0.05 level.
Final Selected Model: Latin America and the Caribbean - 1 Joinpoint, West and Central Africa - 5 Joinpoints. Rejected Parallelism.

Latin America and the Caribbean: 1 Joinpoint versus Sub-Saharan Africa: 3 Joinpoints

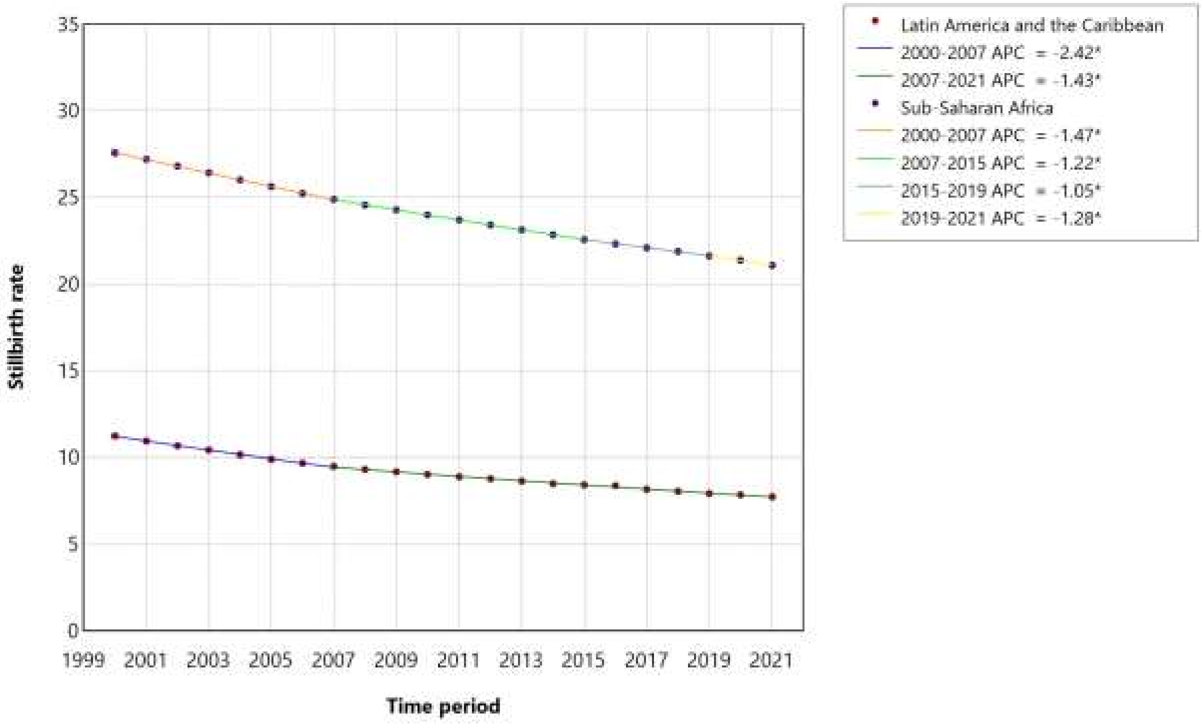

* Indicates that the Annual Percent Change (APC) is significantly different from zero at the alpha = 0.05 level.
Final Selected Model: Latin America and the Caribbean - 1 Joinpoint, Sub-Saharan Africa - 3 Joinpoints. Rejected Parallelism.

Latin America and the Caribbean: 1 Joinpoint versus South Asia: 5 Joinpoints

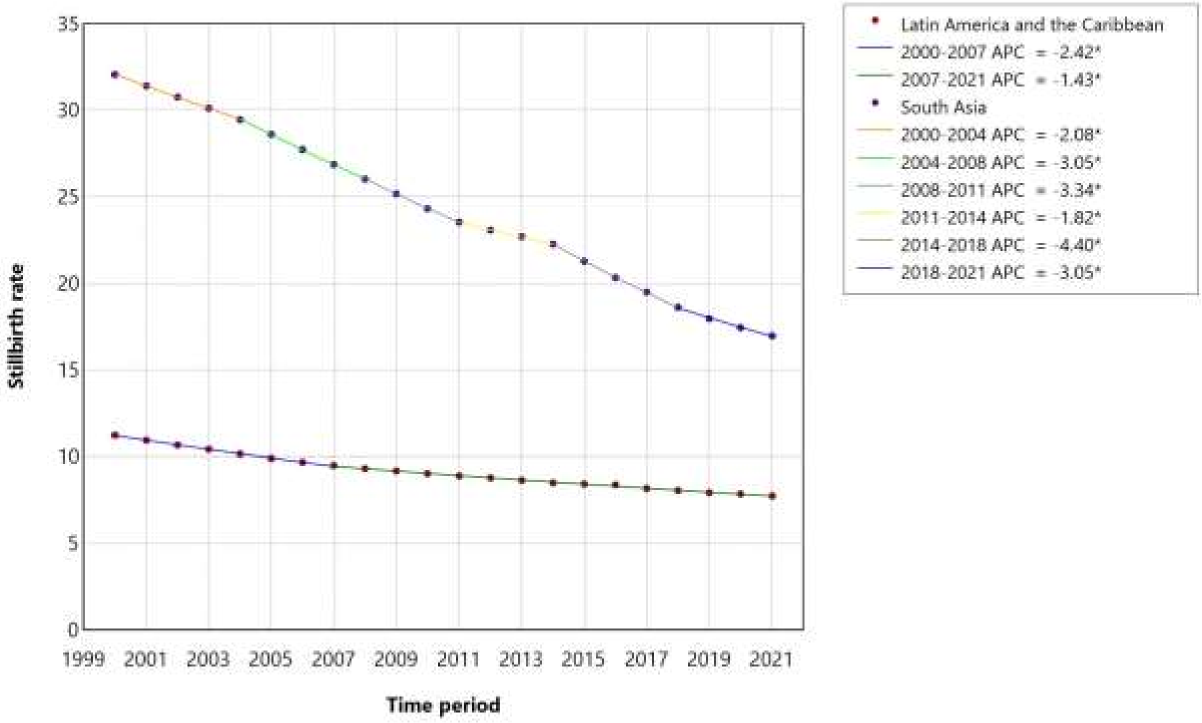

* Indicates that the Annual Percent Change (APC) is significantly different from zero at the alpha = 0.05 level.
Final Selected Model: Latin America and the Caribbean - 1 Joinpoint, South Asia - 5 Joinpoints. Rejected Parallelism.

Latin America and the Caribbean: 1 Joinpoint versus North America: 2 Joinpoints

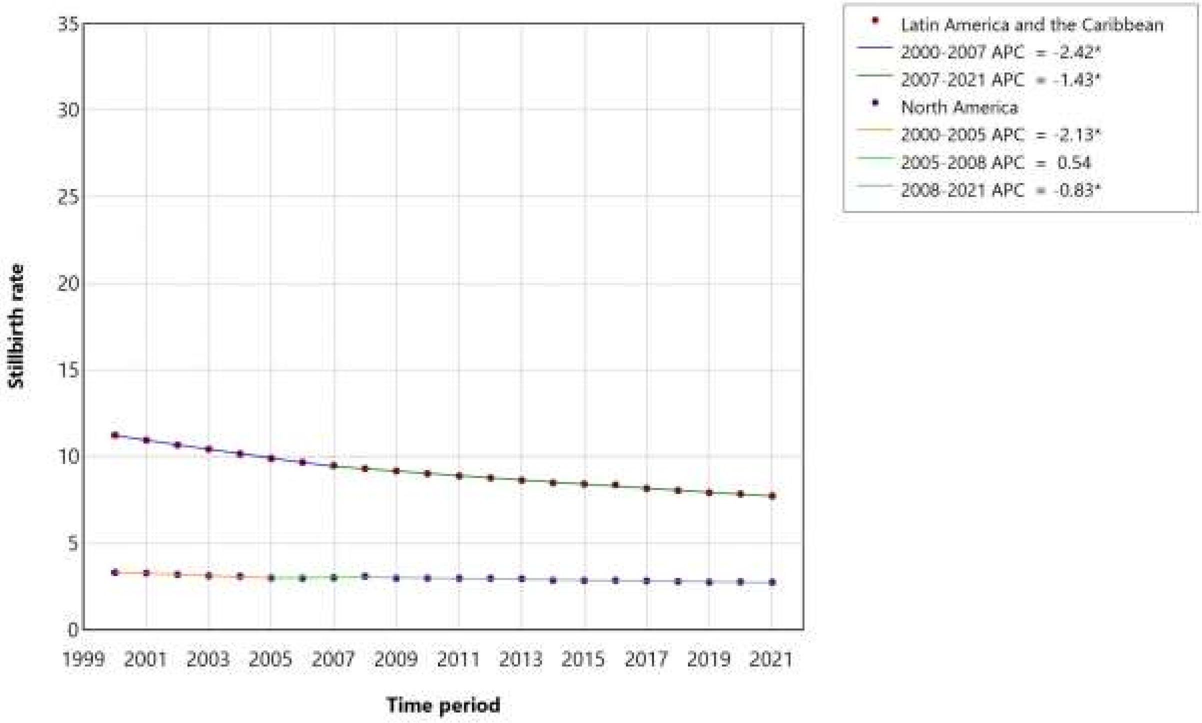

* Indicates that the Annual Percent Change (APC) is significantly different from zero at the alpha = 0.05 level.
Final Selected Model: Latin America and the Caribbean - 1 Joinpoint, North America - 2 Joinpoints. Rejected Parallelism.

Latin America and the Caribbean: 1 Joinpoint versus Middle East and North Africa: 4 Joinpoints

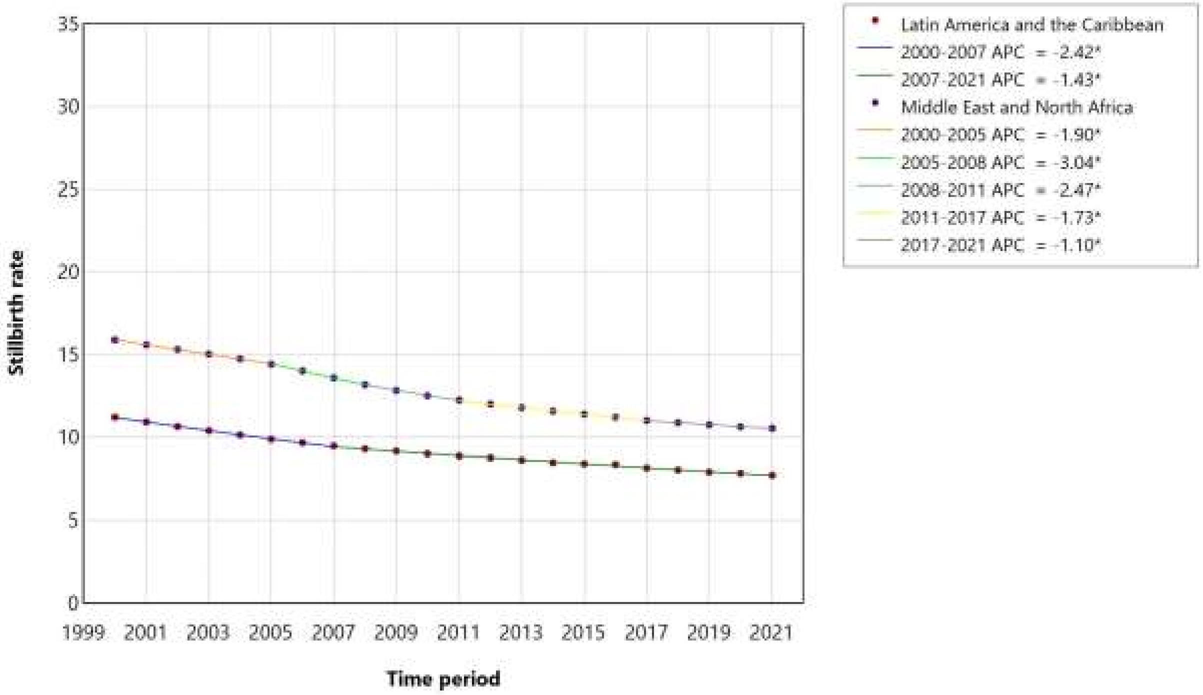

* Indicates that the Annual Percent Change (APC) is significantly different from zero at the alpha = 0.05 level.
Final Selected Model: Latin America and the Caribbean - 1 Joinpoint, Middle East and North Africa - 4 Joinpoints. Rejected Parallelism.

Europe and Central Asia: 2 Joinpoints versus Western Europe: 4 Joinpoints

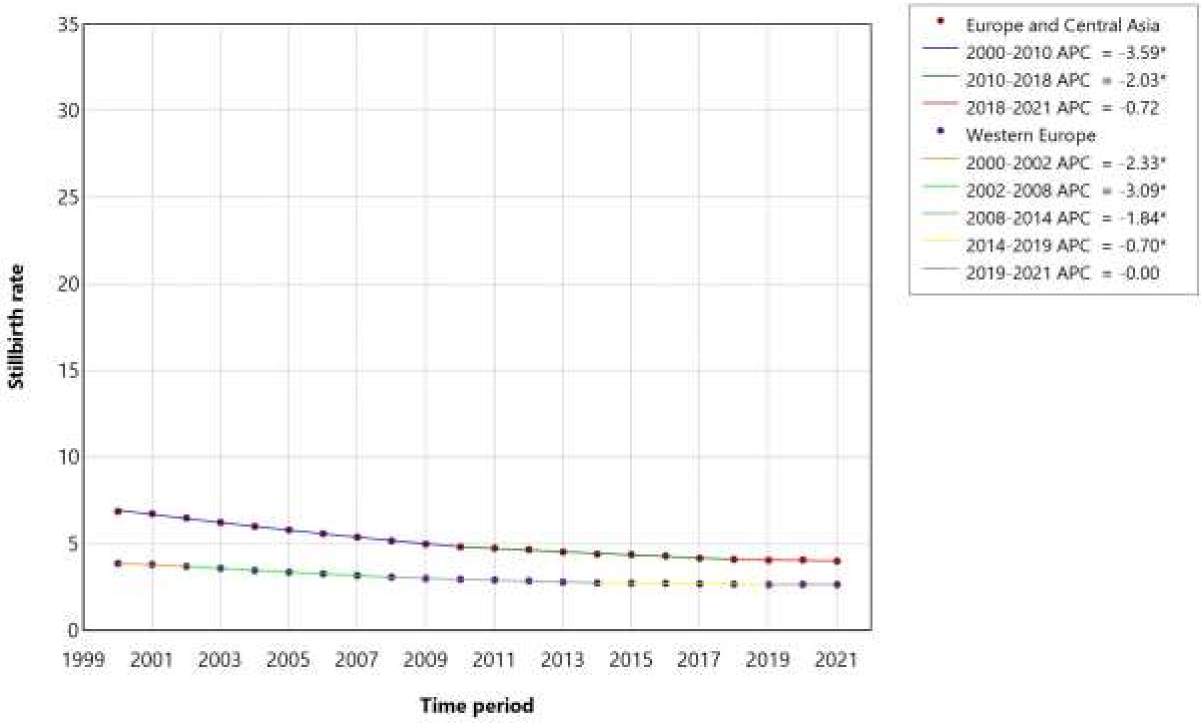

* Indicates that the Annual Percent Change (APC) is significantly different from zero at the alpha = 0.05 level.
Final Selected Model: Europe and Central Asia - 2 Joinpoints, Western Europe - 4 Joinpoints. Rejected Parallelism.

Europe and Central Asia: 2 Joinpoints versus West and Central Africa: 5 Joinpoints

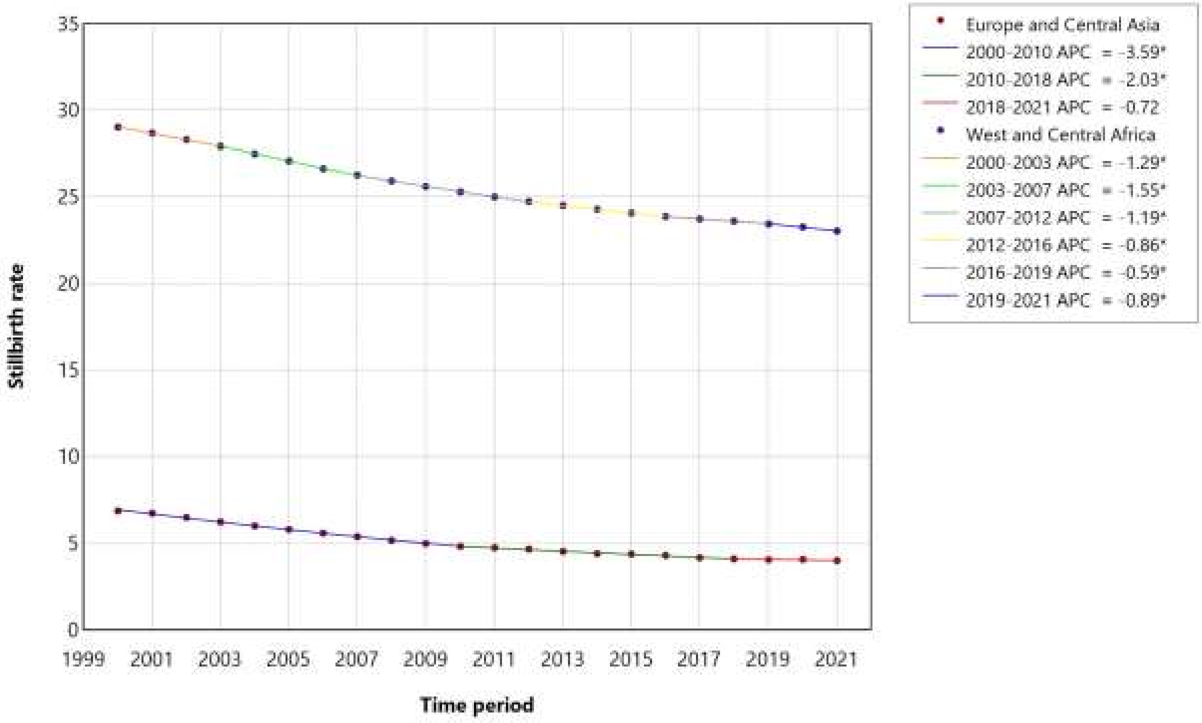

* Indicates that the Annual Percent Change (APC) is significantly different from zero at the alpha = 0.05 level.
Final Selected Model: Europe and Central Asia - 2 Joinpoints, West and Central Africa - 5 Joinpoints. Rejected Parallelism.

Europe and Central Asia: 2 Joinpoints versus Sub-Saharan Africa: 3 Joinpoints

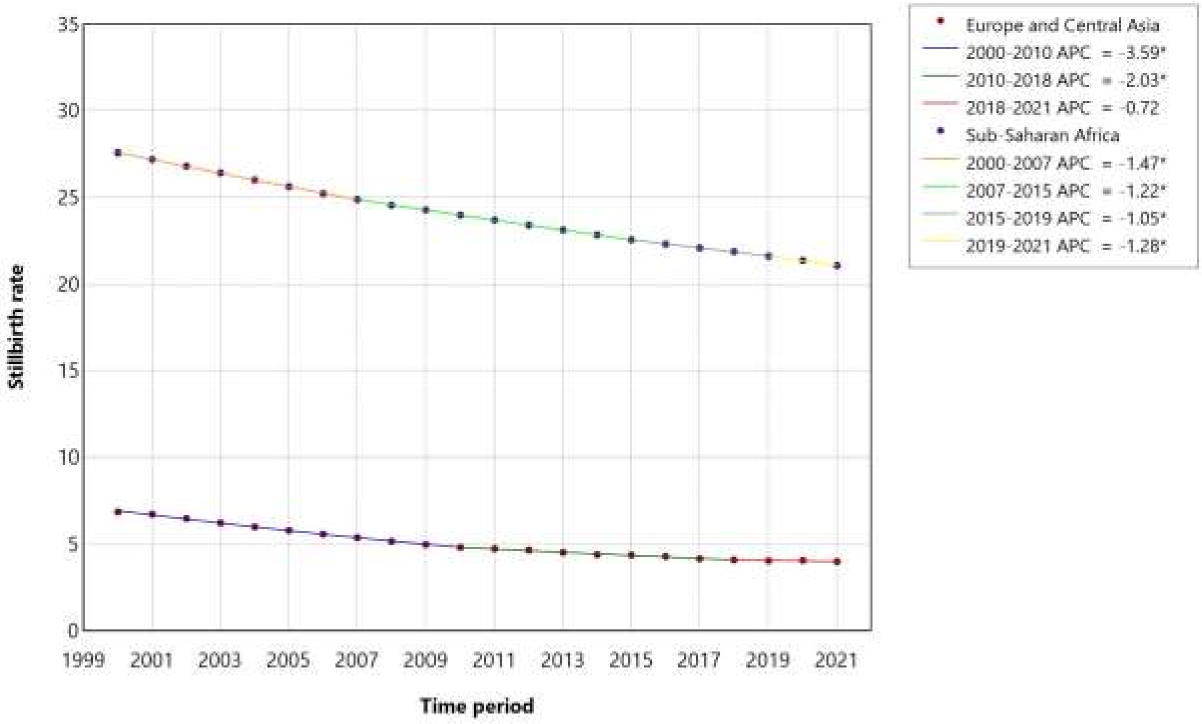

* Indicates that the Annual Percent Change (APC) is significantly different from zero at the alpha = 0.05 level.
Final Selected Model: Europe and Central Asia - 2 Joinpoints, Sub-Saharan Africa - 3 Joinpoints. Rejected Parallelism.

Europe and Central Asia: 2 Joinpoints versus South Asia: 5 Joinpoints

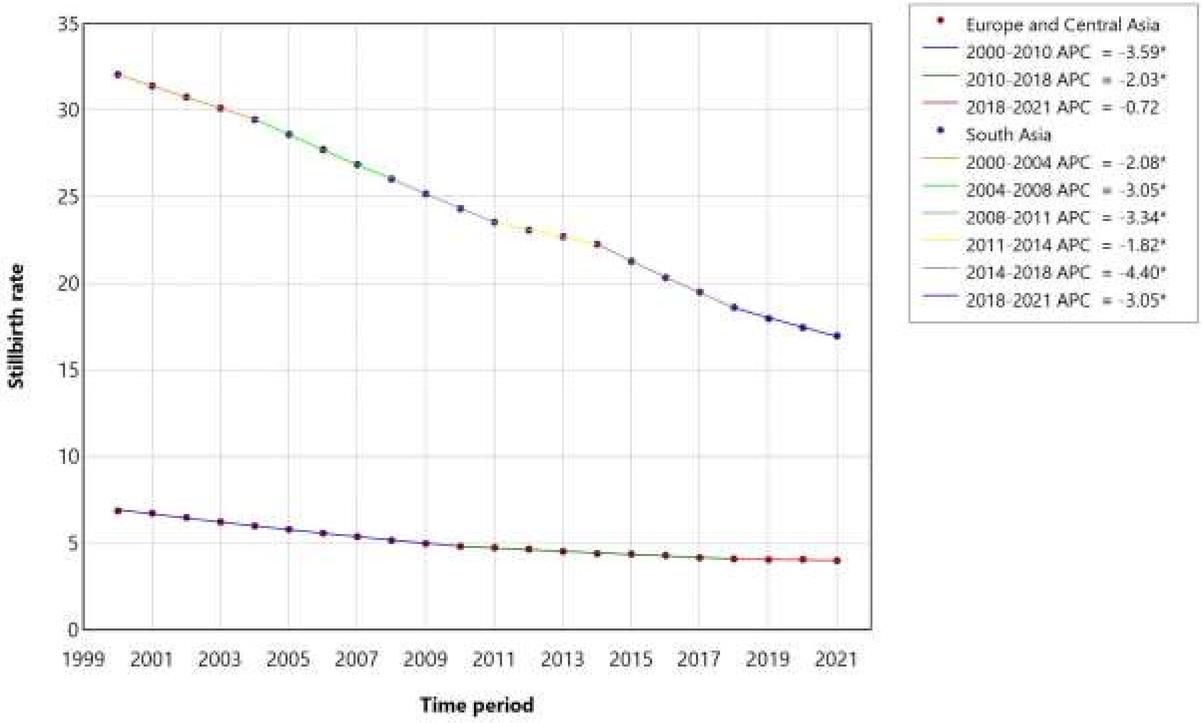

* Indicates that the Annual Percent Change (APC) is significantly different from zero at the alpha = 0.05 level.
Final Selected Model: Europe and Central Asia - 2 Joinpoints, South Asia - 5 Joinpoints. Rejected Parallelism.

Europe and Central Asia: 2 Joinpoints versus North America: 2 Joinpoints

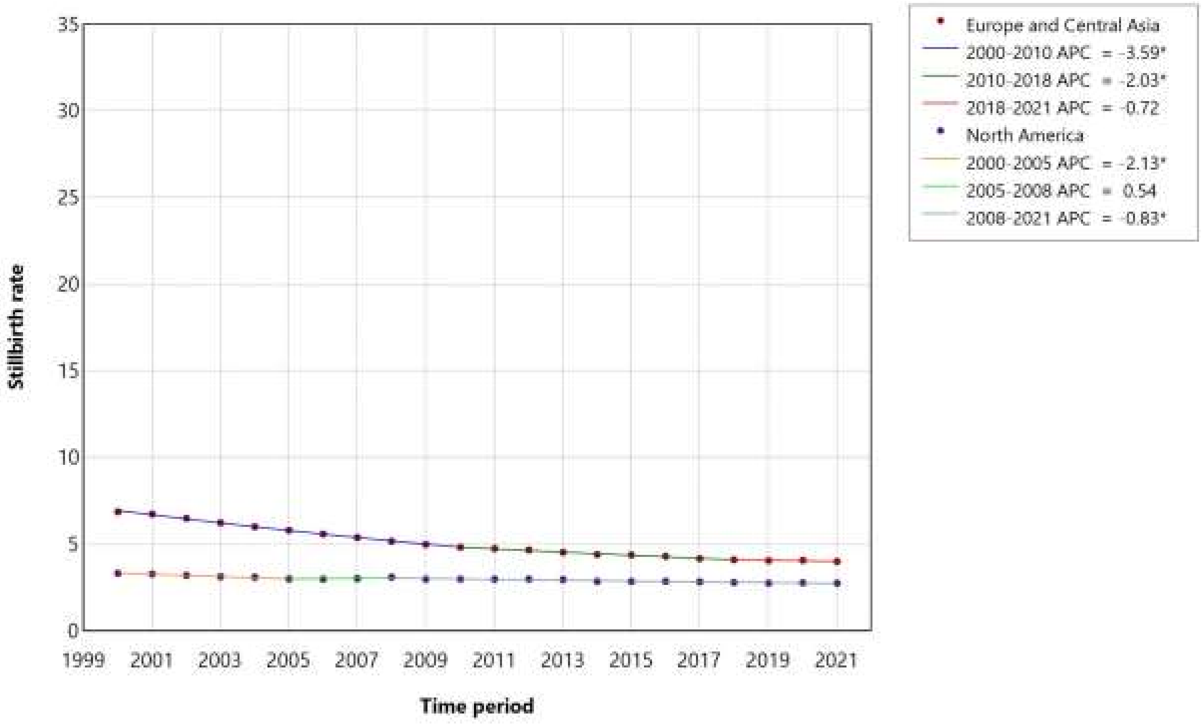

* Indicates that the Annual Percent Change (APC) is significantly different from zero at the alpha = 0.05 level.
Final Selected Model: Europe and Central Asia - 2 Joinpoints, North America - 2 Joinpoints. Rejected Parallelism.

Europe and Central Asia: 2 Joinpoints versus Middle East and North Africa: 4 Joinpoints

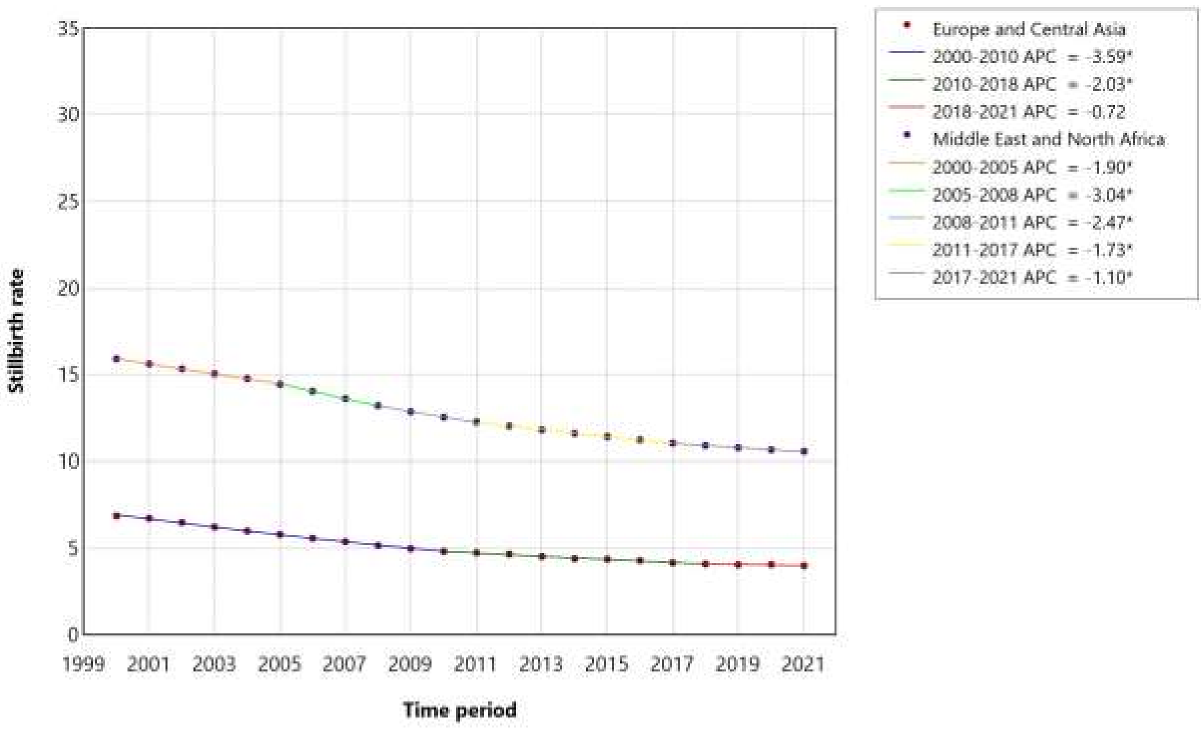

* Indicates that the Annual Percent Change (APC) is significantly different from zero at the alpha = 0.05 level.
Final Selected Model: Europe and Central Asia - 2 Joinpoints, Middle East and North Africa - 4 Joinpoints. Rejected Parallelism.

Europe and Central Asia: 2 Joinpoints versus Latin America and the Caribbean: 1 Joinpoint

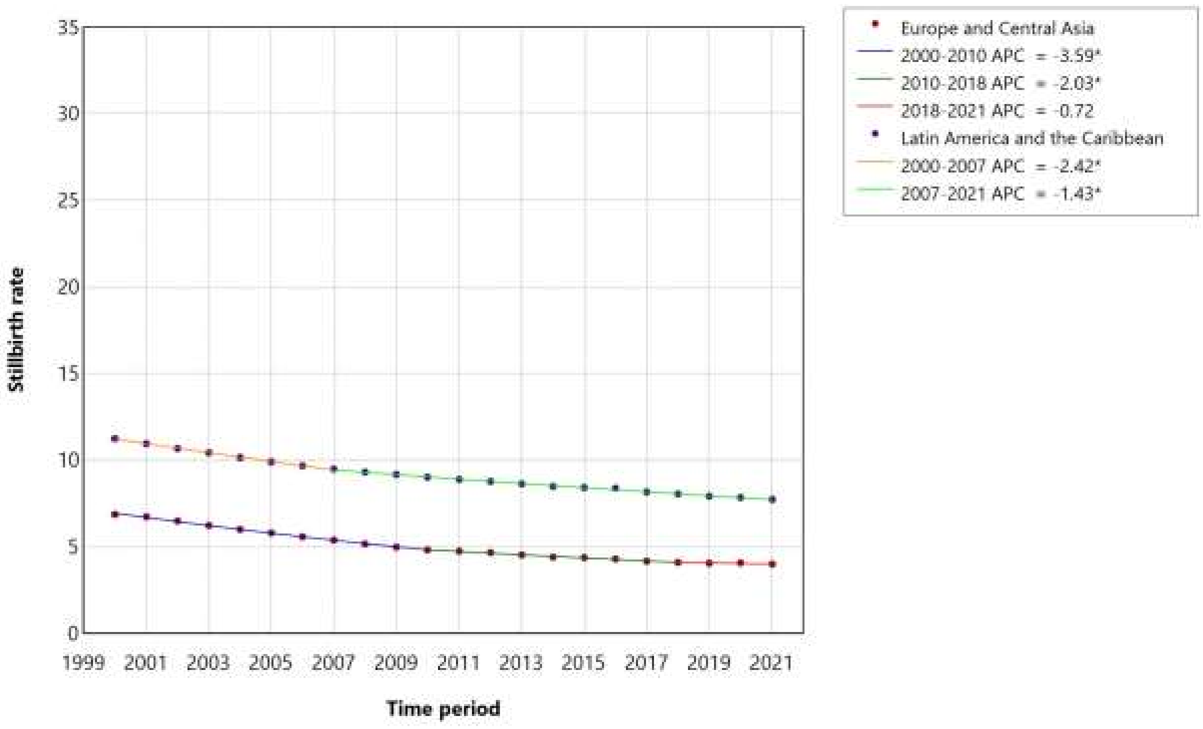

* Indicates that the Annual Percent Change (APC) is significantly different from zero at the alpha = 0.05 level.
Final Selected Model: Europe and Central Asia - 2 Joinpoints, Latin America and the Caribbean - 1 Joinpoint. Rejected Parallelism.

Eastern Europe and Central Asia: 4 Joinpoints versus Western Europe: 4 Joinpoints

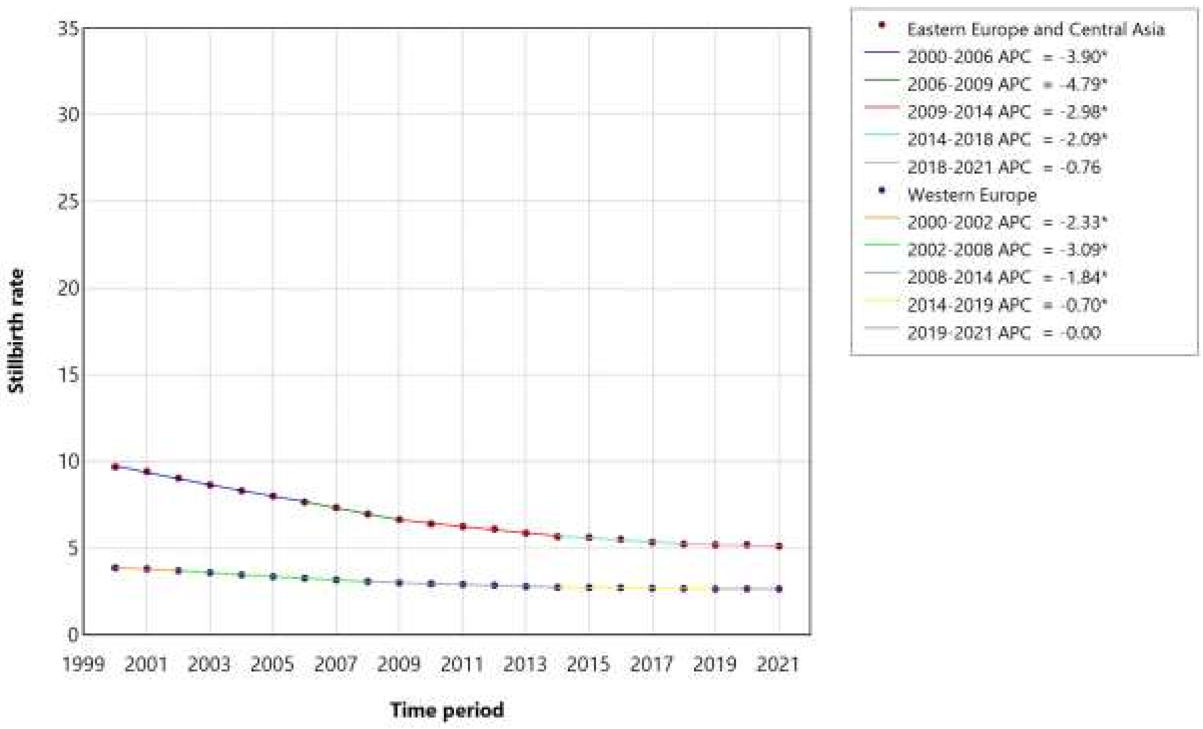

* Indicates that the Annual Percent Change (APC) is significantly different from zero at the alpha = 0.05 level.
Final Selected Model: Eastern Europe and Central Asia - 4 Joinpoints, Western Europe - 4 Joinpoints. Rejected Parallelism.

Eastern Europe and Central Asia: 4 Joinpoints versus West and Central Africa: 5 Joinpoints

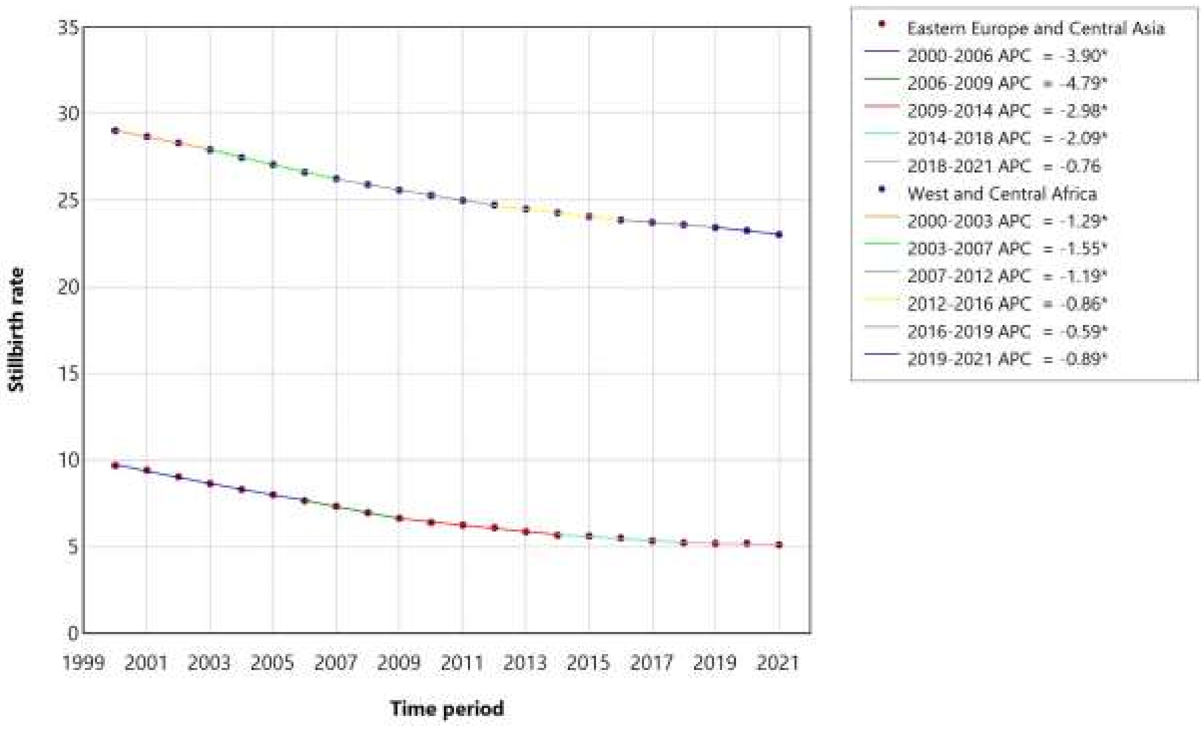

* Indicates that the Annual Percent Change (APC) is significantly different from zero at the alpha = 0.05 level.
Final Selected Model: Eastern Europe and Central Asia - 4 Joinpoints, West and Central Africa - 5 Joinpoints. Rejected Parallelism.

Eastern Europe and Central Asia: 4 Joinpoints versus Sub-Saharan Africa: 3 Joinpoints

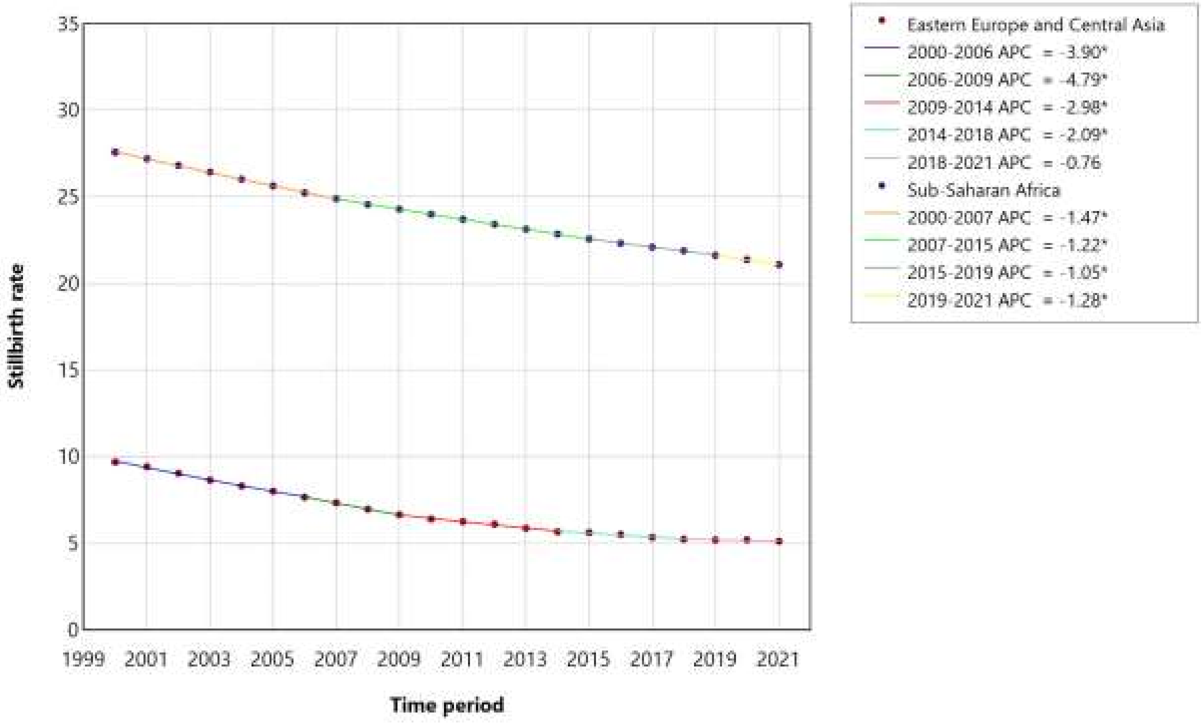

* Indicates that the Annual Percent Change (APC) is significantly different from zero at the alpha = 0.05 level.
Final Selected Model: Eastern Europe and Central Asia - 4 Joinpoints, Sub-Saharan Africa - 3 Joinpoints. Rejected Parallelism.

Eastern Europe and Central Asia: 4 Joinpoints versus South Asia: 5 Joinpoints

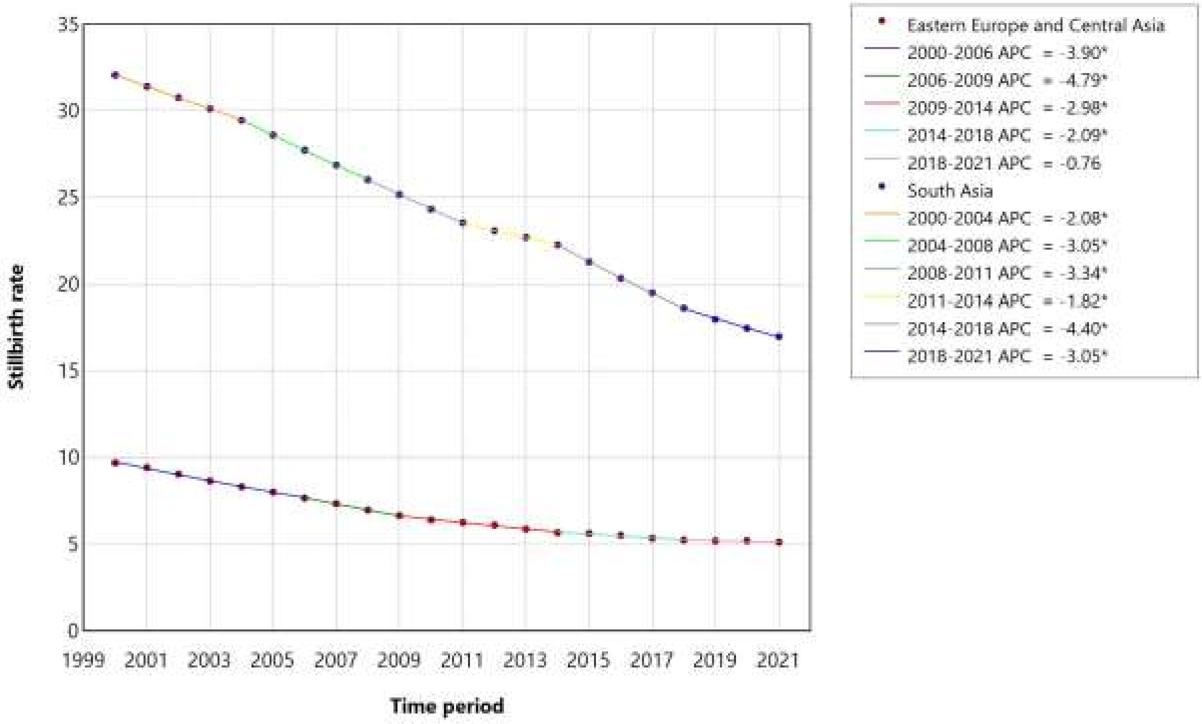

* Indicates that the Annual Percent Change (APC) is significantly different from zero at the alpha = 0.05 level.
Final Selected Model: Eastern Europe and Central Asia - 4 Joinpoints, South Asia - 5 Joinpoints. Rejected Parallelism.

Eastern Europe and Central Asia: 4 Joinpoints versus North America: 2 Joinpoints

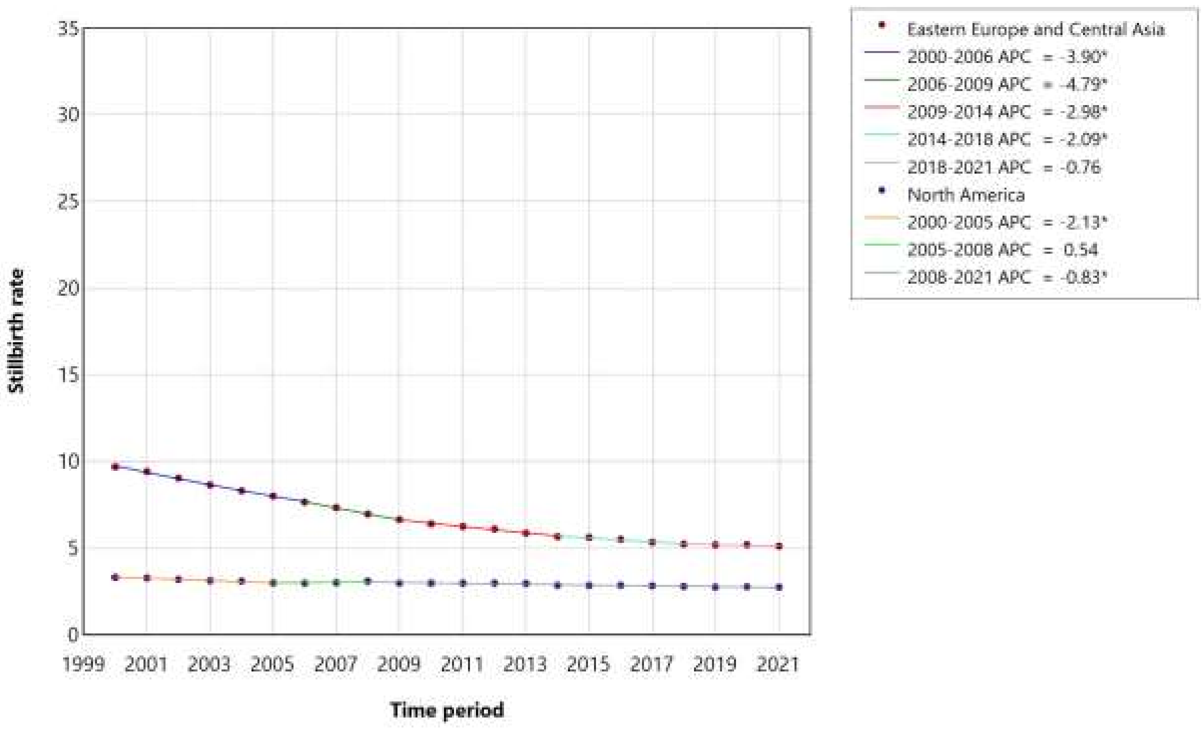

* Indicates that the Annual Percent Change (APC) is significantly different from zero at the alpha = 0.05 level.
Final Selected Model: Eastern Europe and Central Asia - 4 Joinpoints, North America - 2 Joinpoints. Rejected Parallelism.

Eastern Europe and Central Asia: 4 Joinpoints versus Middle East and North Africa: 4 Joinpoints

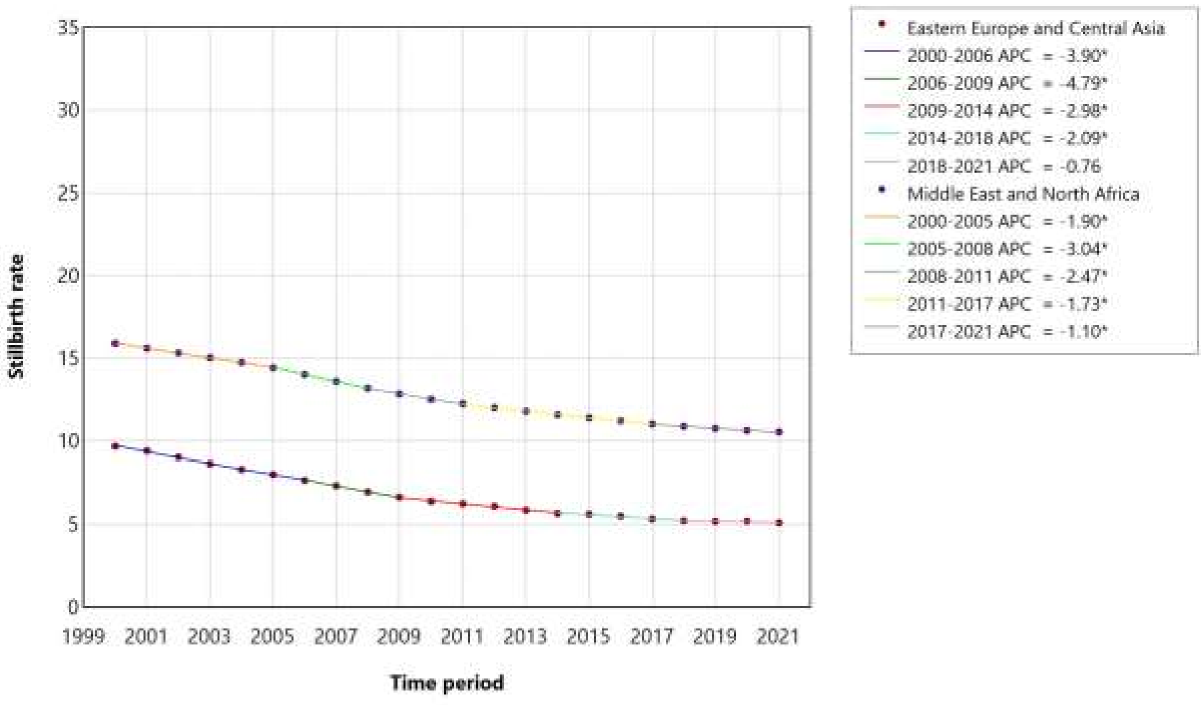

* Indicates that the Annual Percent Change (APC) is significantly different from zero at the alpha = 0.05 level.
Final Selected Model: Eastern Europe and Central Asia - 4 Joinpoints, Middle East and North Africa - 4 Joinpoints. Rejected Parallelism.

Eastern Europe and Central Asia: 4 Joinpoints versus Latin America and the Caribbean: 1 Joinpoint

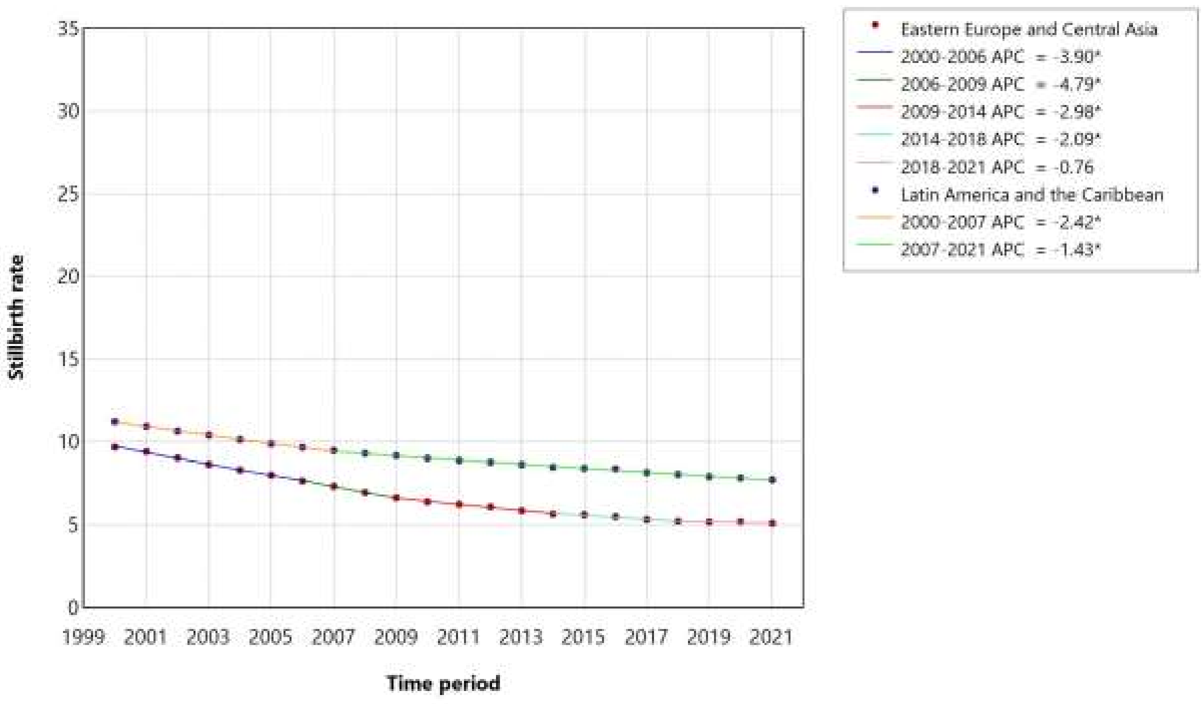

* Indicates that the Annual Percent Change (APC) is significantly different from zero at the alpha = 0.05 level.
Final Selected Model: Eastern Europe and Central Asia - 4 Joinpoints, Latin America and the Caribbean - 1 Joinpoint. Rejected Parallelism.

Eastern Europe and Central Asia: 4 Joinpoints versus Europe and Central Asia: 2 Joinpoints

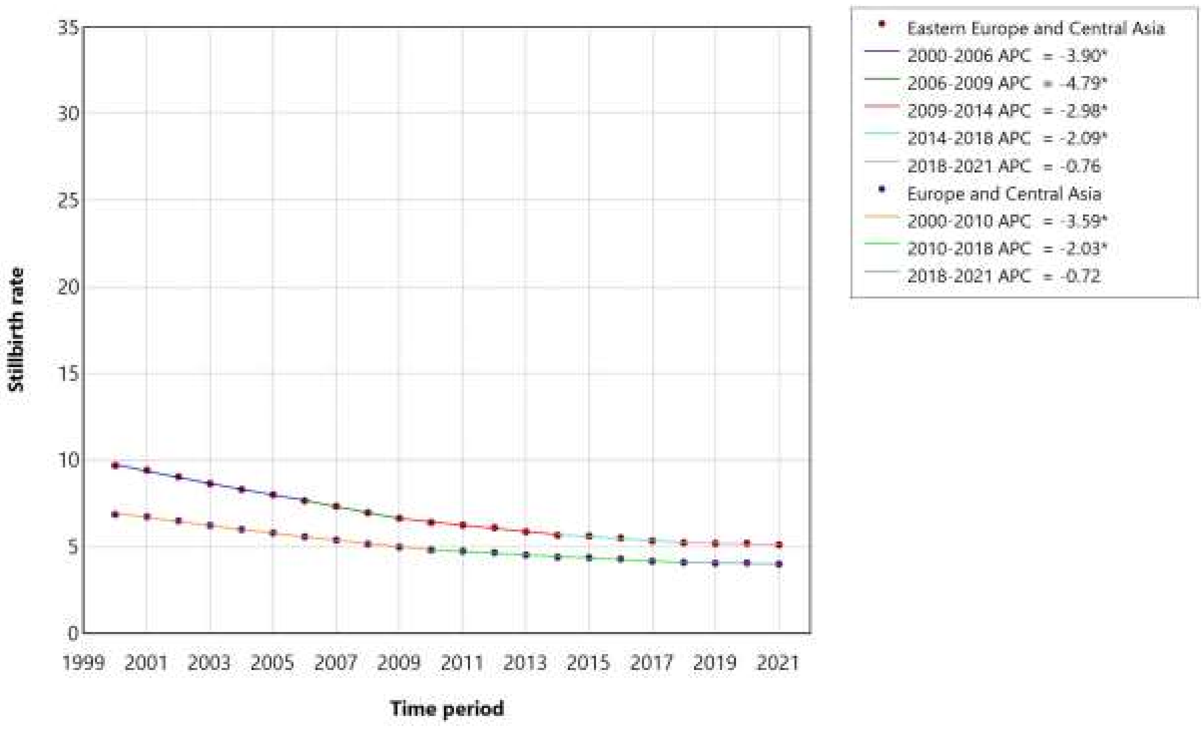

* Indicates that the Annual Percent Change (APC) is significantly different from zero at the alpha = 0.05 level.
Final Selected Model: Eastern Europe and Central Asia - 4 Joinpoints, Europe and Central Asia - 2 Joinpoints. Rejected Parallelism.

Eastern and Southern Africa: 3 Joinpoints versus Western Europe: 4 Joinpoints

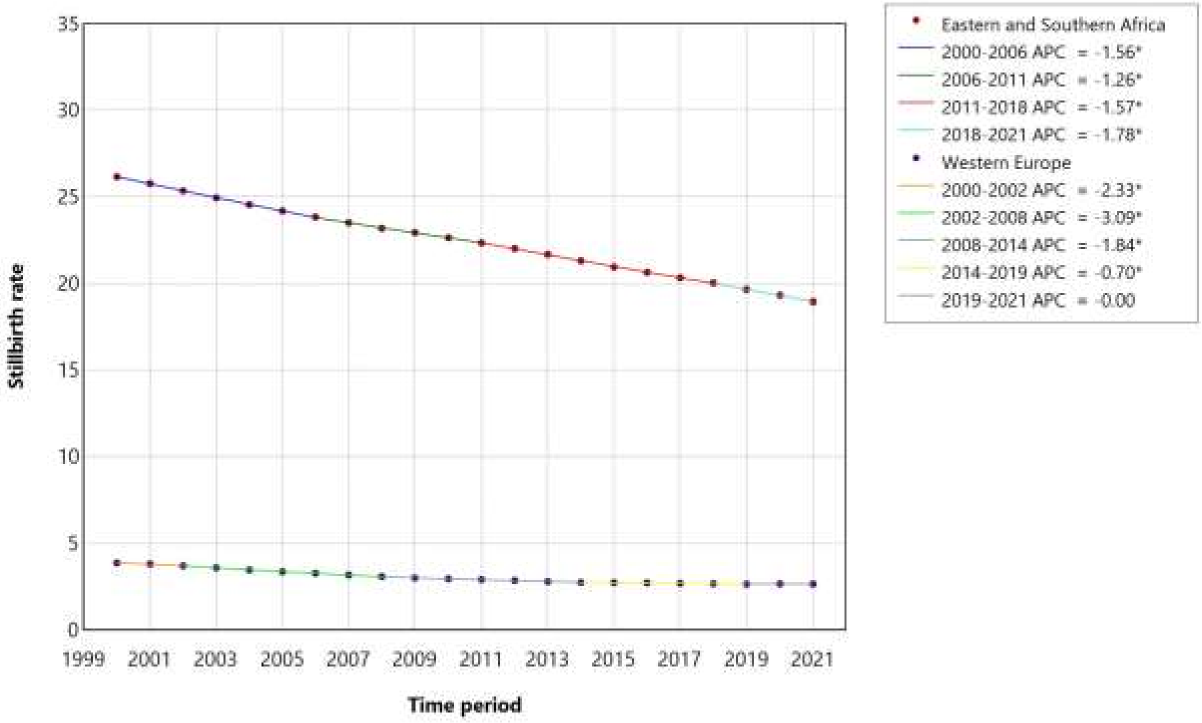

* Indicates that the Annual Percent Change (APC) is significantly different from zero at the alpha = 0.05 level.
Final Selected Model: Eastern and Southern Africa - 3 Joinpoints, Western Europe - 4 Joinpoints. Rejected Parallelism.

Eastern and Southern Africa: 3 Joinpoints versus West and Central Africa: 5 Joinpoints

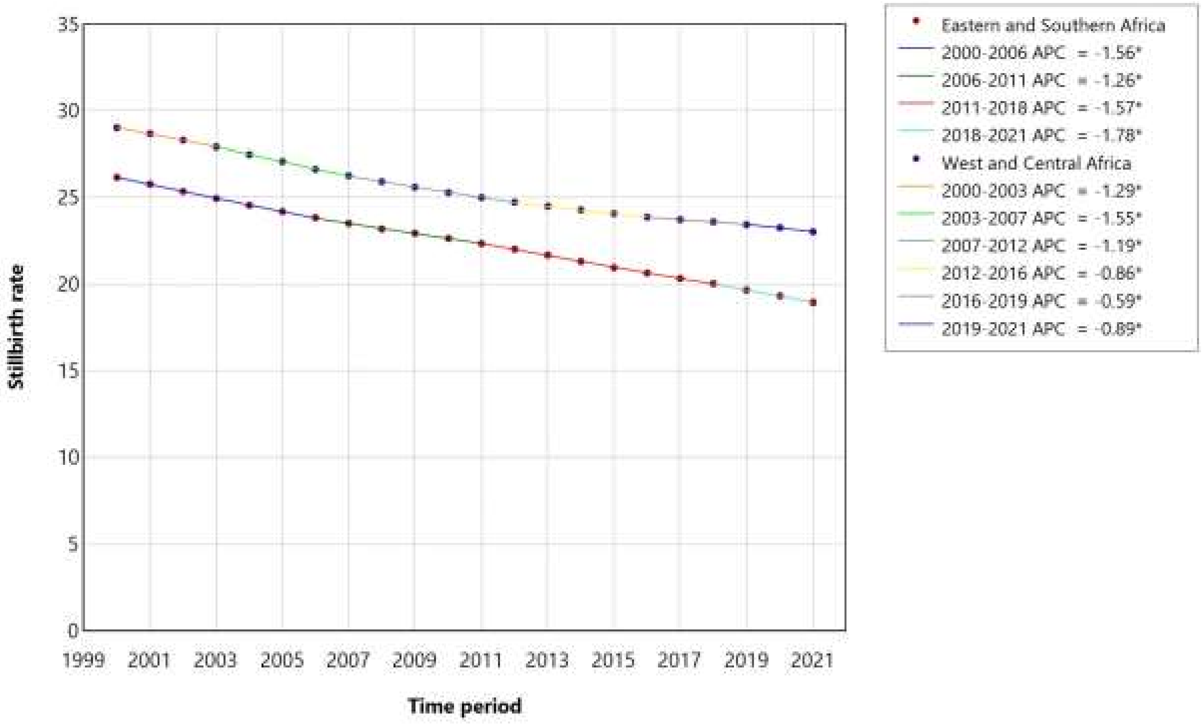

* Indicates that the Annual Percent Change (APC) is significantly different from zero at the alpha = 0.05 level.
Final Selected Model: Eastern and Southern Africa - 3 Joinpoints, West and Central Africa - 5 Joinpoints. Rejected Parallelism.

Eastern and Southern Africa: 3 Joinpoints versus Sub-Saharan Africa: 3 Joinpoints

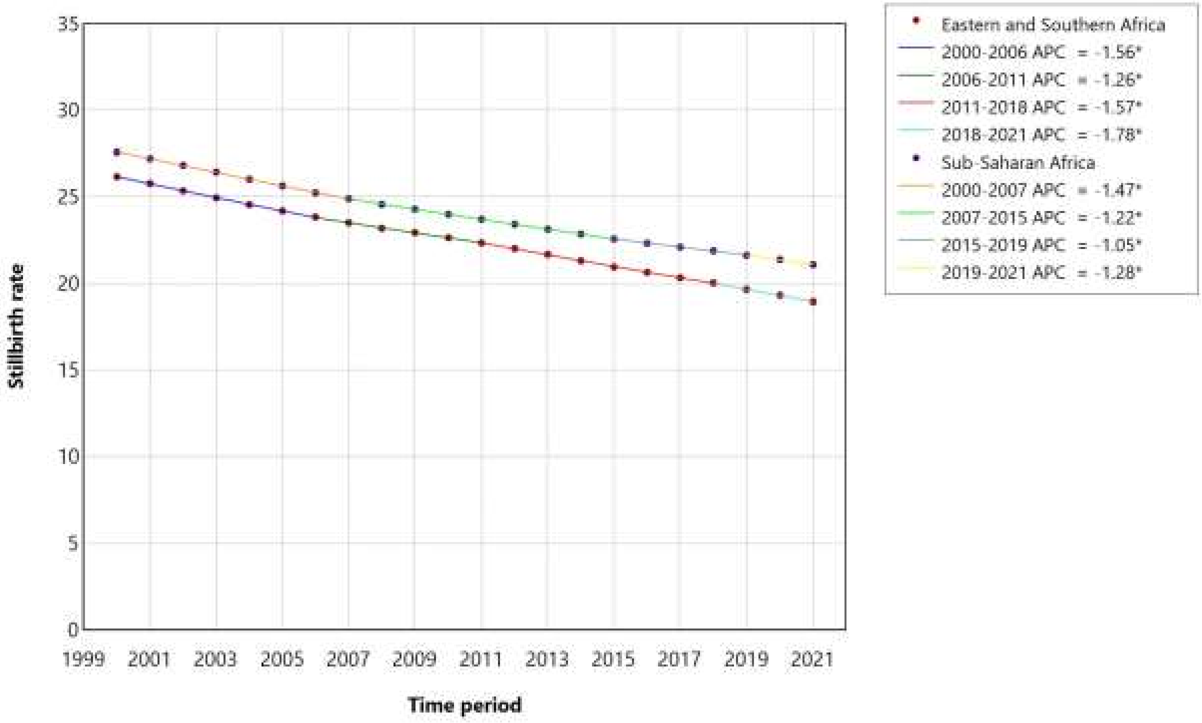

* Indicates that the Annual Percent Change (APC) is significantly different from zero at the alpha = 0.05 level.
Final Selected Model: Eastern and Southern Africa - 3 Joinpoints, Sub-Saharan Africa - 3 Joinpoints. Rejected Parallelism.

Eastern and Southern Africa: 3 Joinpoints versus South Asia: 5 Joinpoints

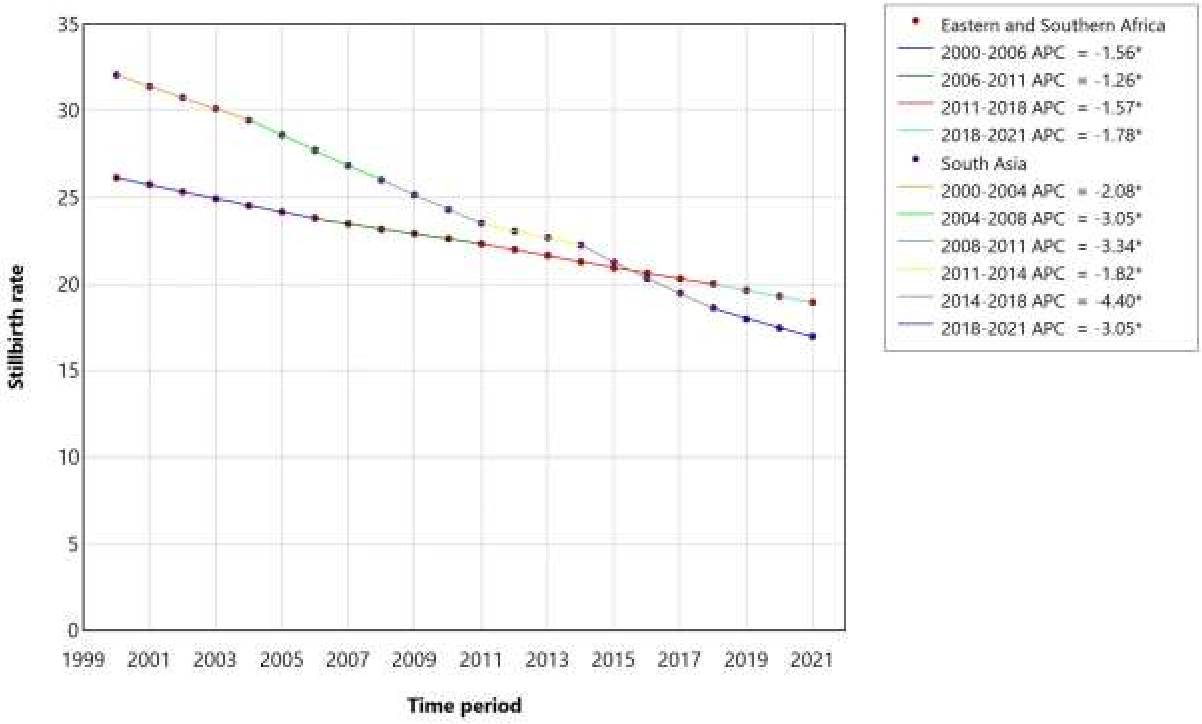

* Indicates that the Annual Percent Change (APC) is significantly different from zero at the alpha = 0.05 level.
Final Selected Model: Eastern and Southern Africa - 3 Joinpoints, South Asia - 5 Joinpoints. Rejected Parallelism.

Eastern and Southern Africa: 3 Joinpoints versus North America: 2 Joinpoints

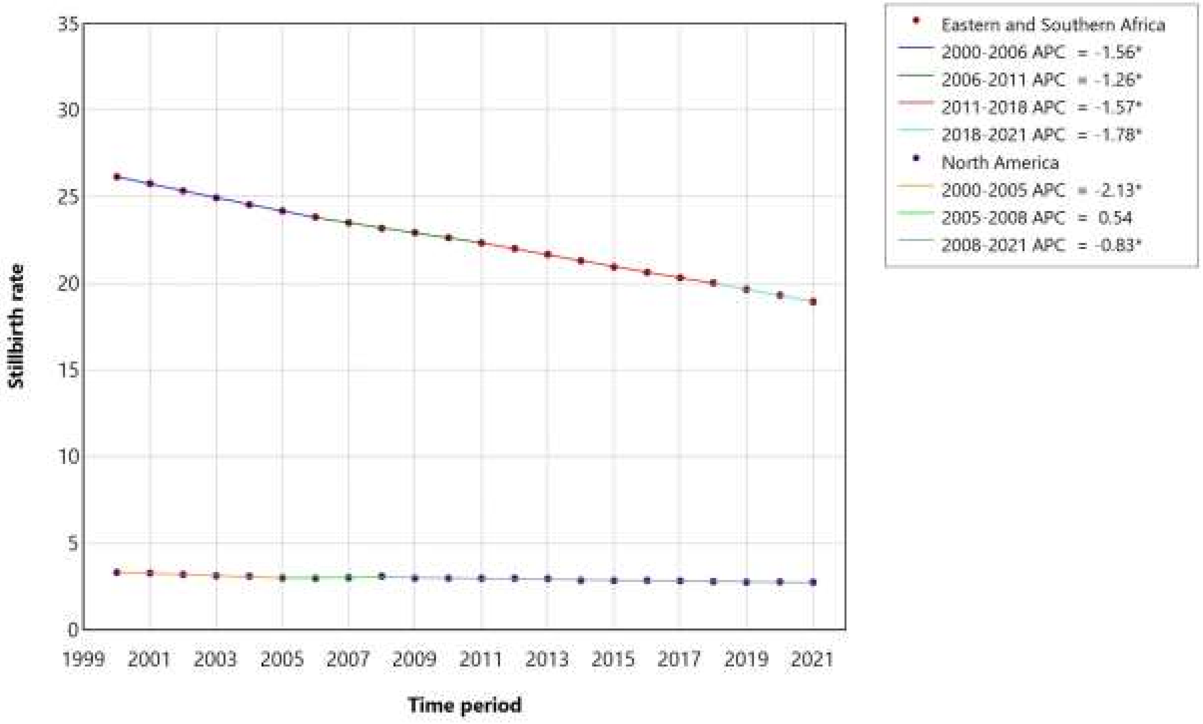

* Indicates that the Annual Percent Change (APC) is significantly different from zero at the alpha = 0.05 level.
Final Selected Model: Eastern and Southern Africa - 3 Joinpoints, North America - 2 Joinpoints. Rejected Parallelism.

Eastern and Southern Africa: 3 Joinpoints versus Middle East and North Africa: 4 Joinpoints

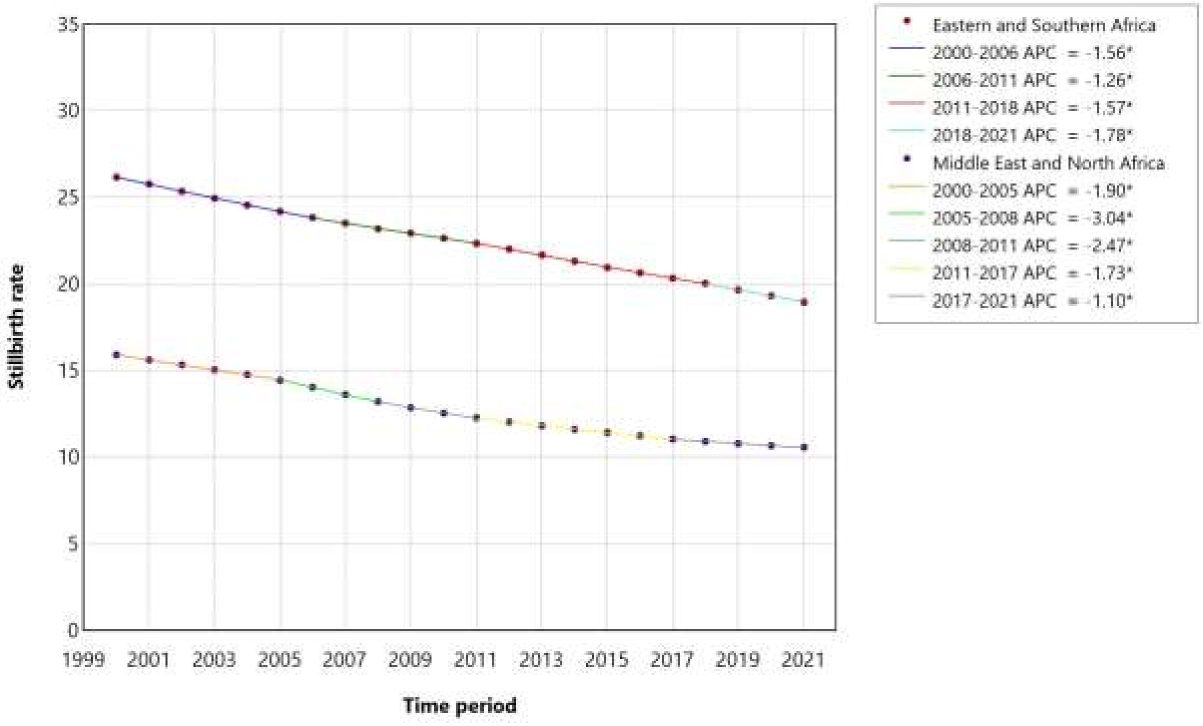

* Indicates that the Annual Percent Change (APC) is significantly different from zero at the alpha = 0.05 level.
Final Selected Model: Eastern and Southern Africa - 3 Joinpoints, Middle East and North Africa - 4 Joinpoints. Rejected Parallelism.

Eastern and Southern Africa: 3 Joinpoints versus Latin America and the Caribbean: 1 Joinpoint

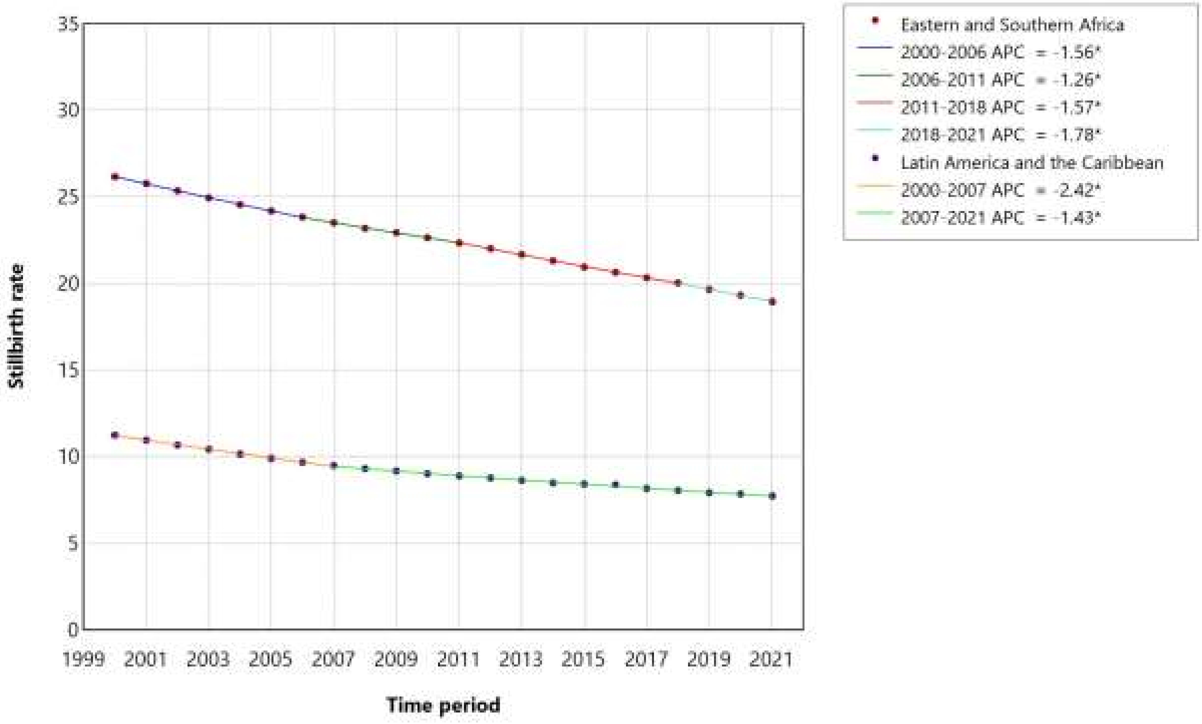

* Indicates that the Annual Percent Change (APC) is significantly different from zero at the alpha = 0.05 level.
Final Selected Model: Eastern and Southern Africa - 3 Joinpoints, Latin America and the Caribbean - 1 Joinpoint. Rejected Parallelism.

Eastern and Southern Africa: 3 Joinpoints versus Europe and Central Asia: 2 Joinpoints

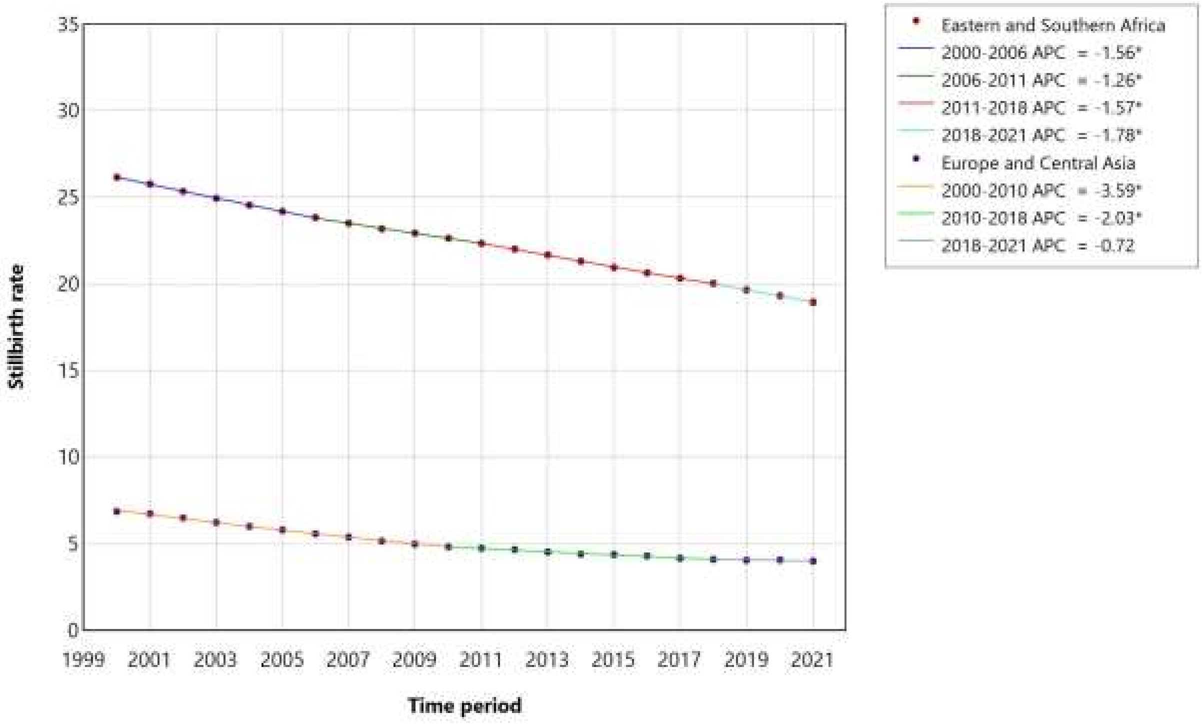

* Indicates that the Annual Percent Change (APC) is significantly different from zero at the alpha = 0.05 level.
Final Selected Model: Eastern and Southern Africa - 3 Joinpoints, Europe and Central Asia - 2 Joinpoints. Rejected Parallelism.

Eastern and Southern Africa: 3 Joinpoints versus Eastern Europe and Central Asia: 4 Joinpoints

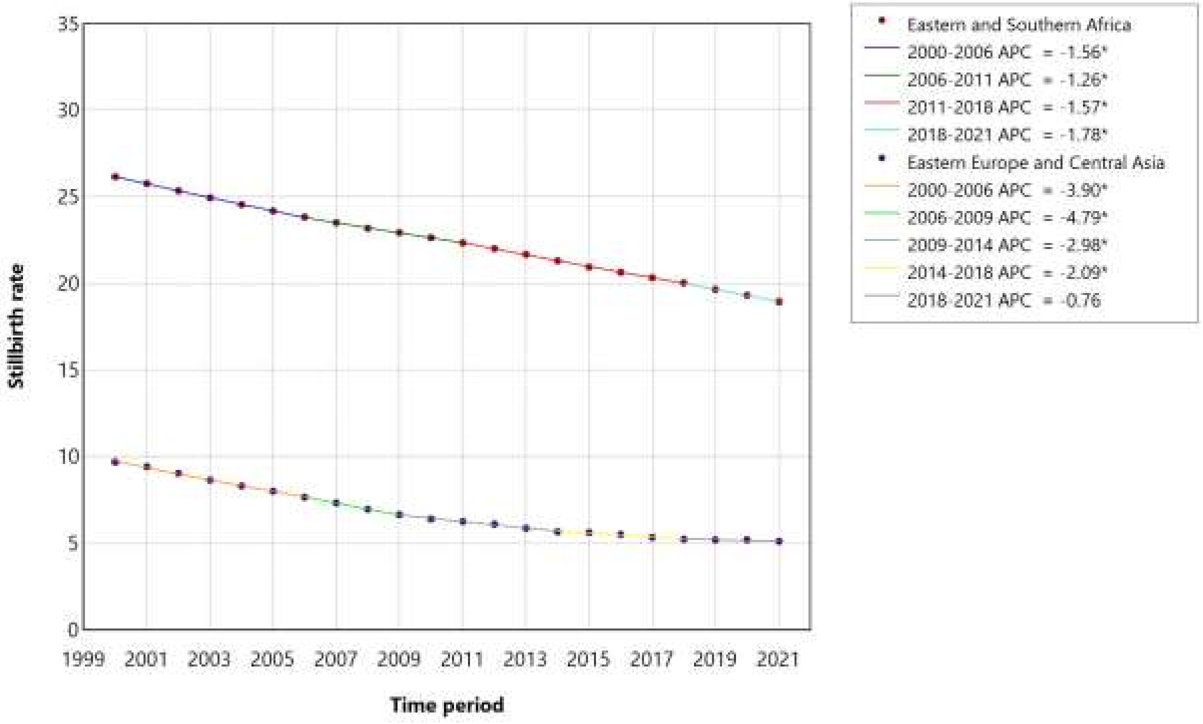

* Indicates that the Annual Percent Change (APC) is significantly different from zero at the alpha = 0.05 level.
Final Selected Model: Eastern and Southern Africa - 3 Joinpoints, Eastern Europe and Central Asia - 4 Joinpoints. Rejected Parallelism.

East Asia and Pacific: 3 Joinpoints versus Western Europe: 4 Joinpoints

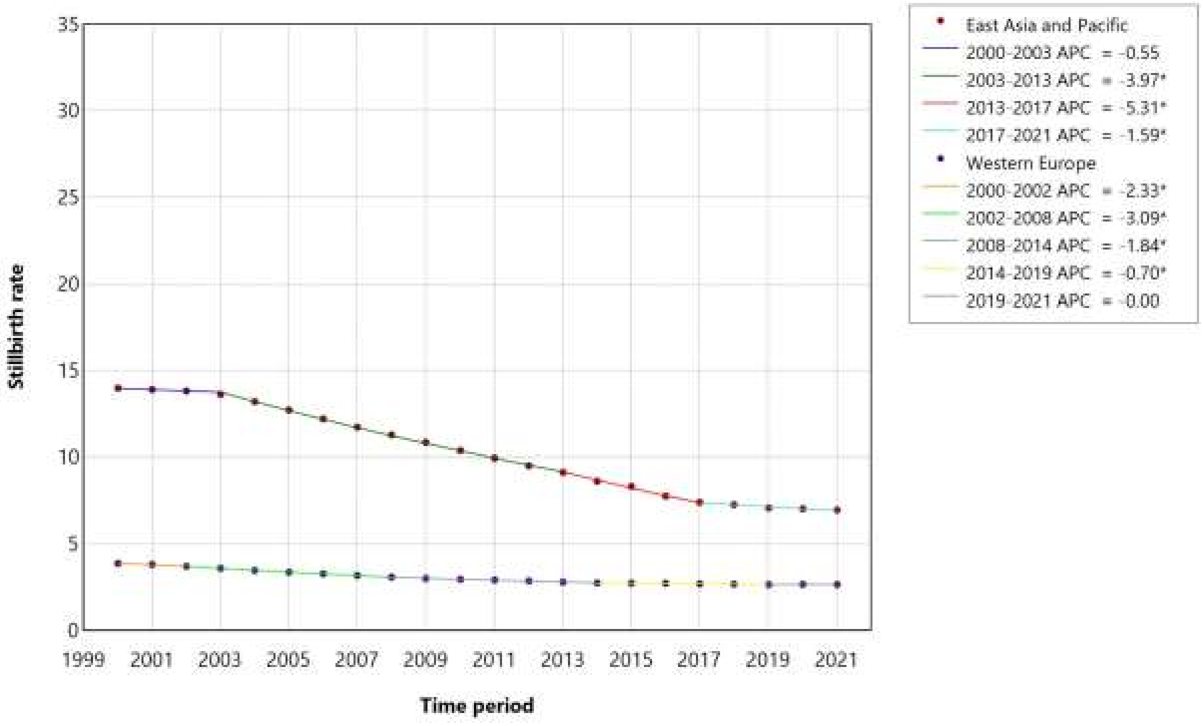

* Indicates that the Annual Percent Change (APC) is significantly different from zero at the alpha = 0.05 level.
Final Selected Model: East Asia and Pacific - 3 Joinpoints, Western Europe - 4 Joinpoints. Rejected Parallelism.

East Asia and Pacific: 3 Joinpoints versus West and Central Africa: 5 Joinpoints

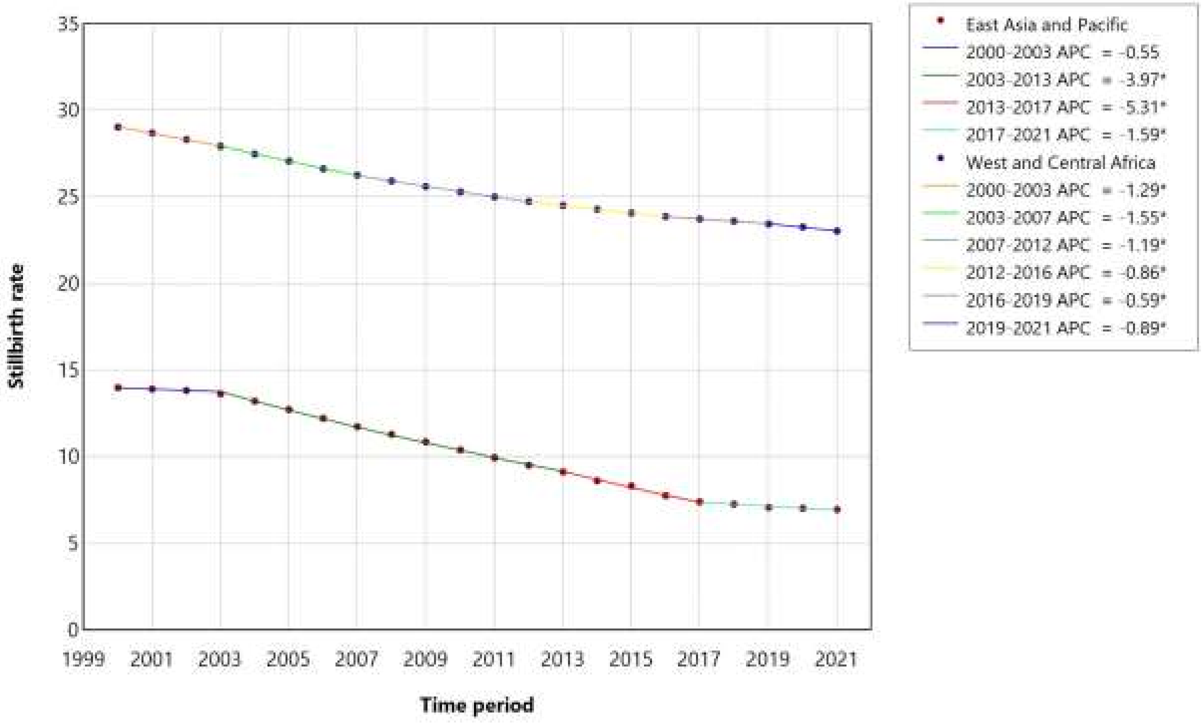

* Indicates that the Annual Percent Change (APC) is significantly different from zero at the alpha = 0.05 level.
Final Selected Model: East Asia and Pacific - 3 Joinpoints, West and Central Africa - 5 Joinpoints. Rejected Parallelism.

East Asia and Pacific: 3 Joinpoints versus Sub-Saharan Africa: 3 Joinpoints

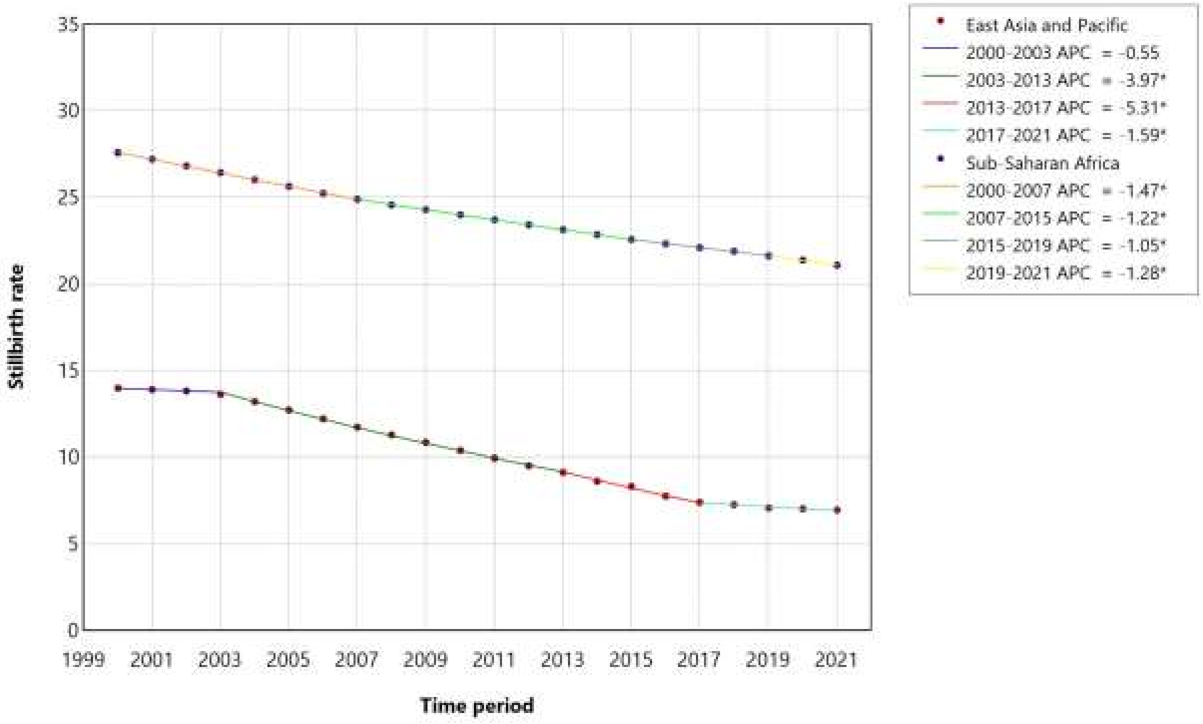

* Indicates that the Annual Percent Change (APC) is significantly different from zero at the alpha = 0.05 level.
Final Selected Model: East Asia and Pacific - 3 Joinpoints, Sub-Saharan Africa - 3 Joinpoints. Rejected Parallelism.

East Asia and Pacific: 3 Joinpoints versus South Asia: 5 Joinpoints

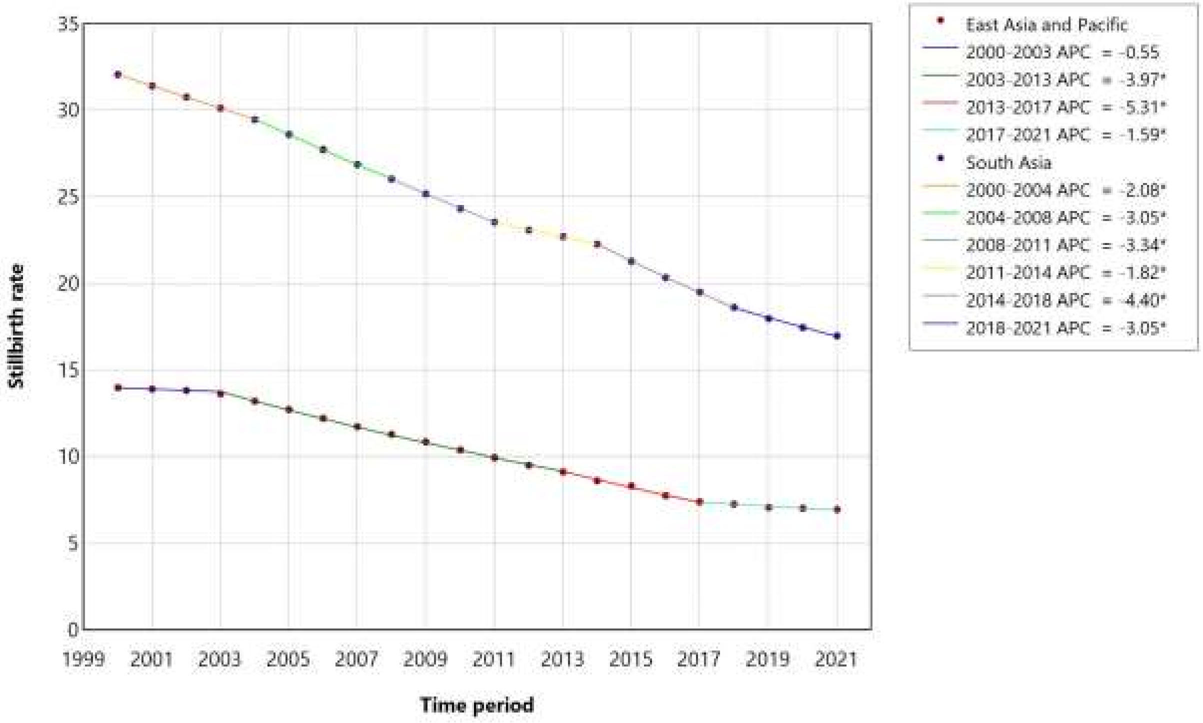

* Indicates that the Annual Percent Change (APC) is significantly different from zero at the alpha = 0.05 level.
Final Selected Model: East Asia and Pacific - 3 Joinpoints, South Asia - 5 Joinpoints. Rejected Parallelism.

Figure S2. Pairwise comparisons of temporal trend in neonatal mortality rates across UNICEF reporting regions using joinpoint regression

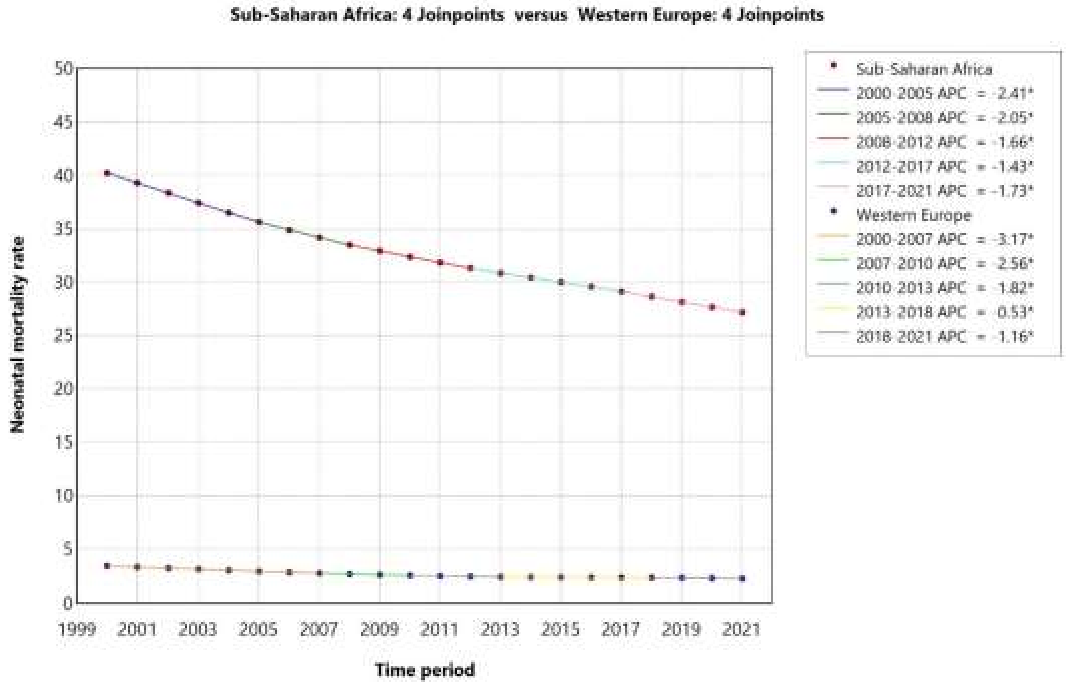

* Indicates that the Annual Percent Change (APC) is significantly different from zero at the alpha = 0.05 level.
Final Selected Model: Sub-Saharan Africa - 4 Joinpoints, Western Europe - 4 Joinpoints. Rejected Parallelism.

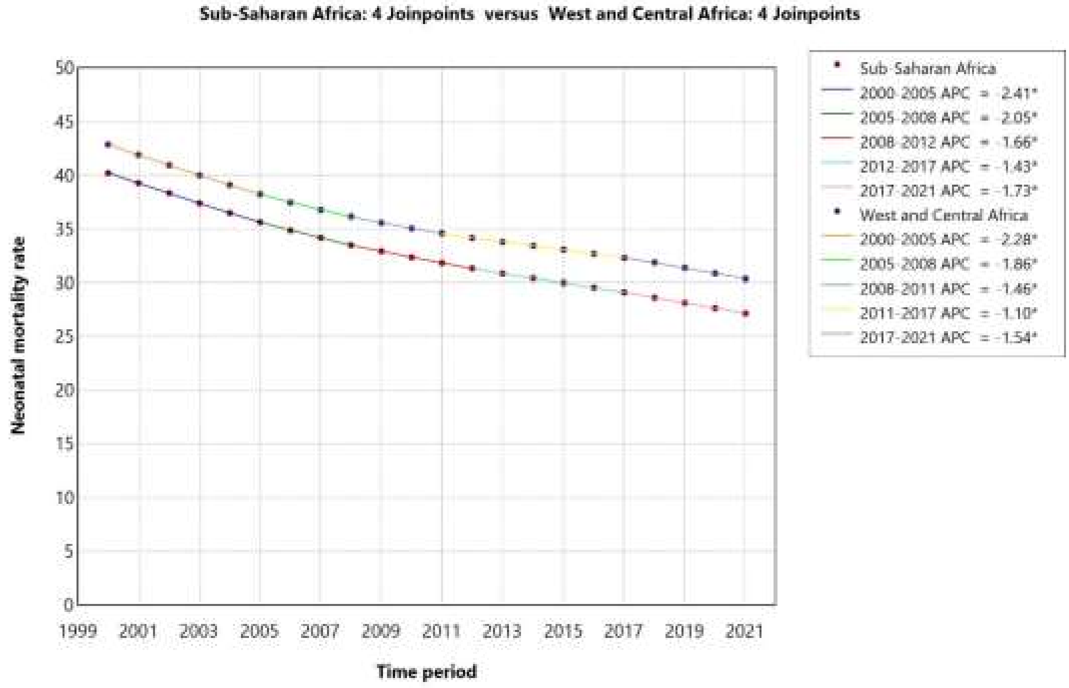

* Indicates that the Annual Percent Change (APC) is significantly different from zero at the alpha = 0.05 level.
Final Selected Model: Sub-Saharan Africa - 4 Joinpoints, West and Central Africa - 4 Joinpoints. Rejected Parallelism.

South Asia: 4 Joinpoints versus Western Europe: 4 Joinpoints

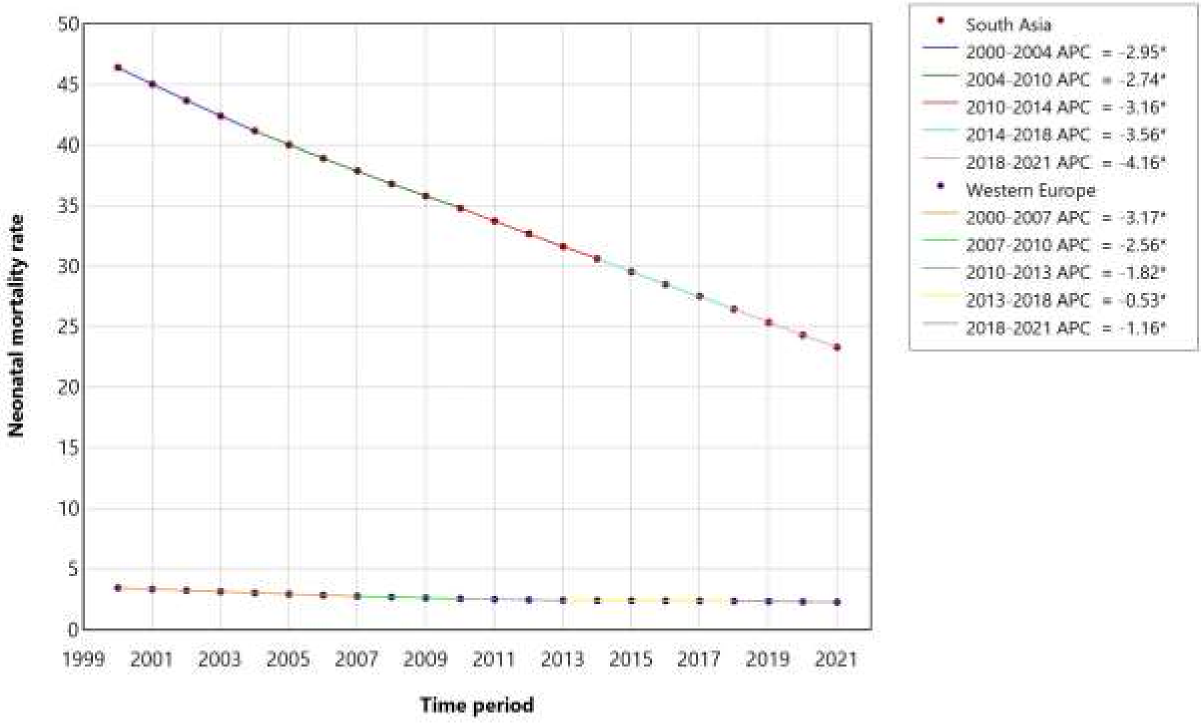

* Indicates that the Annual Percent Change (APC) is significantly different from zero at the alpha = 0.05 level.
Final Selected Model: South Asia - 4 Joinpoints, Western Europe - 4 Joinpoints. Rejected Parallelism.

South Asia: 4 Joinpoints versus West and Central Africa: 4 Joinpoints

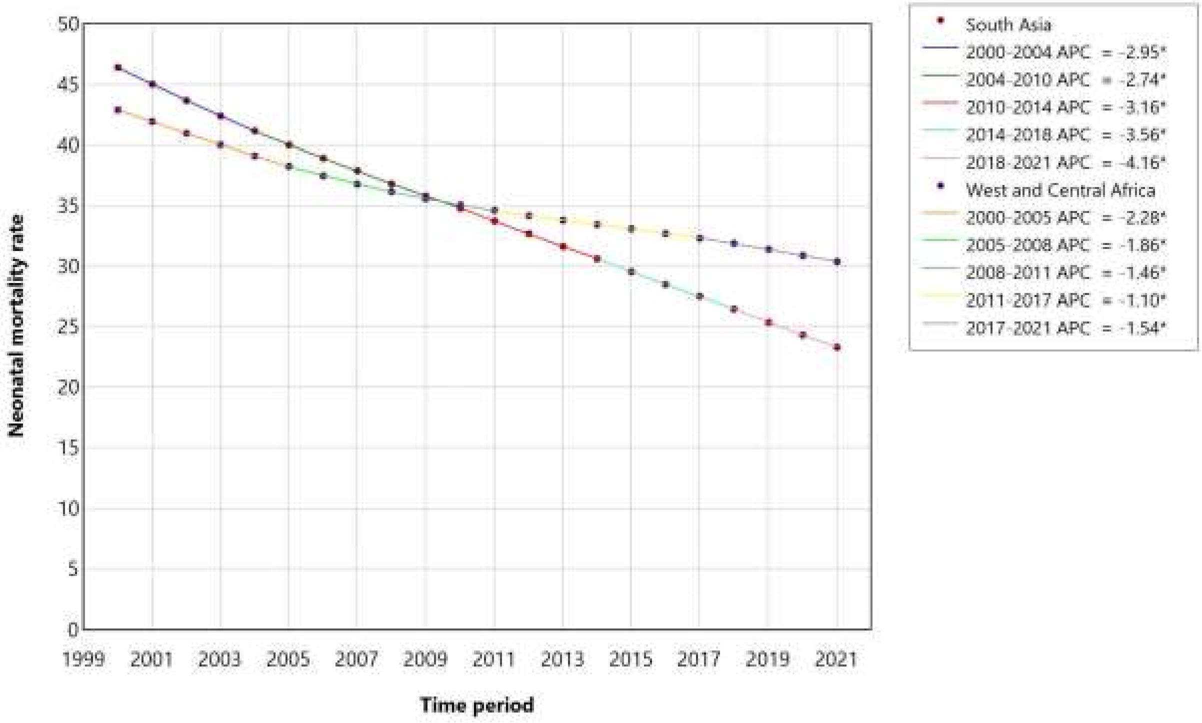

* Indicates that the Annual Percent Change (APC) is significantly different from zero at the alpha = 0.05 level.
Final Selected Model: South Asia - 4 Joinpoints, West and Central Africa - 4 Joinpoints. Rejected Parallelism.

South Asia: 4 Joinpoints versus Sub-Saharan Africa: 4 Joinpoints

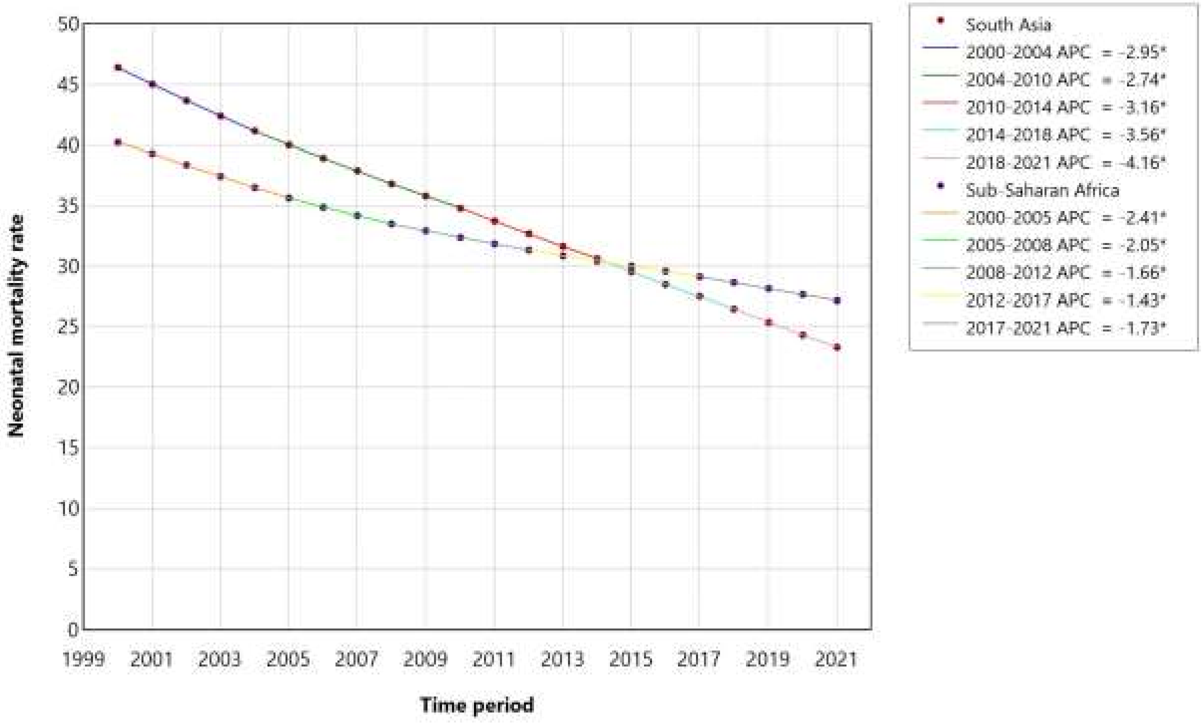

* Indicates that the Annual Percent Change (APC) is significantly different from zero at the alpha = 0.05 level.
Final Selected Model: South Asia - 4 Joinpoints, Sub-Saharan Africa - 4 Joinpoints. Rejected Parallelism.

North America: 5 Joinpoints versus Western Europe: 4 Joinpoints

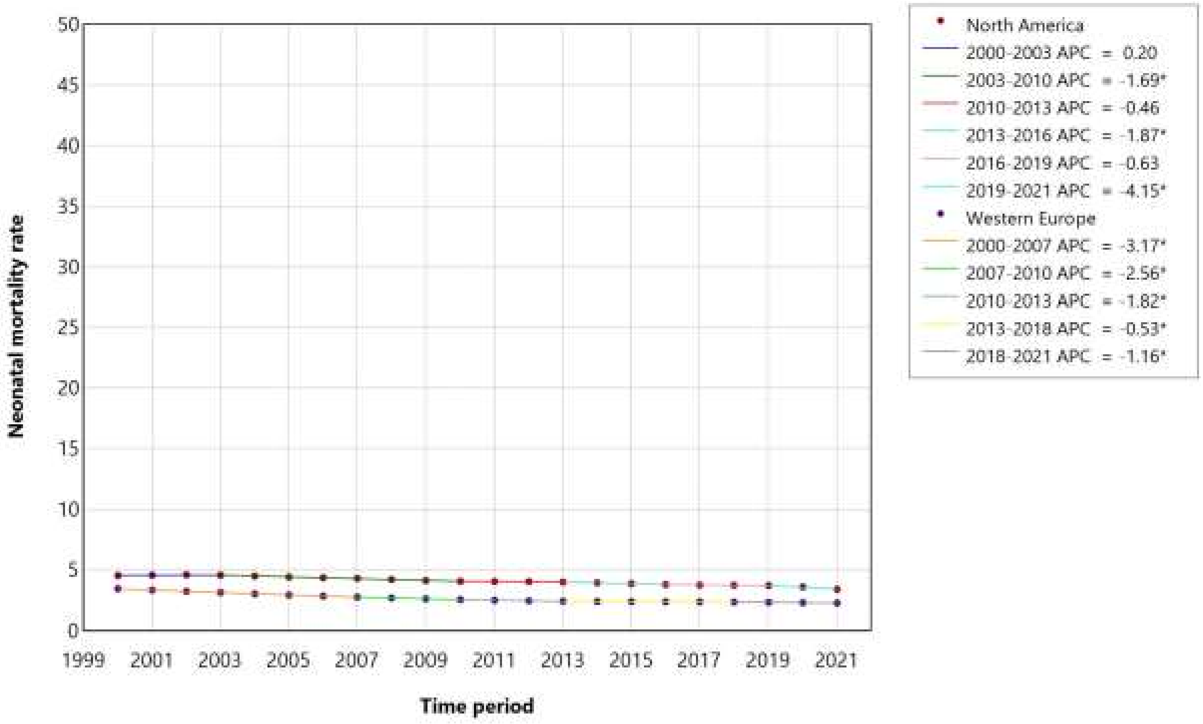

* Indicates that the Annual Percent Change (APC) is significantly different from zero at the alpha = 0.05 level.
Final Selected Model: North America - 5 Joinpoints, Western Europe - 4 Joinpoints. Rejected Parallelism.

North America: 5 Joinpoints versus West and Central Africa: 4 Joinpoints

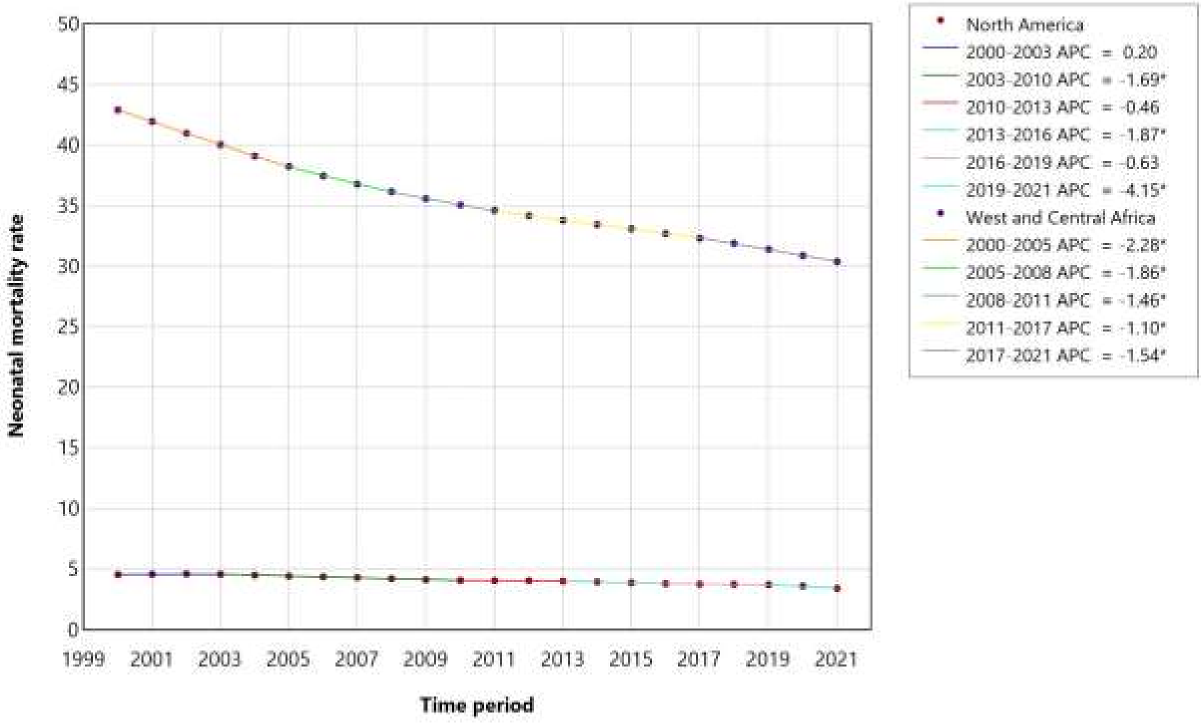

* Indicates that the Annual Percent Change (APC) is significantly different from zero at the alpha = 0.05 level.
Final Selected Model: North America - 5 Joinpoints, West and Central Africa - 4 Joinpoints. Rejected Parallelism.

North America: 5 Joinpoints versus Sub-Saharan Africa: 4 Joinpoints

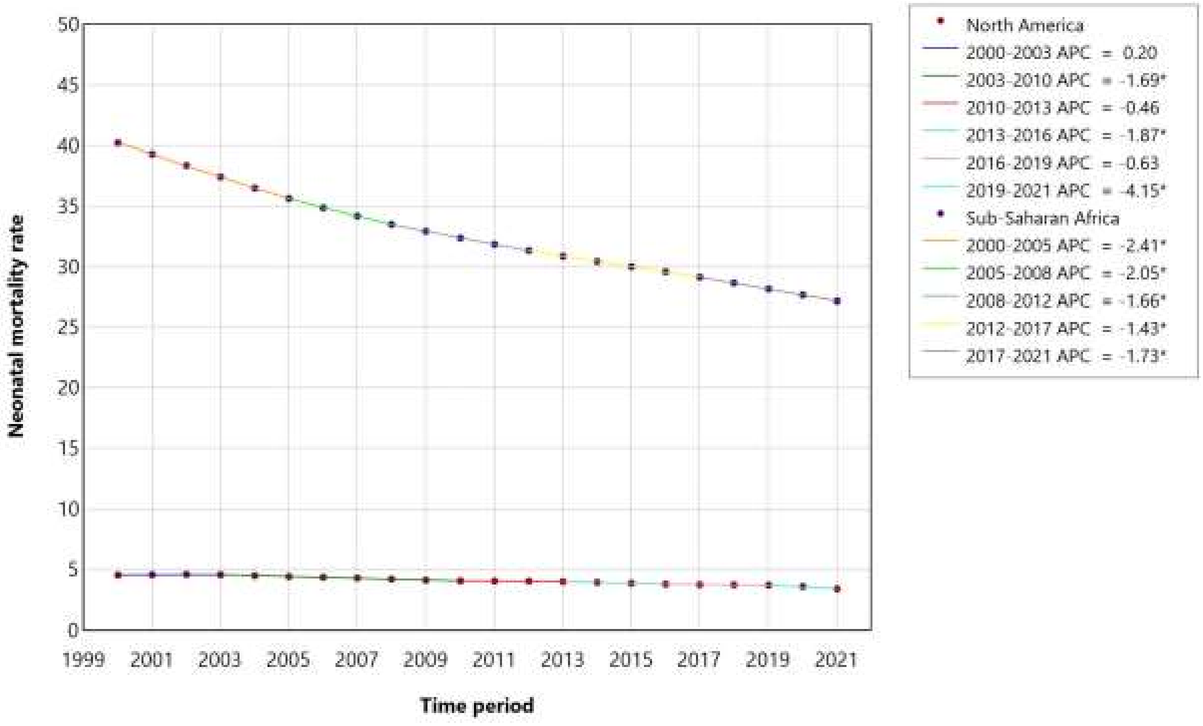

* Indicates that the Annual Percent Change (APC) is significantly different from zero at the alpha = 0.05 level.
Final Selected Model: North America - 5 Joinpoints, Sub-Saharan Africa - 4 Joinpoints. Rejected Parallelism.

North America: 5 Joinpoints versus South Asia: 4 Joinpoints

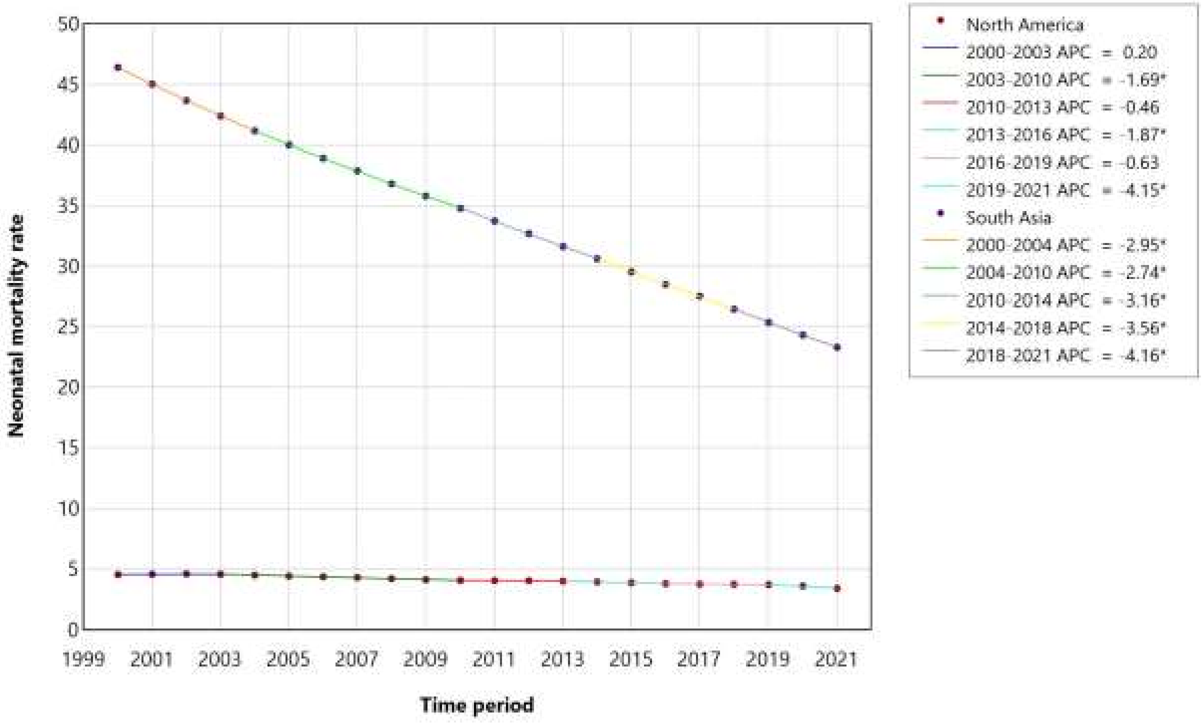

* Indicates that the Annual Percent Change (APC) is significantly different from zero at the alpha = 0.05 level.
Final Selected Model: North America - 5 Joinpoints, South Asia - 4 Joinpoints. Rejected Parallelism.

Middle East and North Africa: 3 Joinpoints versus Western Europe: 4 Joinpoints

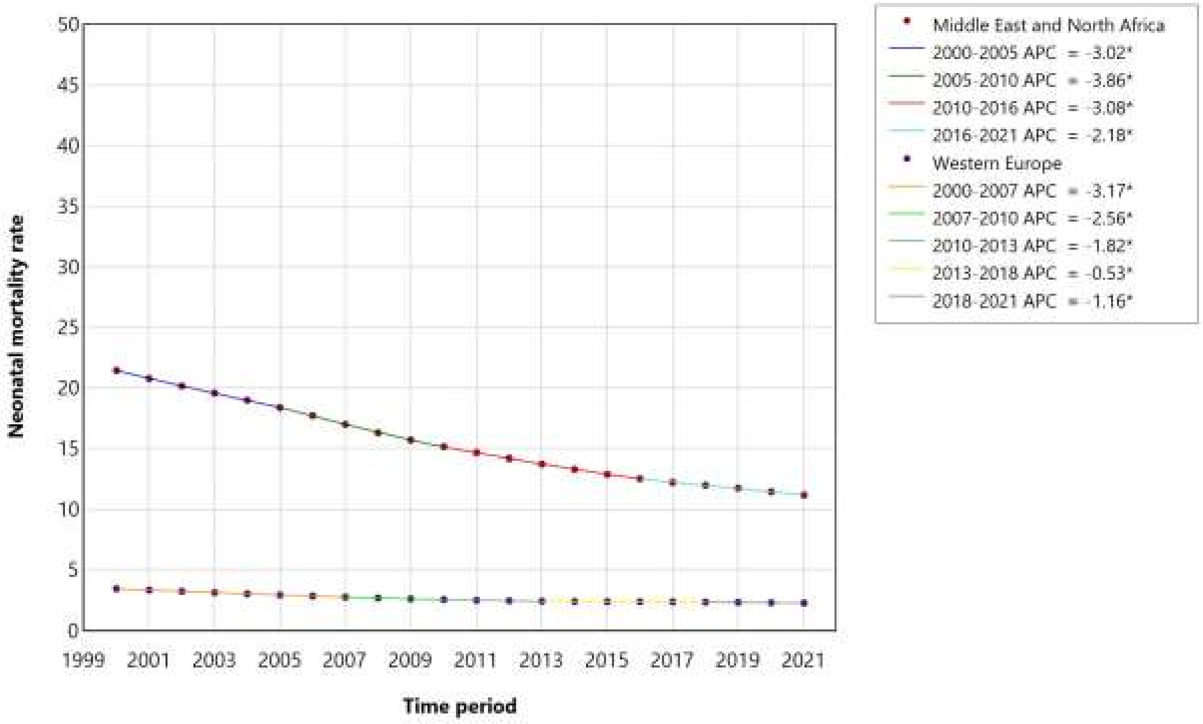

* Indicates that the Annual Percent Change (APC) is significantly different from zero at the alpha = 0.05 level.
Final Selected Model: Middle East and North Africa - 3 Joinpoints, Western Europe - 4 Joinpoints. Rejected Parallelism.

Middle East and North Africa: 3 Joinpoints versus West and Central Africa: 4 Joinpoints

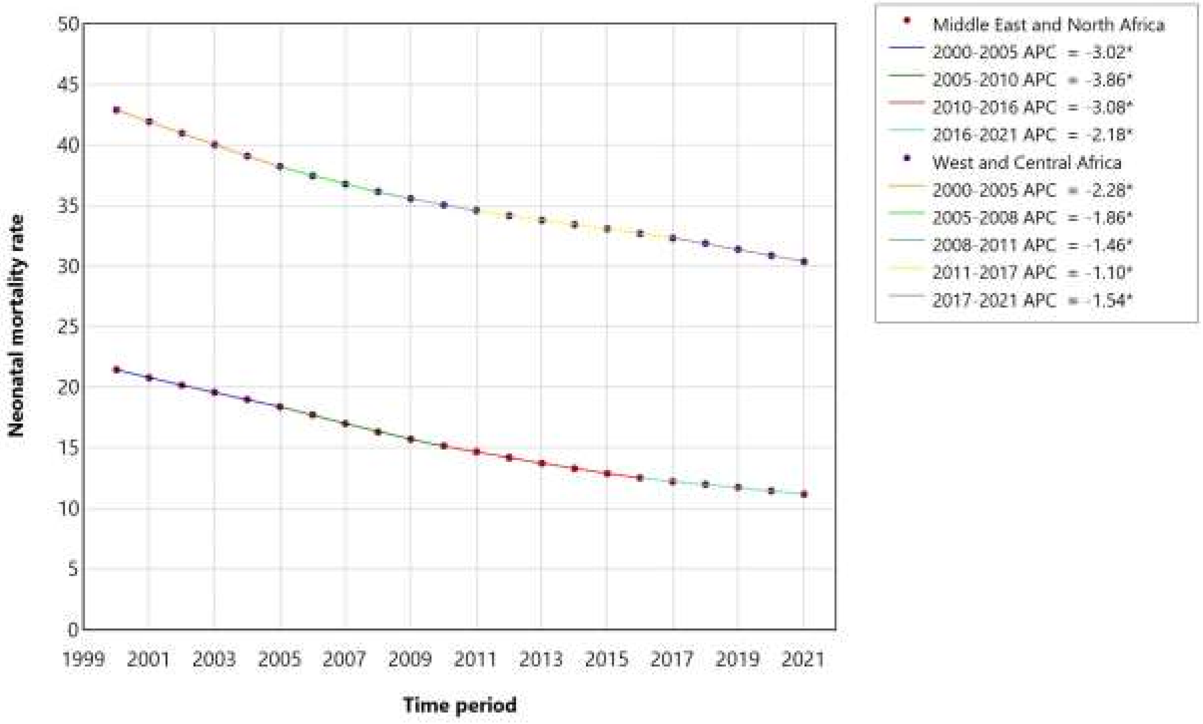

* Indicates that the Annual Percent Change (APC) is significantly different from zero at the alpha = 0.05 level.
Final Selected Model: Middle East and North Africa - 3 Joinpoints, West and Central Africa - 4 Joinpoints. Rejected Parallelism.

Middle East and North Africa: 3 Joinpoints versus Sub-Saharan Africa: 4 Joinpoints

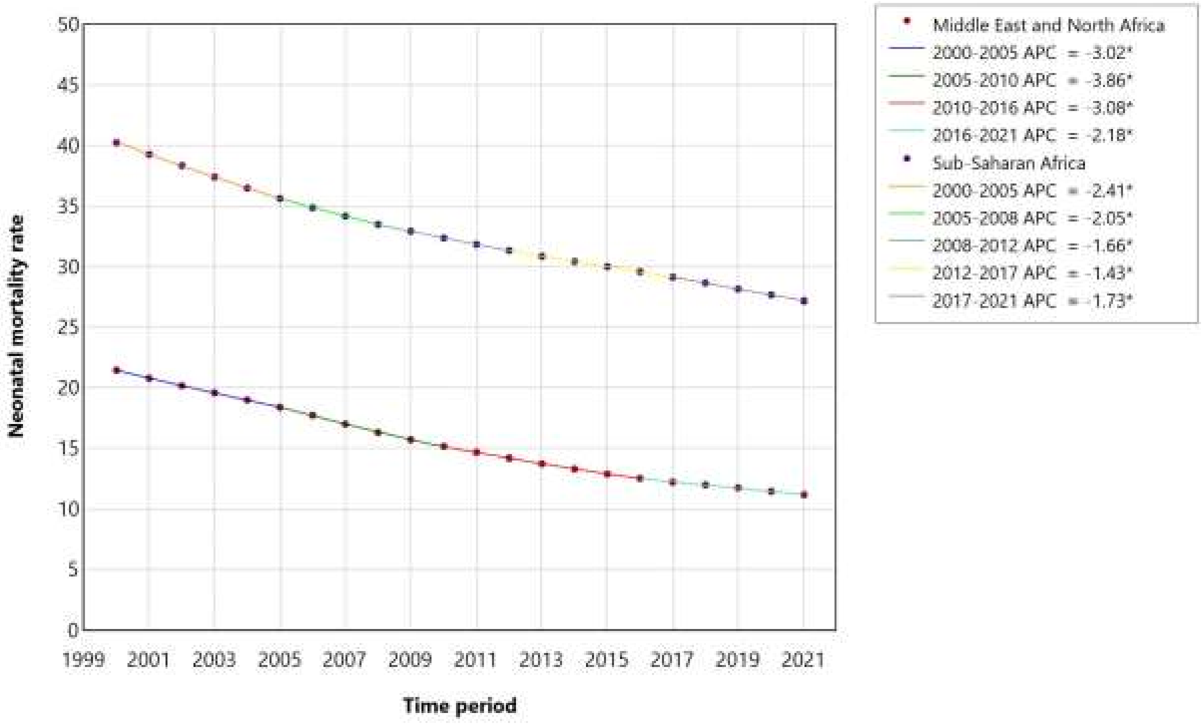

* Indicates that the Annual Percent Change (APC) is significantly different from zero at the alpha = 0.05 level.
Final Selected Model: Middle East and North Africa - 3 Joinpoints, Sub-Saharan Africa - 4 Joinpoints. Rejected Parallelism.

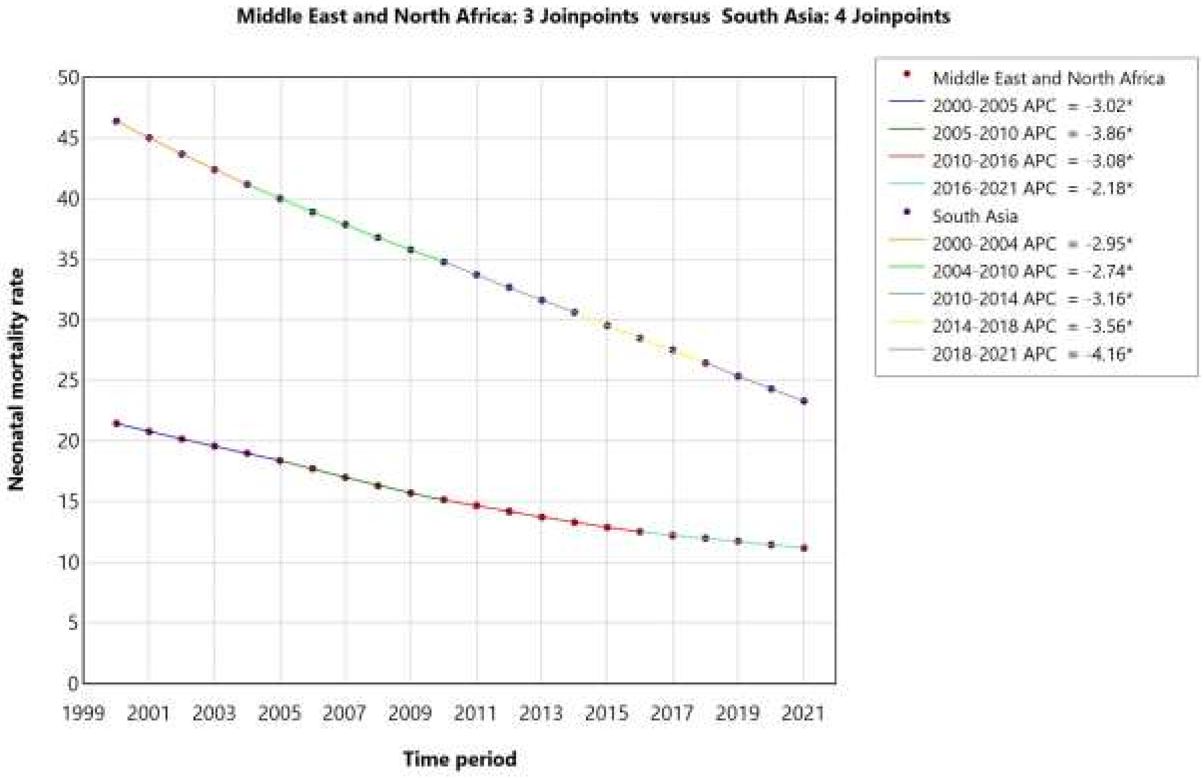

* Indicates that the Annual Percent Change (APC) is significantly different from zero at the alpha = 0.05 level.
Final Selected Model: Middle East and North Africa - 3 Joinpoints, South Asia - 4 Joinpoints. Rejected Parallelism.

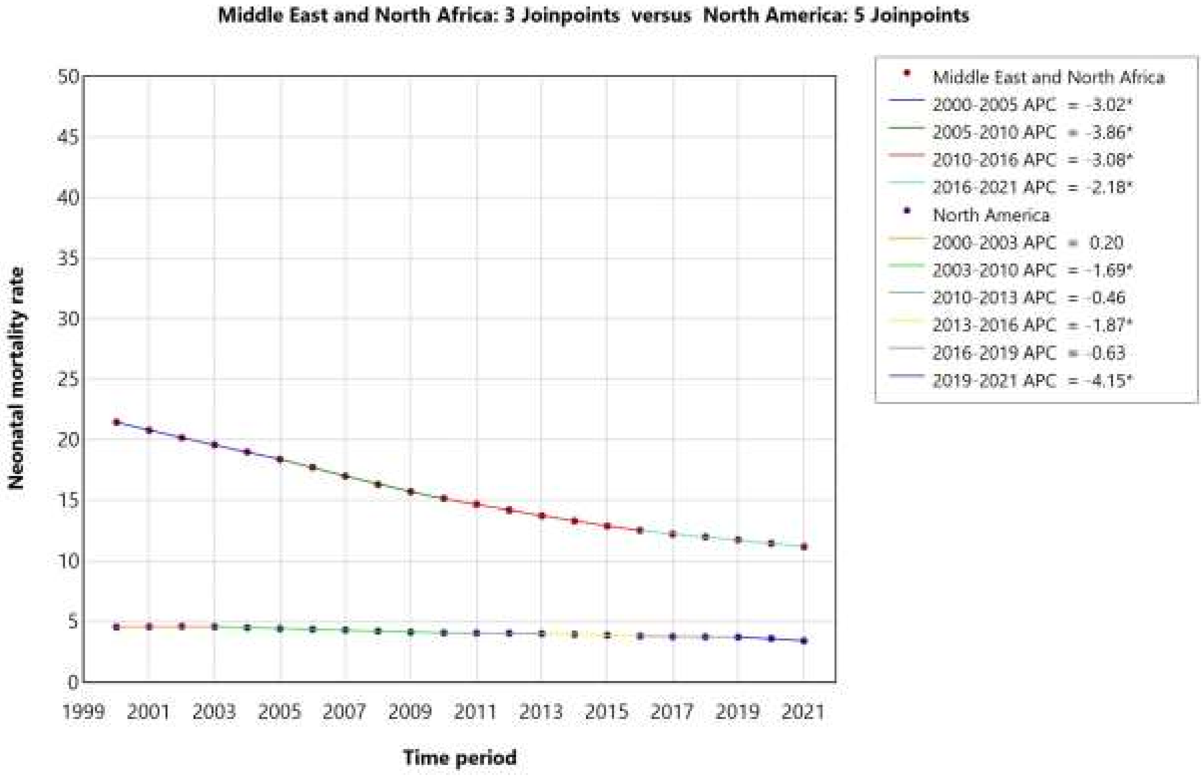

* Indicates that the Annual Percent Change (APC) is significantly different from zero at the alpha = 0.05 level.
Final Selected Model: Middle East and North Africa - 3 Joinpoints, North America - 5 Joinpoints. Rejected Parallelism.

Latin America and the Caribbean: 3 Joinpoints versus Western Europe: 4 Joinpoints

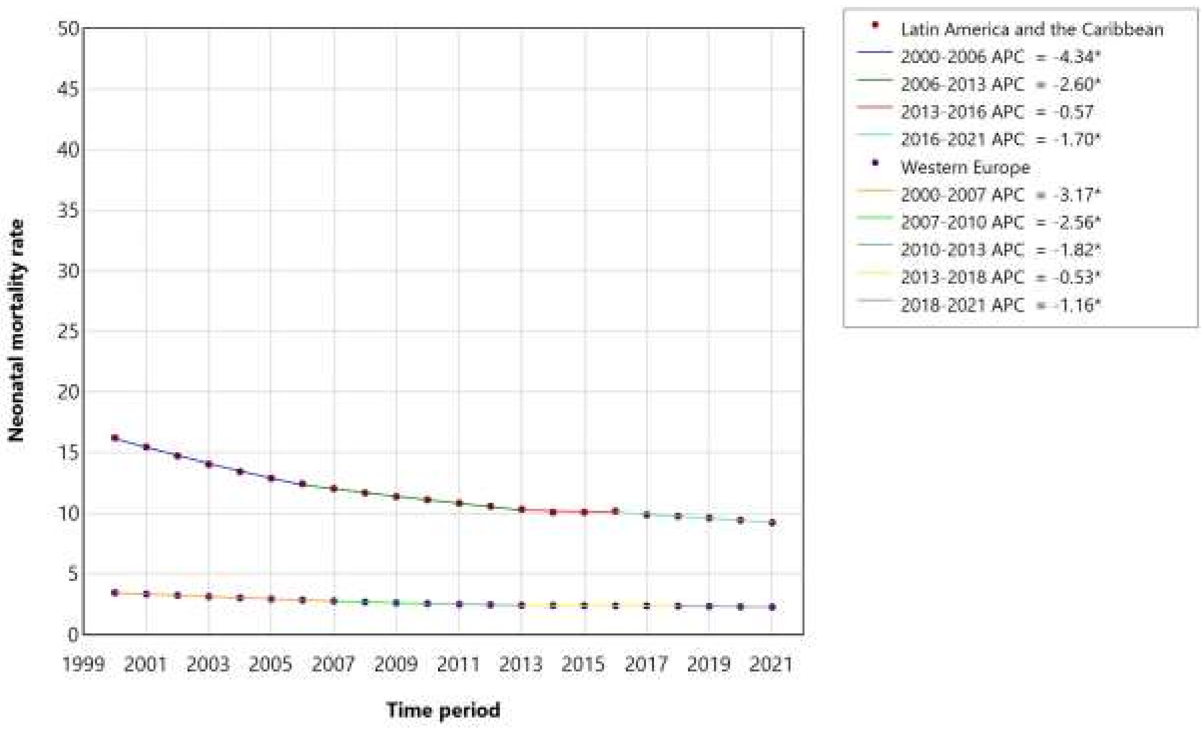

* Indicates that the Annual Percent Change (APC) is significantly different from zero at the alpha = 0.05 level.
Final Selected Model: Latin America and the Caribbean - 3 Joinpoints, Western Europe - 4 Joinpoints. Rejected Parallelism.

Latin America and the Caribbean: 3 Joinpoints versus West and Central Africa: 4 Joinpoints

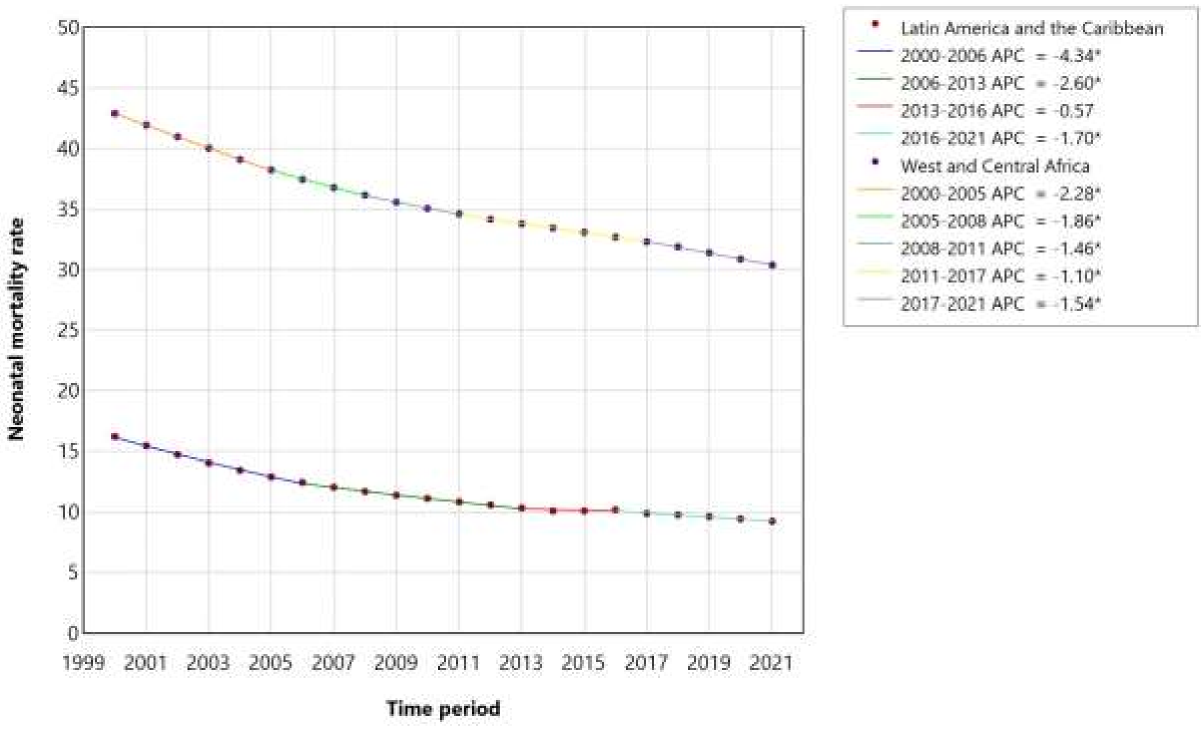

* Indicates that the Annual Percent Change (APC) is significantly different from zero at the alpha = 0.05 level.
Final Selected Model: Latin America and the Caribbean - 3 Joinpoints, West and Central Africa - 4 Joinpoints. Rejected Parallelism.

Latin America and the Caribbean: 3 Joinpoints versus Sub-Saharan Africa: 4 Joinpoints

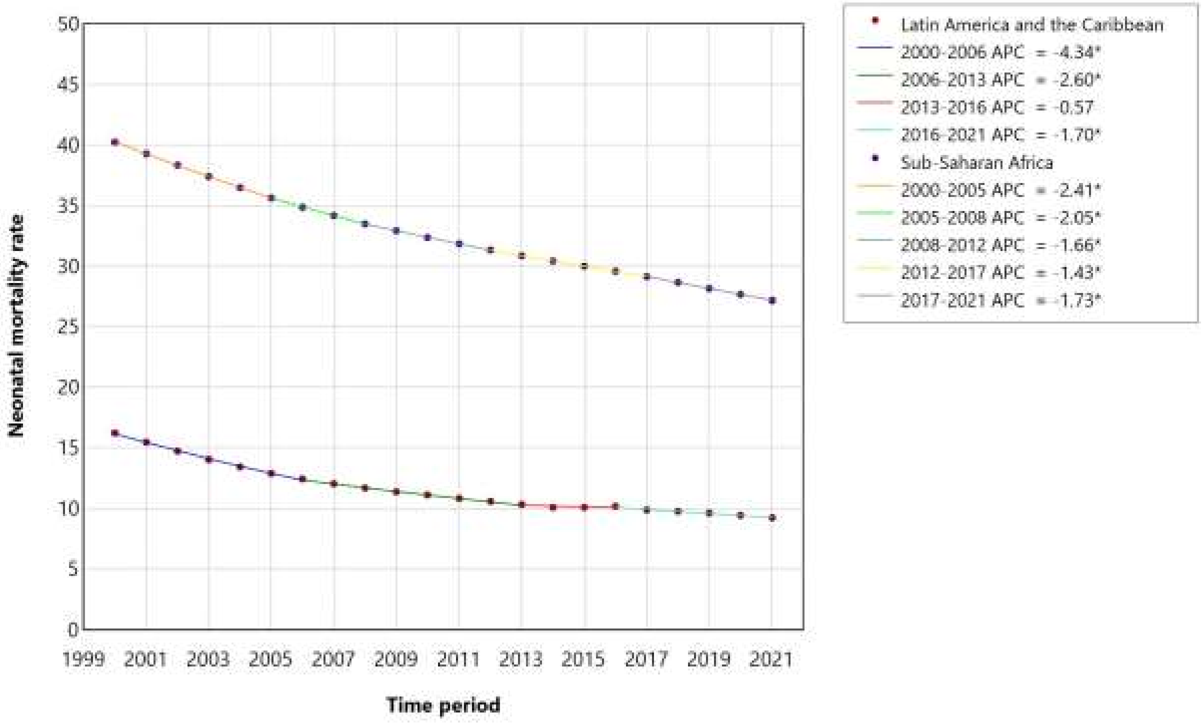

* Indicates that the Annual Percent Change (APC) is significantly different from zero at the alpha = 0.05 level.
Final Selected Model: Latin America and the Caribbean - 3 Joinpoints, Sub-Saharan Africa - 4 Joinpoints. Rejected Parallelism.

Latin America and the Caribbean: 3 Joinpoints versus South Asia: 4 Joinpoints

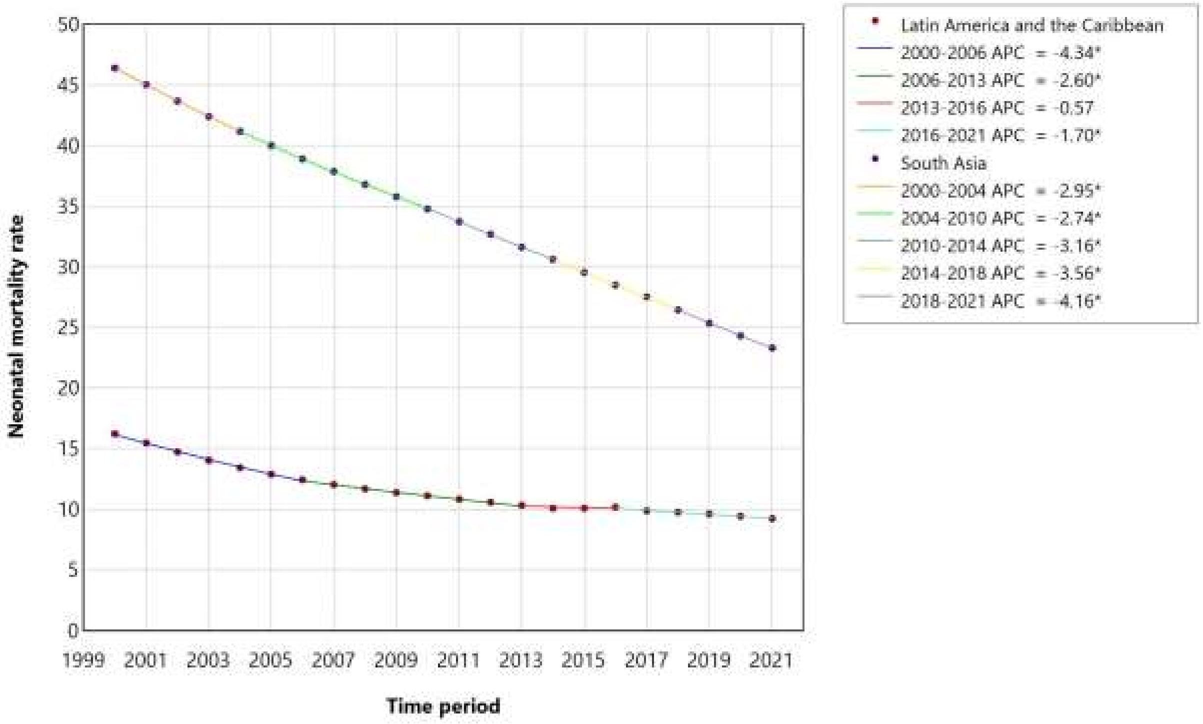

* Indicates that the Annual Percent Change (APC) is significantly different from zero at the alpha = 0.05 level.
Final Selected Model: Latin America and the Caribbean - 3 Joinpoints, South Asia - 4 Joinpoints. Rejected Parallelism.

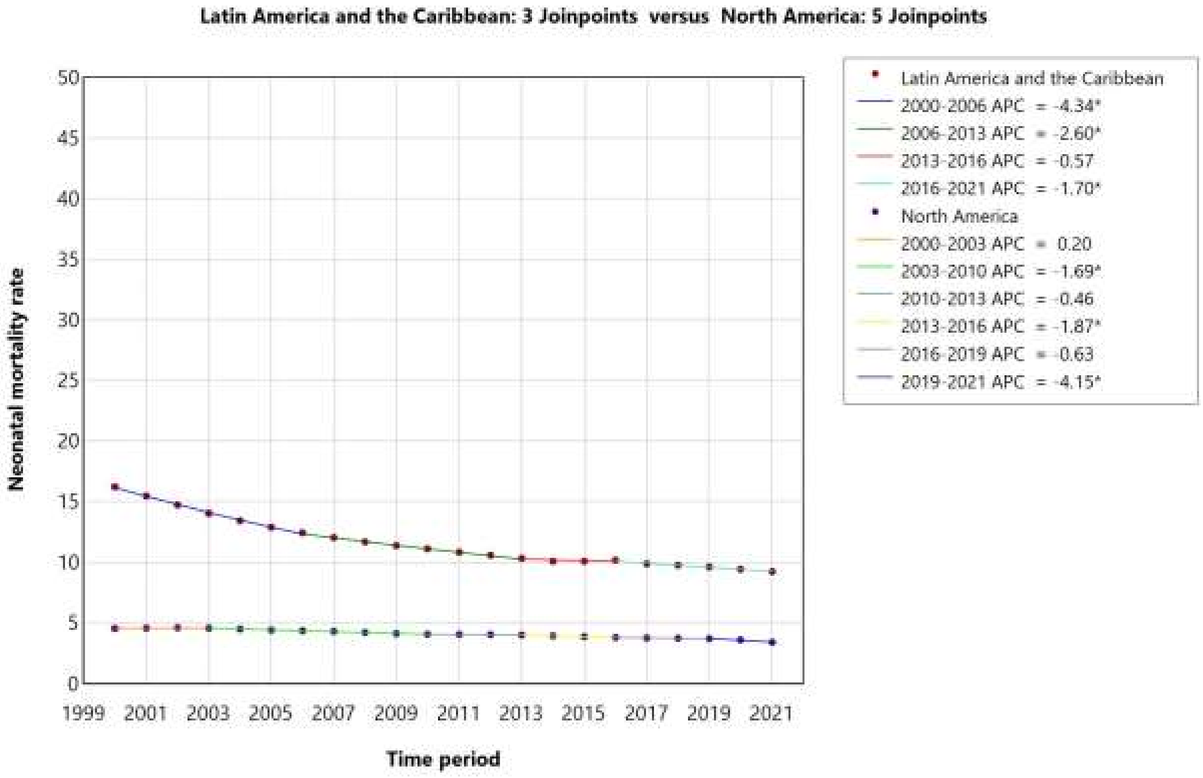

* Indicates that the Annual Percent Change (APC) is significantly different from zero at the alpha = 0.05 level.
Final Selected Model: Latin America and the Caribbean - 3 Joinpoints, North America - 5 Joinpoints. Rejected Parallelism.

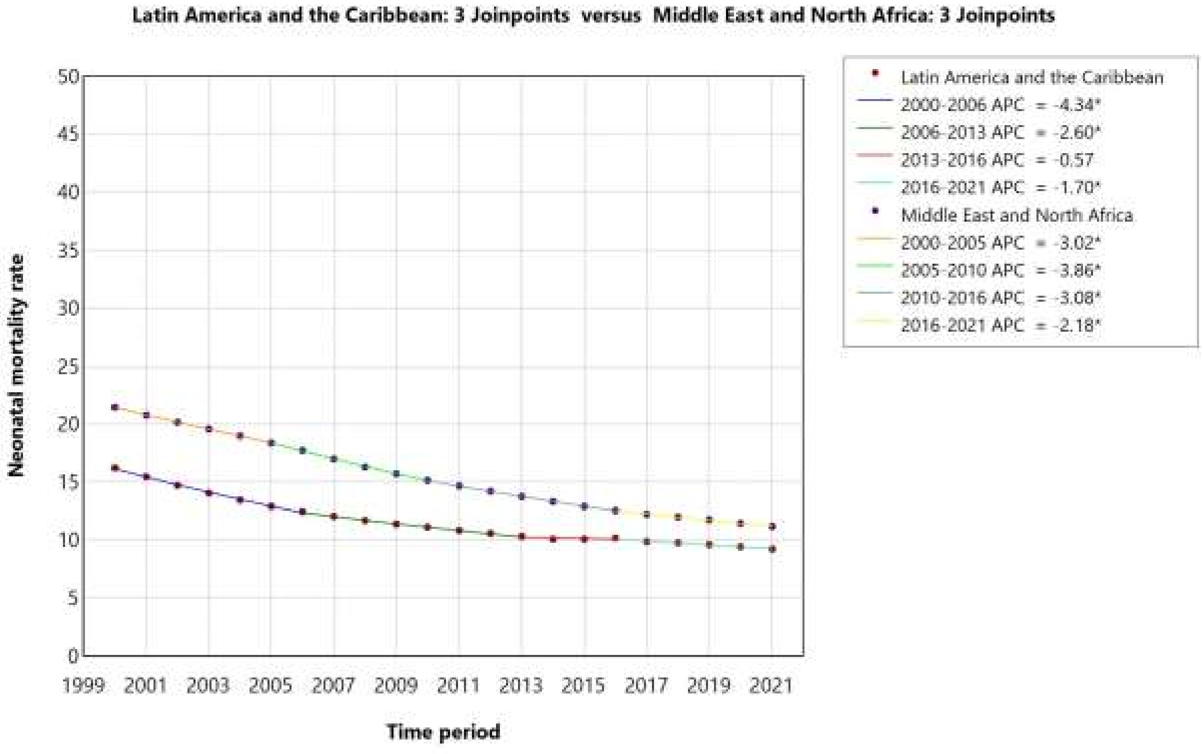

* Indicates that the Annual Percent Change (APC) is significantly different from zero at the alpha = 0.05 level.
Final Selected Model: Latin America and the Caribbean - 3 Joinpoints, Middle East and North Africa - 3 Joinpoints. Rejected Parallelism.

Europe and Central Asia: 2 Joinpoints versus Western Europe: 4 Joinpoints

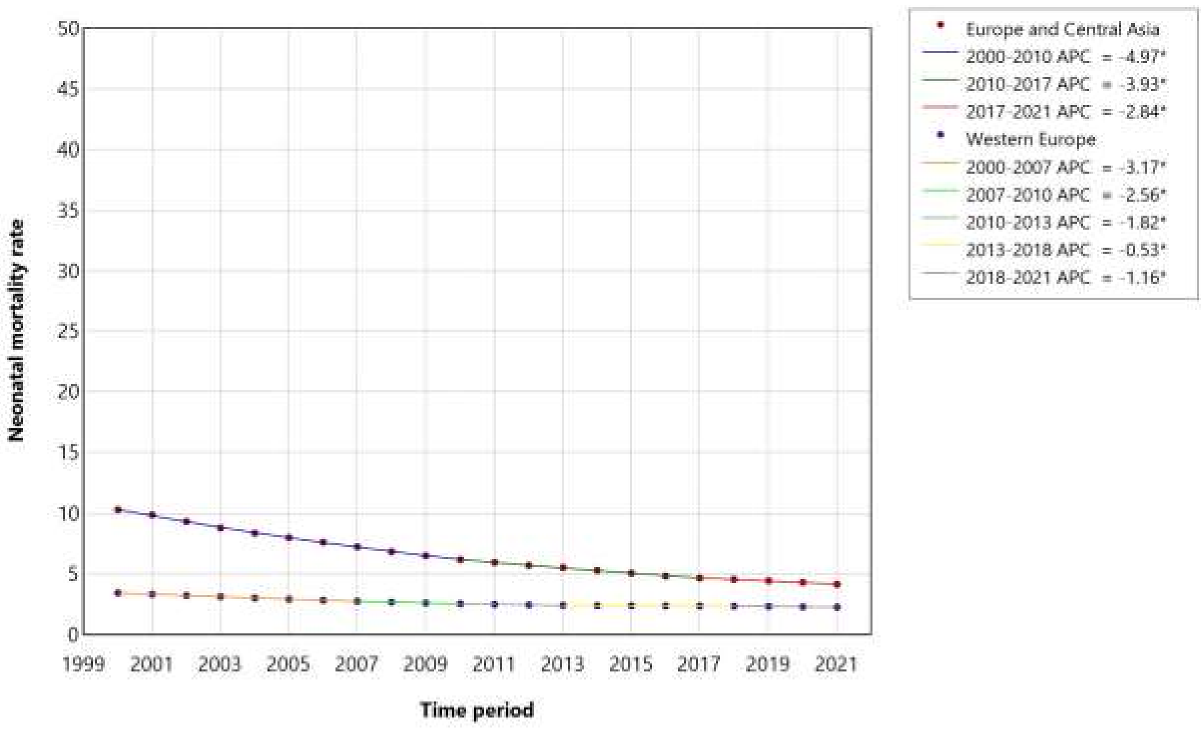

* Indicates that the Annual Percent Change (APC) is significantly different from zero at the alpha = 0.05 level.
Final Selected Model: Europe and Central Asia - 2 Joinpoints, Western Europe - 4 Joinpoints. Rejected Parallelism.

Europe and Central Asia: 2 Joinpoints versus West and Central Africa: 4 Joinpoints

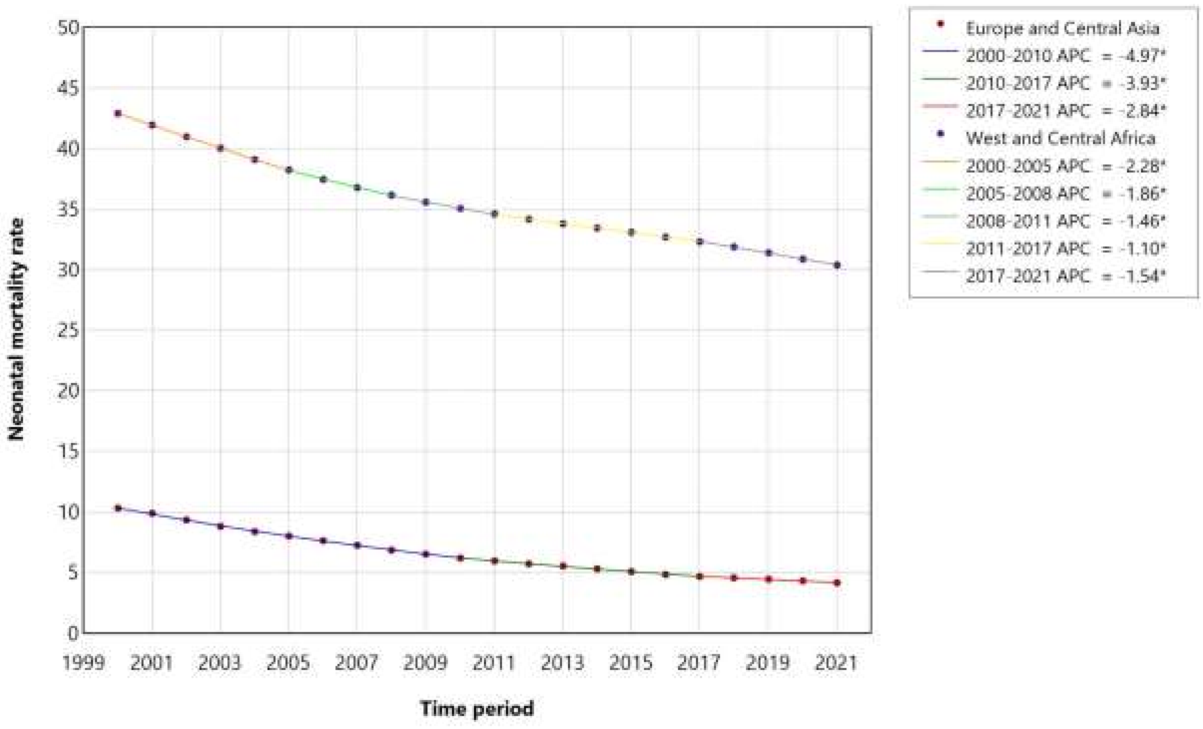

* Indicates that the Annual Percent Change (APC) is significantly different from zero at the alpha = 0.05 level.
Final Selected Model: Europe and Central Asia - 2 Joinpoints, West and Central Africa - 4 Joinpoints. Rejected Parallelism.

Europe and Central Asia: 2 Joinpoints versus Sub-Saharan Africa: 4 Joinpoints

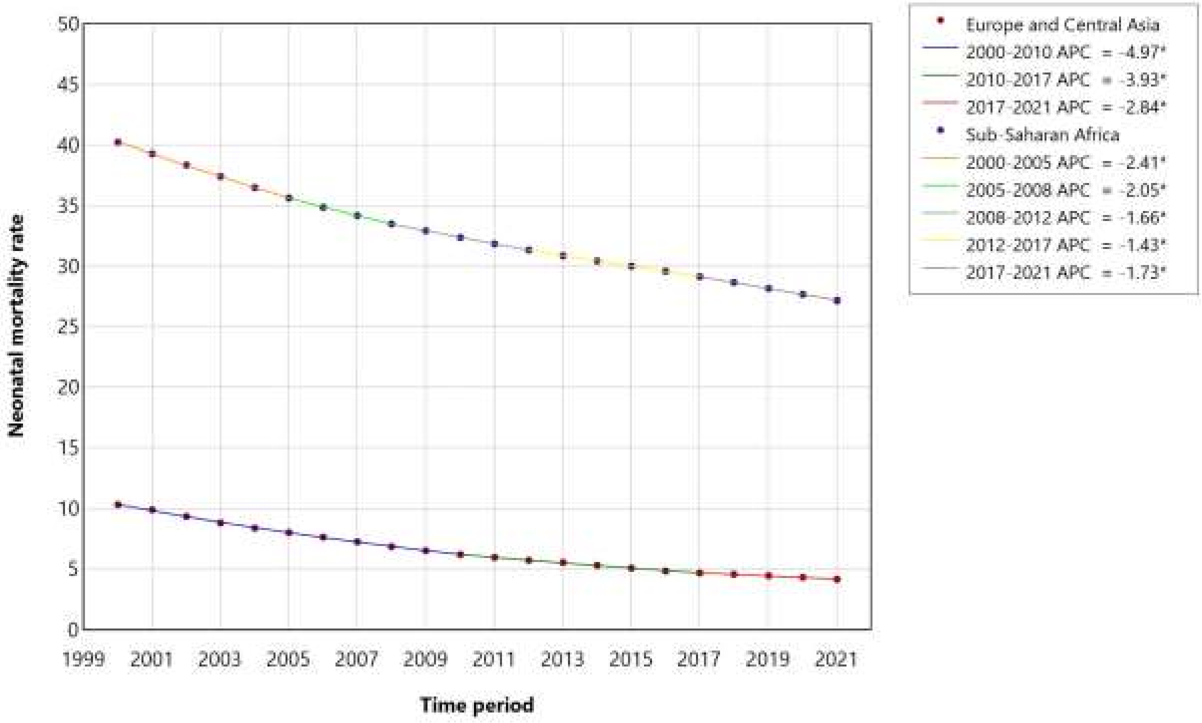

* Indicates that the Annual Percent Change (APC) is significantly different from zero at the alpha = 0.05 level.
Final Selected Model: Europe and Central Asia - 2 Joinpoints, Sub-Saharan Africa - 4 Joinpoints. Rejected Parallelism.

Europe and Central Asia: 2 Joinpoints versus South Asia: 4 Joinpoints

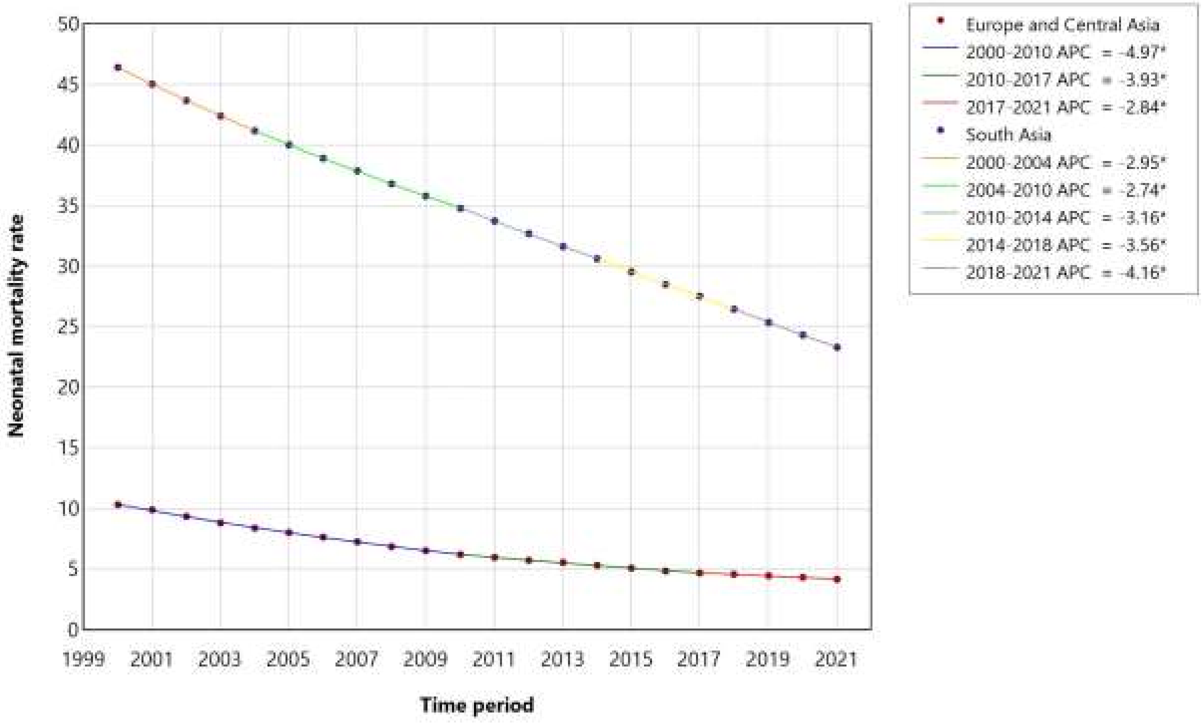

* Indicates that the Annual Percent Change (APC) is significantly different from zero at the alpha = 0.05 level.
Final Selected Model: Europe and Central Asia - 2 Joinpoints, South Asia - 4 Joinpoints. Rejected Parallelism.

Europe and Central Asia: 2 Joinpoints versus North America: 5 Joinpoints

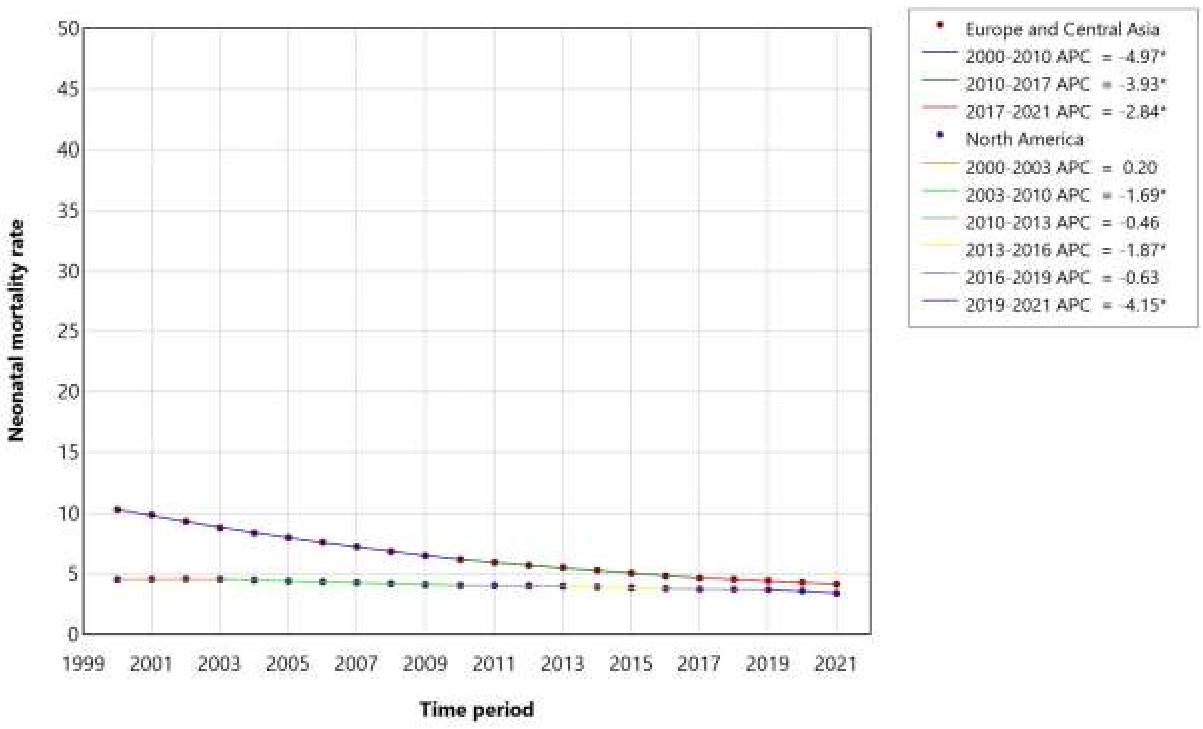

* Indicates that the Annual Percent Change (APC) is significantly different from zero at the alpha = 0.05 level.
Final Selected Model: Europe and Central Asia - 2 Joinpoints, North America - 5 Joinpoints. Rejected Parallelism.

Europe and Central Asia: 2 Joinpoints versus Middle East and North Africa: 3 Joinpoints

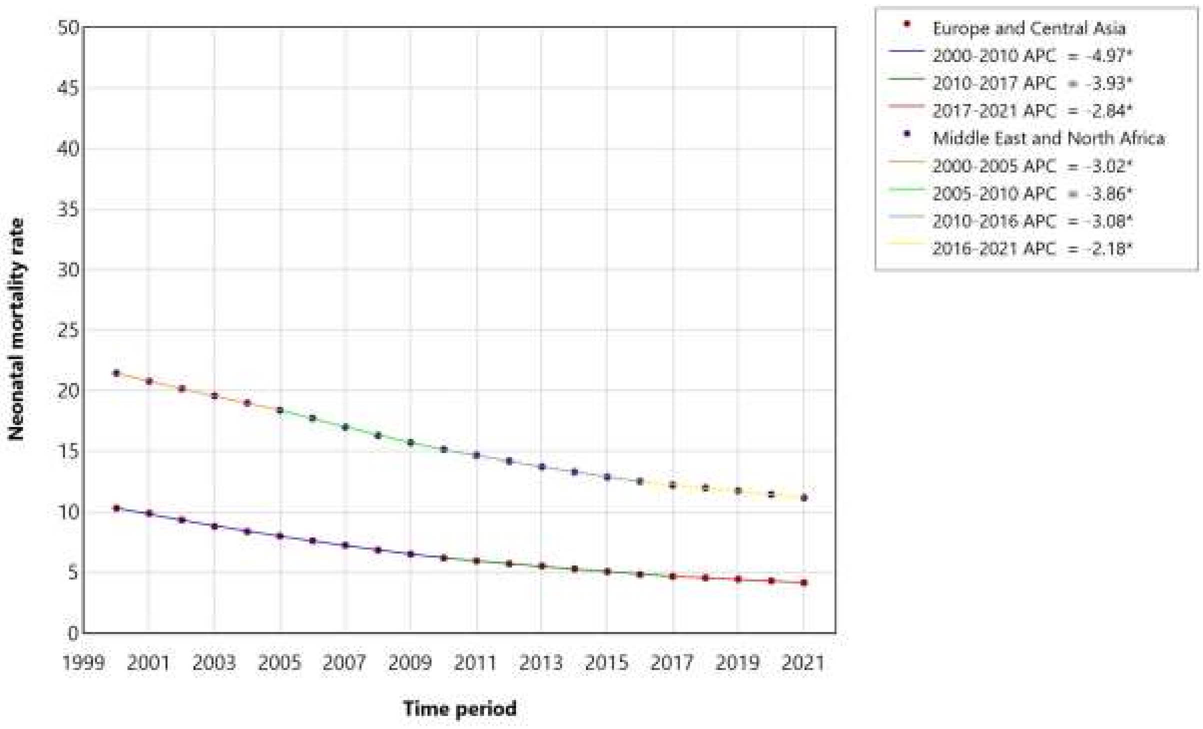

* Indicates that the Annual Percent Change (APC) is significantly different from zero at the alpha = 0.05 level.
Final Selected Model: Europe and Central Asia - 2 Joinpoints, Middle East and North Africa - 3 Joinpoints. Rejected Parallelism.

Europe and Central Asia: 2 Joinpoints versus Latin America and the Caribbean: 3 Joinpoints

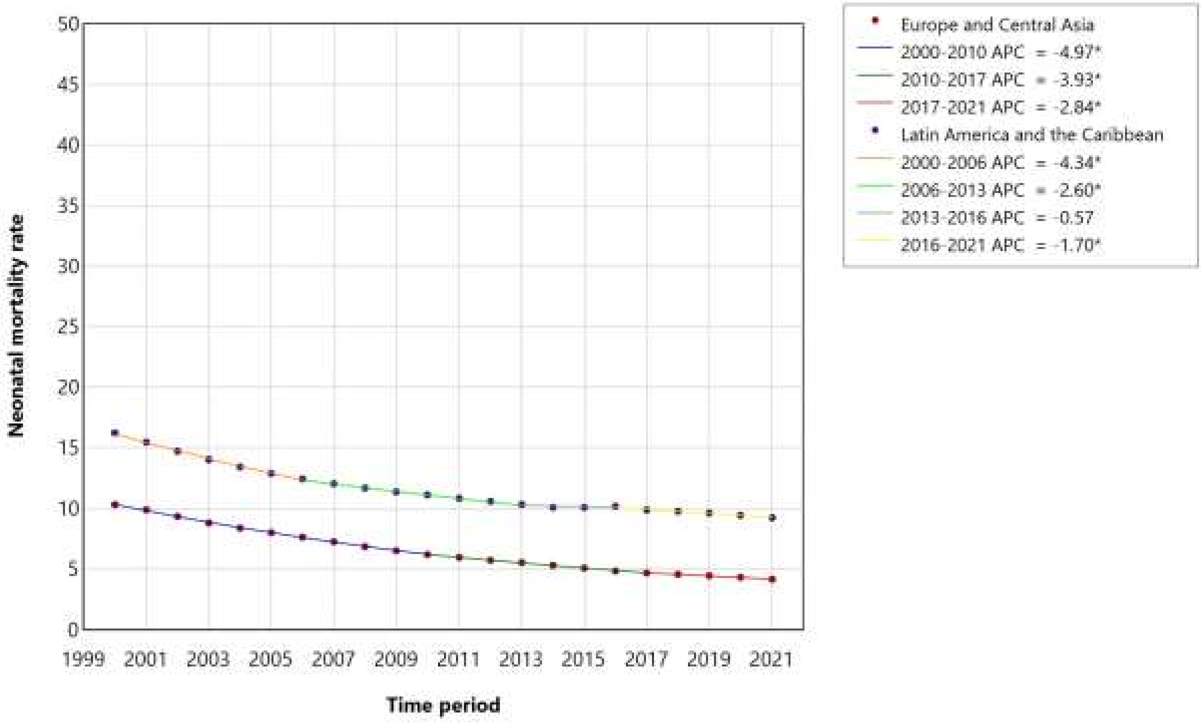

* Indicates that the Annual Percent Change (APC) is significantly different from zero at the alpha = 0.05 level.
Final Selected Model: Europe and Central Asia - 2 Joinpoints, Latin America and the Caribbean - 3 Joinpoints. Rejected Parallelism.

Eastern Europe and Central Asia: 5 Joinpoints versus Western Europe: 4 Joinpoints

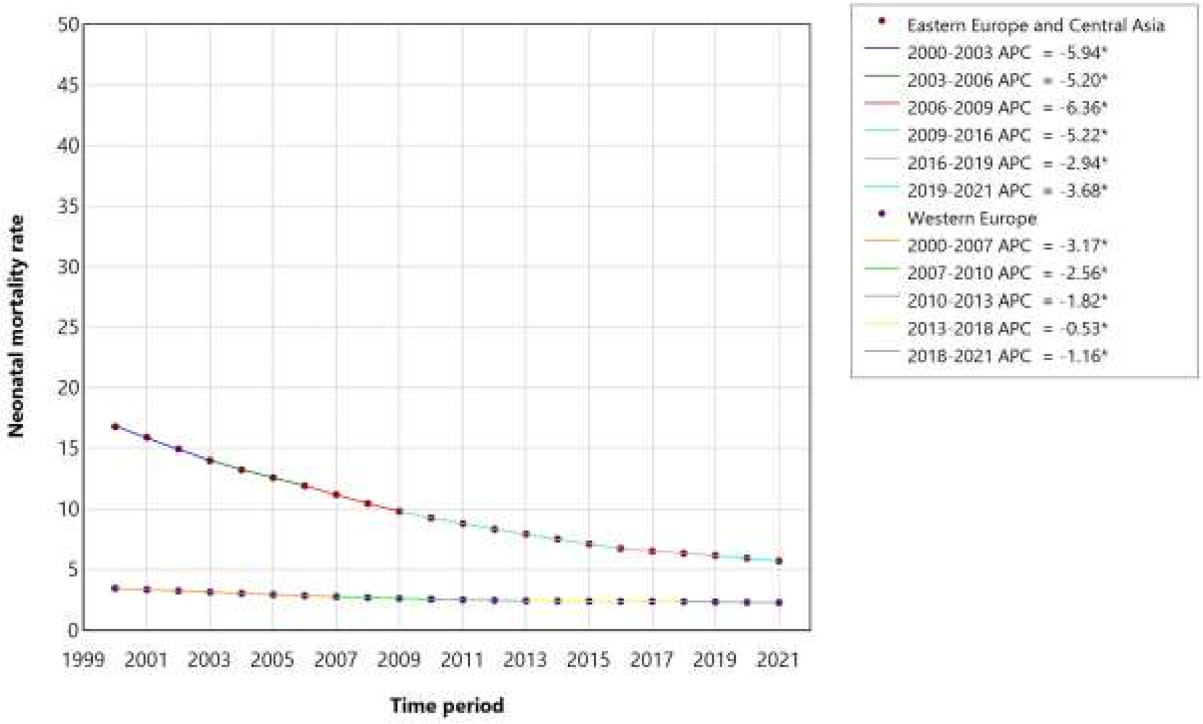

* Indicates that the Annual Percent Change (APC) is significantly different from zero at the alpha = 0.05 level.
Final Selected Model: Eastern Europe and Central Asia - 5 Joinpoints, Western Europe - 4 Joinpoints. Rejected Parallelism.

Eastern Europe and Central Asia: 5 Joinpoints versus West and Central Africa: 4 Joinpoints

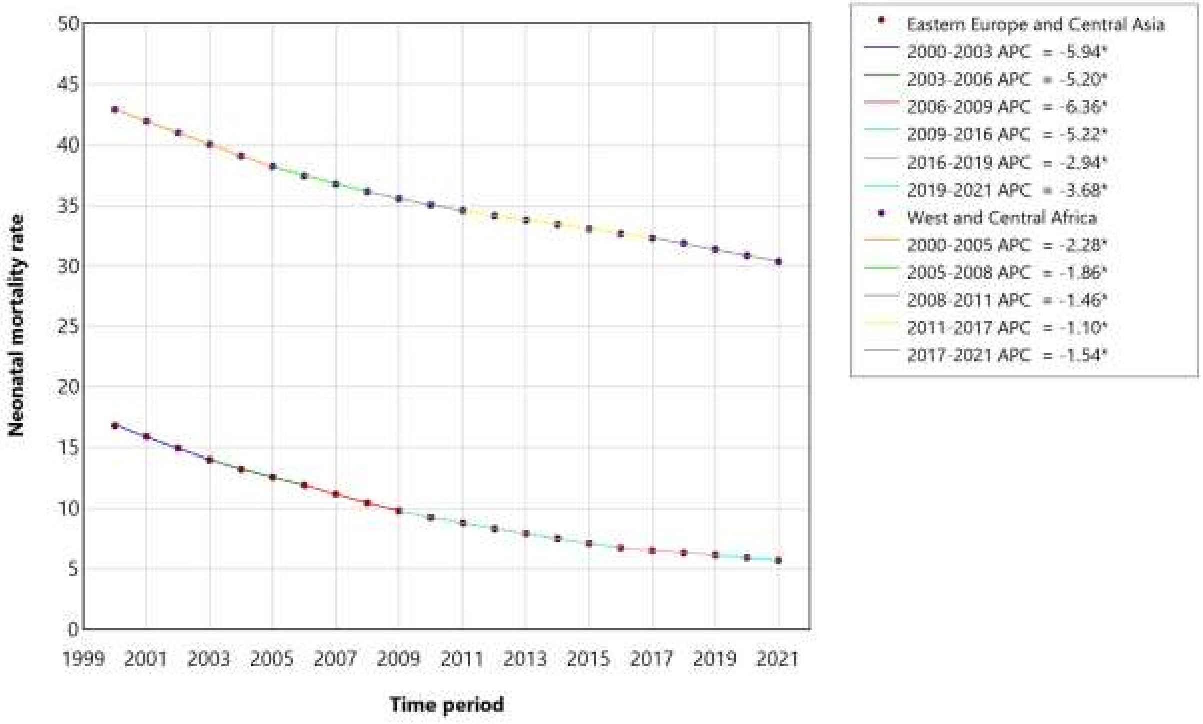

* Indicates that the Annual Percent Change (APC) is significantly different from zero at the alpha = 0.05 level.
Final Selected Model: Eastern Europe and Central Asia - 5 Joinpoints, West and Central Africa - 4 Joinpoints. Rejected Parallelism.

Eastern Europe and Central Asia: 5 Joinpoints versus Sub-Saharan Africa: 4 Joinpoints

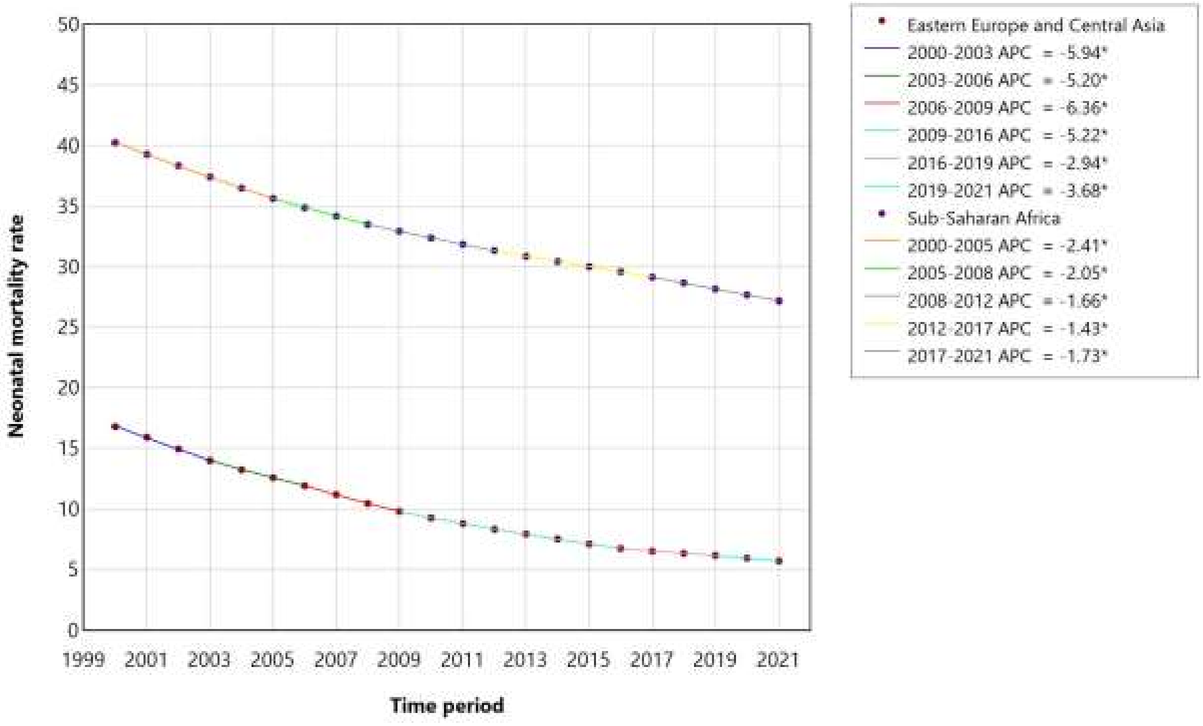

* Indicates that the Annual Percent Change (APC) is significantly different from zero at the alpha = 0.05 level.
Final Selected Model: Eastern Europe and Central Asia - 5 Joinpoints, Sub-Saharan Africa - 4 Joinpoints. Rejected Parallelism.

Eastern Europe and Central Asia: 5 Joinpoints versus South Asia: 4 Joinpoints

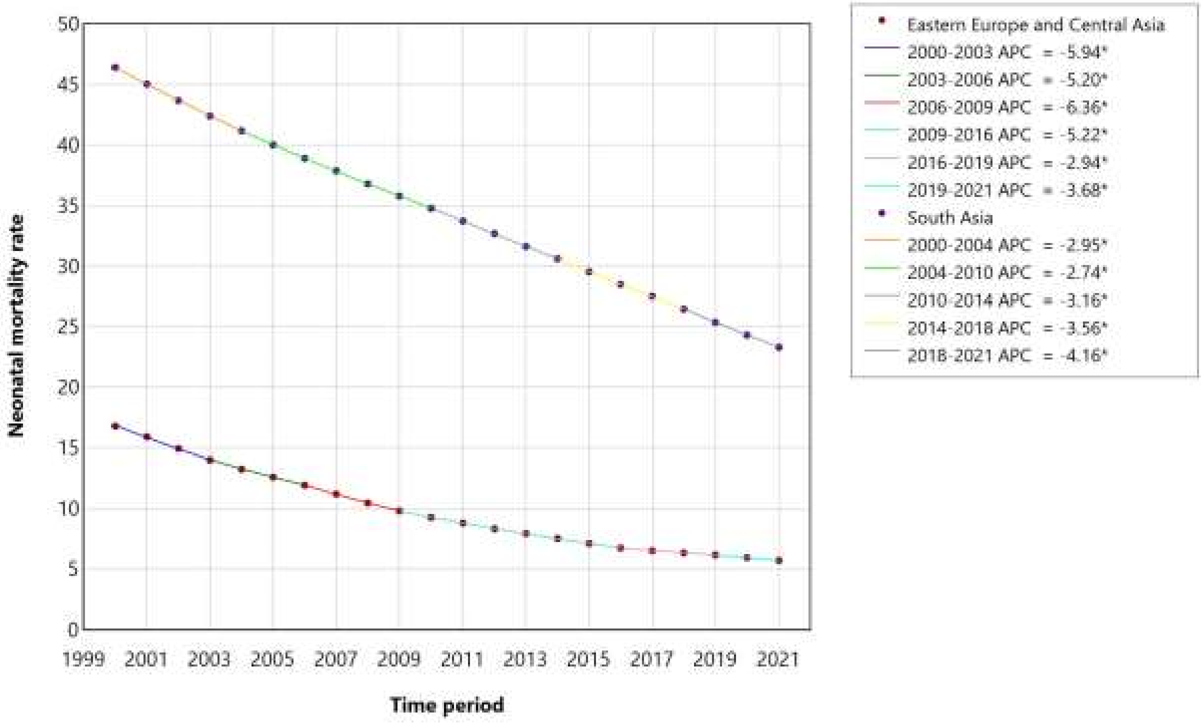

* Indicates that the Annual Percent Change (APC) is significantly different from zero at the alpha = 0.05 level.
Final Selected Model: Eastern Europe and Central Asia - 5 Joinpoints, South Asia - 4 Joinpoints. Rejected Parallelism.

Eastern Europe and Central Asia: 5 Joinpoints versus North America: 5 Joinpoints

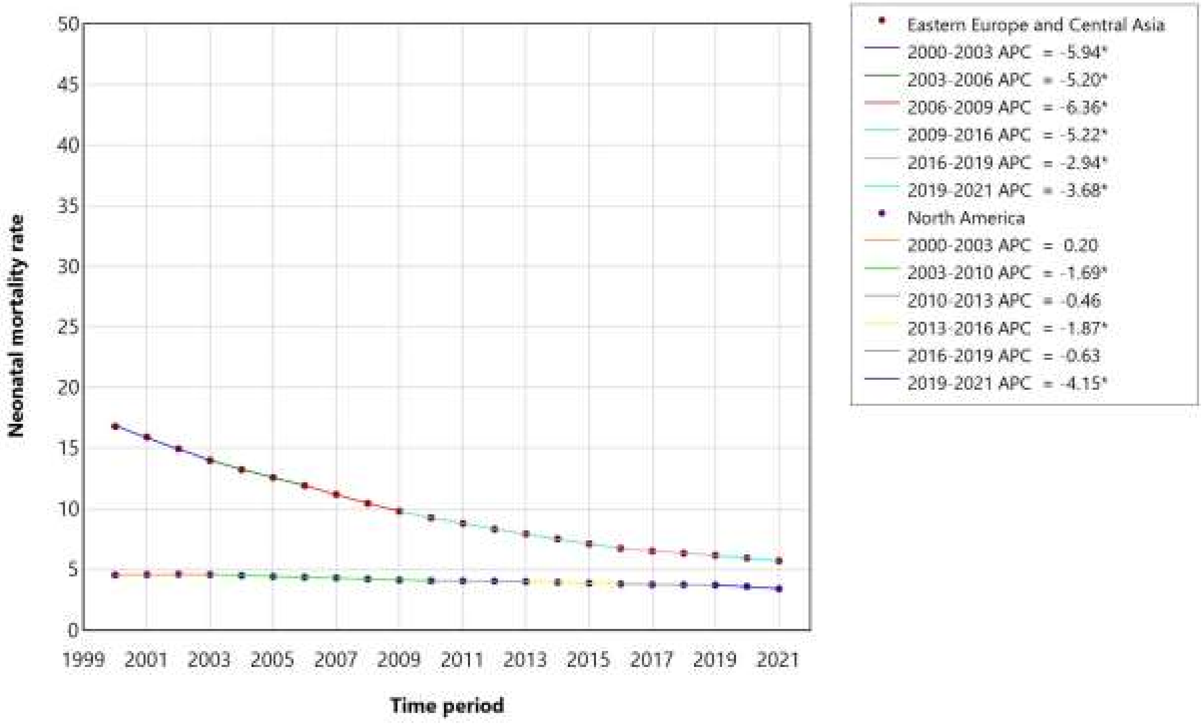

* Indicates that the Annual Percent Change (APC) is significantly different from zero at the alpha = 0.05 level.
Final Selected Model: Eastern Europe and Central Asia - 5 Joinpoints, North America - 5 Joinpoints. Rejected Parallelism.

Eastern Europe and Central Asia: 5 Joinpoints versus Middle East and North Africa: 3 Joinpoints

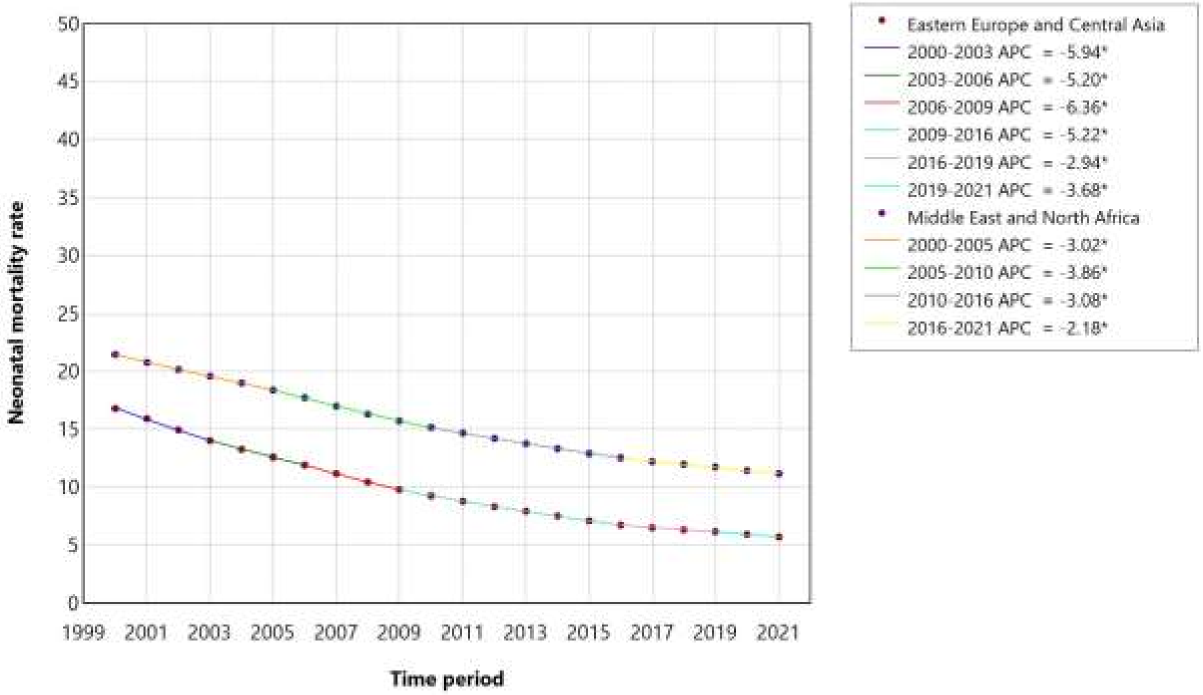

* Indicates that the Annual Percent Change (APC) is significantly different from zero at the alpha = 0.05 level.
Final Selected Model: Eastern Europe and Central Asia - 5 Joinpoints, Middle East and North Africa - 3 Joinpoints. Rejected Parallelism.

Eastern Europe and Central Asia: 5 Joinpoints versus Latin America and the Caribbean: 3 Joinpoints

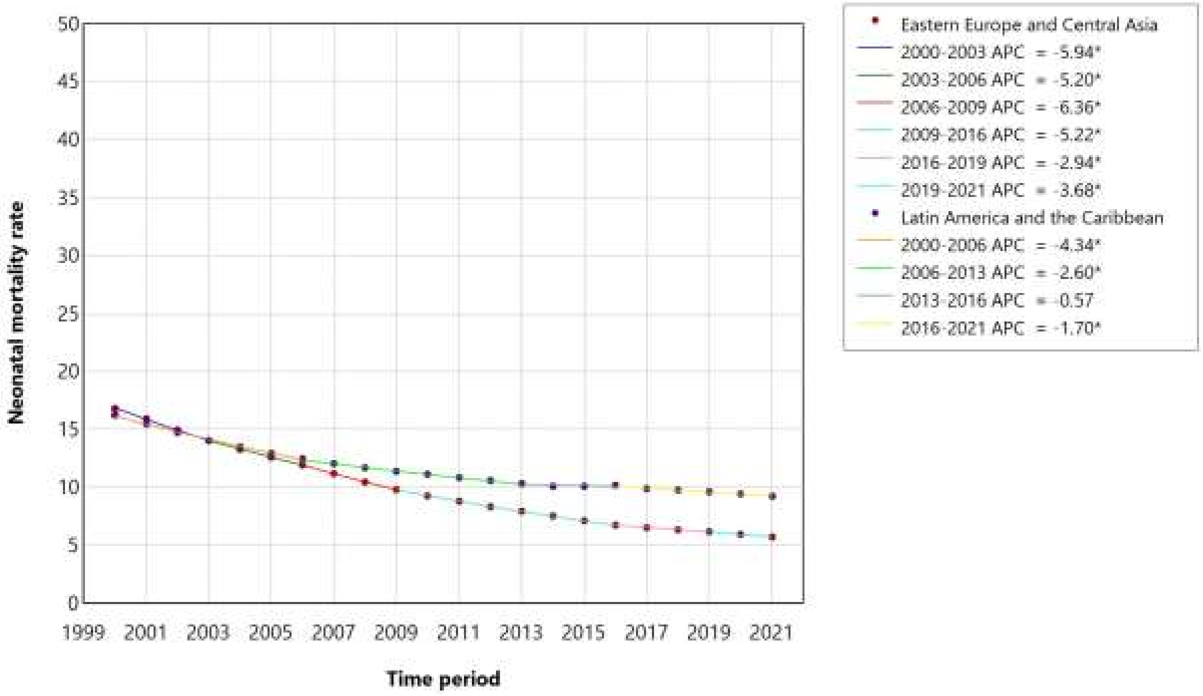

* Indicates that the Annual Percent Change (APC) is significantly different from zero at the alpha = 0.05 level.
Final Selected Model: Eastern Europe and Central Asia - 5 Joinpoints, Latin America and the Caribbean - 3 Joinpoints. Rejected Parallelism.

Eastern Europe and Central Asia: 5 Joinpoints versus Europe and Central Asia: 2 Joinpoints

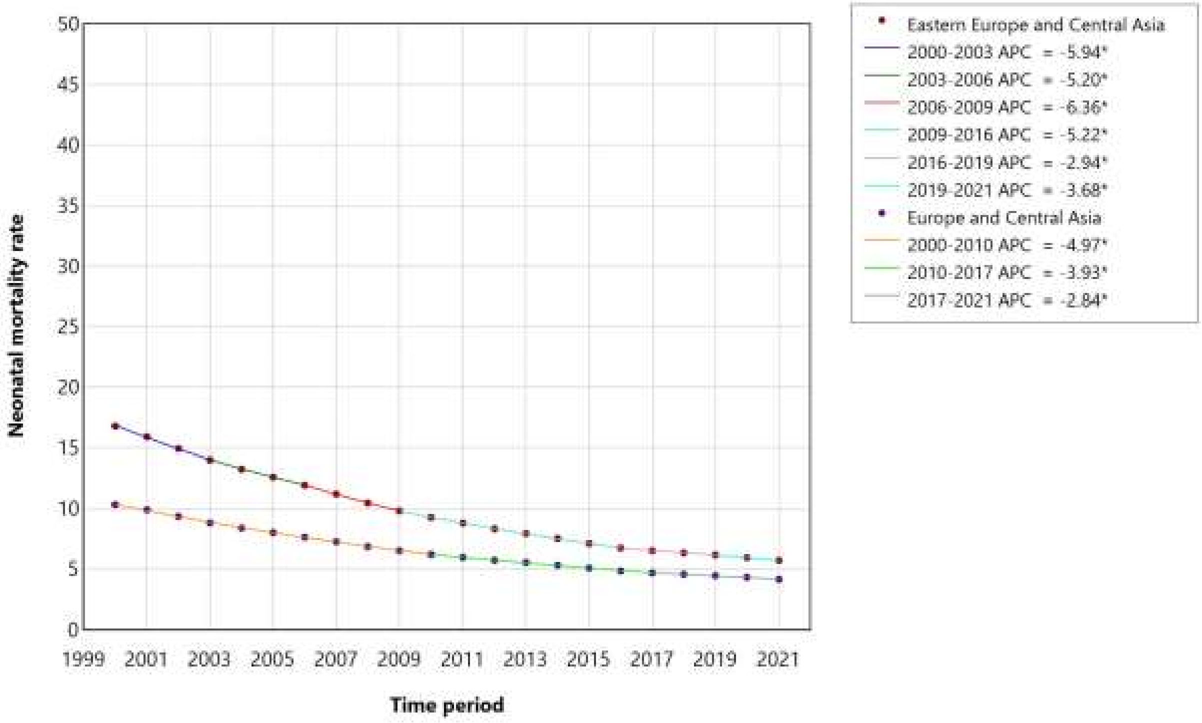

* Indicates that the Annual Percent Change (APC) is significantly different from zero at the alpha = 0.05 level.
Final Selected Model: Eastern Europe and Central Asia - 5 Joinpoints, Europe and Central Asia - 2 Joinpoints. Rejected Parallelism.

Eastern and Southern Africa: 3 Joinpoints versus Western Europe: 4 Joinpoints

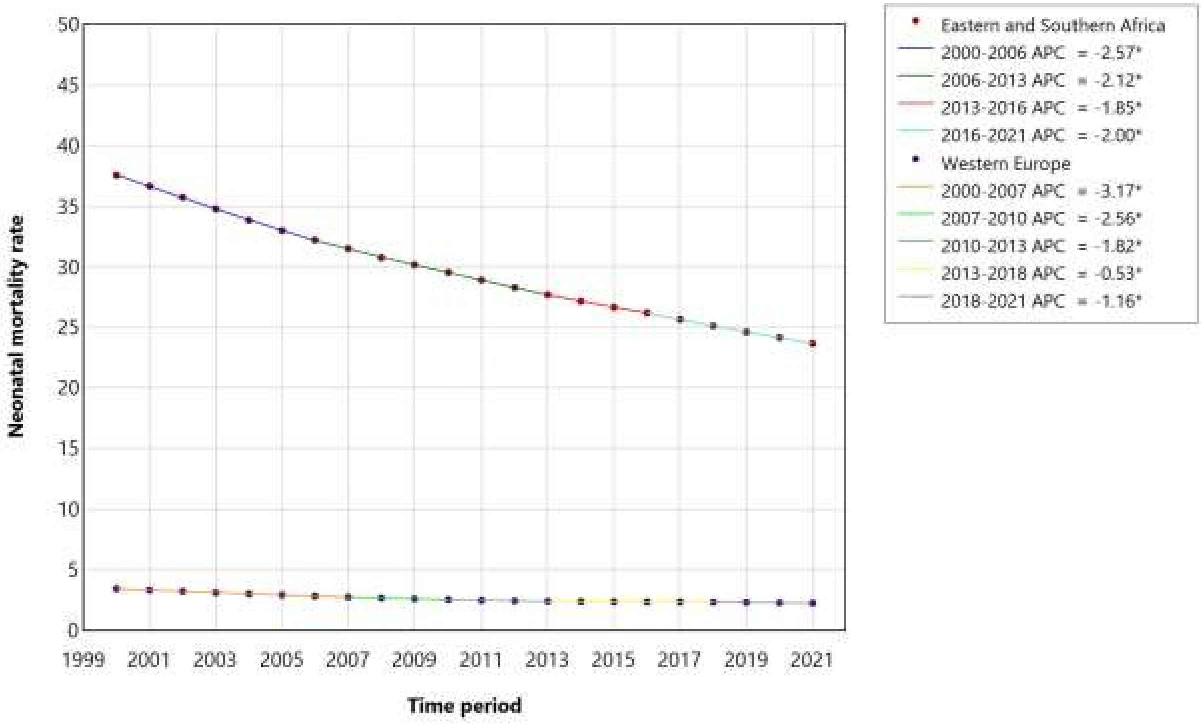

* Indicates that the Annual Percent Change (APC) is significantly different from zero at the alpha = 0.05 level.
Final Selected Model: Eastern and Southern Africa - 3 Joinpoints, Western Europe - 4 Joinpoints. Rejected Parallelism.

Eastern and Southern Africa: 3 Joinpoints versus West and Central Africa: 4 Joinpoints

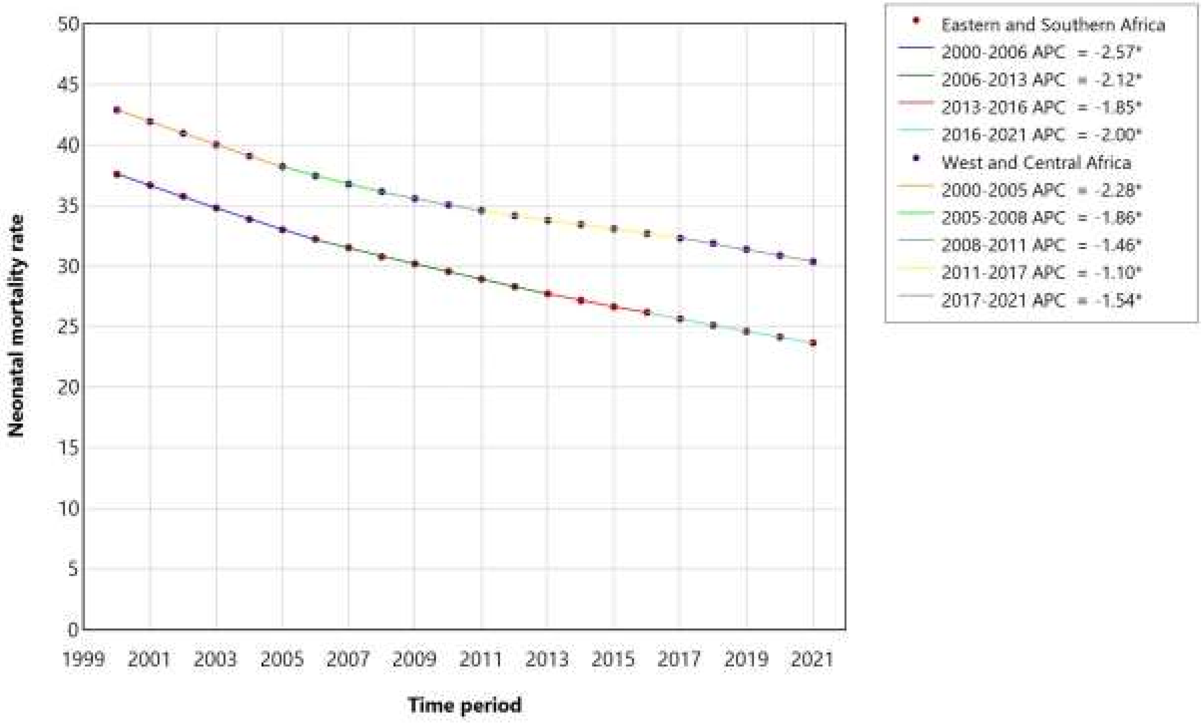

* Indicates that the Annual Percent Change (APC) is significantly different from zero at the alpha = 0.05 level.
Final Selected Model: Eastern and Southern Africa - 3 Joinpoints, West and Central Africa - 4 Joinpoints. Rejected Parallelism.

Eastern and Southern Africa: 3 Joinpoints versus Sub-Saharan Africa: 4 Joinpoints

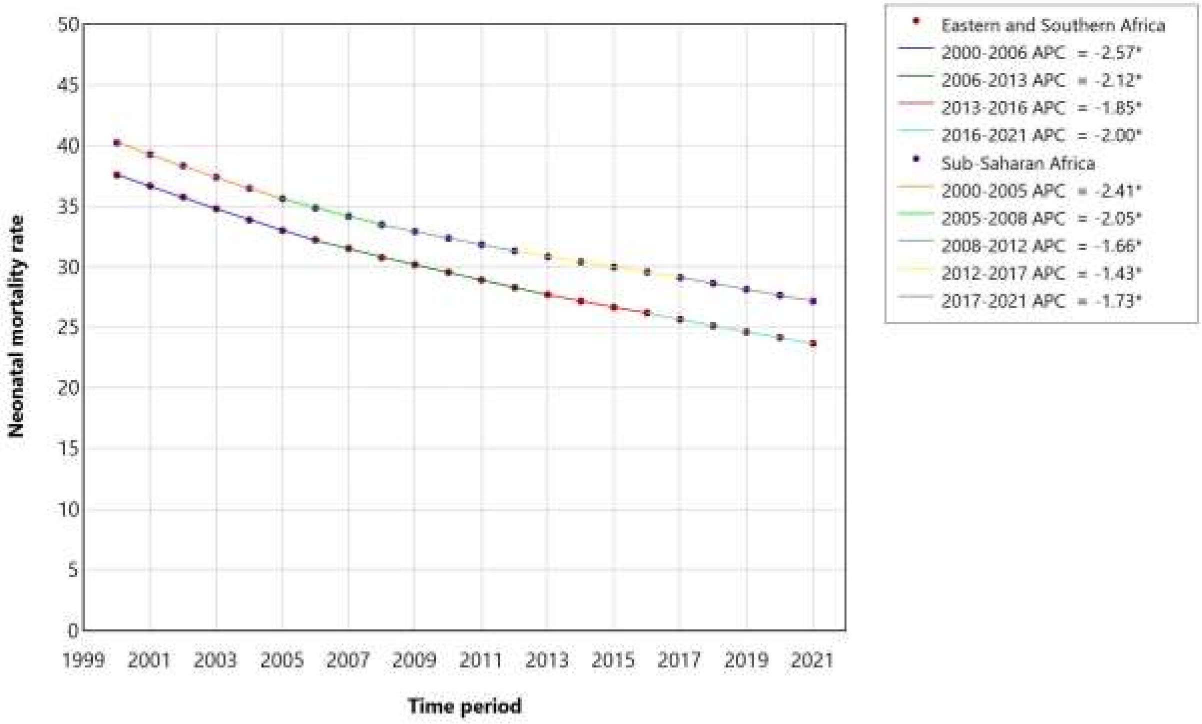

* Indicates that the Annual Percent Change (APC) is significantly different from zero at the alpha = 0.05 level.
Final Selected Model: Eastern and Southern Africa - 3 Joinpoints, Sub-Saharan Africa - 4 Joinpoints. Rejected Parallelism.

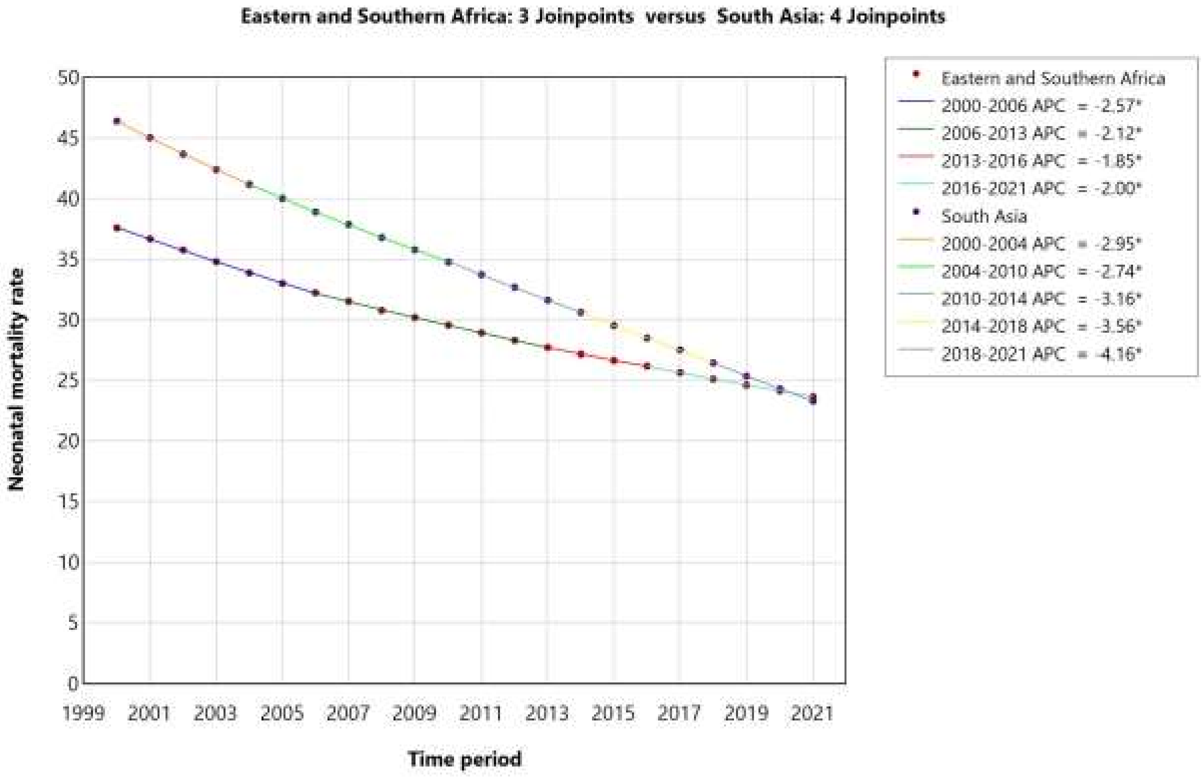

* Indicates that the Annual Percent Change (APC) is significantly different from zero at the alpha = 0.05 level.
Final Selected Model: Eastern and Southern Africa - 3 Joinpoints, South Asia - 4 Joinpoints. Rejected Parallelism.

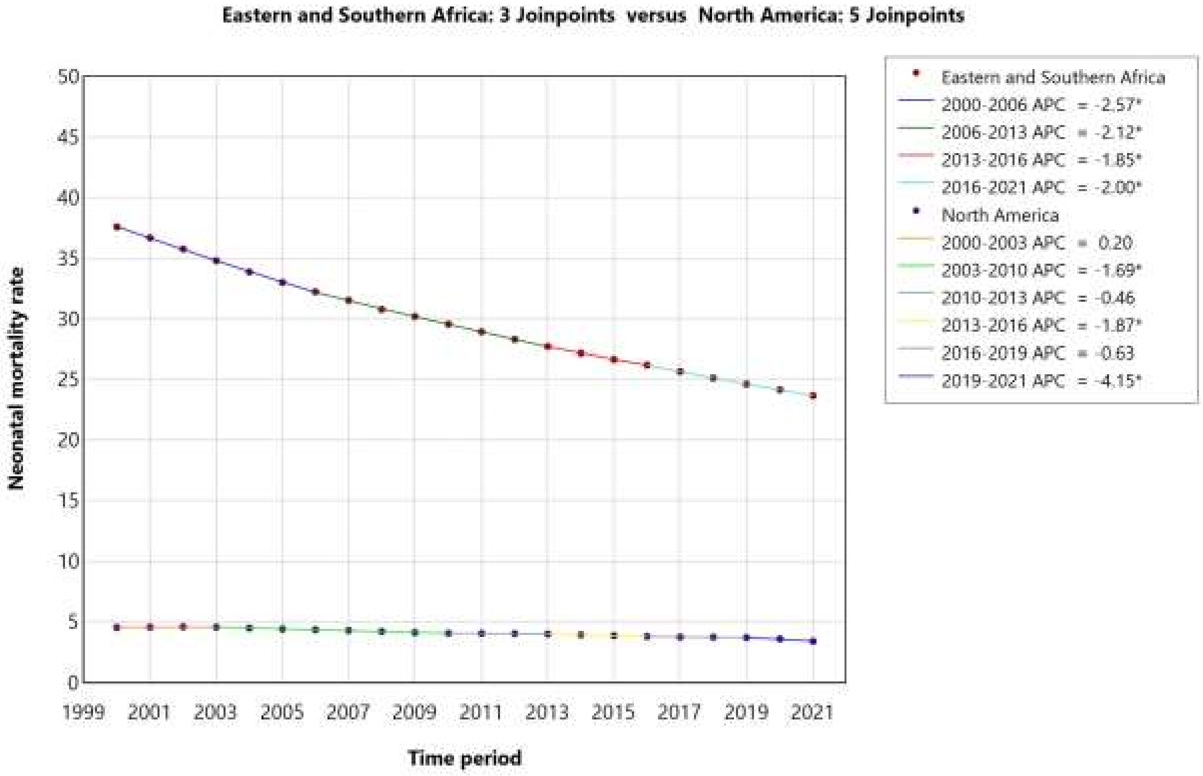

* Indicates that the Annual Percent Change (APC) is significantly different from zero at the alpha = 0.05 level.
Final Selected Model: Eastern and Southern Africa - 3 Joinpoints, North America - 5 Joinpoints. Rejected Parallelism.

Eastern and Southern Africa: 3 Joinpoints versus Middle East and North Africa: 3 Joinpoints

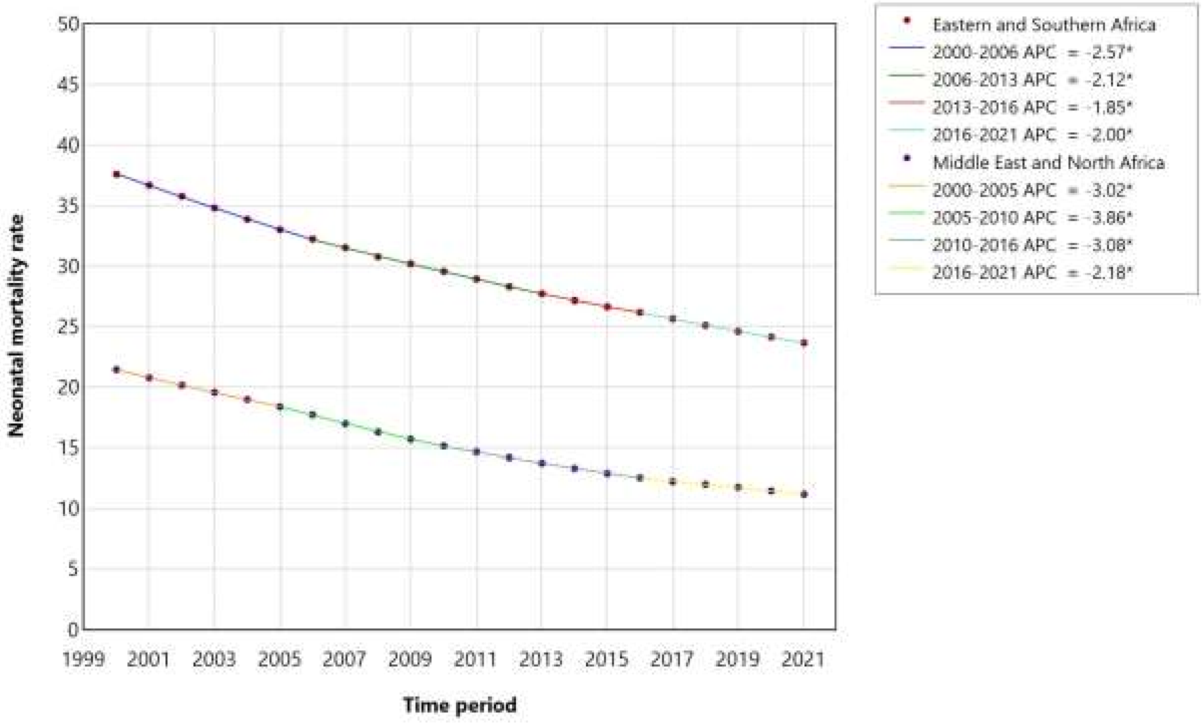

* Indicates that the Annual Percent Change (APC) is significantly different from zero at the alpha = 0.05 level.
Final Selected Model: Eastern and Southern Africa - 3 Joinpoints, Middle East and North Africa - 3 Joinpoints. Rejected Parallelism.

Eastern and Southern Africa: 3 Joinpoints versus Latin America and the Caribbean: 3 Joinpoints

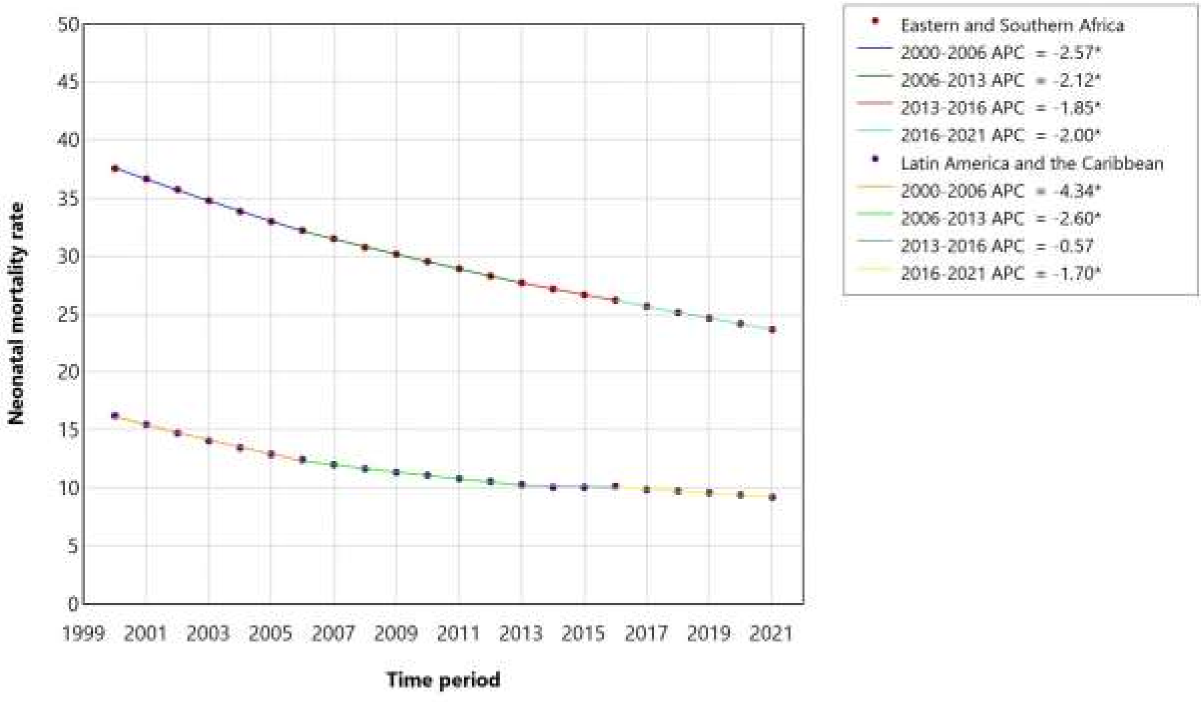

* Indicates that the Annual Percent Change (APC) is significantly different from zero at the alpha = 0.05 level.
Final Selected Model: Eastern and Southern Africa - 3 Joinpoints, Latin America and the Caribbean - 3 Joinpoints. Rejected Parallelism.

Eastern and Southern Africa: 3 Joinpoints versus Europe and Central Asia: 2 Joinpoints

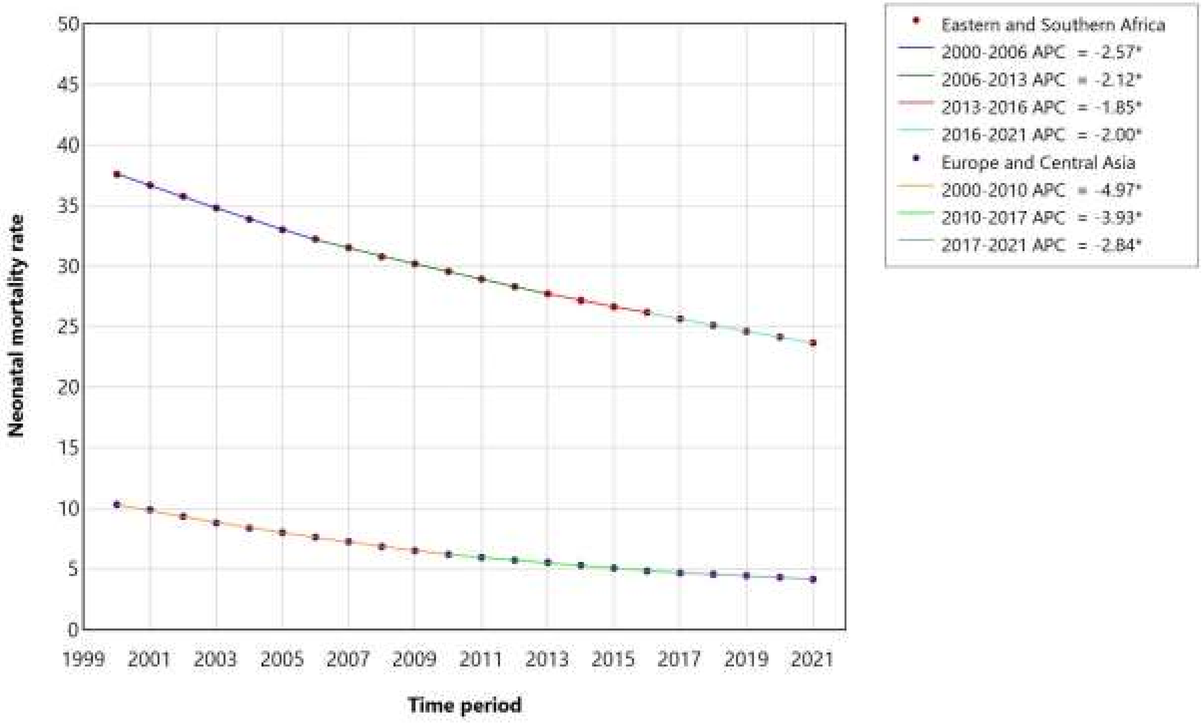

* Indicates that the Annual Percent Change (APC) is significantly different from zero at the alpha = 0.05 level.
Final Selected Model: Eastern and Southern Africa - 3 Joinpoints, Europe and Central Asia - 2 Joinpoints. Rejected Parallelism.

Eastern and Southern Africa: 3 Joinpoints versus Eastern Europe and Central Asia: 5 Joinpoints

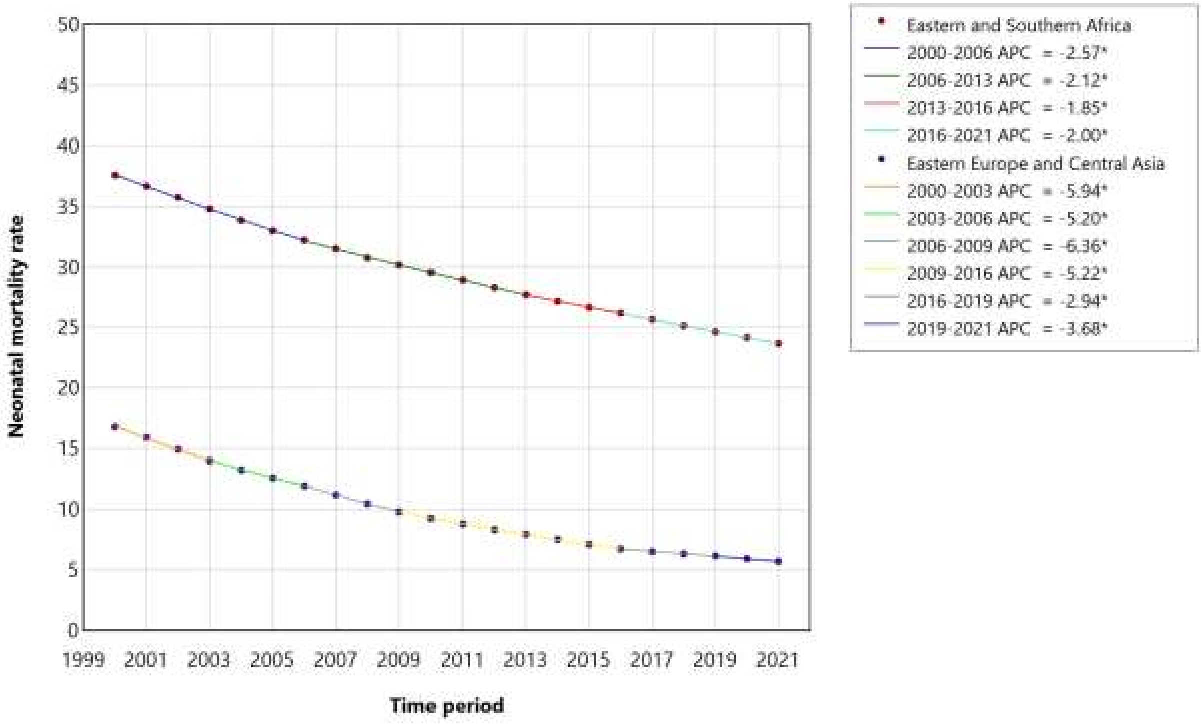

* Indicates that the Annual Percent Change (APC) is significantly different from zero at the alpha = 0.05 level.
Final Selected Model: Eastern and Southern Africa - 3 Joinpoints, Eastern Europe and Central Asia - 5 Joinpoints. Rejected Parallelism.

East Asia and Pacific: 2 Joinpoints versus Western Europe: 4 Joinpoints

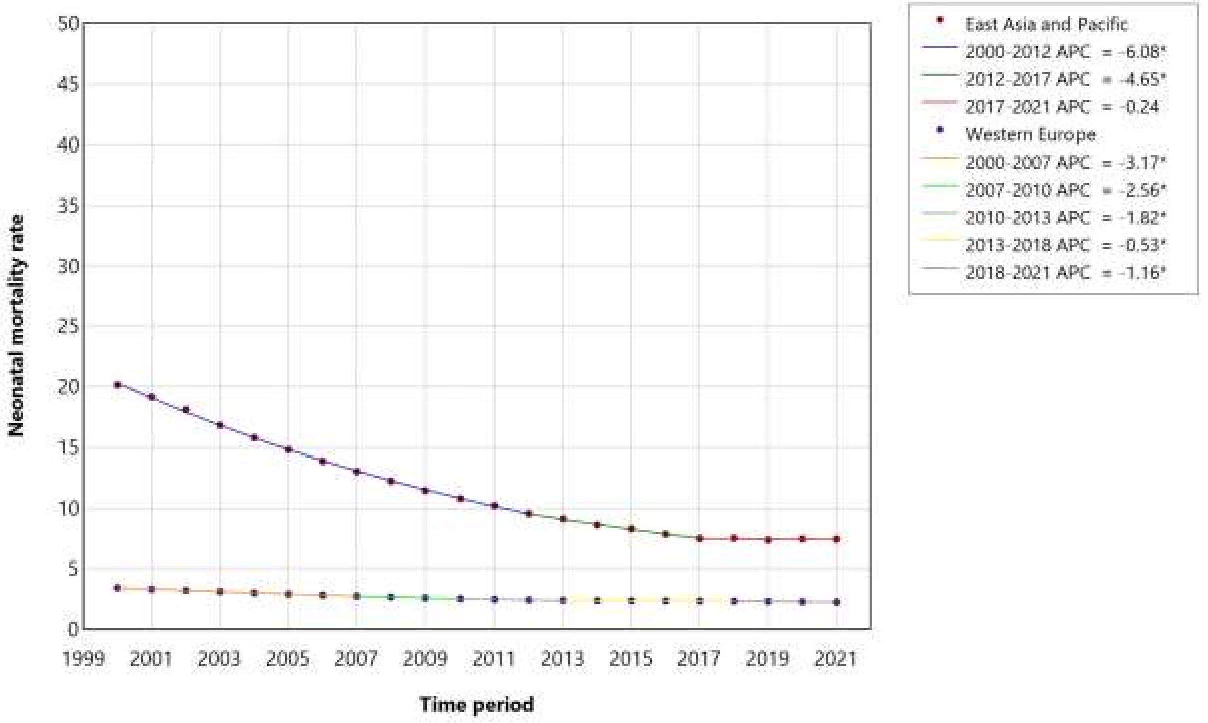

* Indicates that the Annual Percent Change (APC) is significantly different from zero at the alpha = 0.05 level.
Final Selected Model: East Asia and Pacific - 2 Joinpoints, Western Europe - 4 Joinpoints. Rejected Parallelism.

East Asia and Pacific: 2 Joinpoints versus West and Central Africa: 4 Joinpoints

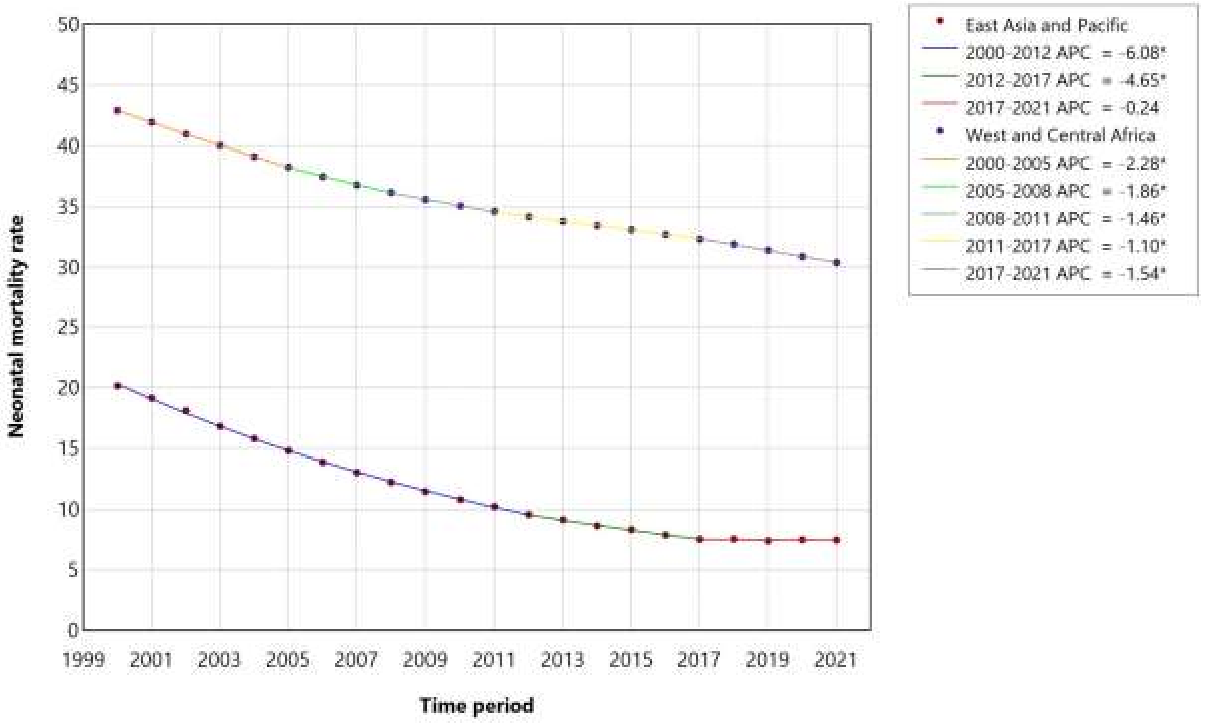

* Indicates that the Annual Percent Change (APC) is significantly different from zero at the alpha = 0.05 level.
Final Selected Model: East Asia and Pacific - 2 Joinpoints, West and Central Africa - 4 Joinpoints. Rejected Parallelism.

East Asia and Pacific: 2 Joinpoints versus Sub-Saharan Africa: 4 Joinpoints

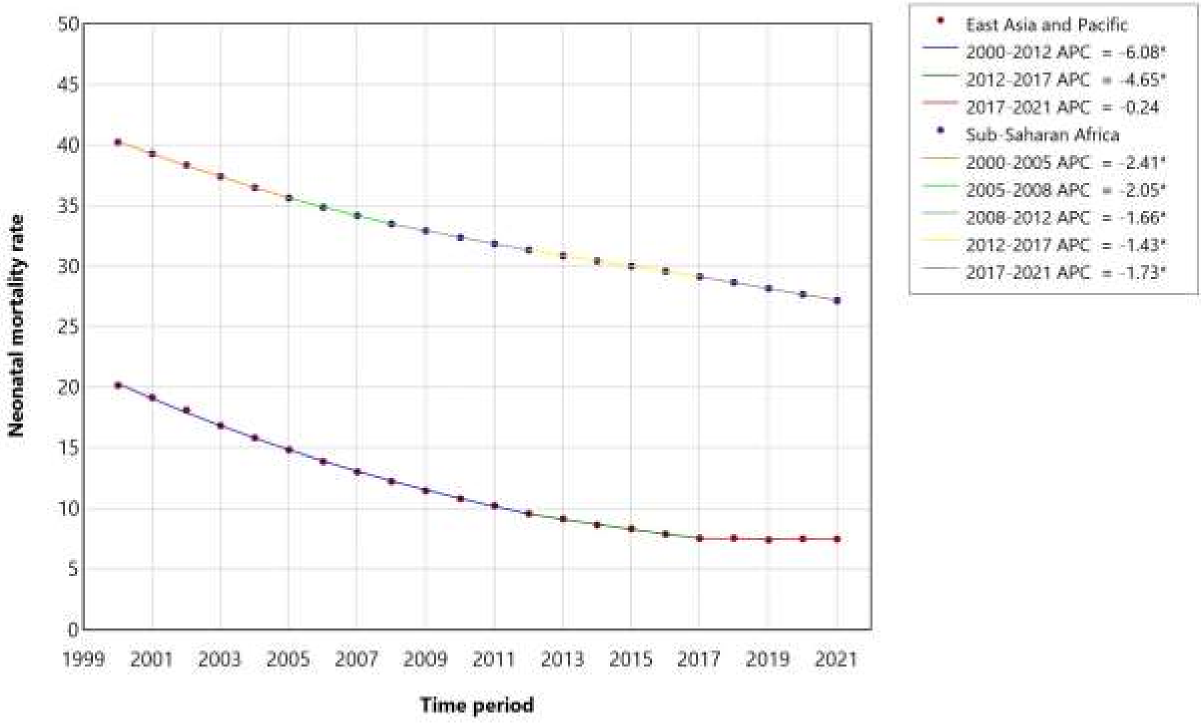

* Indicates that the Annual Percent Change (APC) is significantly different from zero at the alpha = 0.05 level.
Final Selected Model: East Asia and Pacific - 2 Joinpoints, Sub-Saharan Africa - 4 Joinpoints. Rejected Parallelism.

East Asia and Pacific: 2 Joinpoints versus South Asia: 4 Joinpoints

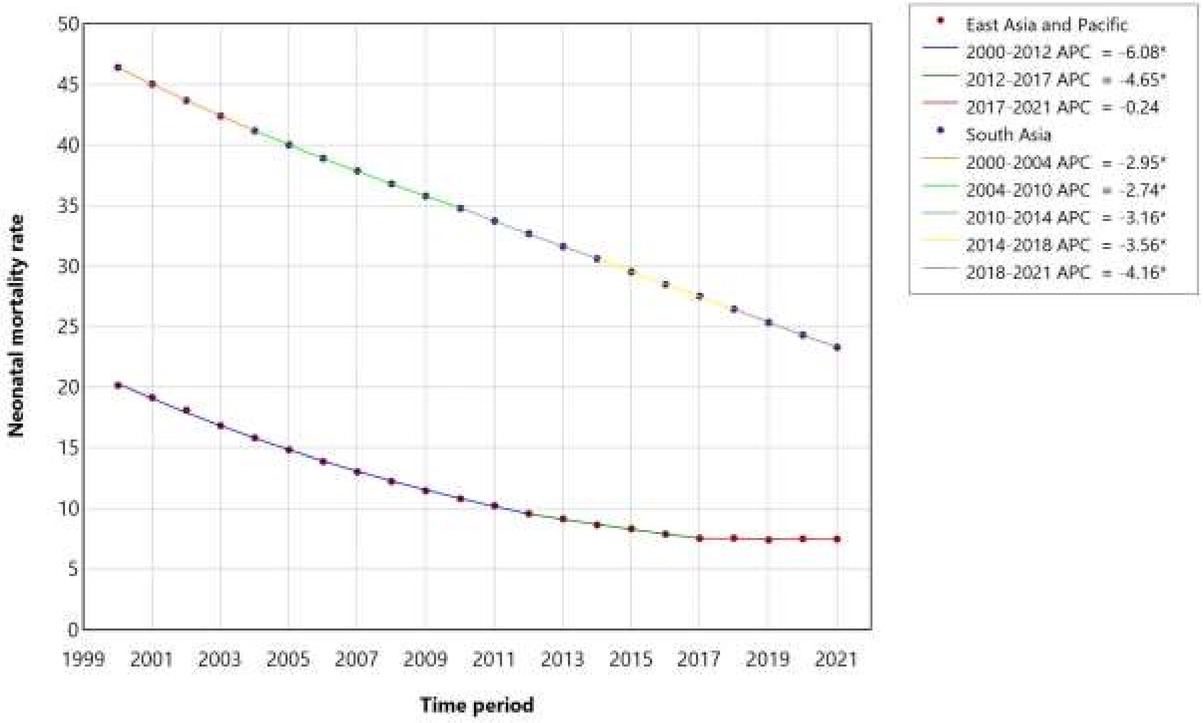

* Indicates that the Annual Percent Change (APC) is significantly different from zero at the alpha = 0.05 level.
Final Selected Model: East Asia and Pacific - 2 Joinpoints, South Asia - 4 Joinpoints. Rejected Parallelism.

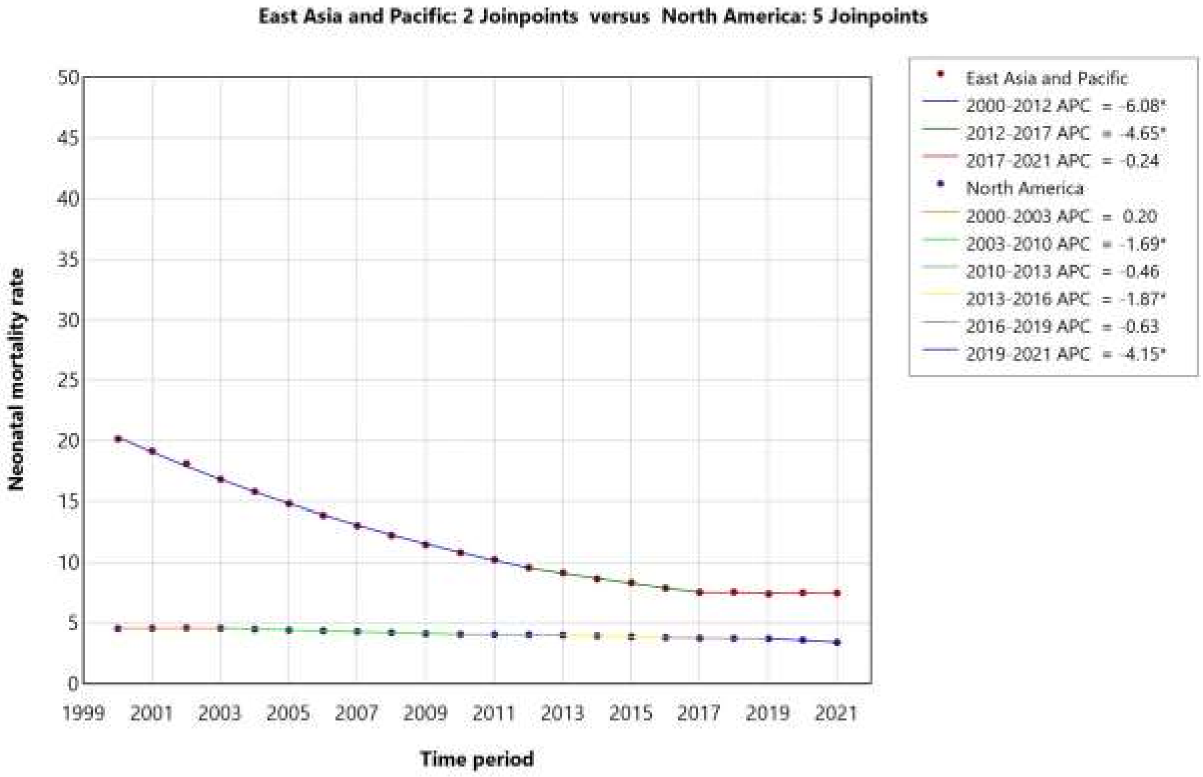

* Indicates that the Annual Percent Change (APC) is significantly different from zero at the alpha = 0.05 level.
Final Selected Model: East Asia and Pacific - 2 Joinpoints, North America - 5 Joinpoints. Rejected Parallelism.

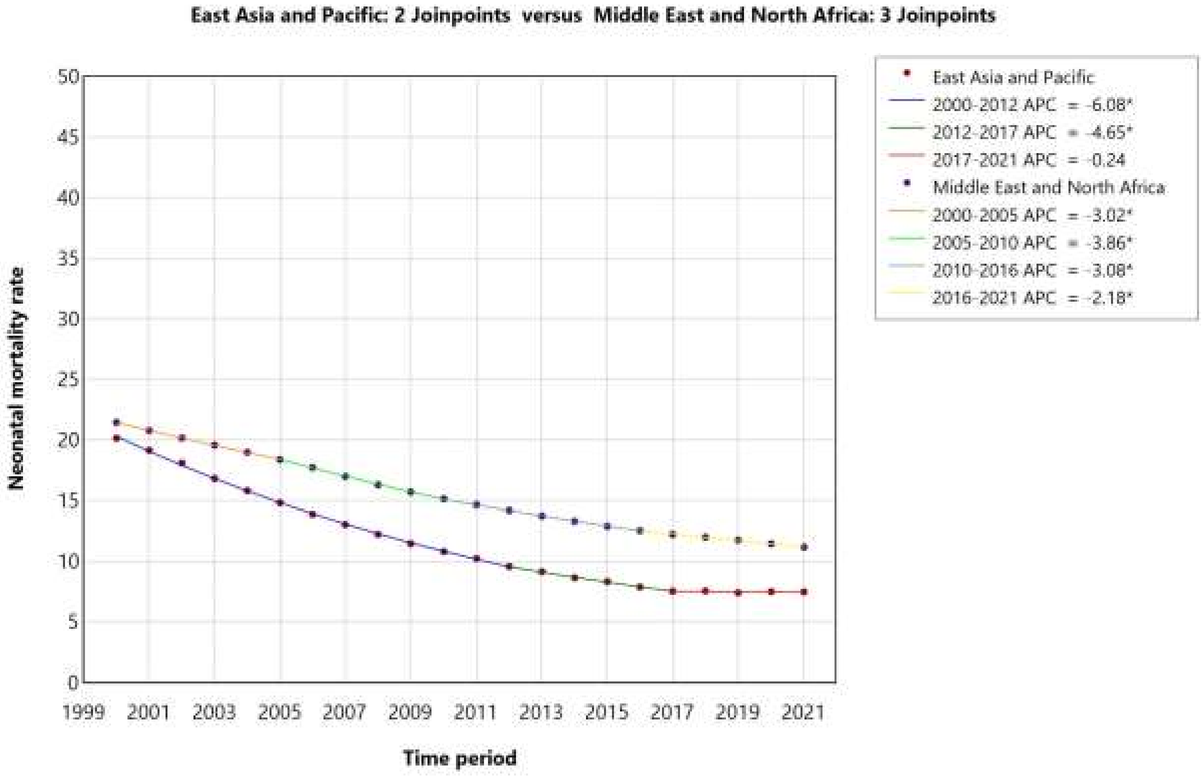

* Indicates that the Annual Percent Change (APC) is significantly different from zero at the alpha = 0.05 level.
Final Selected Model: East Asia and Pacific - 2 Joinpoints, Middle East and North Africa - 3 Joinpoints. Rejected Parallelism.

East Asia and Pacific: 2 Joinpoints versus Latin America and the Caribbean: 3 Joinpoints

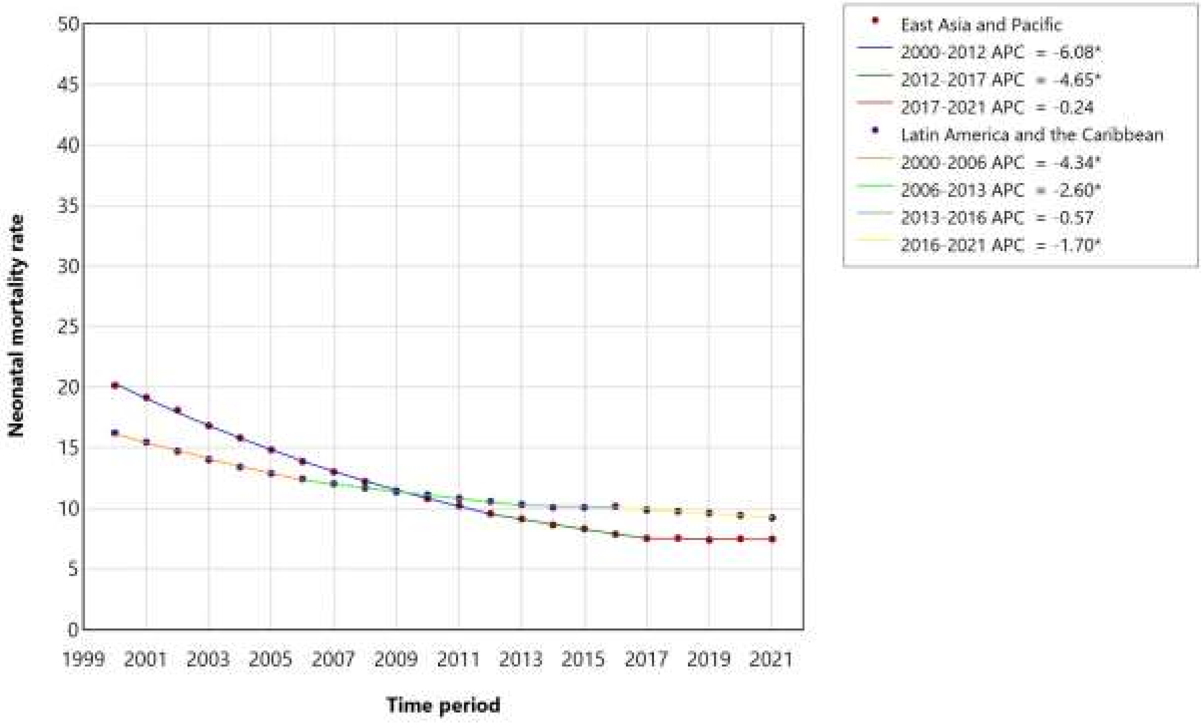

* Indicates that the Annual Percent Change (APC) is significantly different from zero at the alpha = 0.05 level.
Final Selected Model: East Asia and Pacific - 2 Joinpoints, Latin America and the Caribbean - 3 Joinpoints. Rejected Parallelism.

East Asia and Pacific: 2 Joinpoints versus Europe and Central Asia: 2 Joinpoints

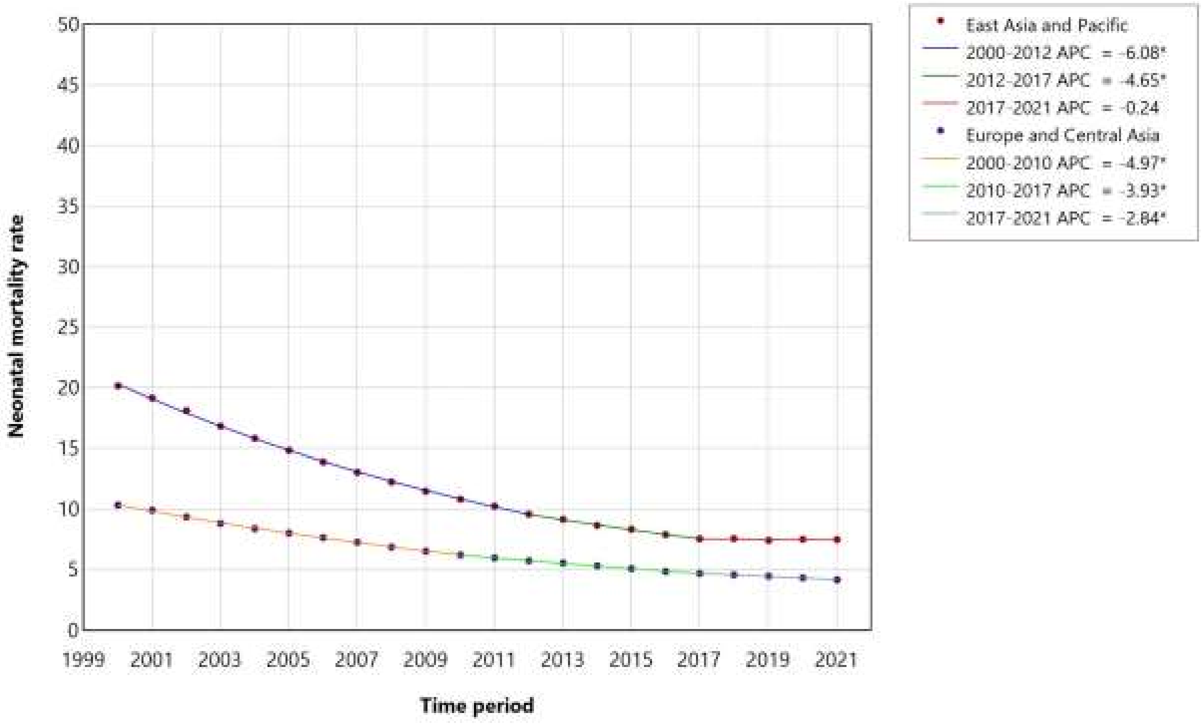

* Indicates that the Annual Percent Change (APC) is significantly different from zero at the alpha = 0.05 level.
Final Selected Model: East Asia and Pacific - 2 Joinpoints, Europe and Central Asia - 2 Joinpoints. Rejected Parallelism.

East Asia and Pacific: 2 Joinpoints versus Eastern Europe and Central Asia: 5 Joinpoints

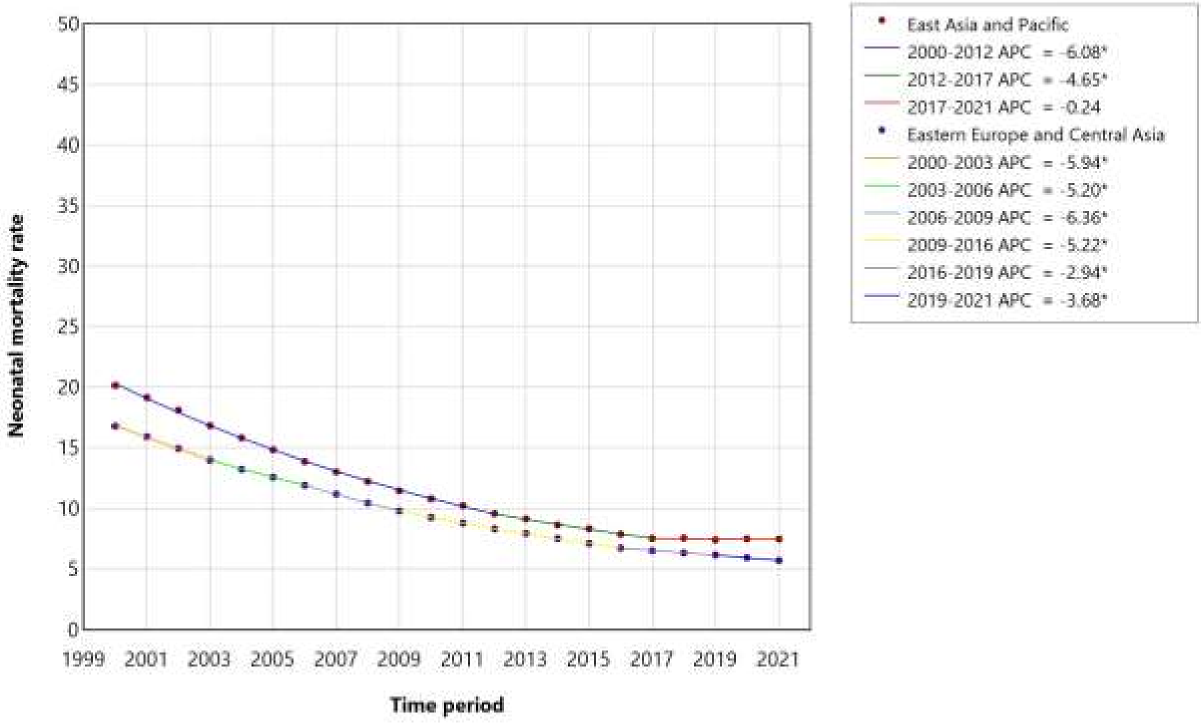

* Indicates that the Annual Percent Change (APC) is significantly different from zero at the alpha = 0.05 level.
Final Selected Model: East Asia and Pacific - 2 Joinpoints, Eastern Europe and Central Asia - 5 Joinpoints. Rejected Parallelism.

East Asia and Pacific: 2 Joinpoints versus Eastern and Southern Africa: 3 Joinpoints

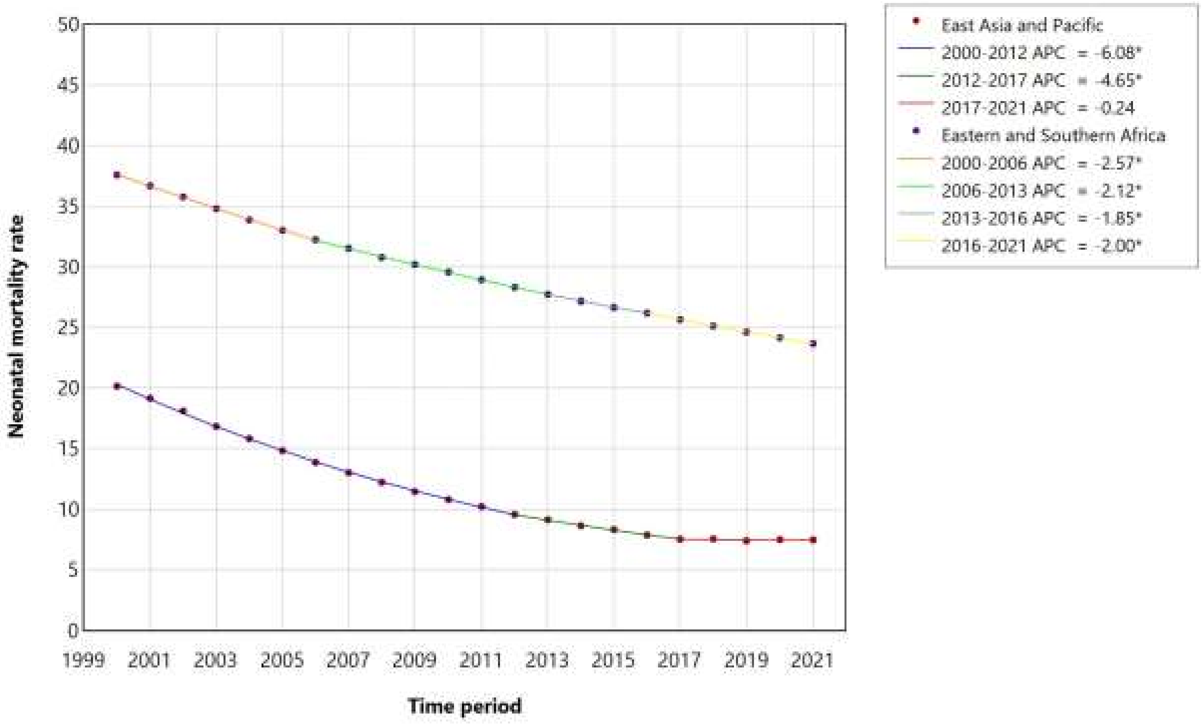

* Indicates that the Annual Percent Change (APC) is significantly different from zero at the alpha = 0.05 level.
Final Selected Model: East Asia and Pacific - 2 Joinpoints, Eastern and Southern Africa - 3 Joinpoints. Rejected Parallelism.

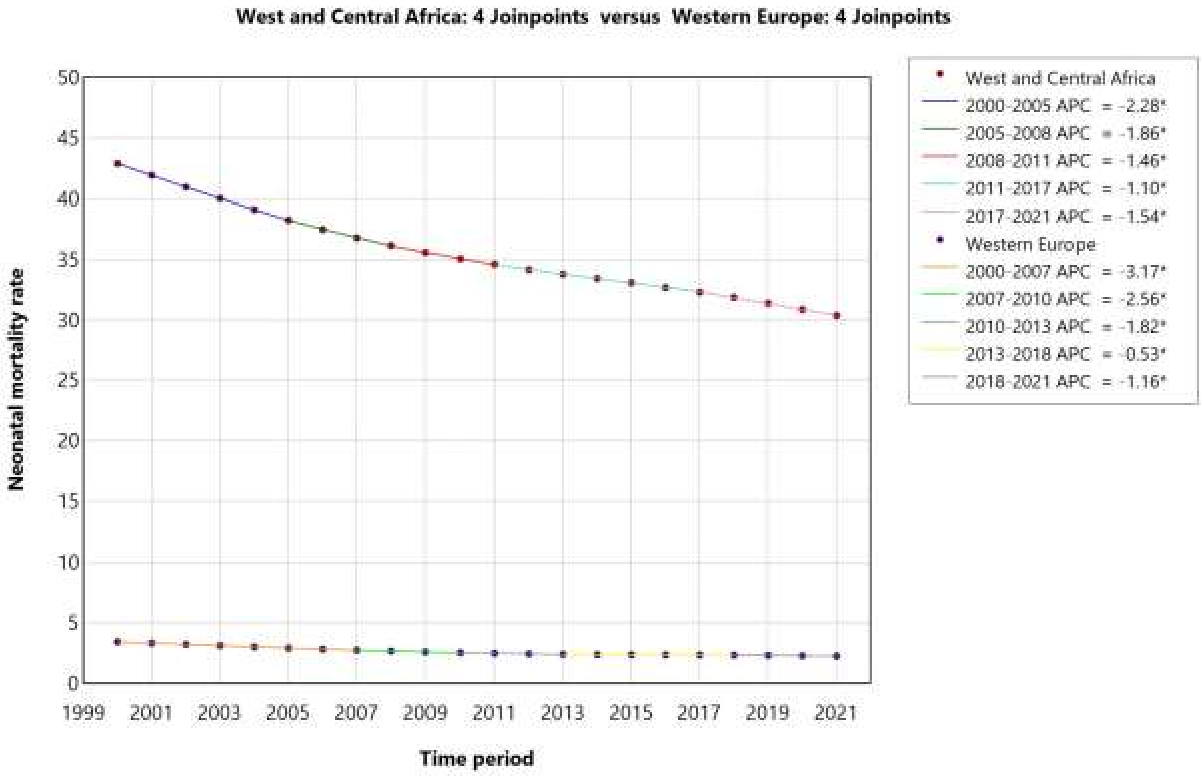

* Indicates that the Annual Percent Change (APC) is significantly different from zero at the alpha = 0.05 level.
Final Selected Model: West and Central Africa - 4 Joinpoints, Western Europe - 4 Joinpoints. Rejected Parallelism.

Figure S3. Pairwise comparisons of temporal trend in infant mortality rates across UNICEF reporting regions using joinpoint regression

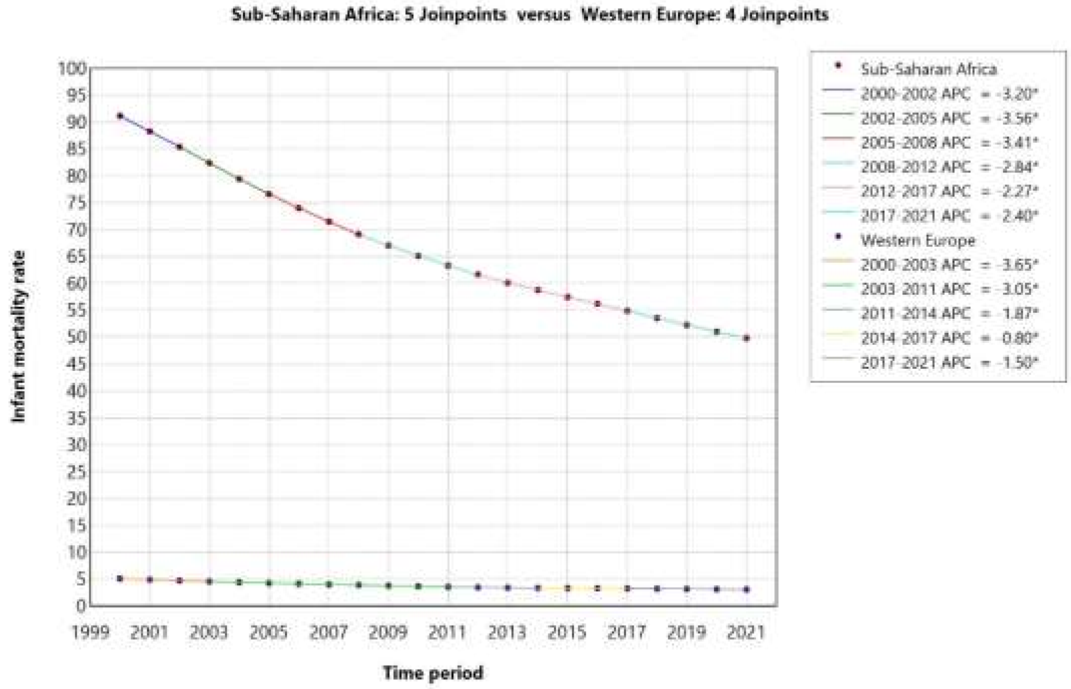

* Indicates that the Annual Percent Change (APC) is significantly different from zero at the alpha = 0.05 level.
Final Selected Model: Sub-Saharan Africa - 5 Joinpoints, Western Europe - 4 Joinpoints. Rejected Parallelism.

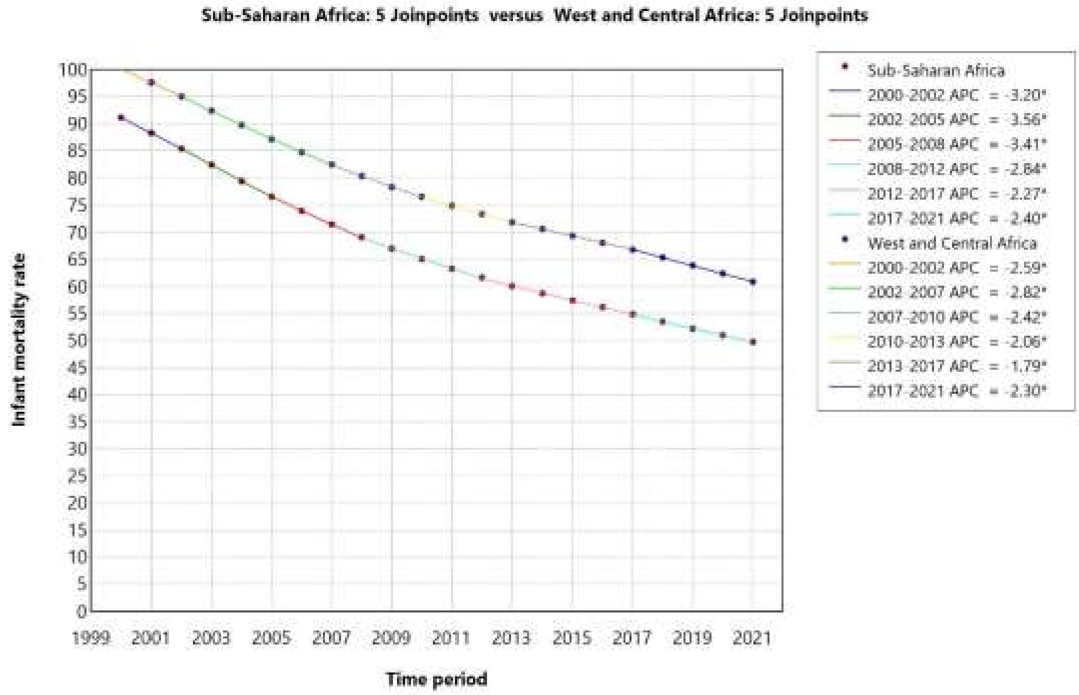

* Indicates that the Annual Percent Change (APC) is significantly different from zero at the alpha = 0.05 level.
Final Selected Model: Sub-Saharan Africa - 5 Joinpoints, West and Central Africa - 5 Joinpoints. Rejected Parallelism.

South Asia: 4 Joinpoints versus Western Europe: 4 Joinpoints

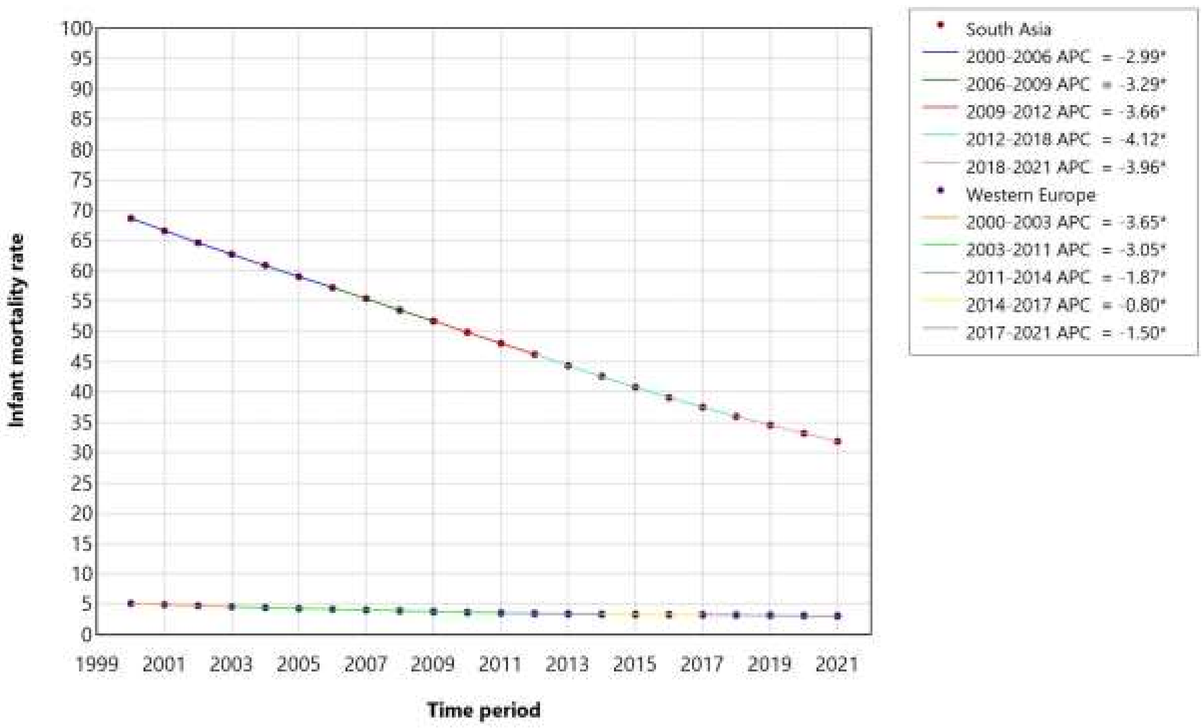

* Indicates that the Annual Percent Change (APC) is significantly different from zero at the alpha = 0.05 level.
Final Selected Model: South Asia - 4 Joinpoints, Western Europe - 4 Joinpoints. Rejected Parallelism.

South Asia: 4 Joinpoints versus West and Central Africa: 5 Joinpoints

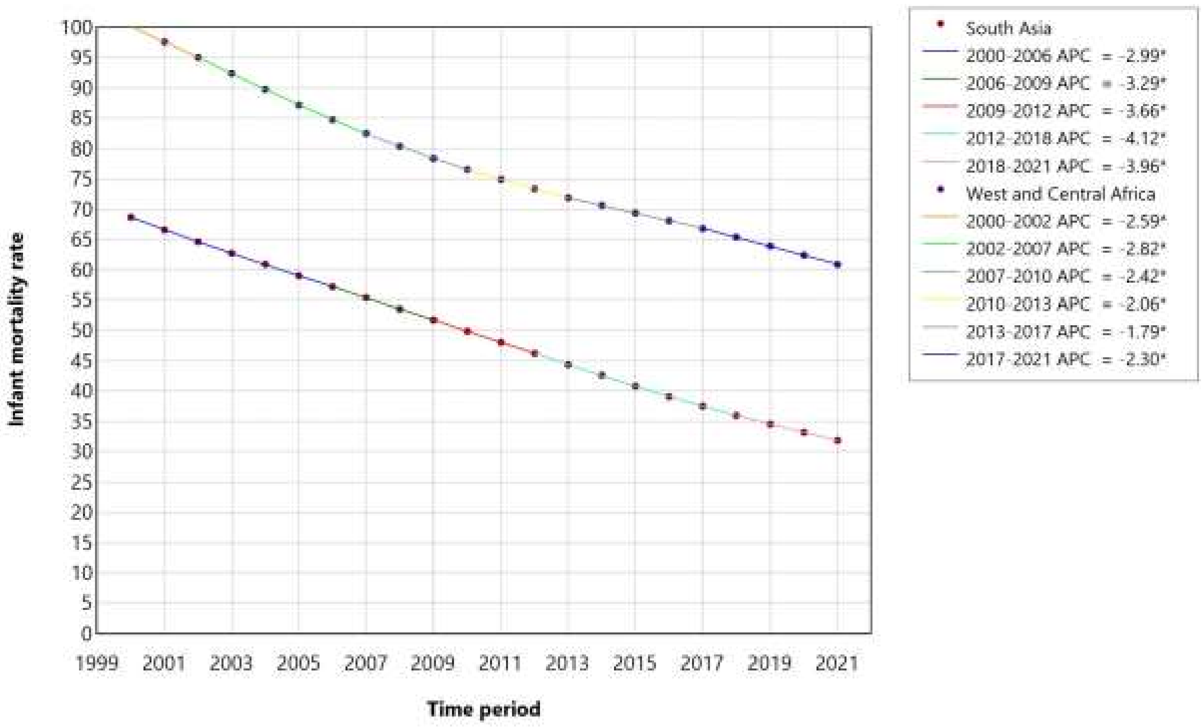

* Indicates that the Annual Percent Change (APC) is significantly different from zero at the alpha = 0.05 level.
Final Selected Model: South Asia - 4 Joinpoints, West and Central Africa - 5 Joinpoints. Rejected Parallelism.

South Asia: 4 Joinpoints versus Sub-Saharan Africa: 5 Joinpoints

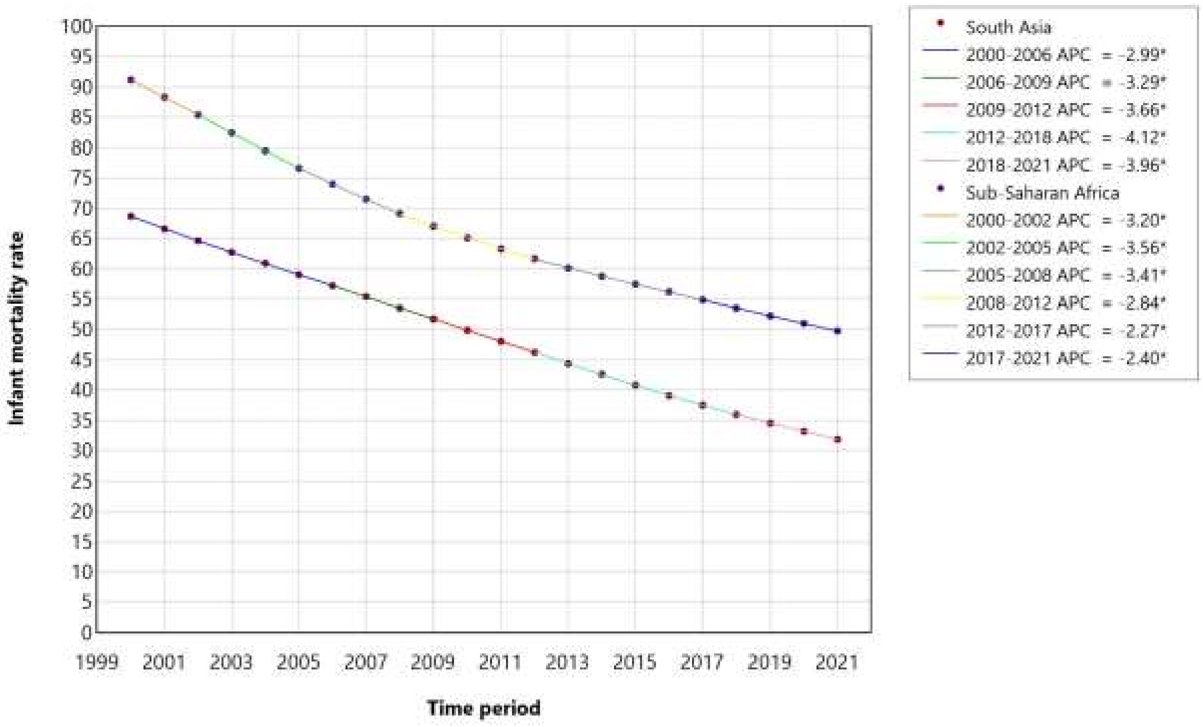

* Indicates that the Annual Percent Change (APC) is significantly different from zero at the alpha = 0.05 level.
Final Selected Model: South Asia - 4 Joinpoints, Sub-Saharan Africa - 5 Joinpoints. Rejected Parallelism.

North America: 3 Joinpoints versus Western Europe: 4 Joinpoints

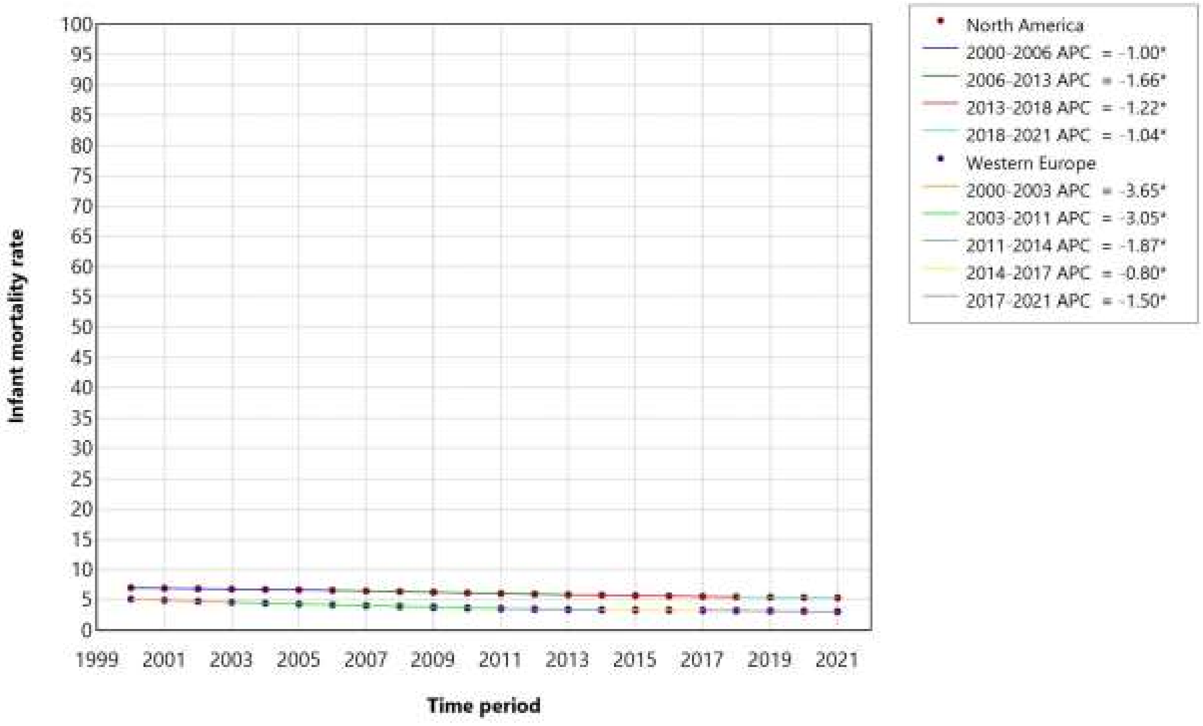

* Indicates that the Annual Percent Change (APC) is significantly different from zero at the alpha = 0.05 level.
Final Selected Model: North America - 3 Joinpoints, Western Europe - 4 Joinpoints. Rejected Parallelism.

North America: 3 Joinpoints versus West and Central Africa: 5 Joinpoints

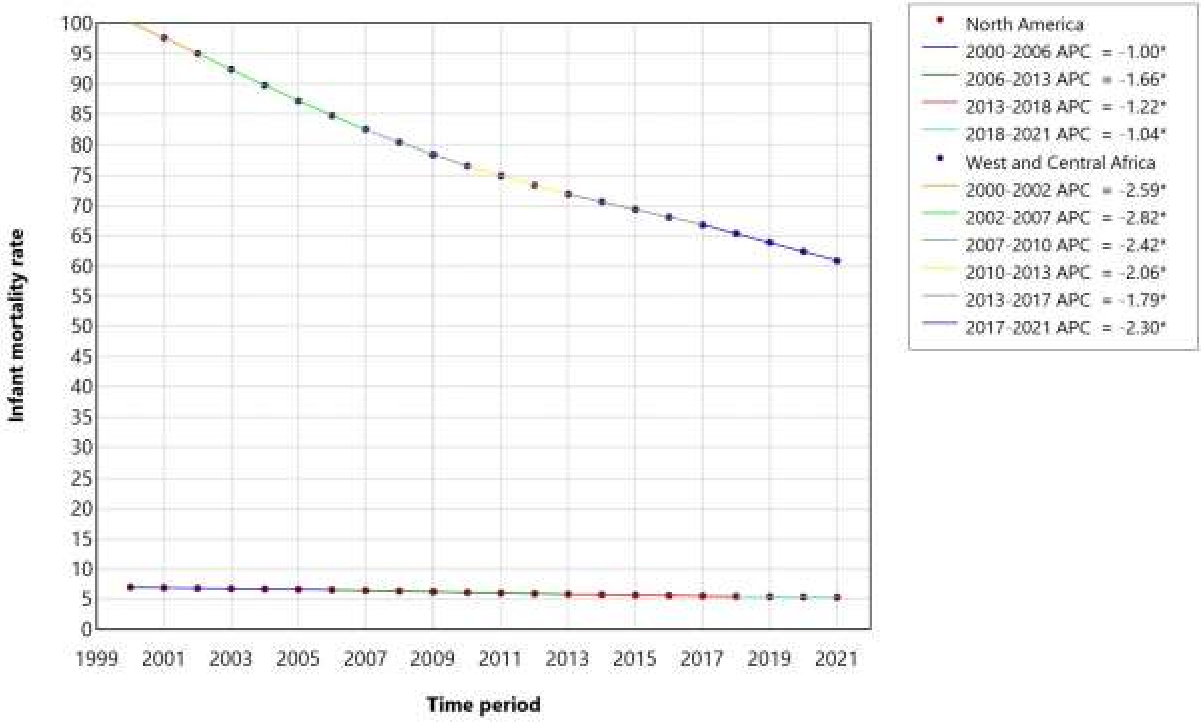

* Indicates that the Annual Percent Change (APC) is significantly different from zero at the alpha = 0.05 level.
Final Selected Model: North America - 3 Joinpoints, West and Central Africa - 5 Joinpoints. Rejected Parallelism.

North America: 3 Joinpoints versus Sub-Saharan Africa: 5 Joinpoints

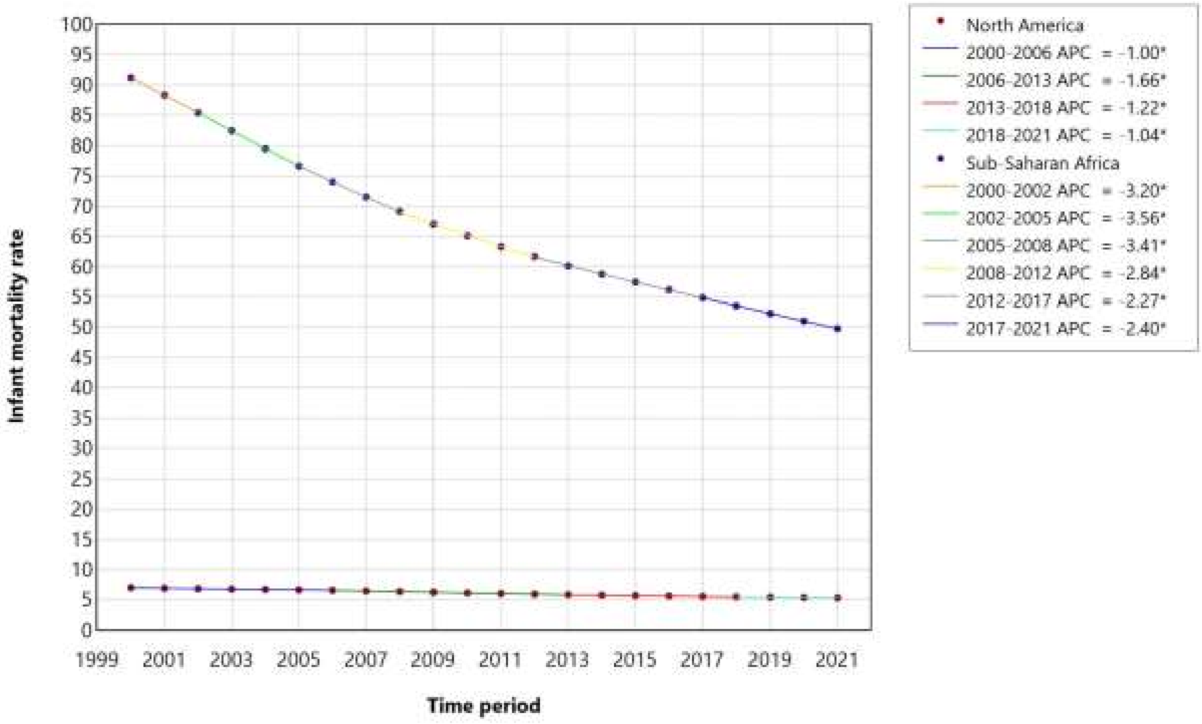

* Indicates that the Annual Percent Change (APC) is significantly different from zero at the alpha = 0.05 level.
Final Selected Model: North America - 3 Joinpoints, Sub-Saharan Africa - 5 Joinpoints. Rejected Parallelism.

North America: 3 Joinpoints versus South Asia: 4 Joinpoints

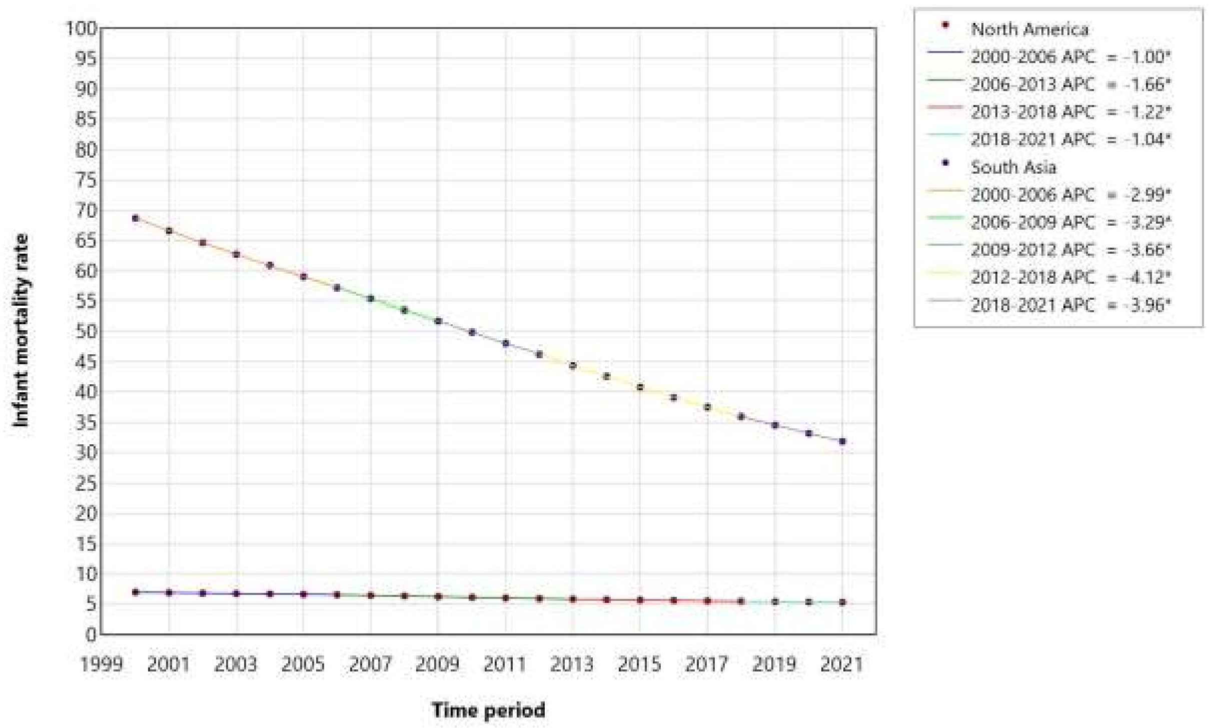

* Indicates that the Annual Percent Change (APC) is significantly different from zero at the alpha = 0.05 level.
Final Selected Model: North America - 3 Joinpoints, South Asia - 4 Joinpoints. Rejected Parallelism.

Middle East and North Africa: 3 Joinpoints versus Western Europe: 4 Joinpoints

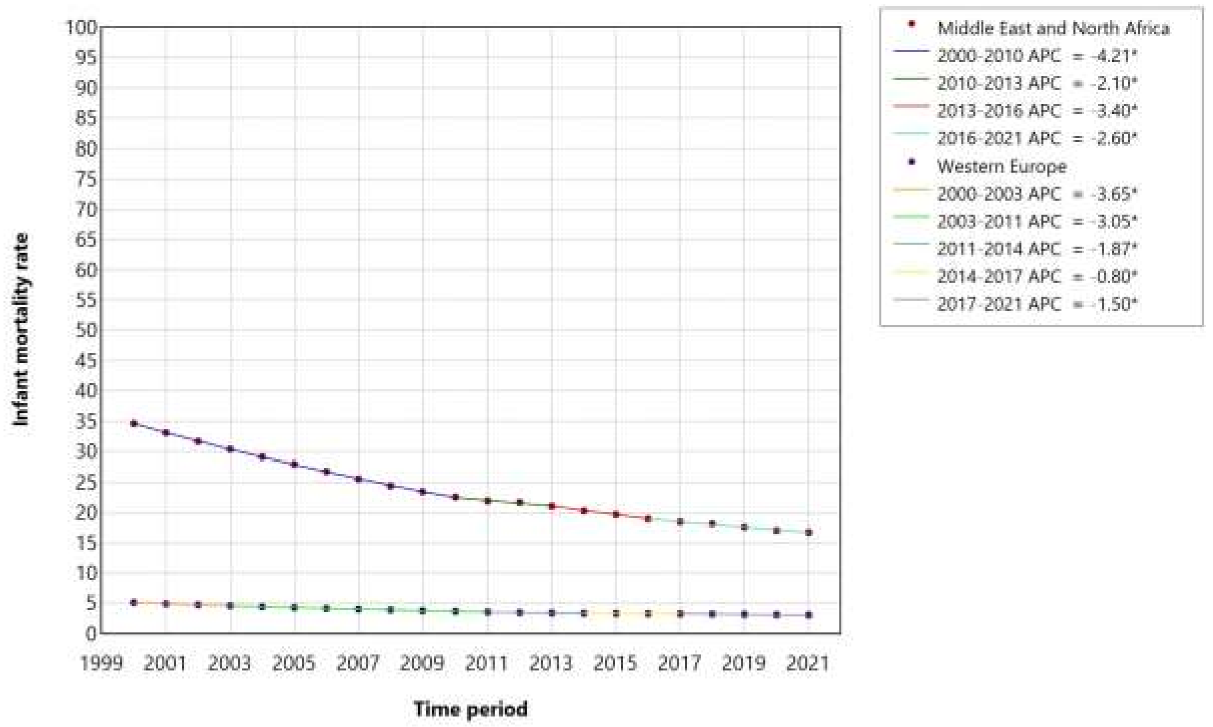

* Indicates that the Annual Percent Change (APC) is significantly different from zero at the alpha = 0.05 level.
Final Selected Model: Middle East and North Africa - 3 Joinpoints, Western Europe - 4 Joinpoints. Rejected Parallelism.

Middle East and North Africa: 3 Joinpoints versus West and Central Africa: 5 Joinpoints

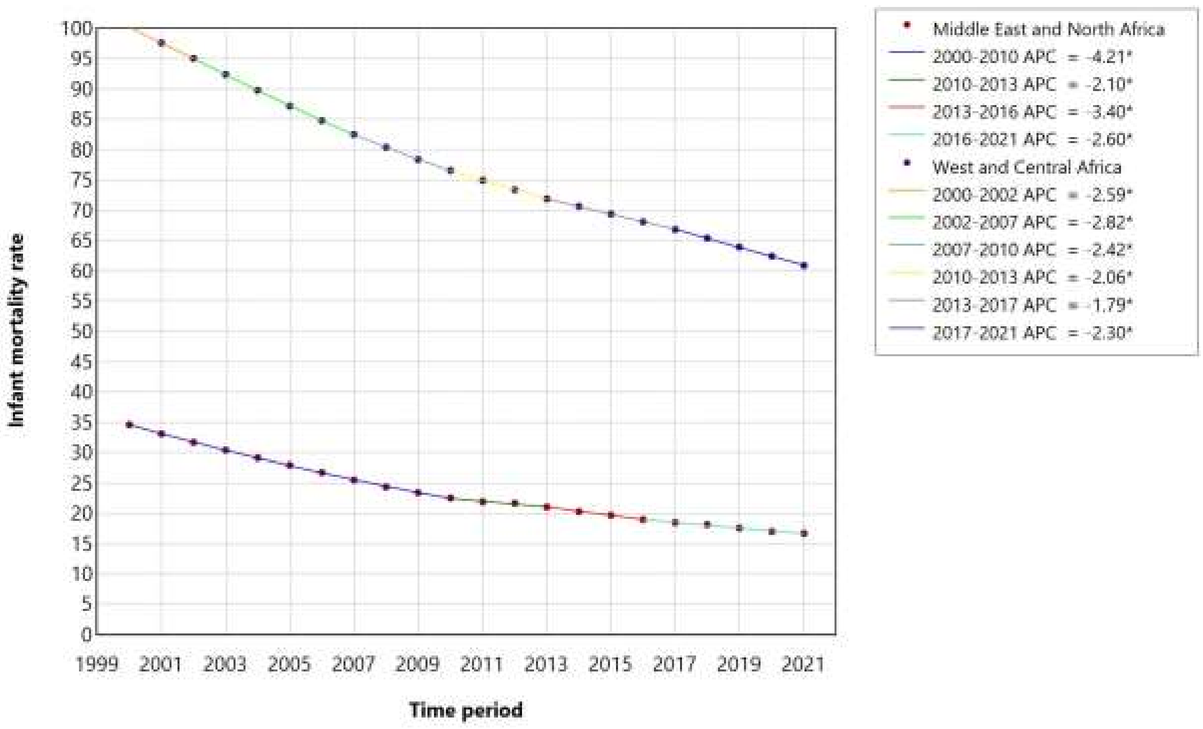

* Indicates that the Annual Percent Change (APC) is significantly different from zero at the alpha = 0.05 level.
Final Selected Model: Middle East and North Africa - 3 Joinpoints, West and Central Africa - 5 Joinpoints. Rejected Parallelism.

Middle East and North Africa: 3 Joinpoints versus Sub-Saharan Africa: 5 Joinpoints

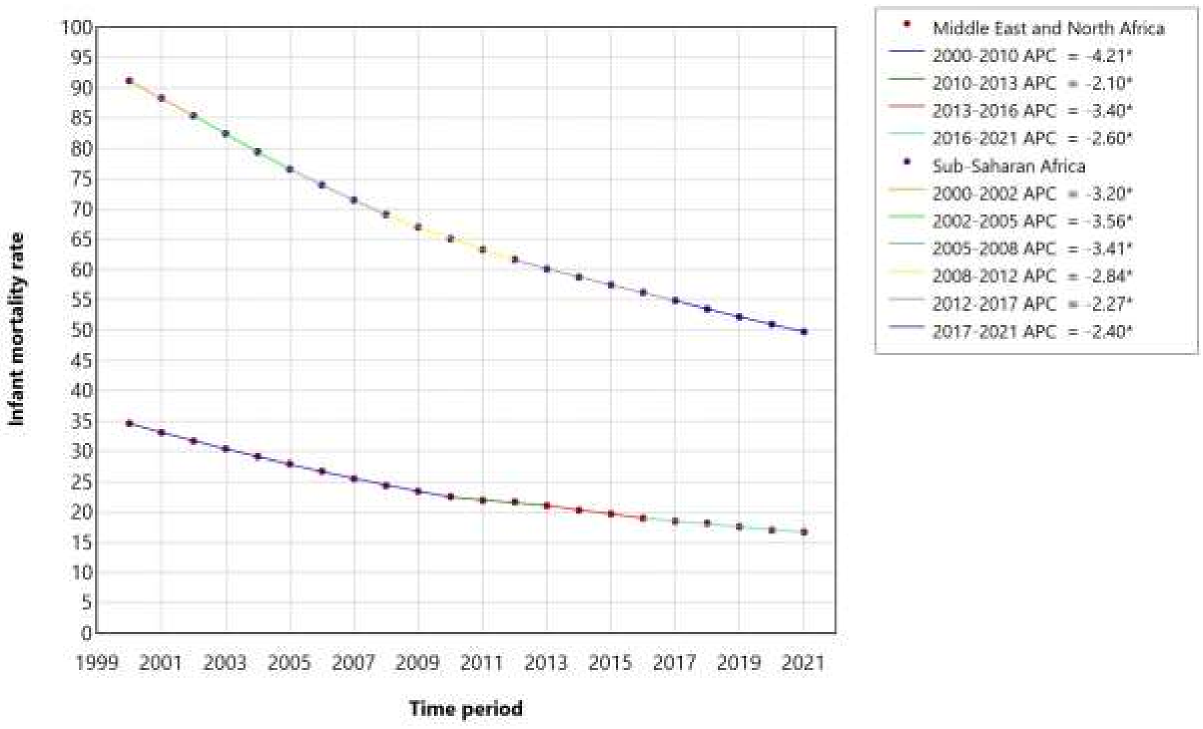

* Indicates that the Annual Percent Change (APC) is significantly different from zero at the alpha = 0.05 level.
Final Selected Model: Middle East and North Africa - 3 Joinpoints, Sub-Saharan Africa - 5 Joinpoints. Rejected Parallelism.

Middle East and North Africa: 3 Joinpoints versus South Asia: 4 Joinpoints

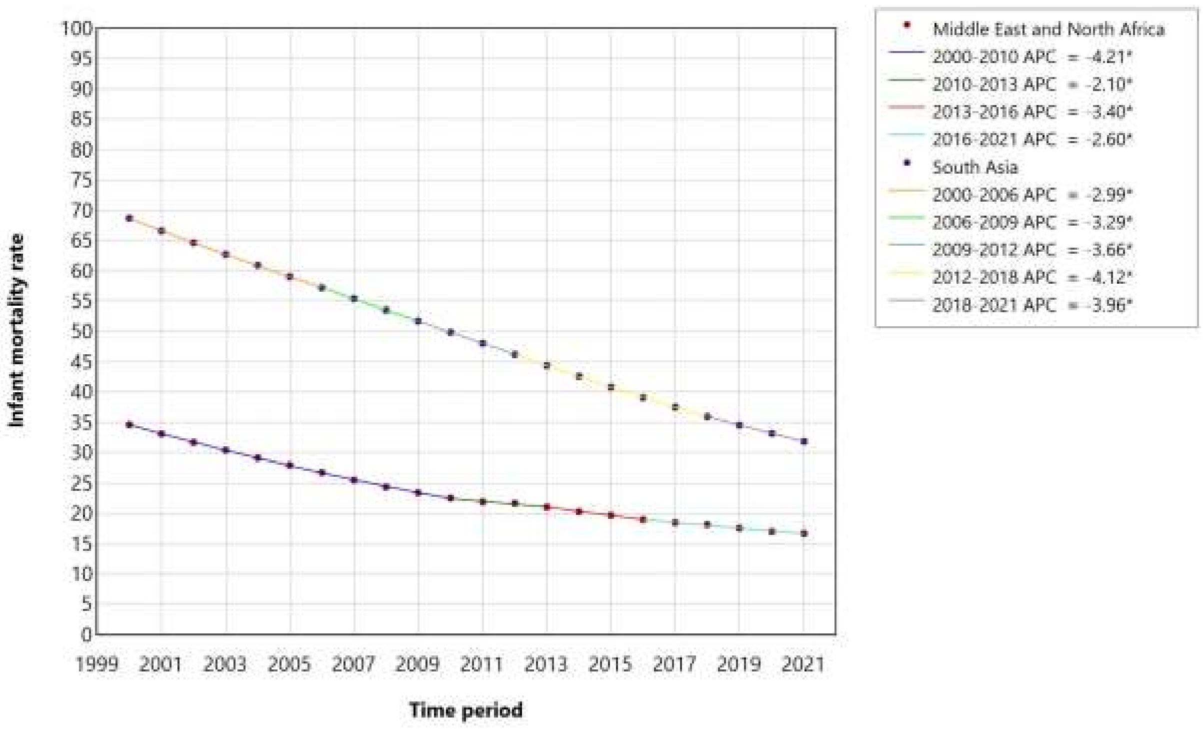

* Indicates that the Annual Percent Change (APC) is significantly different from zero at the alpha = 0.05 level.
Final Selected Model: Middle East and North Africa - 3 Joinpoints, South Asia - 4 Joinpoints. Rejected Parallelism.

Middle East and North Africa: 3 Joinpoints versus North America: 3 Joinpoints

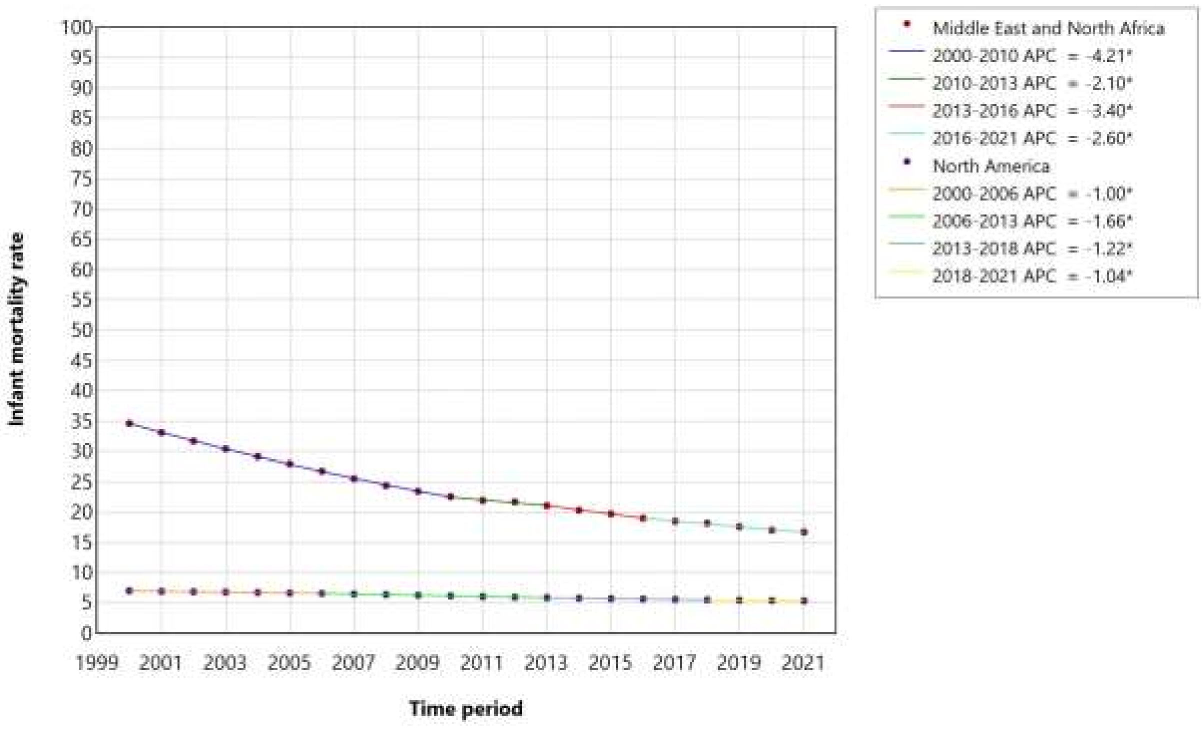

* Indicates that the Annual Percent Change (APC) is significantly different from zero at the alpha = 0.05 level.
Final Selected Model: Middle East and North Africa - 3 Joinpoints, North America - 3 Joinpoints. Rejected Parallelism.

Latin America and the Caribbean: 2 Joinpoints versus Western Europe: 4 Joinpoints

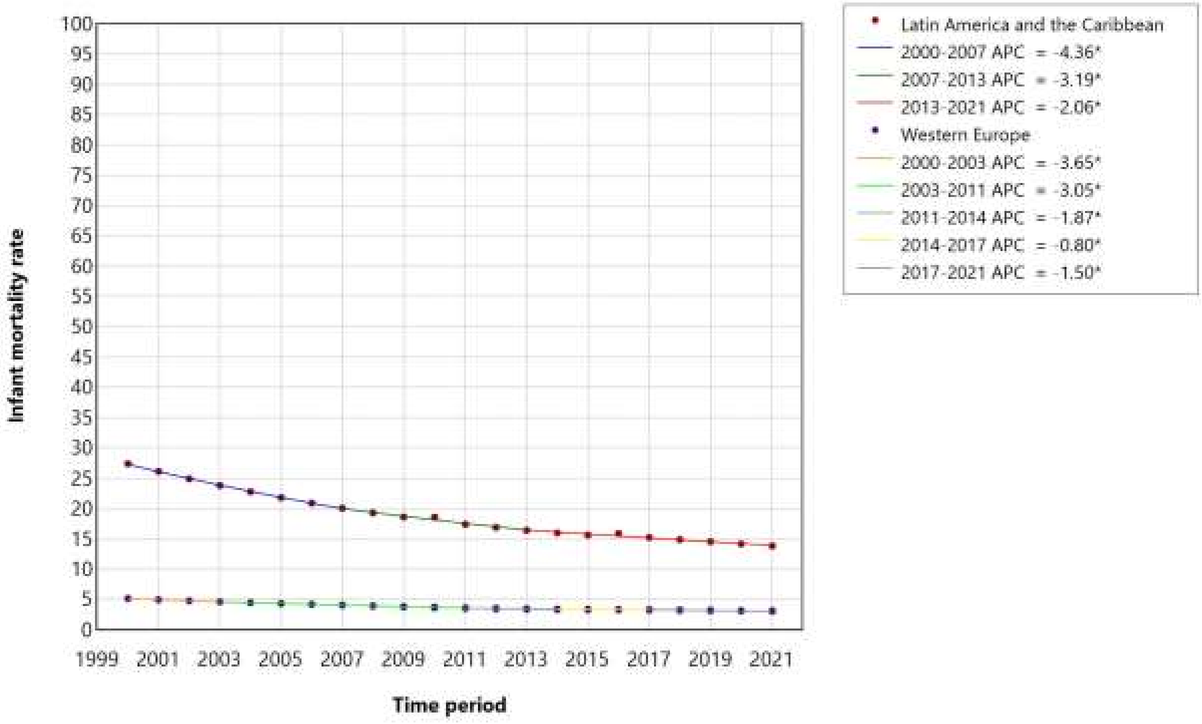

* Indicates that the Annual Percent Change (APC) is significantly different from zero at the alpha = 0.05 level.
Final Selected Model: Latin America and the Caribbean - 2 Joinpoints, Western Europe - 4 Joinpoints. Rejected Parallelism.

Latin America and the Caribbean: 2 Joinpoints versus West and Central Africa: 5 Joinpoints

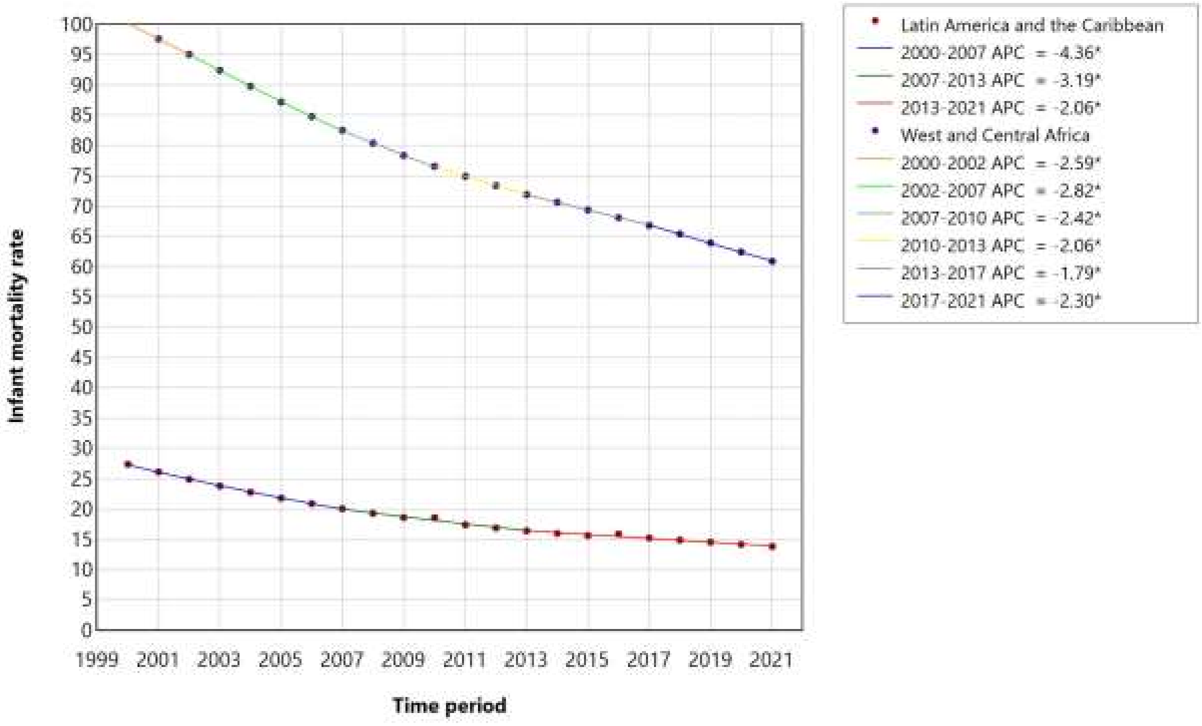

* Indicates that the Annual Percent Change (APC) is significantly different from zero at the alpha = 0.05 level.
Final Selected Model: Latin America and the Caribbean - 2 Joinpoints, West and Central Africa - 5 Joinpoints. Rejected Parallelism.

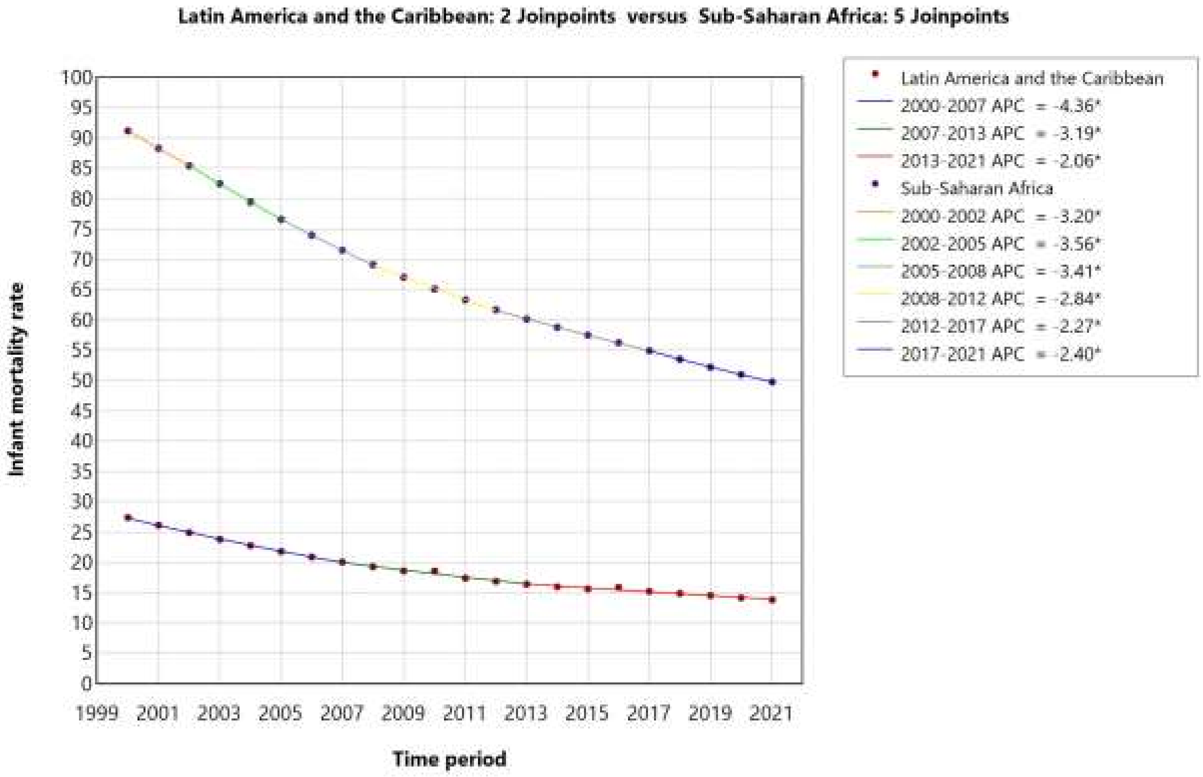

* Indicates that the Annual Percent Change (APC) is significantly different from zero at the alpha = 0.05 level.
Final Selected Model: Latin America and the Caribbean - 2 Joinpoints, Sub-Saharan Africa - 5 Joinpoints. Rejected Parallelism.

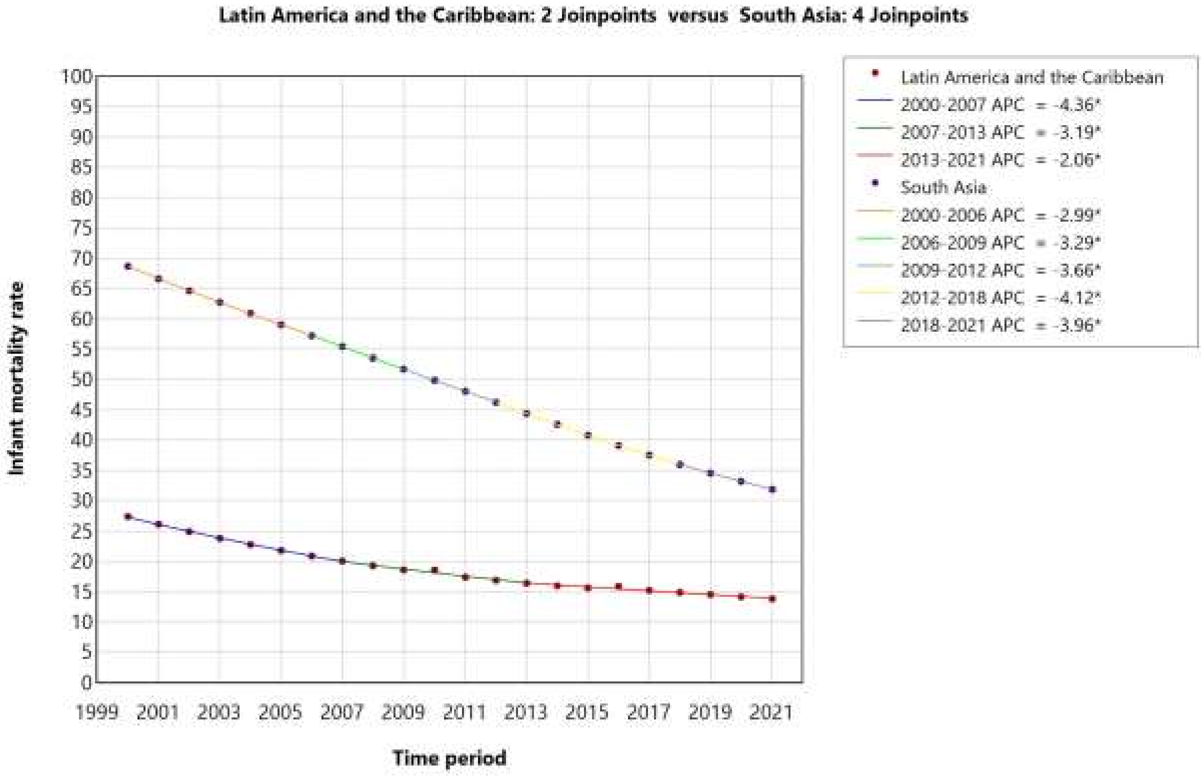

* Indicates that the Annual Percent Change (APC) is significantly different from zero at the alpha = 0.05 level.
Final Selected Model: Latin America and the Caribbean - 2 Joinpoints, South Asia - 4 Joinpoints. Rejected Parallelism.

Latin America and the Caribbean: 2 Joinpoints versus North America: 3 Joinpoints

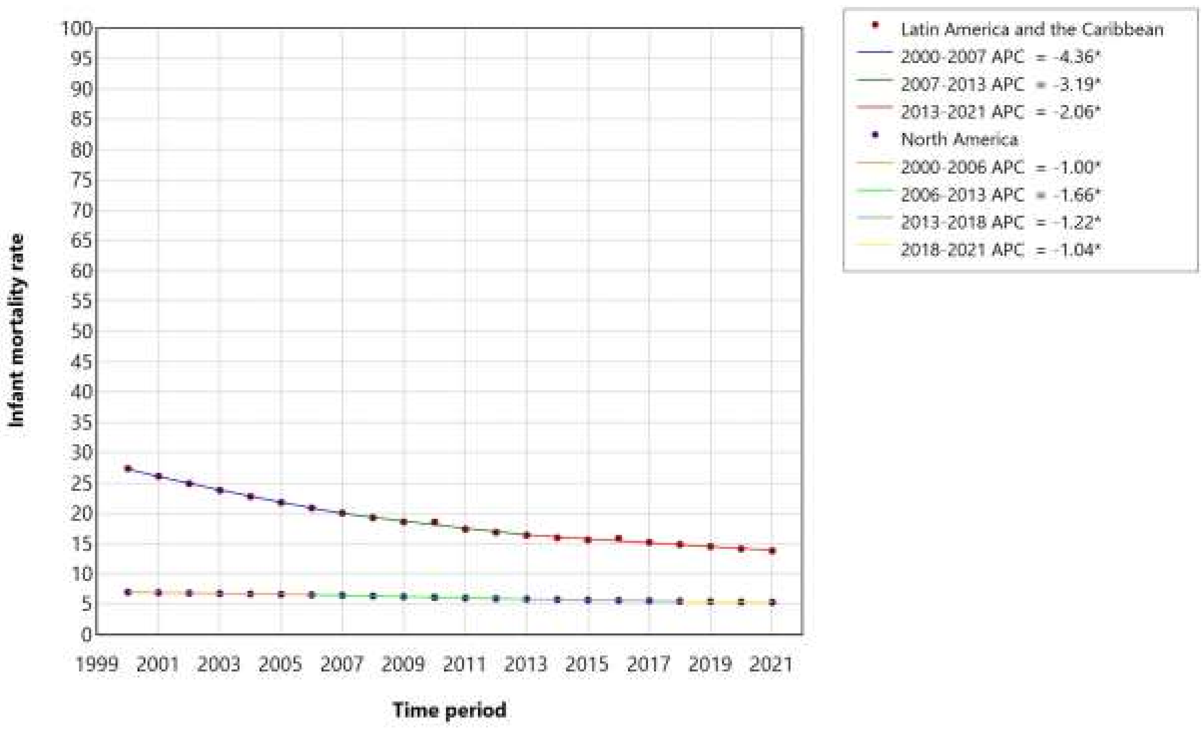

* Indicates that the Annual Percent Change (APC) is significantly different from zero at the alpha = 0.05 level.
Final Selected Model: Latin America and the Caribbean - 2 Joinpoints, North America - 3 Joinpoints. Rejected Parallelism.

Latin America and the Caribbean: 2 Joinpoints versus Middle East and North Africa: 3 Joinpoints

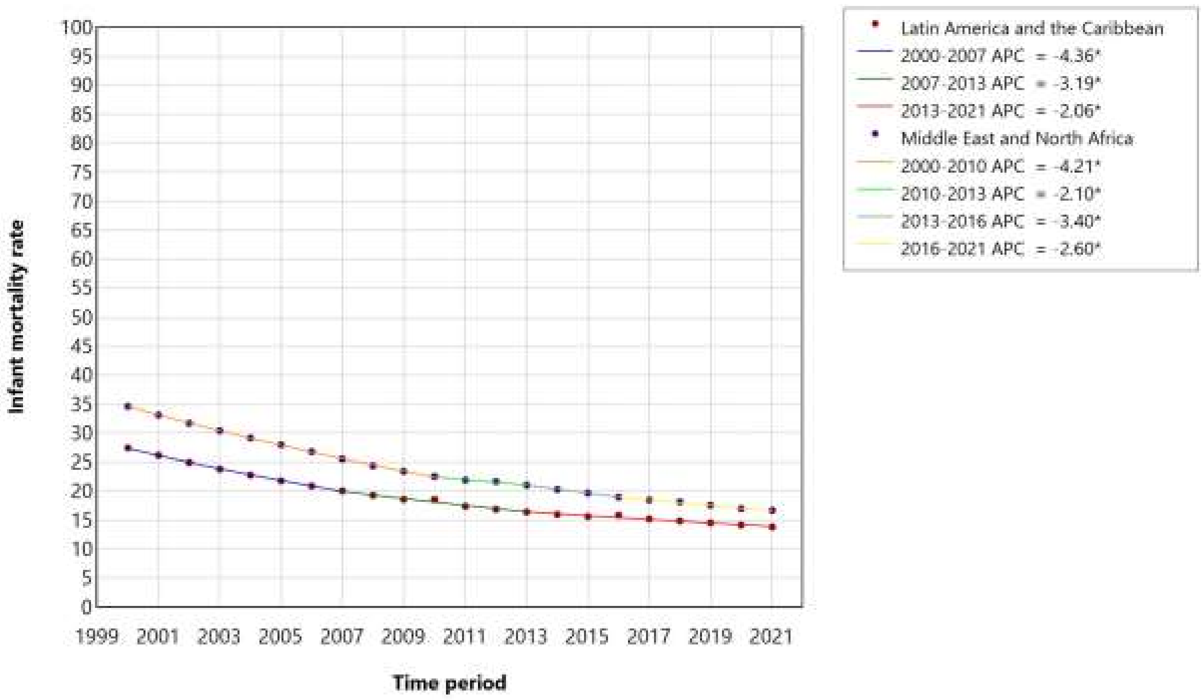

* Indicates that the Annual Percent Change (APC) is significantly different from zero at the alpha = 0.05 level.
Final Selected Model: Latin America and the Caribbean - 2 Joinpoints, Middle East and North Africa - 3 Joinpoints. Rejected Parallelism.

Europe and Central Asia: 3 Joinpoints versus Western Europe: 4 Joinpoints

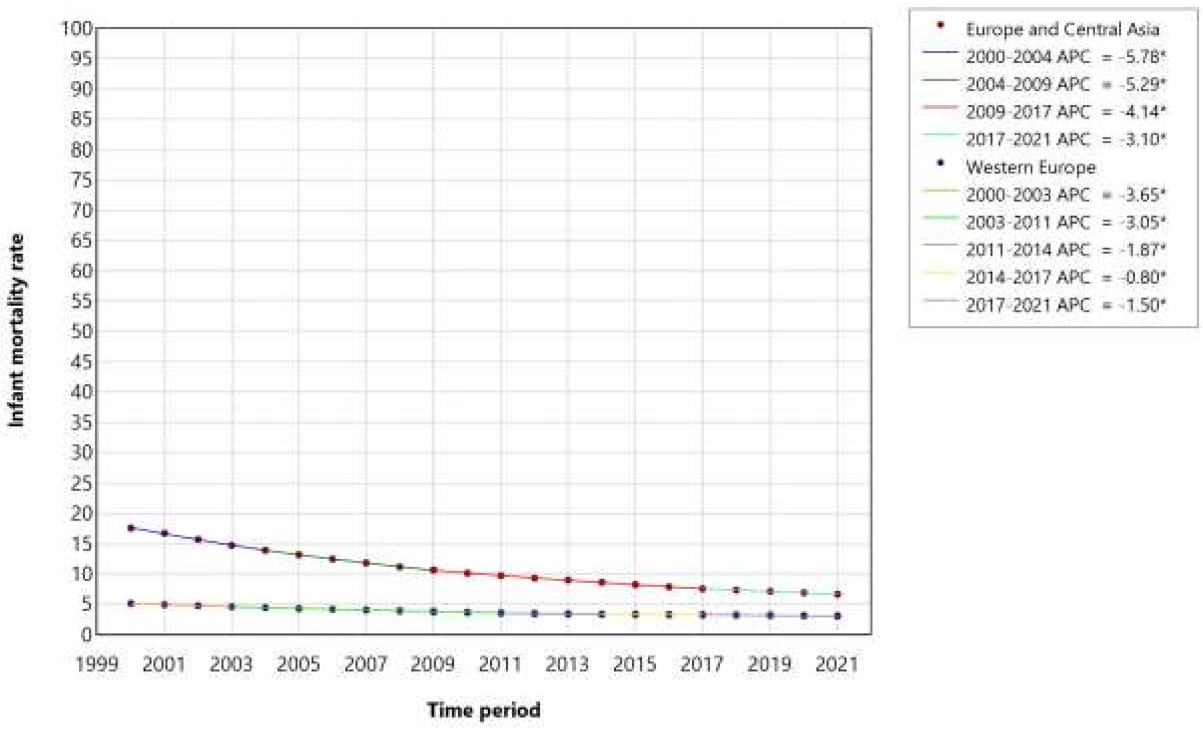

* Indicates that the Annual Percent Change (APC) is significantly different from zero at the alpha = 0.05 level.
Final Selected Model: Europe and Central Asia - 3 Joinpoints, Western Europe - 4 Joinpoints. Rejected Parallelism.

Europe and Central Asia: 3 Joinpoints versus West and Central Africa: 5 Joinpoints

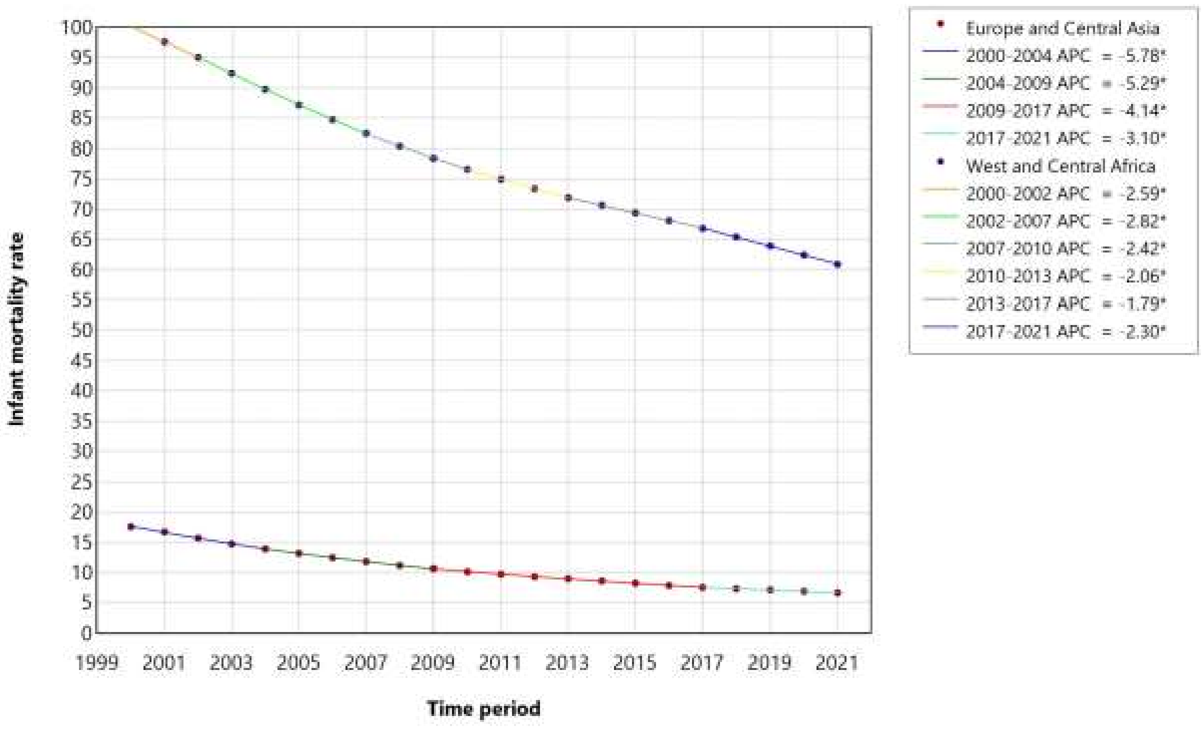

* Indicates that the Annual Percent Change (APC) is significantly different from zero at the alpha = 0.05 level.
Final Selected Model: Europe and Central Asia - 3 Joinpoints, West and Central Africa - 5 Joinpoints. Rejected Parallelism.

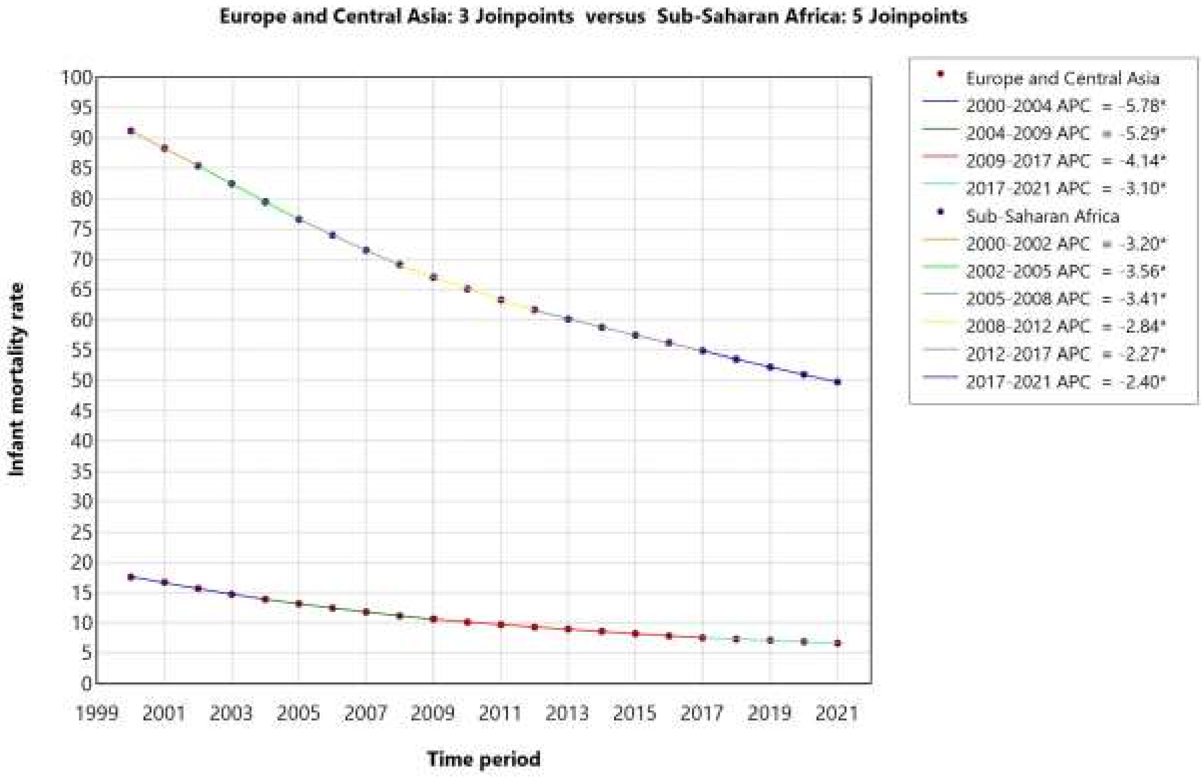

* Indicates that the Annual Percent Change (APC) is significantly different from zero at the alpha = 0.05 level.
Final Selected Model: Europe and Central Asia - 3 Joinpoints, Sub-Saharan Africa - 5 Joinpoints. Rejected Parallelism.

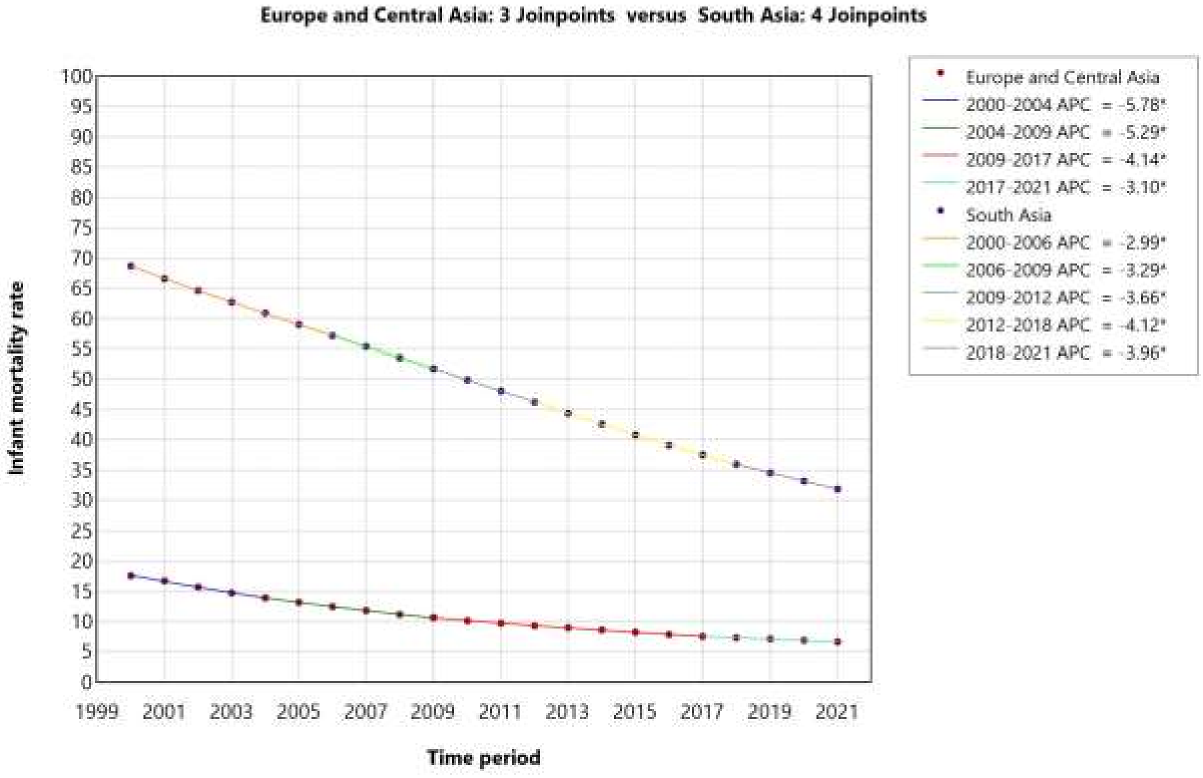

* Indicates that the Annual Percent Change (APC) is significantly different from zero at the alpha = 0.05 level.
Final Selected Model: Europe and Central Asia - 3 Joinpoints, South Asia - 4 Joinpoints. Rejected Parallelism.

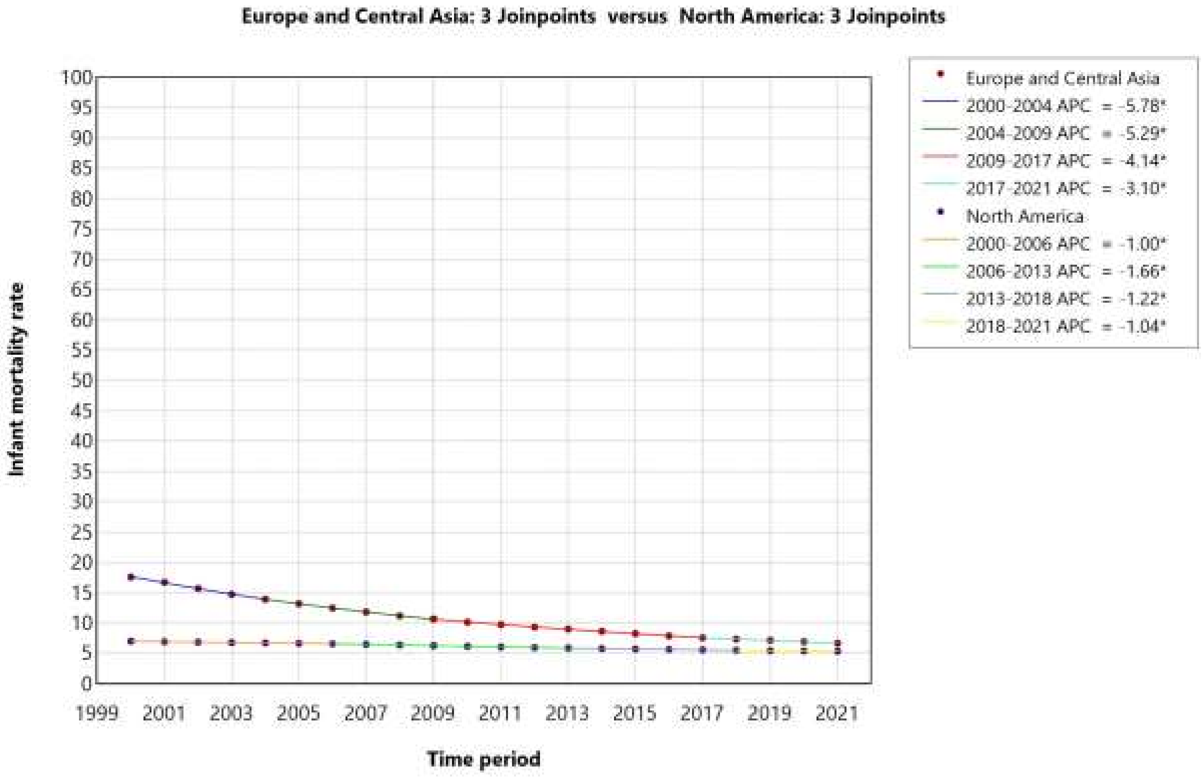

* Indicates that the Annual Percent Change (APC) is significantly different from zero at the alpha = 0.05 level.
Final Selected Model: Europe and Central Asia - 3 Joinpoints, North America - 3 Joinpoints. Rejected Parallelism.

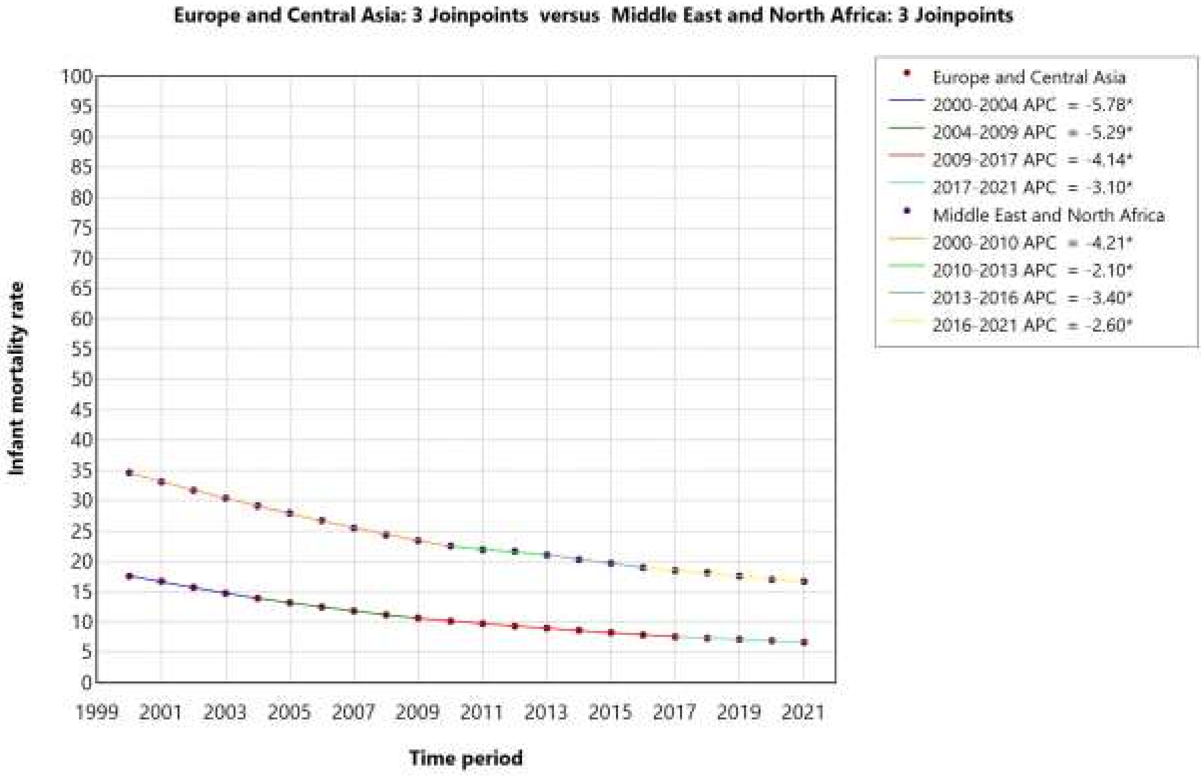

* Indicates that the Annual Percent Change (APC) is significantly different from zero at the alpha = 0.05 level.
Final Selected Model: Europe and Central Asia - 3 Joinpoints, Middle East and North Africa - 3 Joinpoints. Rejected Parallelism.

Europe and Central Asia: 3 Joinpoints versus Latin America and the Caribbean: 2 Joinpoints

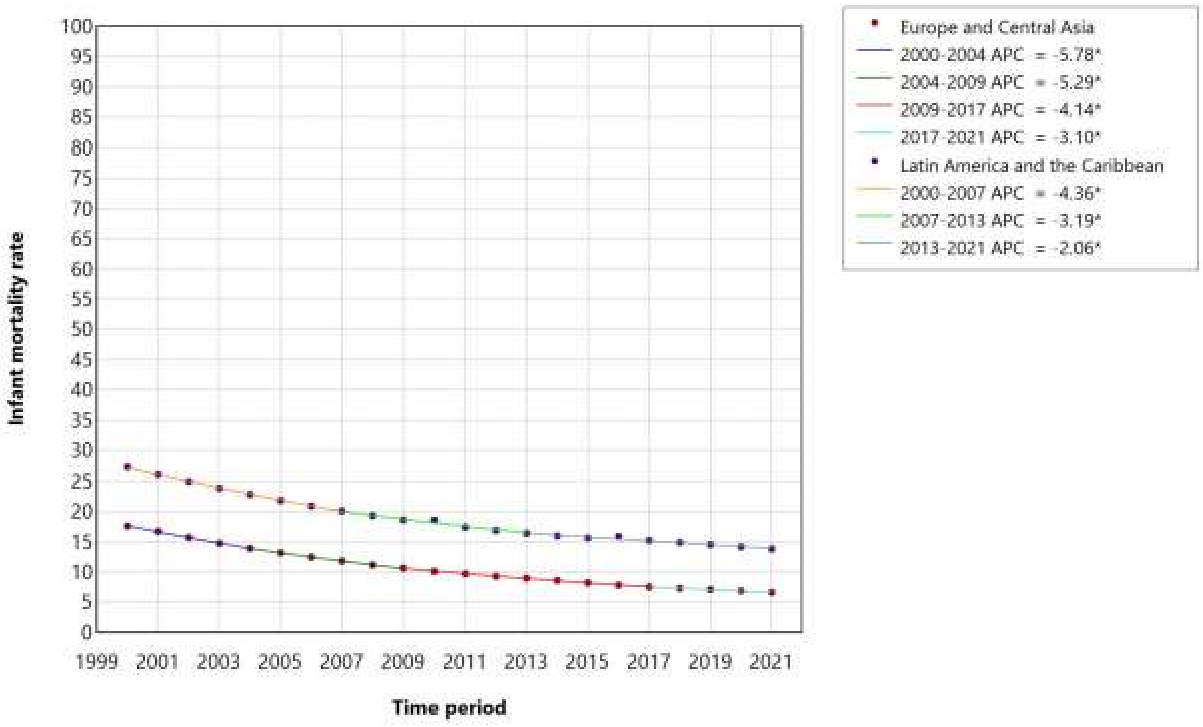

* Indicates that the Annual Percent Change (APC) is significantly different from zero at the alpha = 0.05 level.
Final Selected Model: Europe and Central Asia - 3 Joinpoints, Latin America and the Caribbean - 2 Joinpoints. Rejected Parallelism.

Eastern Europe and Central Asia: 2 Joinpoints versus Western Europe: 4 Joinpoints

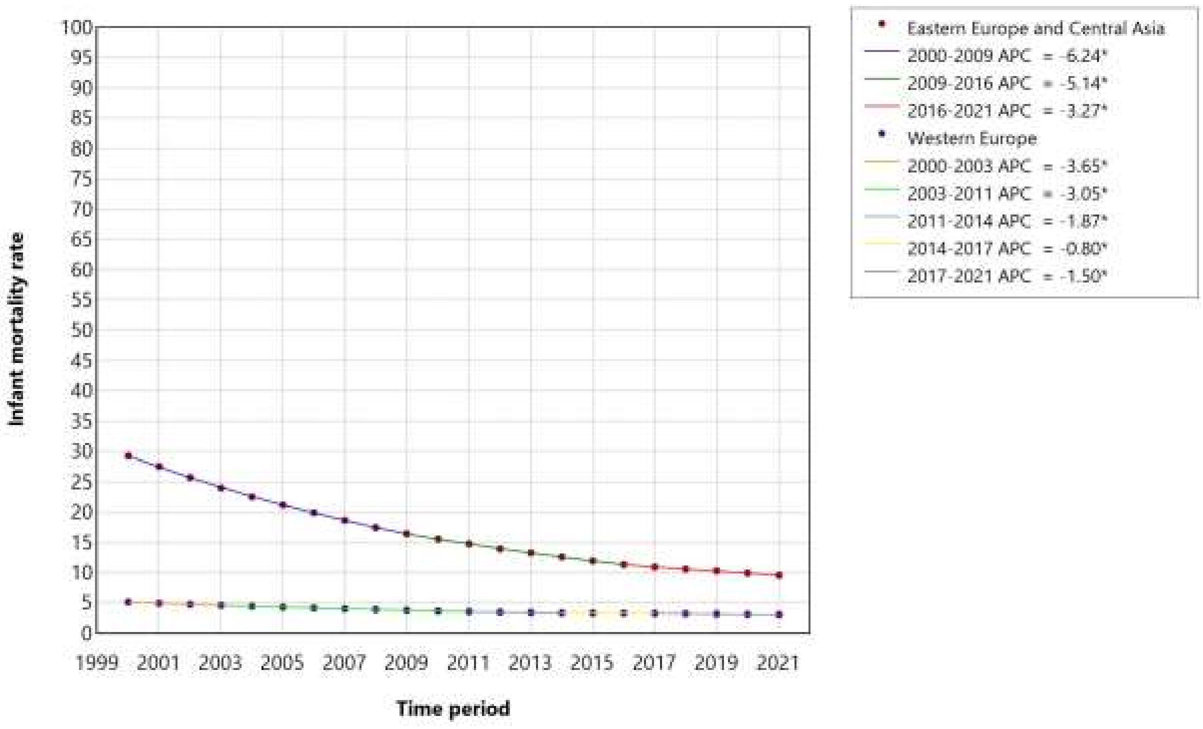

* Indicates that the Annual Percent Change (APC) is significantly different from zero at the alpha = 0.05 level.
Final Selected Model: Eastern Europe and Central Asia - 2 Joinpoints, Western Europe - 4 Joinpoints. Rejected Parallelism.

Eastern Europe and Central Asia: 2 Joinpoints versus West and Central Africa: 5 Joinpoints

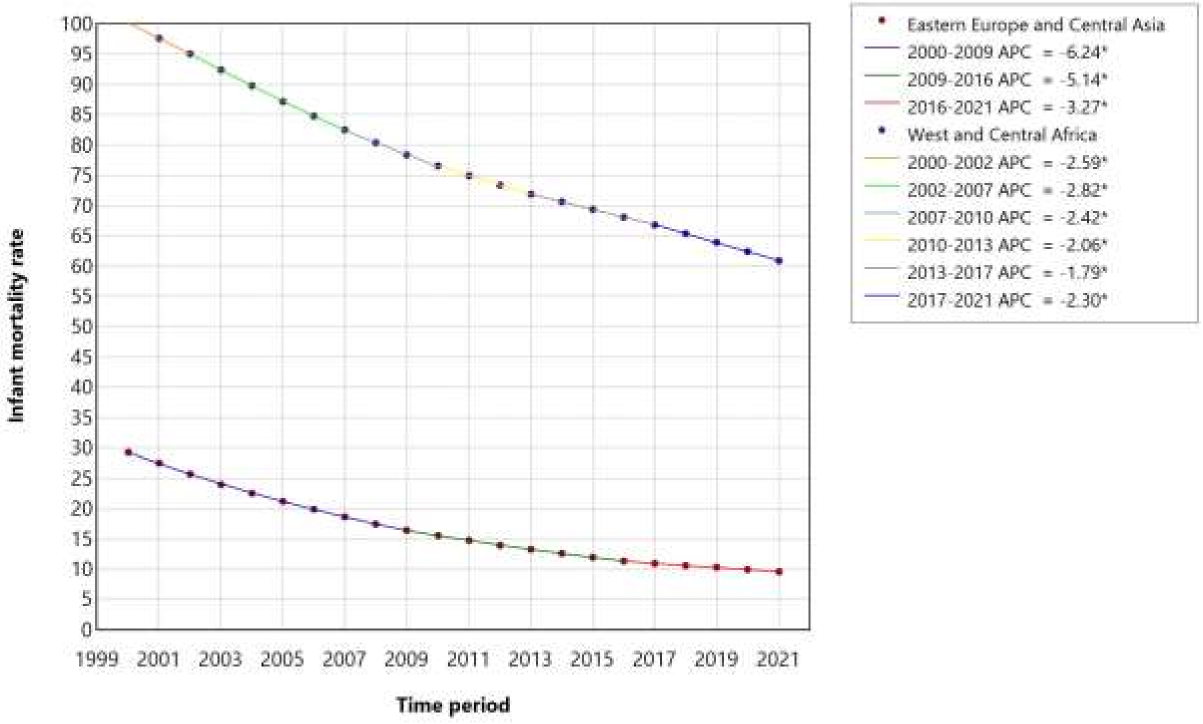

* Indicates that the Annual Percent Change (APC) is significantly different from zero at the alpha = 0.05 level.
Final Selected Model: Eastern Europe and Central Asia - 2 Joinpoints, West and Central Africa - 5 Joinpoints. Rejected Parallelism.

Eastern Europe and Central Asia: 2 Joinpoints versus Sub-Saharan Africa: 5 Joinpoints

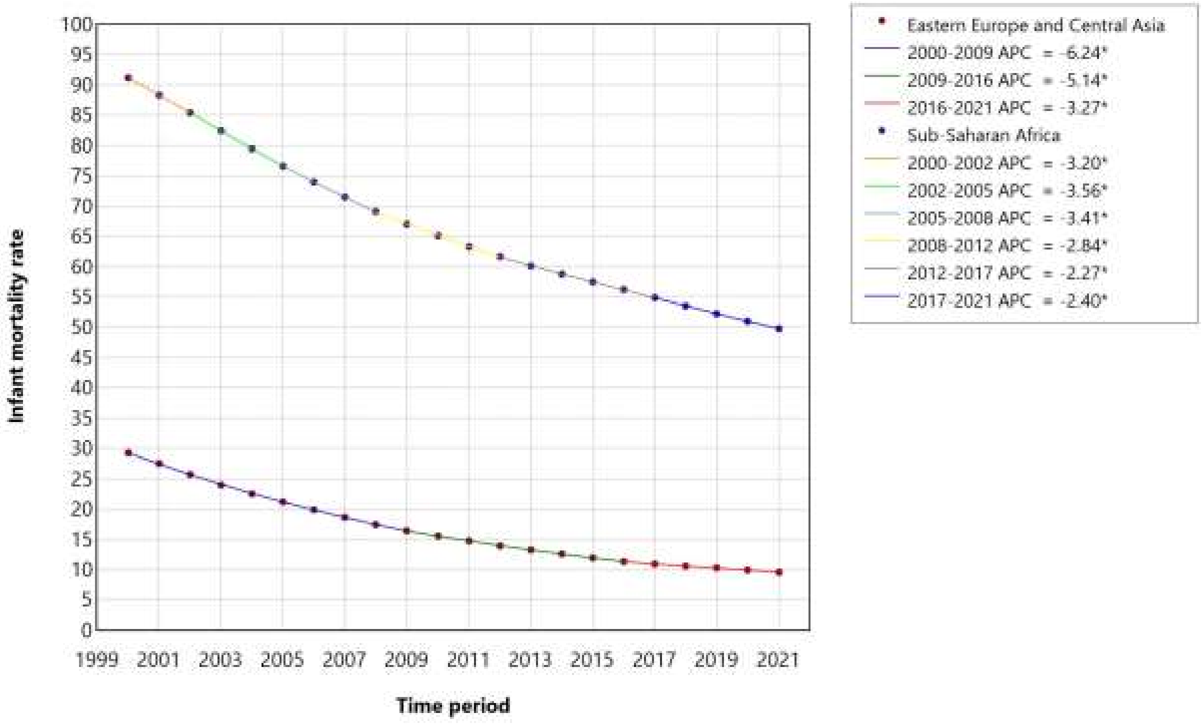

* Indicates that the Annual Percent Change (APC) is significantly different from zero at the alpha = 0.05 level.
Final Selected Model: Eastern Europe and Central Asia - 2 Joinpoints, Sub-Saharan Africa - 5 Joinpoints. Rejected Parallelism.

Eastern Europe and Central Asia: 2 Joinpoints versus South Asia: 4 Joinpoints

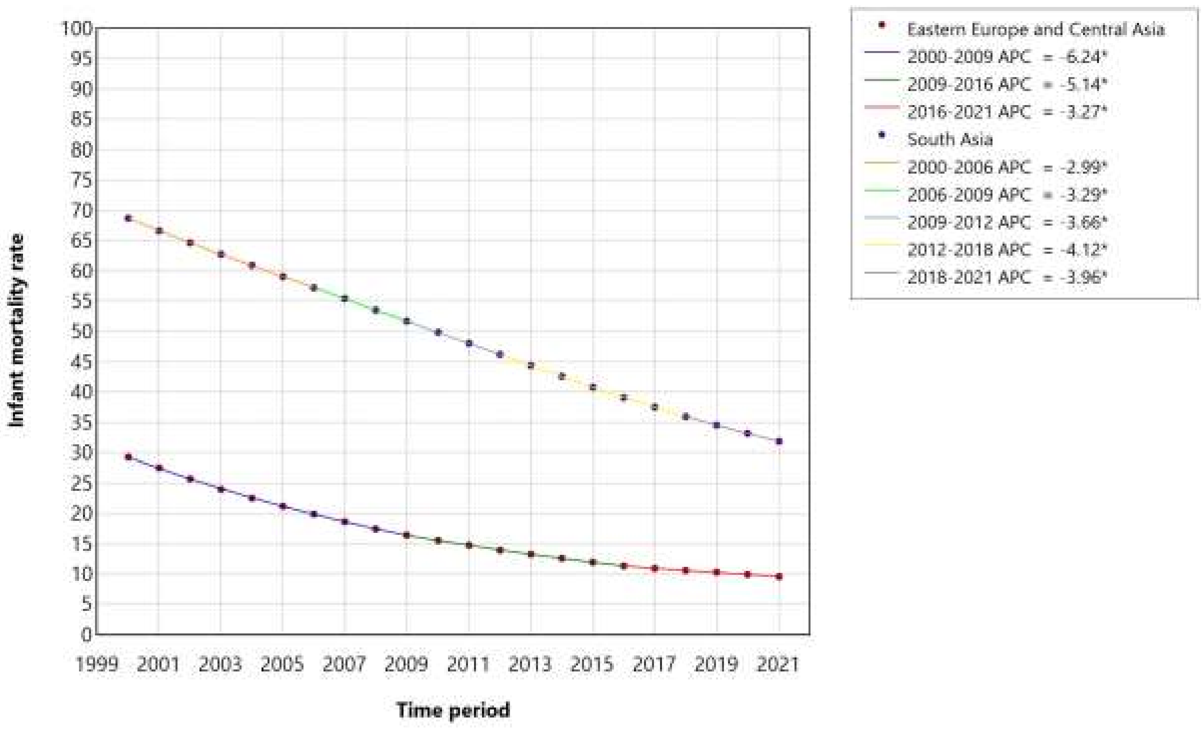

* Indicates that the Annual Percent Change (APC) is significantly different from zero at the alpha = 0.05 level.
Final Selected Model: Eastern Europe and Central Asia - 2 Joinpoints, South Asia - 4 Joinpoints. Rejected Parallelism.

Eastern Europe and Central Asia: 2 Joinpoints versus North America: 3 Joinpoints

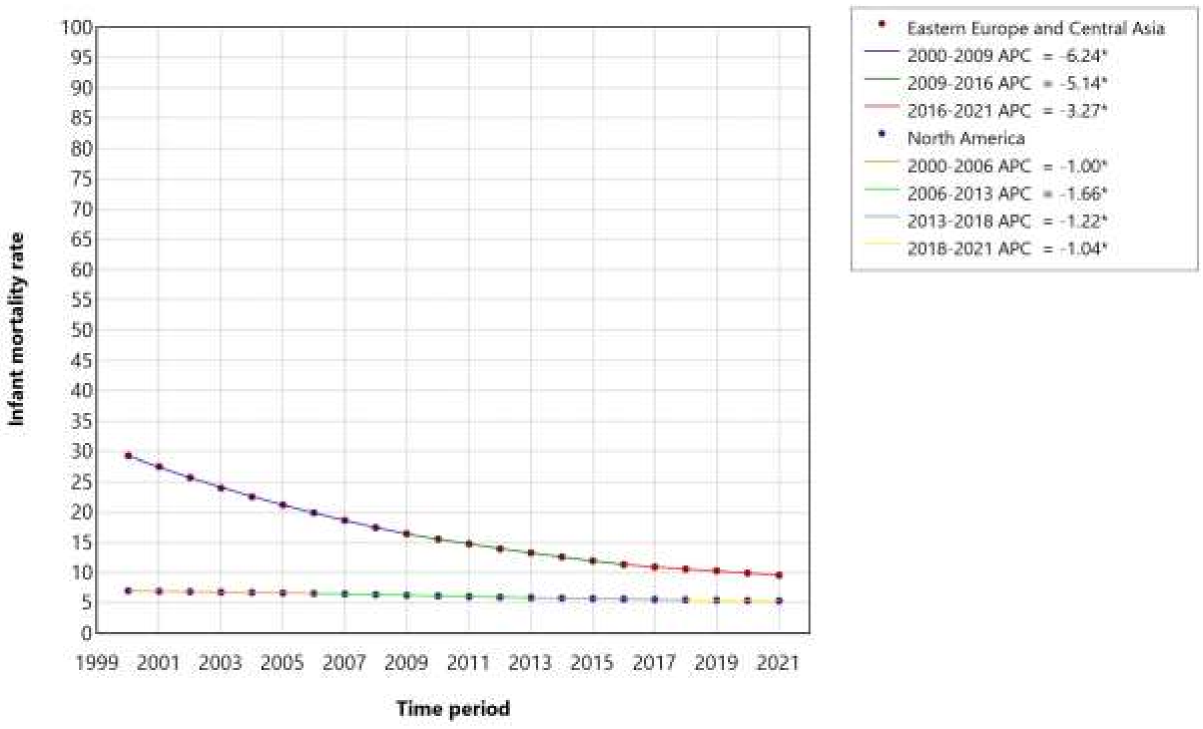

* Indicates that the Annual Percent Change (APC) is significantly different from zero at the alpha = 0.05 level.
Final Selected Model: Eastern Europe and Central Asia - 2 Joinpoints, North America - 3 Joinpoints. Rejected Parallelism.

Eastern Europe and Central Asia: 2 Joinpoints versus Middle East and North Africa: 3 Joinpoints

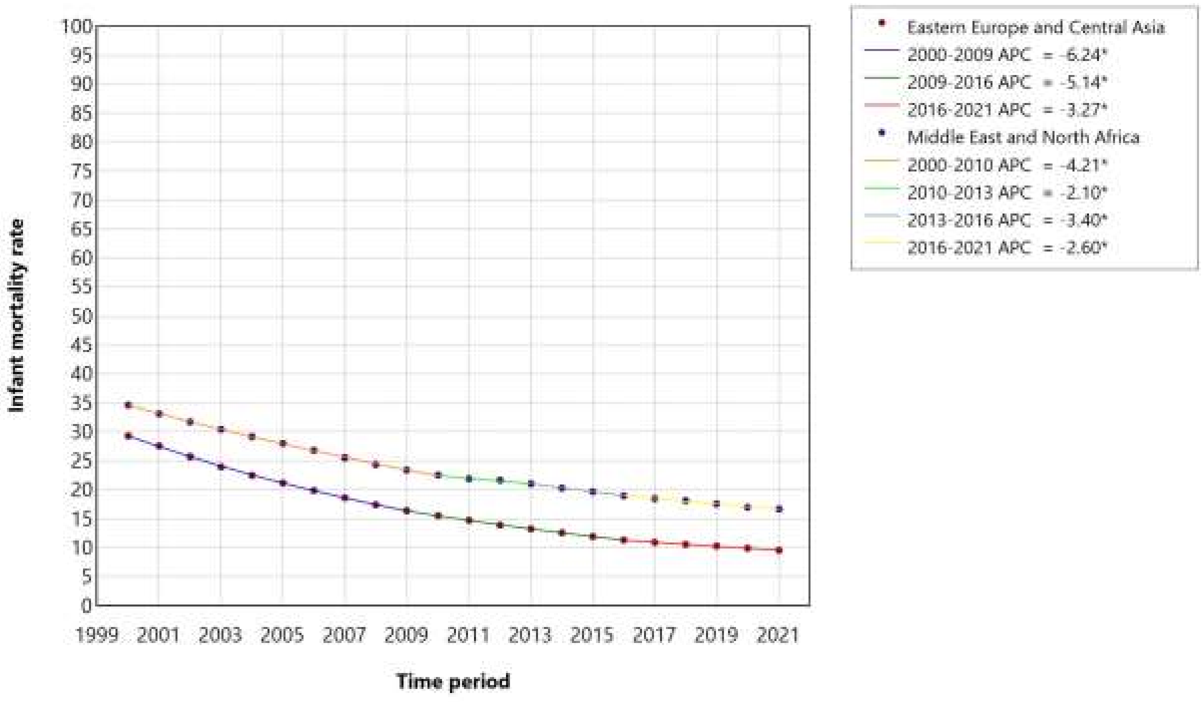

* Indicates that the Annual Percent Change (APC) is significantly different from zero at the alpha = 0.05 level.
Final Selected Model: Eastern Europe and Central Asia - 2 Joinpoints, Middle East and North Africa - 3 Joinpoints. Rejected Parallelism.

Eastern Europe and Central Asia: 2 Joinpoints versus Latin America and the Caribbean: 2 Joinpoints

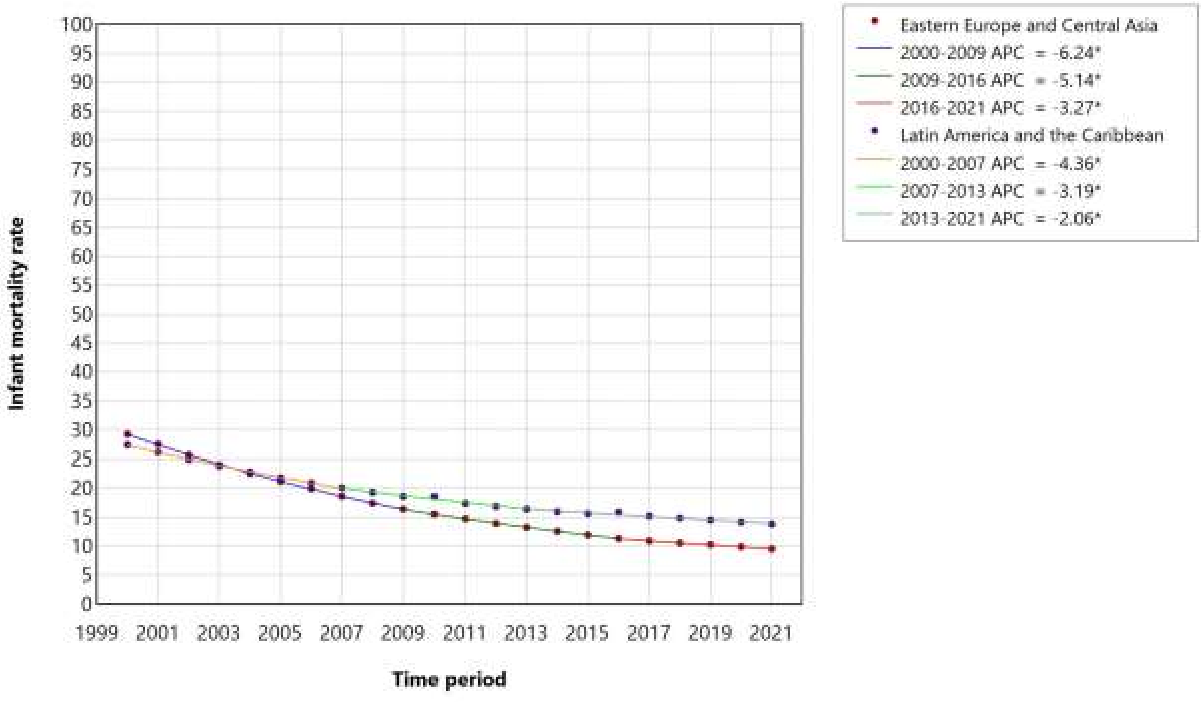

* Indicates that the Annual Percent Change (APC) is significantly different from zero at the alpha = 0.05 level.
Final Selected Model: Eastern Europe and Central Asia - 2 Joinpoints, Latin America and the Caribbean - 2 Joinpoints. Rejected Parallelism.

Eastern Europe and Central Asia: 2 Joinpoints versus Europe and Central Asia: 3 Joinpoints

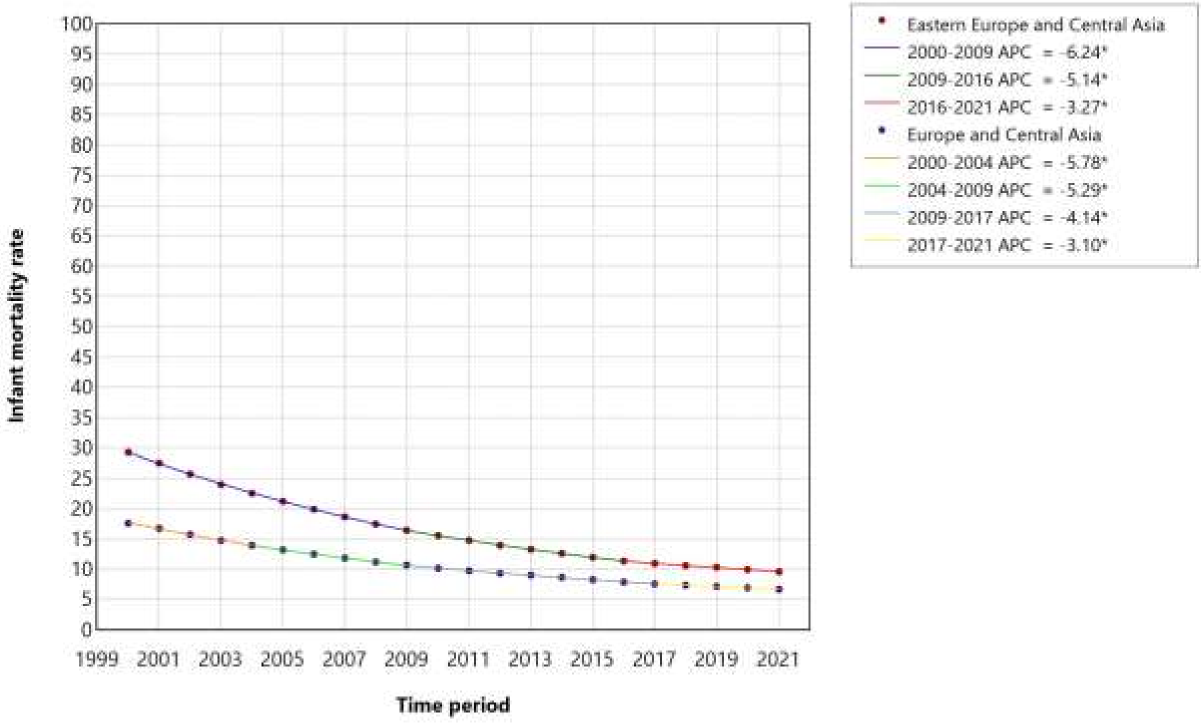

* Indicates that the Annual Percent Change (APC) is significantly different from zero at the alpha = 0.05 level.
Final Selected Model: Eastern Europe and Central Asia - 2 Joinpoints, Europe and Central Asia - 3 Joinpoints. Rejected Parallelism.

Eastern and Southern Africa: 4 Joinpoints versus Western Europe: 4 Joinpoints

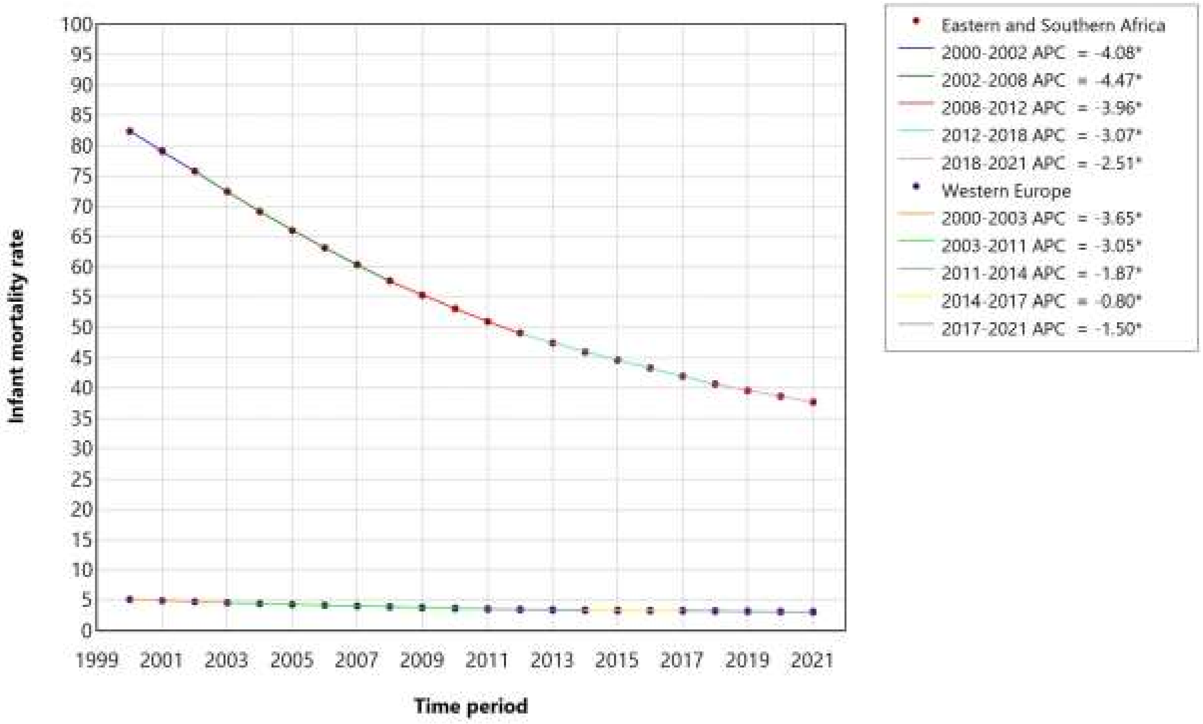

* Indicates that the Annual Percent Change (APC) is significantly different from zero at the alpha = 0.05 level.
Final Selected Model: Eastern and Southern Africa - 4 Joinpoints, Western Europe - 4 Joinpoints. Rejected Parallelism.

Eastern and Southern Africa: 4 Joinpoints versus West and Central Africa: 5 Joinpoints

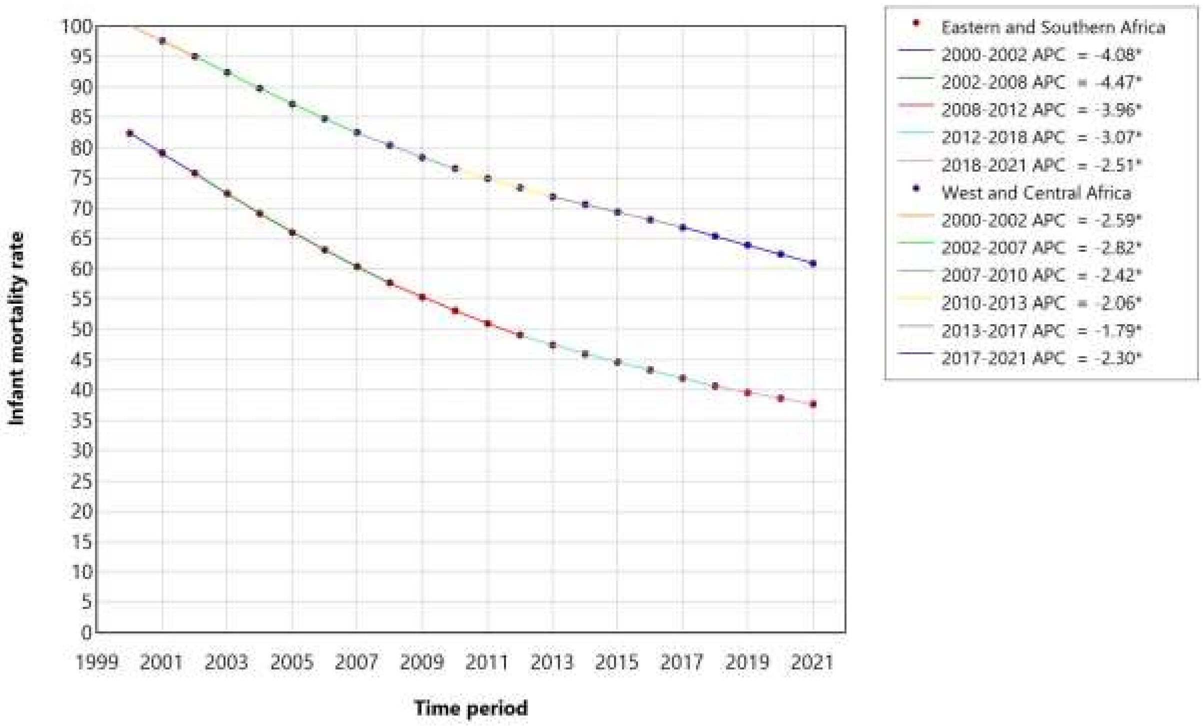

* Indicates that the Annual Percent Change (APC) is significantly different from zero at the alpha = 0.05 level.
Final Selected Model: Eastern and Southern Africa - 4 Joinpoints, West and Central Africa - 5 Joinpoints. Rejected Parallelism.

Eastern and Southern Africa: 4 Joinpoints versus Sub-Saharan Africa: 5 Joinpoints

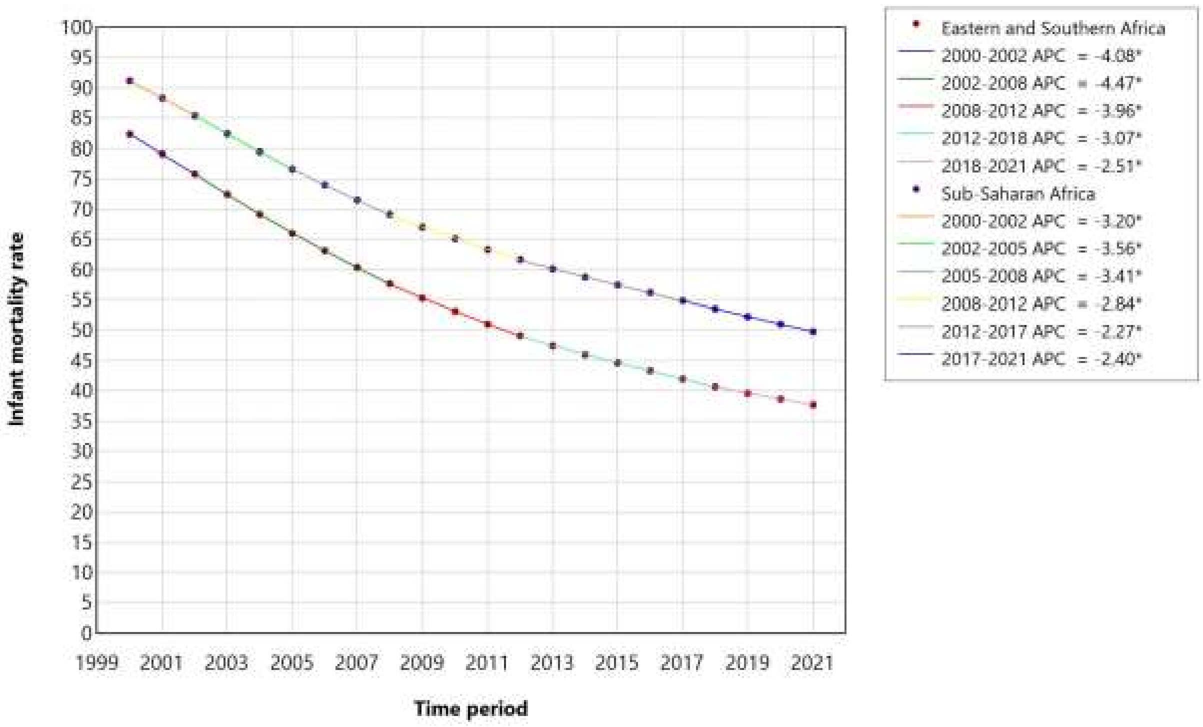

* Indicates that the Annual Percent Change (APC) is significantly different from zero at the alpha = 0.05 level.
Final Selected Model: Eastern and Southern Africa - 4 Joinpoints, Sub-Saharan Africa - 5 Joinpoints. Rejected Parallelism.

Eastern and Southern Africa: 4 Joinpoints versus South Asia: 4 Joinpoints

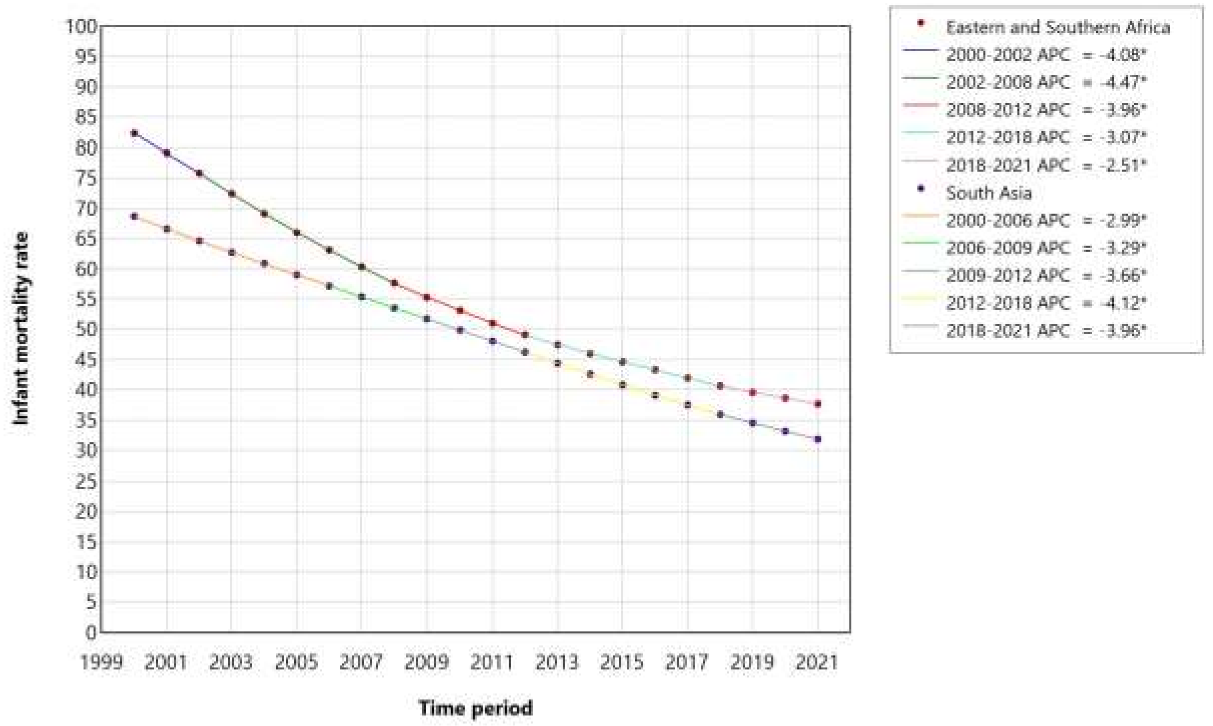

* Indicates that the Annual Percent Change (APC) is significantly different from zero at the alpha = 0.05 level.
Final Selected Model: Eastern and Southern Africa - 4 Joinpoints, South Asia - 4 Joinpoints. Rejected Parallelism.

Eastern and Southern Africa: 4 Joinpoints versus North America: 3 Joinpoints

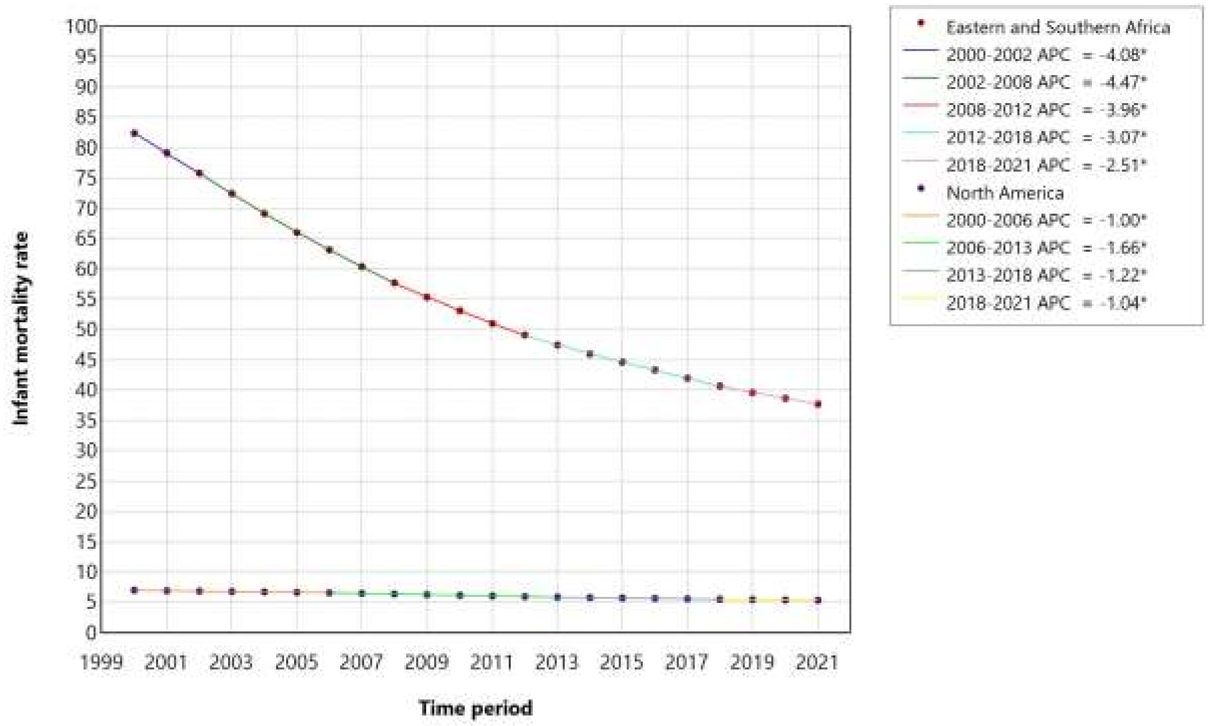

* Indicates that the Annual Percent Change (APC) is significantly different from zero at the alpha = 0.05 level.
Final Selected Model: Eastern and Southern Africa - 4 Joinpoints, North America - 3 Joinpoints. Rejected Parallelism.

Eastern and Southern Africa: 4 Joinpoints versus Middle East and North Africa: 3 Joinpoints

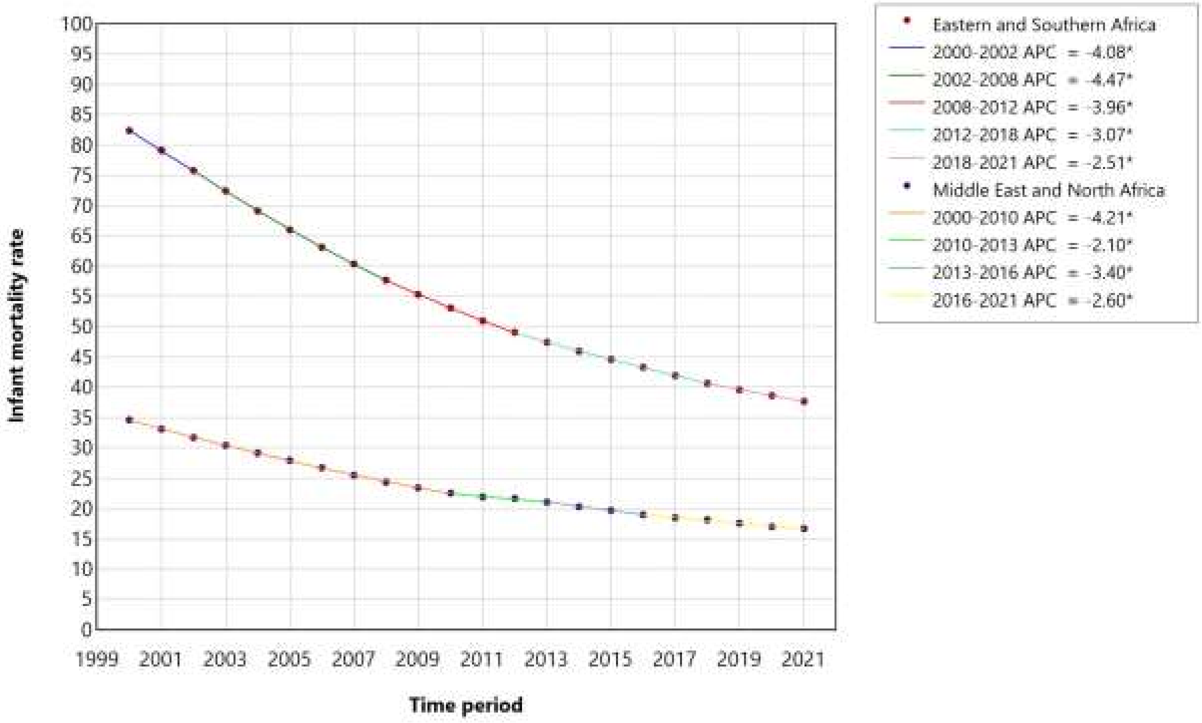

* Indicates that the Annual Percent Change (APC) is significantly different from zero at the alpha = 0.05 level.
Final Selected Model: Eastern and Southern Africa - 4 Joinpoints, Middle East and North Africa - 3 Joinpoints. Rejected Parallelism.

Eastern and Southern Africa: 4 Joinpoints versus Latin America and the Caribbean: 2 Joinpoints

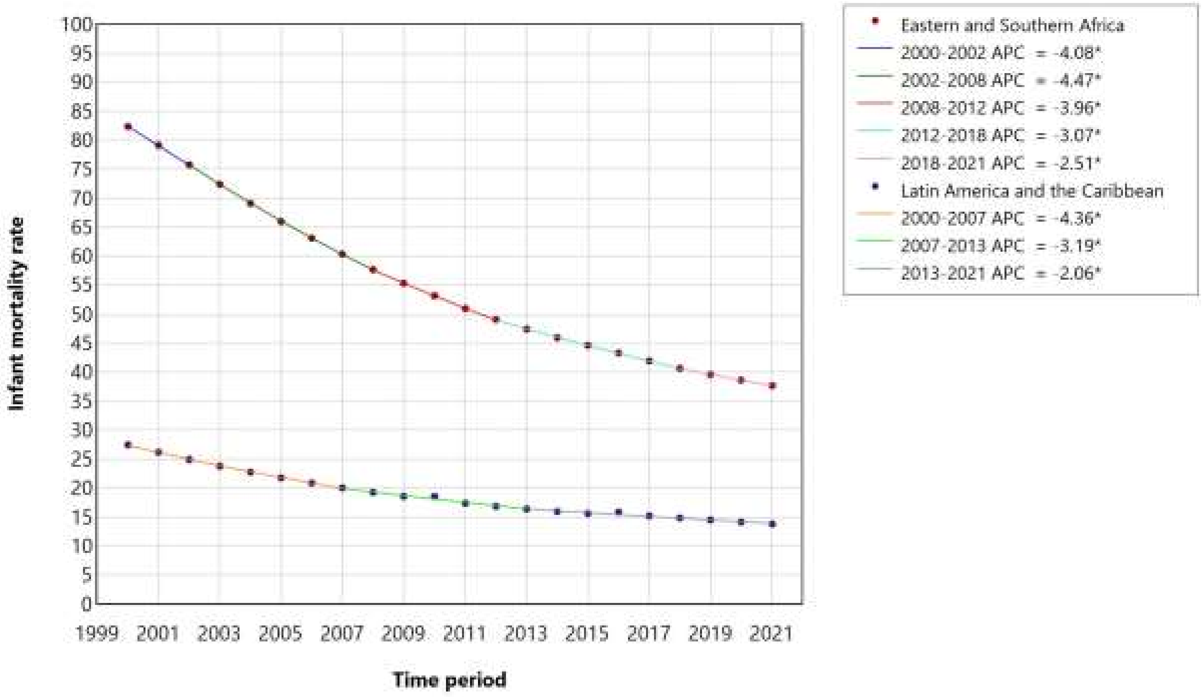

* Indicates that the Annual Percent Change (APC) is significantly different from zero at the alpha = 0.05 level.
Final Selected Model: Eastern and Southern Africa - 4 Joinpoints, Latin America and the Caribbean - 2 Joinpoints. Rejected Parallelism.

Eastern and Southern Africa: 4 Joinpoints versus Europe and Central Asia: 3 Joinpoints

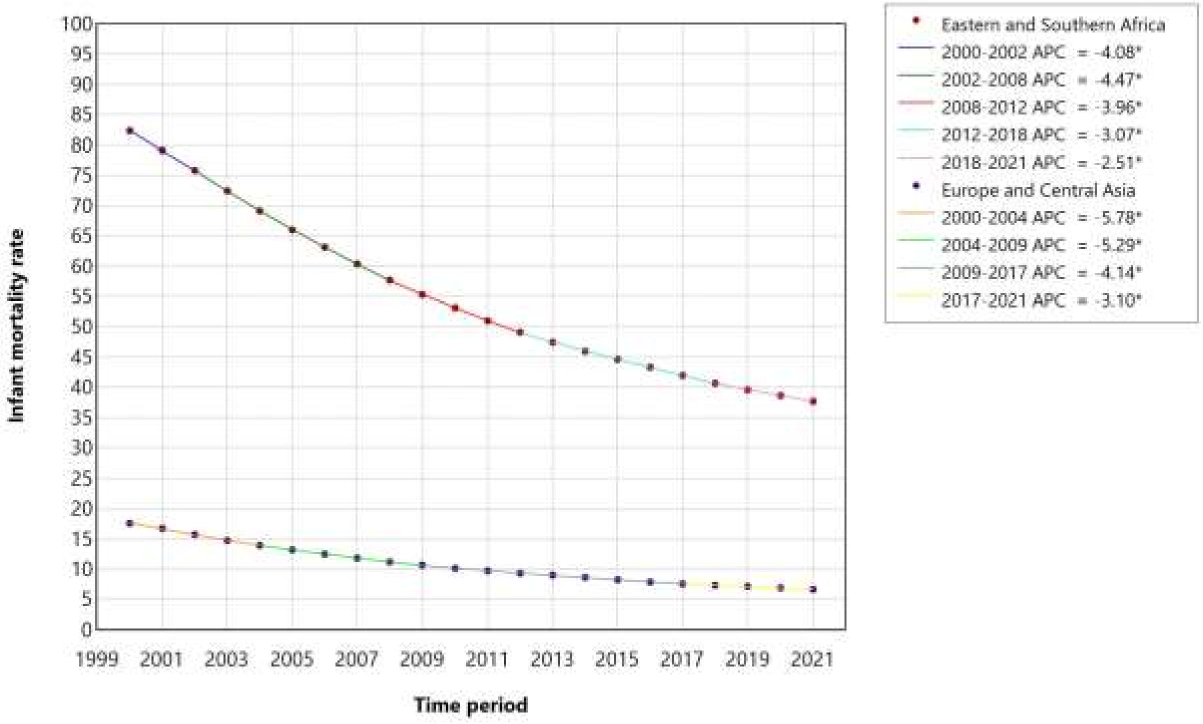

* Indicates that the Annual Percent Change (APC) is significantly different from zero at the alpha = 0.05 level.
Final Selected Model: Eastern and Southern Africa - 4 Joinpoints, Europe and Central Asia - 3 Joinpoints. Rejected Parallelism.

Eastern and Southern Africa: 4 Joinpoints versus Eastern Europe and Central Asia: 2 Joinpoints

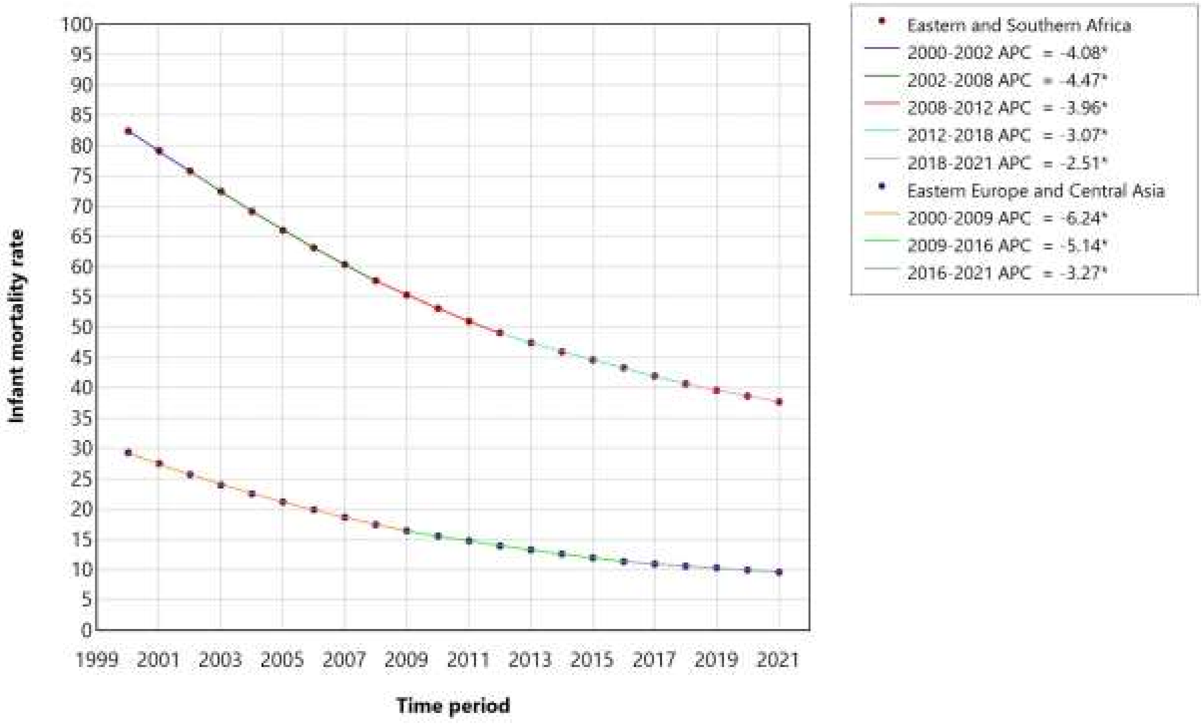

* Indicates that the Annual Percent Change (APC) is significantly different from zero at the alpha = 0.05 level.
Final Selected Model: Eastern and Southern Africa - 4 Joinpoints, Eastern Europe and Central Asia - 2 Joinpoints. Rejected Parallelism.

East Asia and Pacific: 2 Joinpoints versus Western Europe: 4 Joinpoints

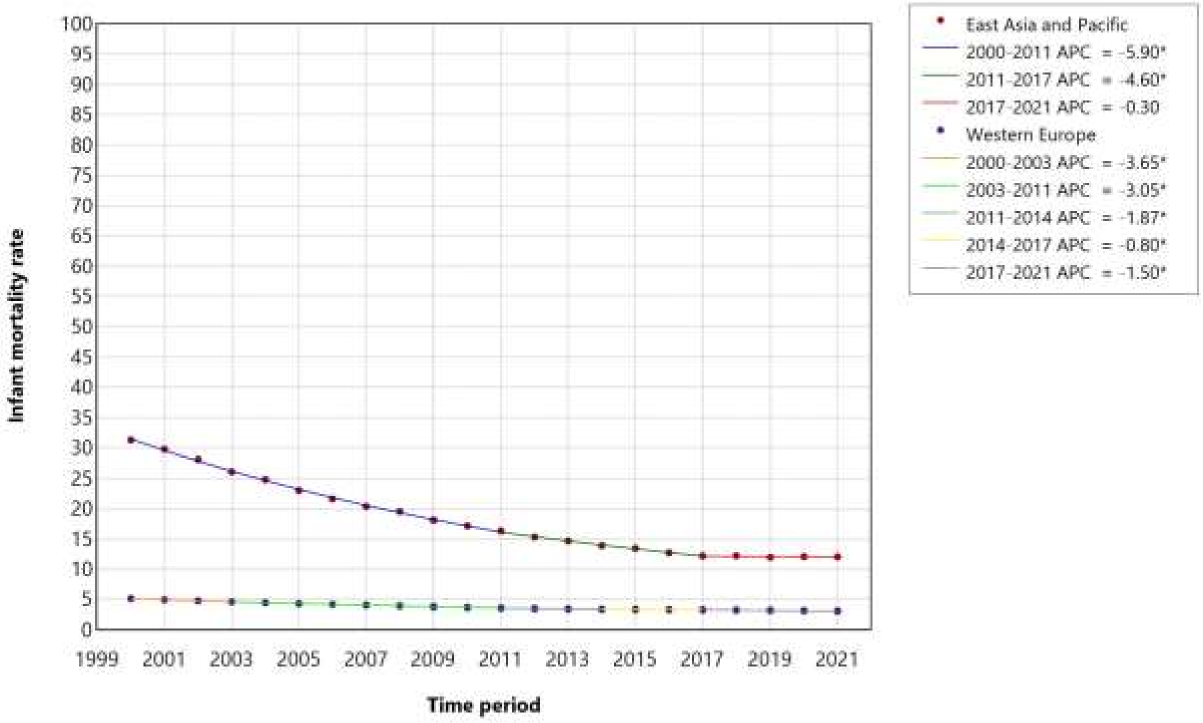

* Indicates that the Annual Percent Change (APC) is significantly different from zero at the alpha = 0.05 level.
Final Selected Model: East Asia and Pacific - 2 Joinpoints, Western Europe - 4 Joinpoints. Rejected Parallelism.

East Asia and Pacific: 2 Joinpoints versus West and Central Africa: 5 Joinpoints

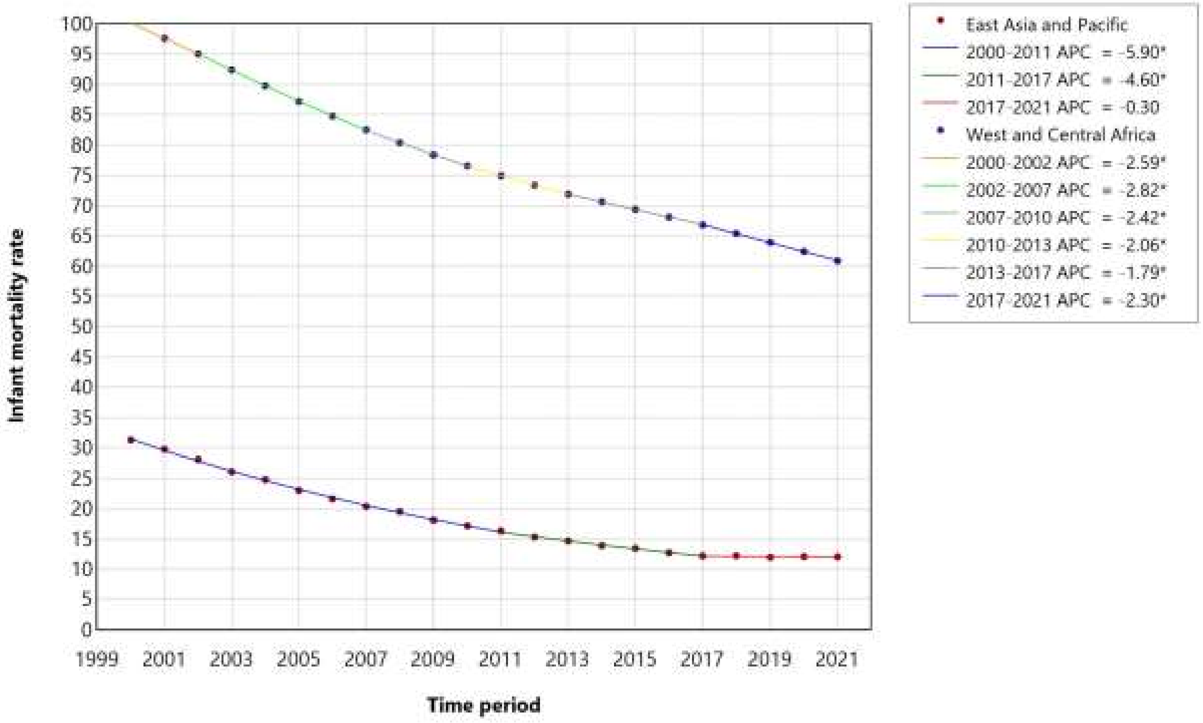

* Indicates that the Annual Percent Change (APC) is significantly different from zero at the alpha = 0.05 level.
Final Selected Model: East Asia and Pacific - 2 Joinpoints, West and Central Africa - 5 Joinpoints. Rejected Parallelism.

East Asia and Pacific: 2 Joinpoints versus Sub-Saharan Africa: 5 Joinpoints

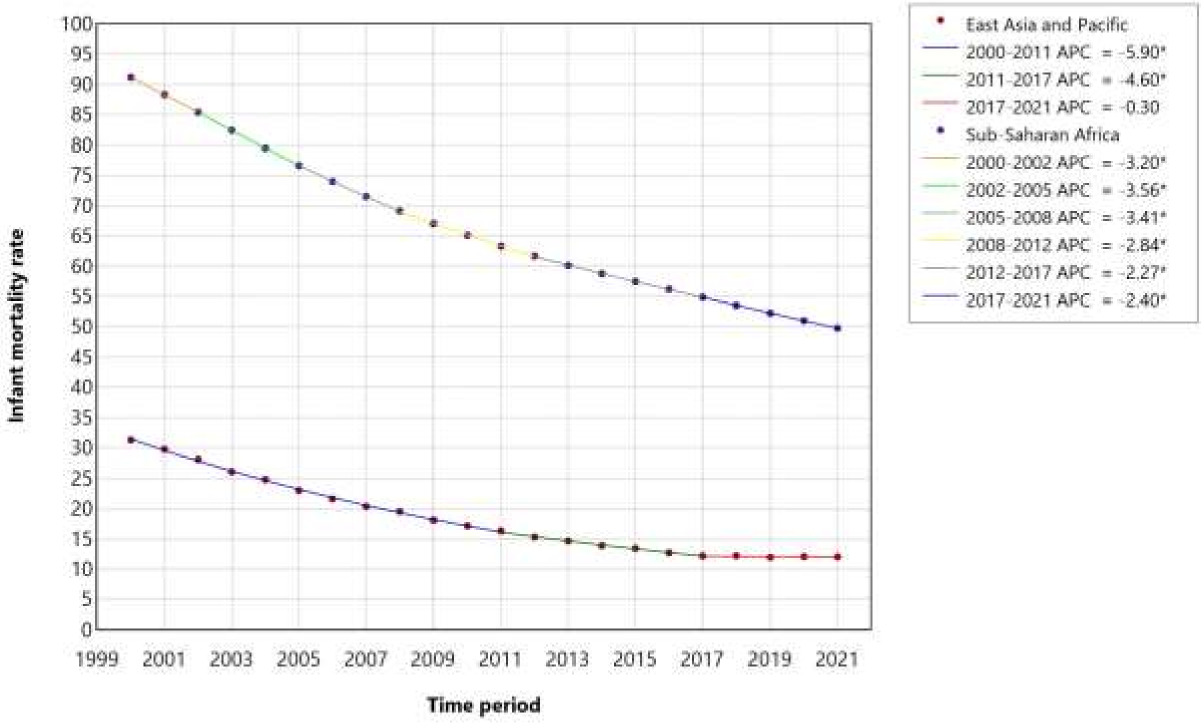

* Indicates that the Annual Percent Change (APC) is significantly different from zero at the alpha = 0.05 level.
Final Selected Model: East Asia and Pacific - 2 Joinpoints, Sub-Saharan Africa - 5 Joinpoints. Rejected Parallelism.

East Asia and Pacific: 2 Joinpoints versus South Asia: 4 Joinpoints

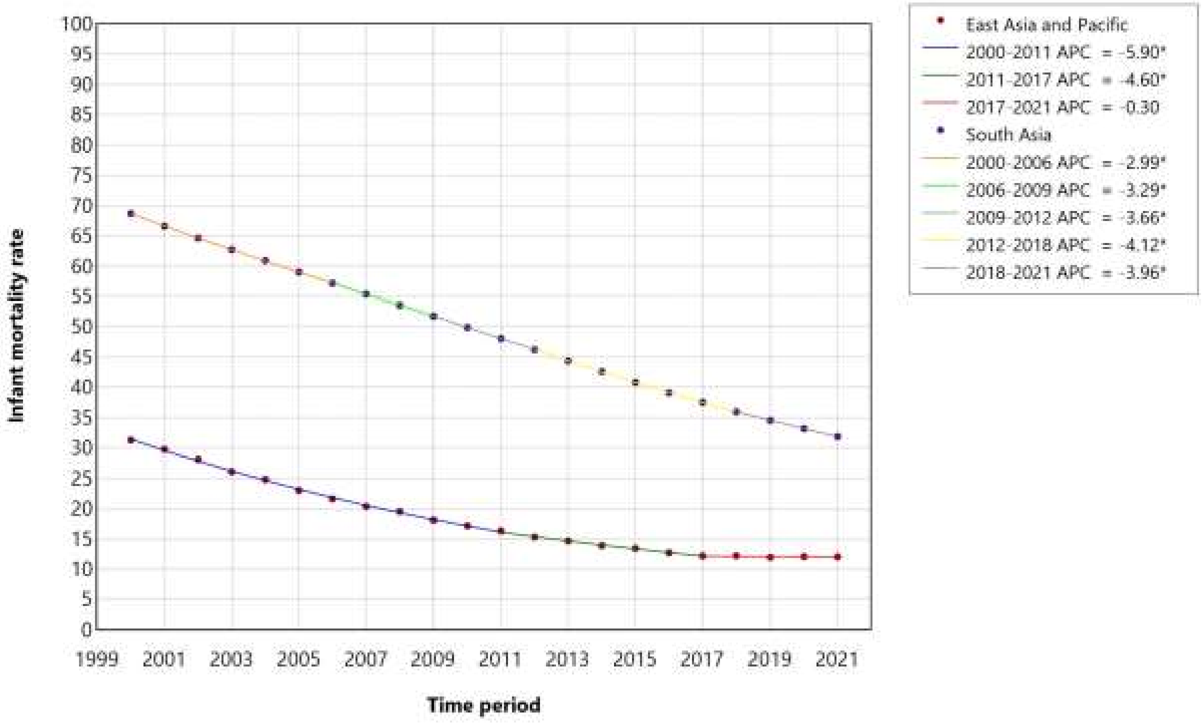

* Indicates that the Annual Percent Change (APC) is significantly different from zero at the alpha = 0.05 level.
Final Selected Model: East Asia and Pacific - 2 Joinpoints, South Asia - 4 Joinpoints. Rejected Parallelism.

East Asia and Pacific: 2 Joinpoints versus North America: 3 Joinpoints

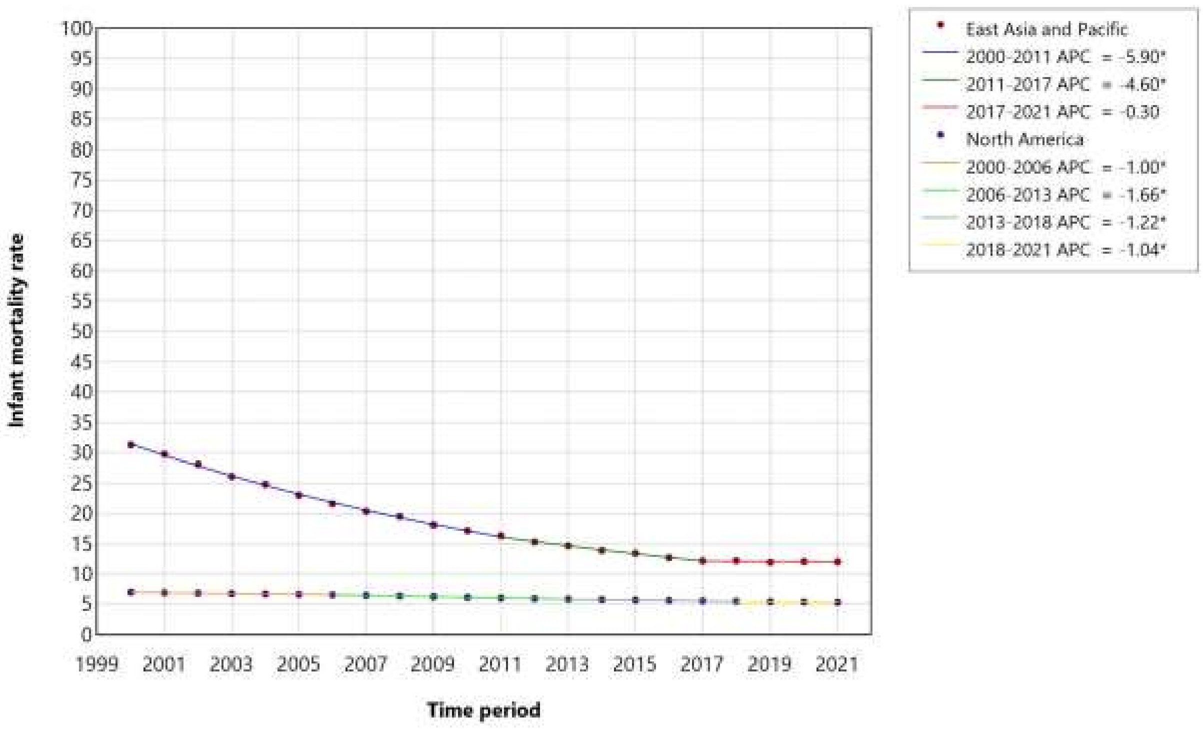

* Indicates that the Annual Percent Change (APC) is significantly different from zero at the alpha = 0.05 level.
Final Selected Model: East Asia and Pacific - 2 Joinpoints, North America - 3 Joinpoints. Rejected Parallelism.

East Asia and Pacific: 2 Joinpoints versus Middle East and North Africa: 3 Joinpoints

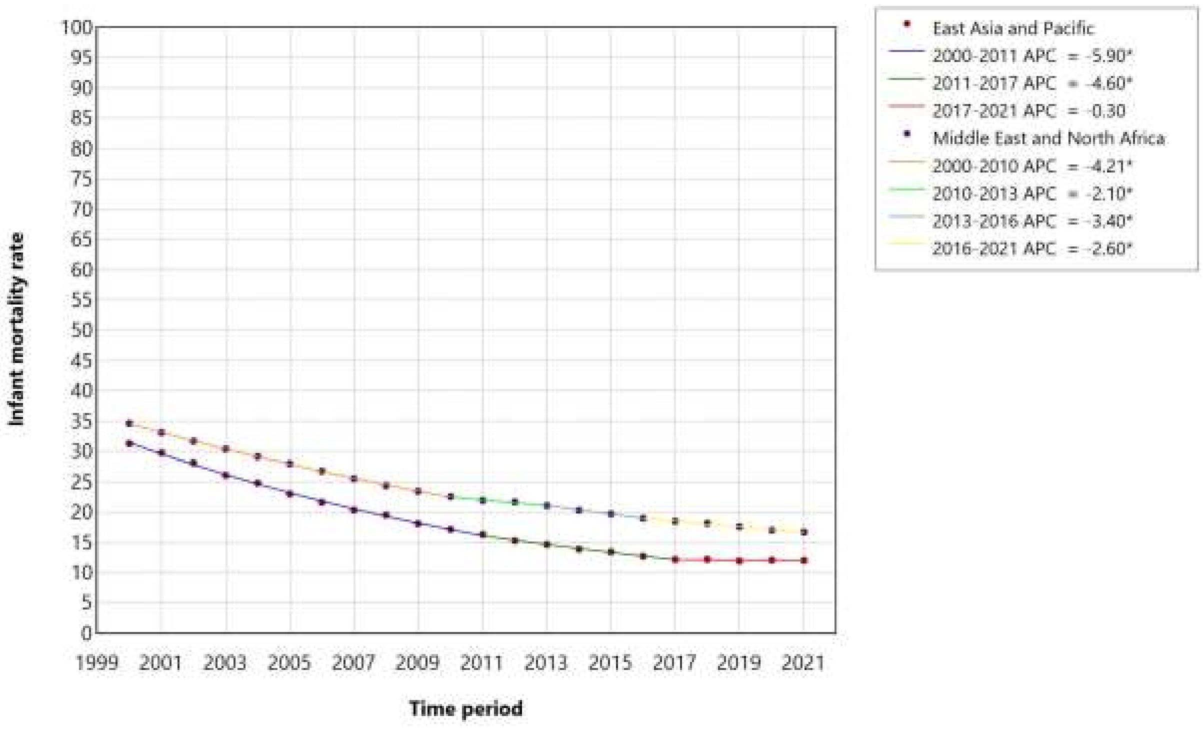

* Indicates that the Annual Percent Change (APC) is significantly different from zero at the alpha = 0.05 level.
Final Selected Model: East Asia and Pacific - 2 Joinpoints, Middle East and North Africa - 3 Joinpoints. Rejected Parallelism.

East Asia and Pacific: 2 Joinpoints versus Latin America and the Caribbean: 2 Joinpoints

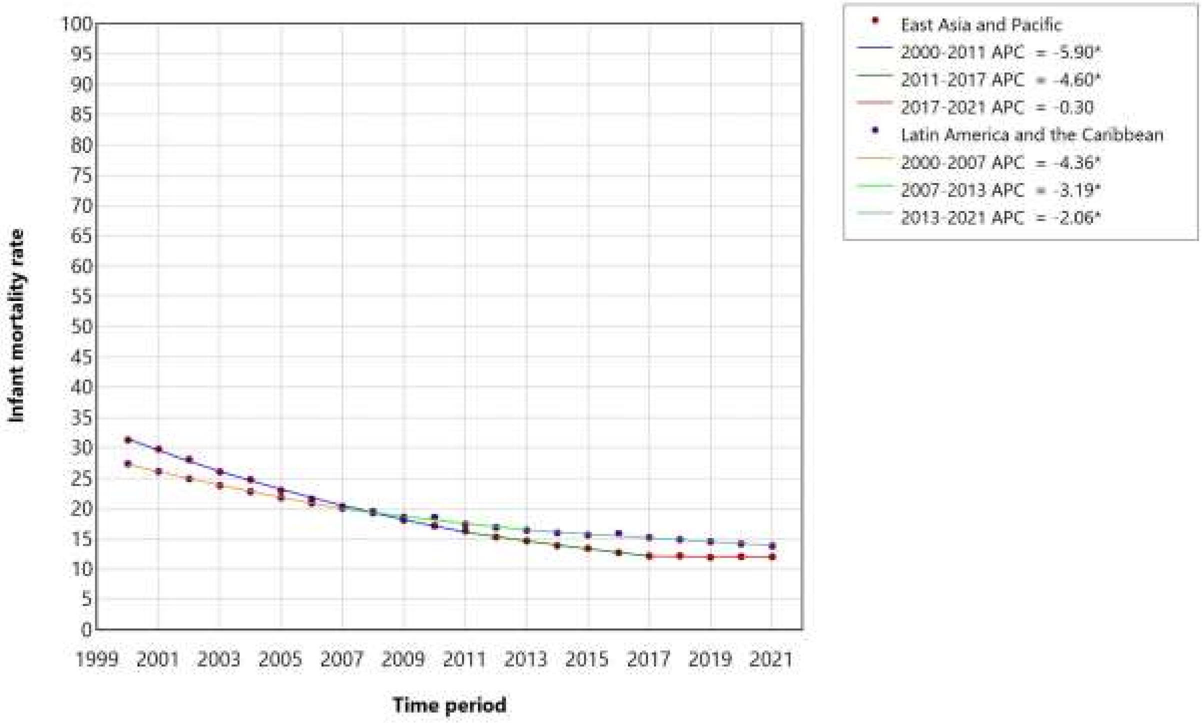

* Indicates that the Annual Percent Change (APC) is significantly different from zero at the alpha = 0.05 level.
Final Selected Model: East Asia and Pacific - 2 Joinpoints, Latin America and the Caribbean - 2 Joinpoints. Rejected Parallelism.

East Asia and Pacific: 2 Joinpoints versus Europe and Central Asia: 3 Joinpoints

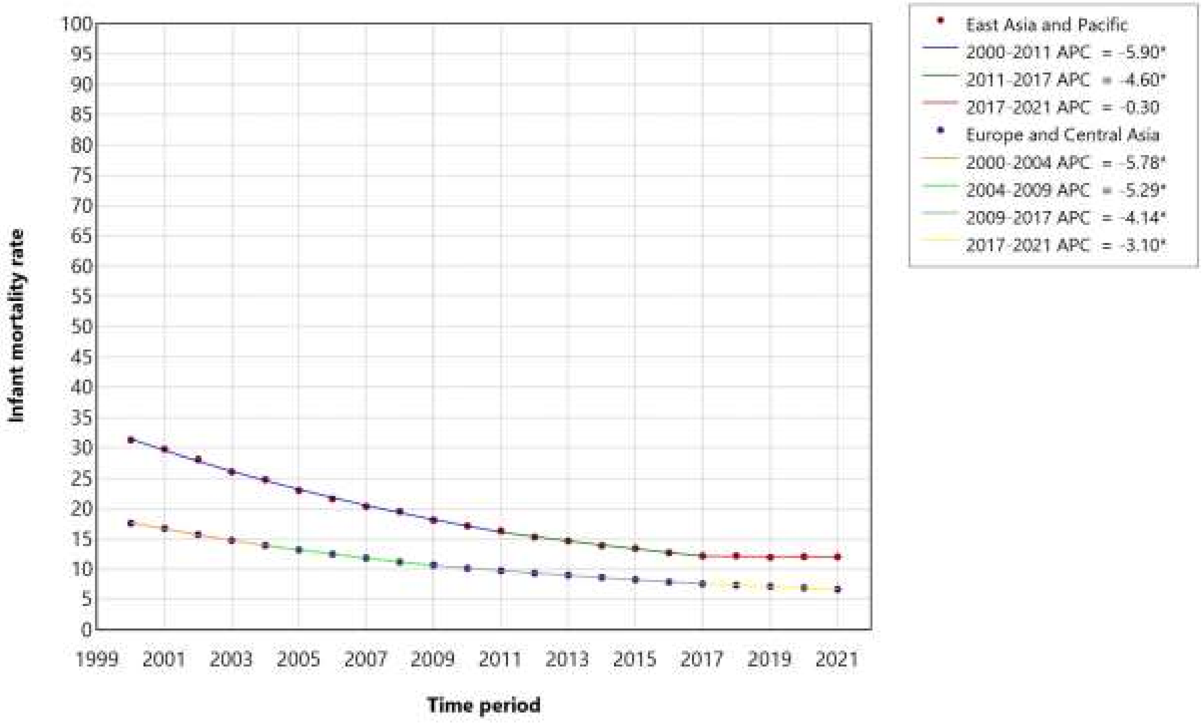

* Indicates that the Annual Percent Change (APC) is significantly different from zero at the alpha = 0.05 level.
Final Selected Model: East Asia and Pacific - 2 Joinpoints, Europe and Central Asia - 3 Joinpoints. Rejected Parallelism.

East Asia and Pacific: 2 Joinpoints versus Eastern Europe and Central Asia: 2 Joinpoints

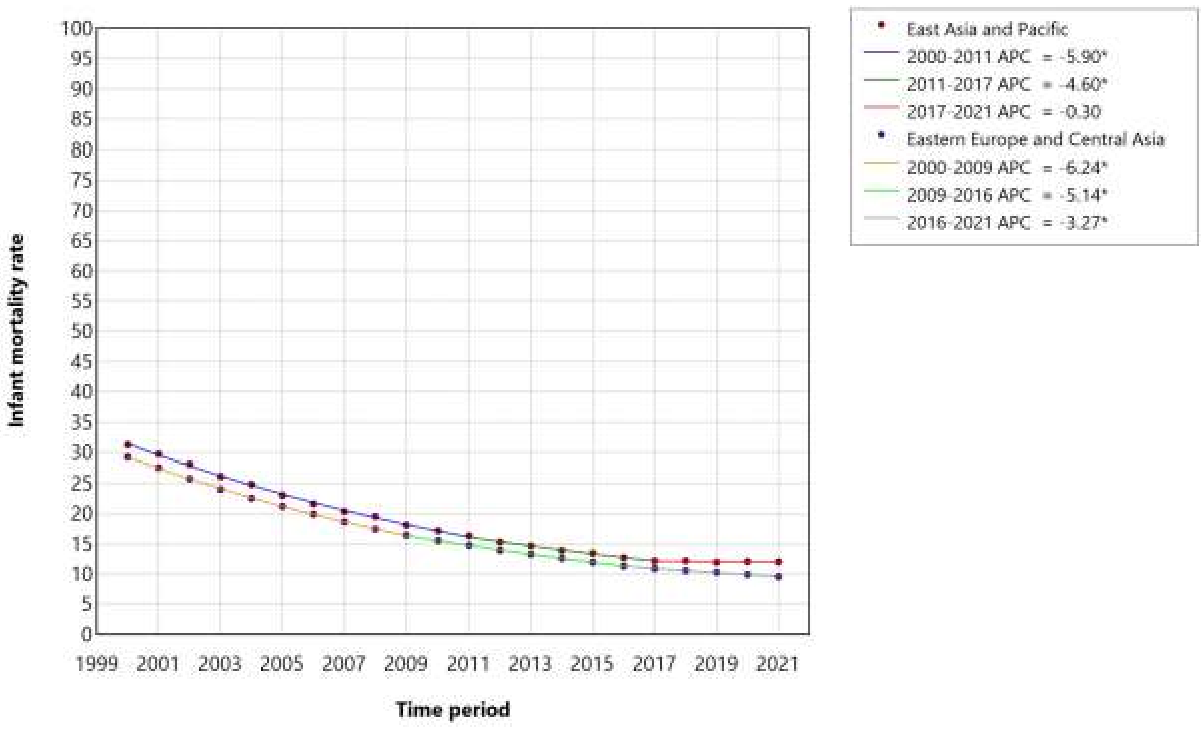

* Indicates that the Annual Percent Change (APC) is significantly different from zero at the alpha = 0.05 level.
Final Selected Model: East Asia and Pacific - 2 Joinpoints, Eastern Europe and Central Asia - 2 Joinpoints. Rejected Parallelism.

East Asia and Pacific: 2 Joinpoints versus Eastern and Southern Africa: 4 Joinpoints

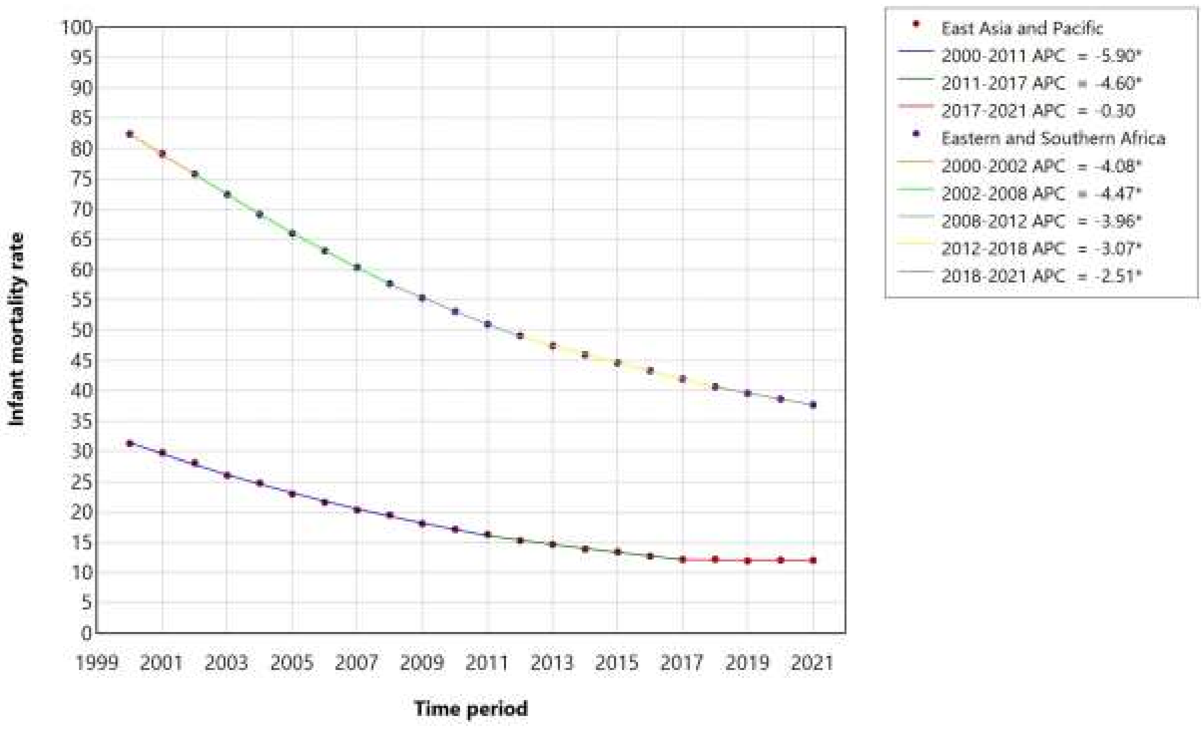

* Indicates that the Annual Percent Change (APC) is significantly different from zero at the alpha = 0.05 level.
Final Selected Model: East Asia and Pacific - 2 Joinpoints, Eastern and Southern Africa - 4 Joinpoints. Rejected Parallelism.

West and Central Africa: 5 Joinpoints versus Western Europe: 4 Joinpoints

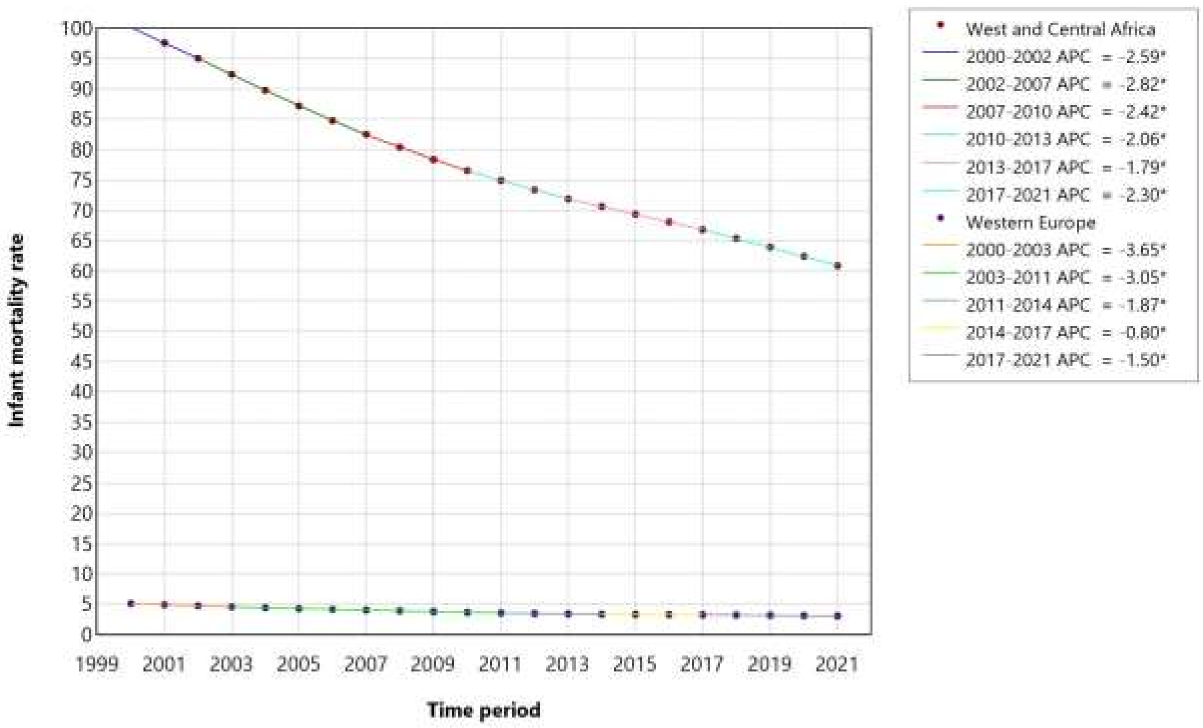

* Indicates that the Annual Percent Change (APC) is significantly different from zero at the alpha = 0.05 level.
Final Selected Model: West and Central Africa - 5 Joinpoints, Western Europe - 4 Joinpoints. Rejected Parallelism.
